# New rRNA Gene-Based Phylogenies of the *Alphaproteobacteria* Provide Perspective on Major Groups, Mitochondrial Ancestry and Phylogenetic Instability

**DOI:** 10.1371/journal.pone.0083383

**Published:** 2013-12-11

**Authors:** Matteo P. Ferla, J. Cameron Thrash, Stephen J. Giovannoni, Wayne M. Patrick

**Affiliations:** 1 Department of Biochemistry, University of Otago, Dunedin, New Zealand; 2 Department of Microbiology, Oregon State University, Corvallis, Oregon, United States of America; 3 Department of Biological Sciences, Louisiana State University, Baton Rouge, Louisiana, United States of America; J. Craig Venter Institute, United States of America

## Abstract

Bacteria in the class *Alphaproteobacteria* have a wide variety of lifestyles and physiologies. They include pathogens of humans and livestock, agriculturally valuable strains, and several highly abundant marine groups. The ancestor of mitochondria also originated in this clade. Despite significant effort to investigate the phylogeny of the *Alphaproteobacteria* with a variety of methods, there remains considerable disparity in the placement of several groups. Recent emphasis on phylogenies derived from multiple protein-coding genes remains contentious due to disagreement over appropriate gene selection and the potential influences of systematic error. We revisited previous investigations in this area using concatenated alignments of the small and large subunit (SSU and LSU) rRNA genes, as we show here that these loci have much lower GC bias than whole genomes. This approach has allowed us to update the canonical 16S rRNA gene tree of the *Alphaproteobacteria* with additional important taxa that were not previously included, and with added resolution provided by concatenating the SSU and LSU genes. We investigated the topological stability of the *Alphaproteobacteria* by varying alignment methods, rate models, taxon selection and RY-recoding to circumvent GC content bias. We also introduce RYMK-recoding and show that it avoids some of the information loss in RY-recoding. We demonstrate that the topology of the *Alphaproteobacteria* is sensitive to inclusion of several groups of taxa, but it is less affected by the choice of alignment and rate methods. The majority of topologies and comparative results from Approximately Unbiased tests provide support for positioning the *Rickettsiales* and the mitochondrial branch within a clade. This composite clade is a sister group to the abundant marine SAR11 clade (*Pelagibacterales*). Furthermore, we add support for taxonomic assignment of several recently sequenced taxa. Accordingly, we propose three subclasses within the *Alphaproteobacteria*: the *Caulobacteridae*, the *Rickettsidae*, and the *Magnetococcidae*.

## Introduction

The 16S rRNA gene has traditionally been the most heavily used molecular taxonomy marker because of its universal presence, its vertical inheritance, and its constant and slow evolution. However, due to drawbacks such as a limited number of informative characters, new markers have also been sought [1]. Thanks to the quantity of sequenced genomes and higher computational power, many recent studies have used concatenations of large numbers of genes to infer phylogenetic history. However, due to differences in phylogenetic strategies, actual gene histories, and systematic error, gene concatenation studies occasionally disagree with each other and with rRNA gene-based studies (e.g. 2).

An example of such incongruence is the phylogeny of the *Alphaproteobacteria* [3-9], which has received considerable attention because it contains many important taxa, including the ancestor of the mitochondria. The *Alphaproteobacteria* contains members that are pathogens of humans, such as *Rickettsia*, and livestock, such as *Ehrlichia*, as well as agriculturally valuable species, such as *Rhizobium radiobacter* (formerly *Agrobacterium tumefaciens*), and several highly abundant marine groups such as *Roseobacter*, SAR116, and SAR11. The commonly accepted alphaproteobacterial orders are the *Rhizobiales*, the *Rhodobacteriales*, the *Caulobacteriales*, the *Parvularculales*, the *Sphingomonadales*, the *Rhodospirillales*, the *Rickettsiales* [10,11] and the recently validated *Magnetococcales* [12]. Several orders that are represented by a single deep-branching species (namely *Kiloniellales* [13], *Kopriimonadales* [14], *Kordiimonadales* [15], *Sneathiellales* [16] and *Rhodothalassiales*) have been proposed but the relationships of these orders have not been addressed. The most controversial order is the *Rickettsiales*, which is composed of the families *Rickettsiaceae*, *Anaplasmataceae*, *Midichloriaceae* [17] and *Holosporaceae*, with the membership of the SAR11 clade (*Pelagibacterales* [9]) currently under debate (*vide infra*).

Several major differences exist between the various phylogenetic studies of the *Alphaproteobacteria*, especially in taxon and marker selection. In the case of taxa, it is hard to shortlist a subset of taxa that is small enough to be computationally feasible, but large enough to cover the diversity of the group. In the case of marker selection, many studies choose highly conserved housekeeping genes (e.g. 3), but in some cases subsets of these genes with particular properties are chosen (e.g. 6), and the criteria for inclusion vary between studies. These methodological differences sometimes result in the poor choice of markers, such as horizontally transferred genes or those with adaptive properties, genes not universally conserved, or genes inadequately screened against contaminated draft assemblies. Moreover, it has been demonstrated that the most important factor in the correct resolution of a phylogeny is the selection of only genes with a strong phylogenetic signal and without significant incongruence, whereas an increase in the number of genes used does not result in a better resolution [18].

In 2005, Lee et al. inferred the most comprehensive phylogeny of the *Alphaproteobacteria* at that time, by using the 16S rRNA gene and all existing type strains [10]. This study became the basis of current classifications. However, it excluded candidate species, such as members of the *Pelagibacterales* and many members of the *Holosporaceae*, and many more species have been discovered and sequenced since. As a result, further studies have been conducted. In 2007, Williams et al. used a thoroughly selected set of 104 protein-encoding genes and found that *Candidatus* Pelagibacter ubique (*P. ubique*) was basal in the *Rickettsiales* and that mitochondria were sister to the *Rickettsiaceae* and *Anaplasmataceae* [3]. Unfortunately, the study pre-dated the sequencing of *Magnetococcus marinus* (formerly *Magentococcus* *sp.* MC-1 [12,19]) and *Odyssella thessalonicensis* [8], so the clades of these two species were absent from the tree.

In 2011 and 2012, several studies on the phylogenetic placement of SAR11 and mitochondria in the *Alphaproteobacteria* were published near-simultaneously, with conflicting conclusions. Thrash et al. used a variable number of conserved genes and included several newly sequenced members of the “*Pelagibacterales*” in their analysis [4]. They found the same topology for the *Alphaproteobacteria* as that obtained previously [3], but with evidence for *Pelagibacterales* and mitochondria as sister groups. Viklund et al. raised concerns of AT-driven artefacts by finding that the trees inferred from concatenations of proteins with high GC bias favoured the *Pelagibacterales* as a sister clade to the *Rickettsiales*, whereas those from less biased proteins favoured the *Pelagibacterales* as a sister clade to the group of *Rhizobiales*, *Caulobacterales* and *Rhodobacterales* [6]. However, when the species sampling was increased or when maximum likelihood was used instead of Bayesian inference, the resulting trees supported the membership of *Pelagibacterales* within the clade *Rickettsiales*. In that study, mitochondria, *Odyssella thessalonicensis* and *Magnetococcus marinus* were omitted. On the other hand, Georgiades et al. found that the *Pelagibacterales* and the mitochondria formed a sister clade to the *Rickettsiales*, whereas *Odyssella thessalonicensis* was found to be basal to the clade composed of the remaining alphaproteobacterial orders [8]. Rodríguez-Ezpeleta and Embley used a variety of different approaches and found the *Pelagibacterales*–*Rickettsiales* topology with several methods, including RY-recoding in an attempt to account for GC bias, but a different topology was concluded to be correct [7]. This study also omitted *Odyssella thessalonicensis* and *Magnetococcus marinus*, but included mitochondria, which clustered in different locations depending on the methodology.

The key nodes in the alphaproteobacterial tree, such as the branch leading to modern mitochondria, are very ancient (dating to >2 billion years ago; [20]). We chose to revisit the debate over alphaproteobacterial phylogeny using rRNA genes. Being universally conserved and under strong structural and functional constraints, we assert that the rRNA genes are ideal for shedding light on the relationships between the major groups. The 16S rRNA gene remains the gold standard for microbial taxonomy and current ecological studies depend on classifying organisms based on this marker. Furthermore, the large quantity of 23S sequences now available allowed us to use concatenated 16S and 23S sequences, to improve on the only limitation of the 16S (i.e. limited characters) and to provide a better signal in ascertaining problematic inner nodes [21]. While rRNA genes are problematic with regards to long branch attraction artefacts in Eukaryotes [22], we also show that they drastically reduce GC content bias in Bacteria, and thus they allowed us to substantially alleviate this potential source of systematic error. Our results do not support grouping the *Holosporaceae* family with the *Rickettsiales*, and do support the hypothesis that the *Pelagibacterales* is a sister group to the *Rickettsiales*, in a new subclass (*Rickettsidae subcl. nov.*) that also includes the mitochondria.

## Results

### GC content of rRNA genes is comparatively unbiased

**Figure 1:**
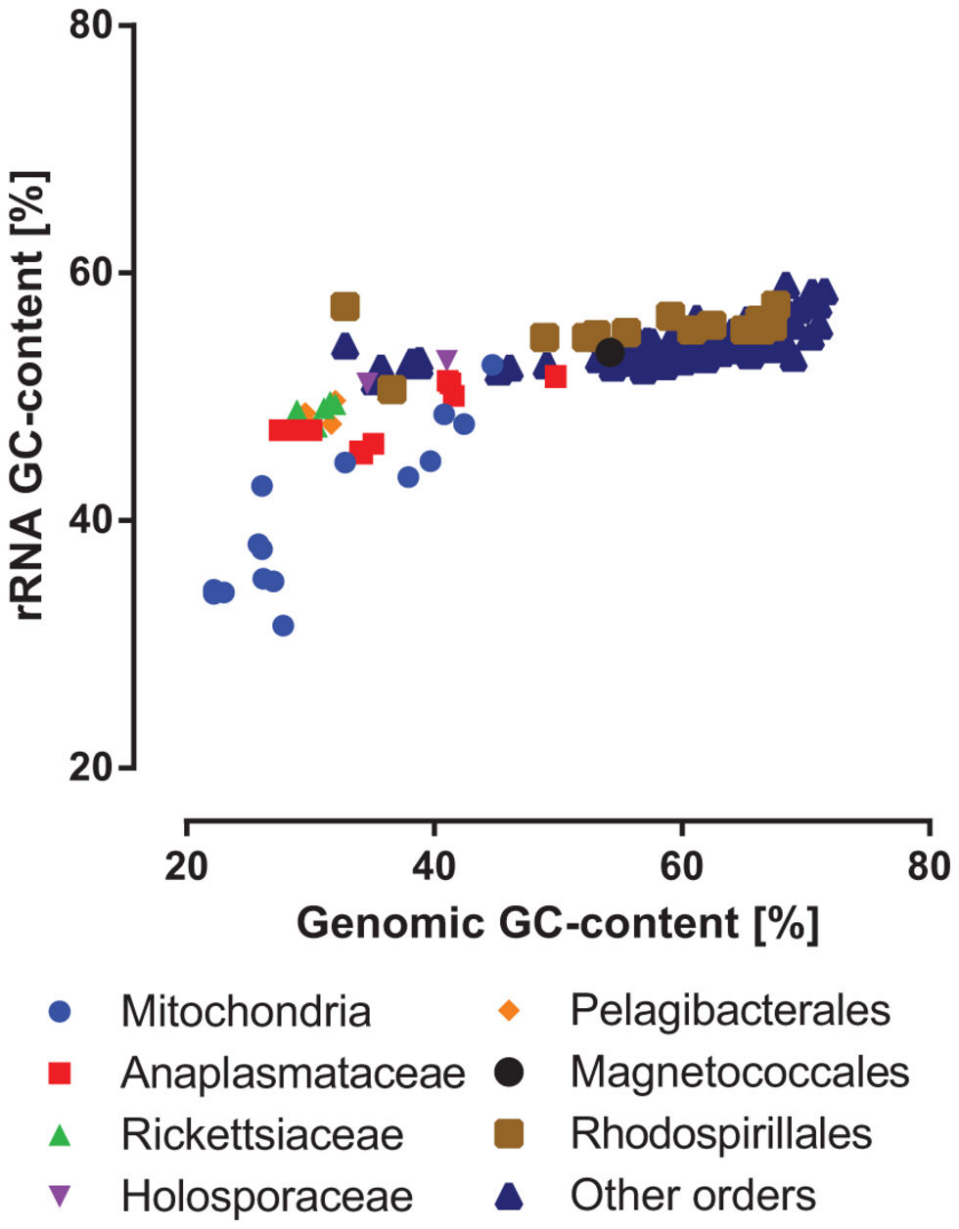
Relationship of rRNA gene vs. genomic GC content for *Alphaproteobacteria* and mitochondria. The rRNA gene GC content was calculated from the entire sequence using a perl script, while the genomic GC content was taken from the IMG database. The “other orders” group includes the *Caulobacterales*, *Sphingomonadales*, *Rhizobiales*, *Rhodobacterales* and *Parvularculales*.

Several alphaproteobacterial groups have extremely AT-rich genomes. This has led to speculation that the topology in which the *Pelagibacterales* cluster with the *Rickettsiales* may be an artefact, due to a spurious attraction of AT-rich taxa [7]. We hypothesized that there may be less freedom for GC content to change in rRNA genes, compared with protein-coding genes, because rRNA is under structural constraints. To test this hypothesis, we analyzed the GC content of genomes, compared to rRNA genes, for single members of each species that was available in the IMG v350 database. We found that the GC content of rRNA genes is considerably less varied than that of the corresponding genomes (Figure 1). Linear regression analyses for the alphaproteobacterial groups gave a slope of 0.15 for the 16S sequences (*R* ^2^ = 0.63) and a slope of 0.20 for the 23S sequences (*R* ^2^ = 0.72), demonstrating that there is 5- to 6-fold less variation in rRNA gene GC content than in genomic GC content. There was even less bias after the highly variable sites were removed from our alignments (Figure S1 and Figure S2). These results validated our decision to use rRNA gene sequences for building trees that do not suffer from artefactual attraction of AT-rich taxa. However, the trend did not hold for mitochondrial LSU and SSU rRNA gene sequences. Linear regression yielded slopes of 0.69 (*R* ^2^ = 0.82) and 0.71 (*R* ^2^ = 0.79) for the 16S and 23S sequences in Figure 1, respectively. Therefore, the mitochondrial sequences were treated with caution and topologies were inferred both with and without them.

### Tree building strategy

We examined the phylogeny of the *Alphaproteobacteria* using single members of each species for which both the 16S and 23S rRNA genes were available, together with seven outgroup taxa from adjacent *Proteobacteria* classes (‘complete’ trees). All of the taxa used in our analyses are listed in Table S1. Using this complete dataset, trees were made from 16S and 23S rRNA genes alone to generate initial topologies, and subsequently with concatenations of the 16S and 23S alignments. We also tested the effect of reducing taxon selection across the tree by subsampling to an idealized membership based on the monophyletic groupings observed in the complete trees (‘trimmed’ dataset). All sets of taxa were aligned with both Muscle [23] and Arb-Sina [24] to test for incongruence between common rRNA gene alignment methods, and computed with maximum-likelihood (RAxML; [25]) using both GTRΓ and GTRCAT rate models. Each of these datasets was evaluated for compositional bias using RY recoding [26], as well as a novel method that we name RYMK recoding, and all were evaluated with and without mitochondrial sequences. Additionally, to understand the effect of sampling bias from the lack of any one group, we employed a clade-specific jackknifing approach in which we removed each of the monophyletic groups from the dataset and re-calculated the tree. This was done for both alignment methods, both rate categories, and plus or minus mitochondrial sequences. Finally, because many important under-examined alphaproteobacterial taxa have neither genome sequences nor 23S rRNA gene sequences available, we examined their taxonomic affiliation by aligning their 16S sequences with the concatenated 16S-23S bacteria dataset (‘combo’ dataset). This dataset was also tested under both alignment and rate model algorithms. These variations generated a total of 140 trees (summarized in Table 1), all of which are available in the Supporting Information. 

**Table 1:** Summary statistics for the datasets used in this study.

Dataset name**^*†*^**	# Taxa	# Chars (Muscle/ Arb)	Coding	Average support value ± SD (%)**^*‡*^**	Average Robinson-Foulds metric within the set ± SD**^*‡*^**
16S^£^ + mito	190	1371/1078	regular	67 ± 1.7	57.3 ± 7.5
23S^£^ + mito	190	2621/2159	regular	80.5 ± 0.6	30.0 ± 7.0
complete* + mito	190	3992/3237	regular	82.7 ± 0.5	26.3 ± 4.8
complete* + mito	190	7984/6474	RYMK	85.7 ± 0.7	25.5 ± 4.1
complete* + mito	190	3992/3237	RY	72.5 ± 0.6	48 ± 8.9
complete* + mito	190	3992/3237	MK	79.2 ± 0.8	31.8 ± 4.7
16S^£^ − mito	166	1412/1204	regular	69 ± 0	28.5 ± 5.0
23S^£^ − mito	166	2661/2246	regular	83 ± 0	12.8 ± 2.1
complete* − mito	166	4073/3473	regular	83.2 ± 0.5	15.6 ± 2.3
complete* − mito	166	8146/6946	RYMK	83 ± 0.4	15.8 ± 2.3
complete* − mito	166	4073/3473	RY	73.7 ± 0.6	32 ± 4.6
complete* − mito	166	4073/3473	MK	80.0 ± 0.7	22.3 ± 3.4
complete mtDel	166	3992/3237	regular	81.2 ± 0.7	21.6 ± 4.5
trimmed* + mito	111	3990/3335	regular	77.2 ± 0.5	22.8 ± 4.9
trimmed* + mito	111	7980/6670	RYMK	79 ± 0.9	27.8 ± 4.8
trimmed* + mito	111	3990/3335	RY	66.7 ± 2.5	26.1 ± 5.4
trimmed* + mito	111	3990/3335	MK	71.5 ± 0.9	17.2 ± 3.9
trimmed* − mito	87	4121/3542	regular	76.7 ± 0.5	18.3 ± 2.9
trimmed* − mito	87	8242/7084	RYMK	74.2 ± 0.2	20.8 ± 2.6
trimmed* − mito	87	4121/3542	RY	67 ± 2.3	19.5 ± 4
trimmed* − mito	87	4121/3542	MK	72.7 ± 0.7	12.3 ± 2.4
exorickettsialess**^*#*^** + mito	171	4000/3166	regular	82.5 ± 1.2	27.5 ± 6.9
exorickettsialess**^*#*^** − mito	147	4097/3488	regular	83.5 ± 0.3	14.8 ± 3.3
hololess**^*#*^** + mito	188	4009/3281	regular	83.7 ± 0.5	24.1 ± 5.3
hololess**^*#*^** − mito	164	4069/3463	regular	83.5 ± 0.3	14.1 ± 1.2
magnetoless**^*#*^** + mito	189	4015/3275	regular	83.0 ± 0.6	27.2 ± 6.6
magnetoless**^*#*^** − mito	165	4080/3448	regular	83.5 ± 0.3	19.5 ± 3.9
pelagiless**^*#*^** + mito	182	3452/3999	regular	81.0 ± 0.9	33.0 ± 6.7
pelagiless**^*#*^** – mito	158	3275/4048	regular	82.7 ± 0.7	20.5 ± 3.8
rhodoless**^*#*^** + mito	174	3984/3281	regular	82.5 ± 0.5	24.8 ± 5.3
rhodoless**^*#*^** − mito	150	4114/3466	regular	83.2 ± 0.5	18.8 ± 2.9
rickettsialess**^*#*^** + mito	149	3282/3980	regular	82.0 ± 0.9	25.6 ± 5.1
rickettsialess**^*#*^** − mito	149	3472/4096	regular	82.2 ± 0.5	17.5 ± 2.6
combo^$^ + mito	277	3992/3237	regular	49.3 ± 12.6	74.2 ± 0.5
combo^$^ − mito	253	4073/3473	regular	37.8 ± 8.1	76.0 ± 0.4

***^†^*** Four trees were constructed for each dataset, using all combinations of alignment method (Muscle and Arb-Sina) and rate model (GTRΓ and GTRCAT). ***^‡^*** Values are not normalised by number of taxa, so only the metrics for datasets containing the same taxa can be compared with each other. **^*£*^** Trees inferred from only 16S or 23S sequences; *primary trees; **^*#*^**trees with single clades removed; and **^*$*^**concatenated bacterial trees with additional 16S rRNA gene sequences.

### The 16S and 23S rRNA gene trees

We began by creating phylogenies of the 16S and 23S rRNA genes separately (the ‘16S’ and ‘23S’ datasets in Table 1). These topologies provided a baseline to which we could compare our concatenated rRNA gene trees. The phylogenies were completed with taxa from the complete dataset, both alignment methods, both rate models, and both with and without mitochondrial taxa, for a total of eight trees each of the 16S and 23S rRNA genes (Figure S11). Whereas the 16S trees resolved the *Holosporaceae* within the clade including the *Rhodospirillales* and other orders (with low bootstrap support), the 23S trees did not consistently resolve the monophyly of *Holosporaceae*, nor its location (bootstrap supports are summarized in Figure S3 and Figure S4). Conversely, the 16S trees did not consistently resolve the *Pelagibacterales* in a specific location. Neither the 16S nor 23S phylogenies could consistently resolve the *Caulobacterales*, *Rhizobiales* and *Rhodobacterales*. A 16S tree that was used previously to classify the *Alphaproteobacteria* [10] had many similar results, but also some differences. For example, the *Holosporaceae* did not resolve monophyletically and was basal to the *Rickettsiales* (the *Pelagibacterales* were absent), whereas here the *Holosporaceae* is close to the *Rhodospirillales*.

### The complete concatenated trees

**Figure 2:**
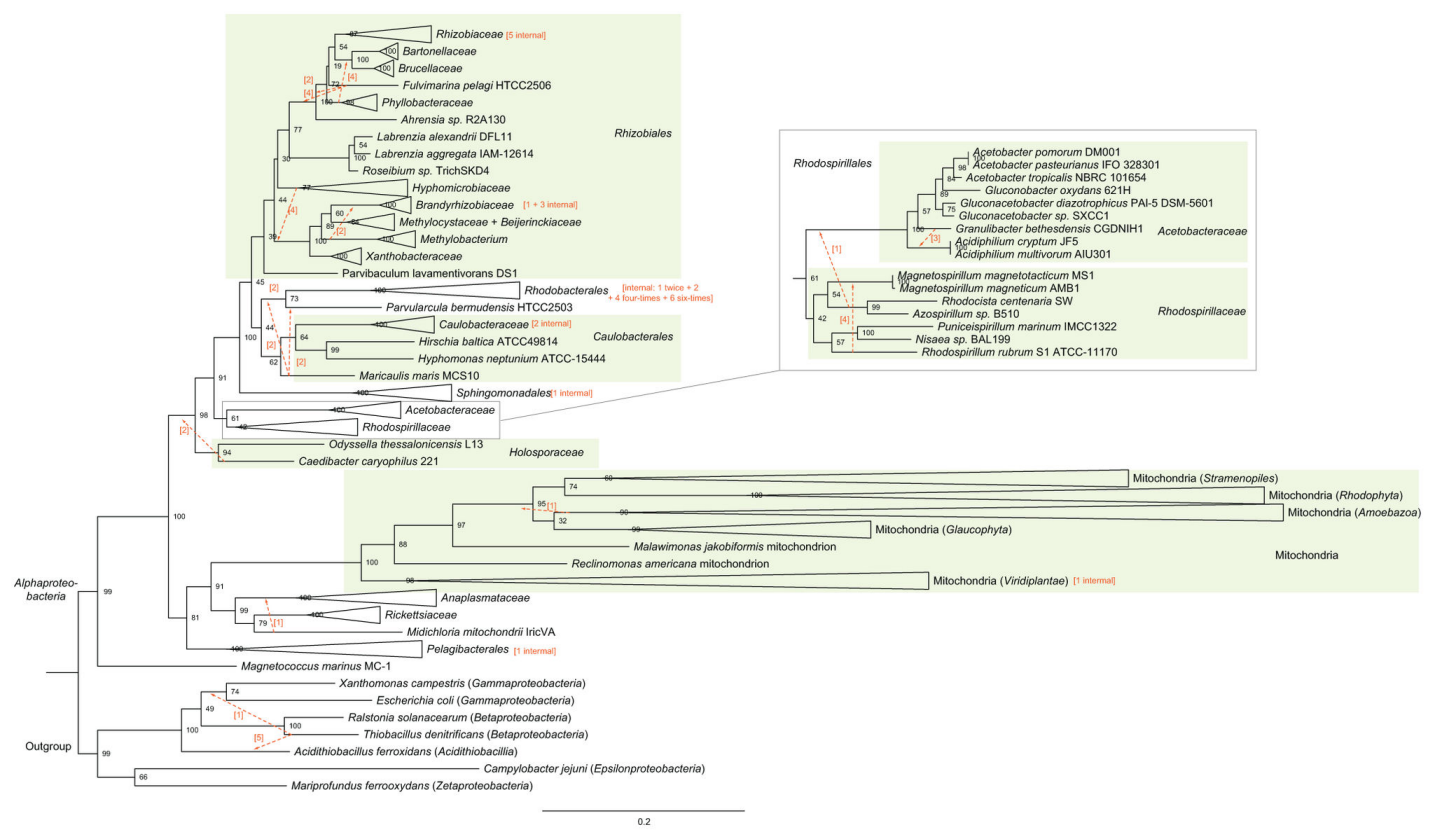
Tree inferred with the Arb-sina aligned complete dataset under a GTRΓ model. Bootstrap values (n = 1000) are indicated at the nodes. Red arrows indicate how a taxon or clade differs in the other regularly coded trees, with values in square brackets indicating in how many trees this is seen. If there are one or more differences within a family, this is indicated after the name of the family. The leaves of the phylogram are collapsed into taxonomic families and into the host phyla for mitochondria. The internal topology of the *Rhodospirillales* order is not the same in all primary trees, therefore it has been expanded to show all leaves (inset).

**Figure 3:**
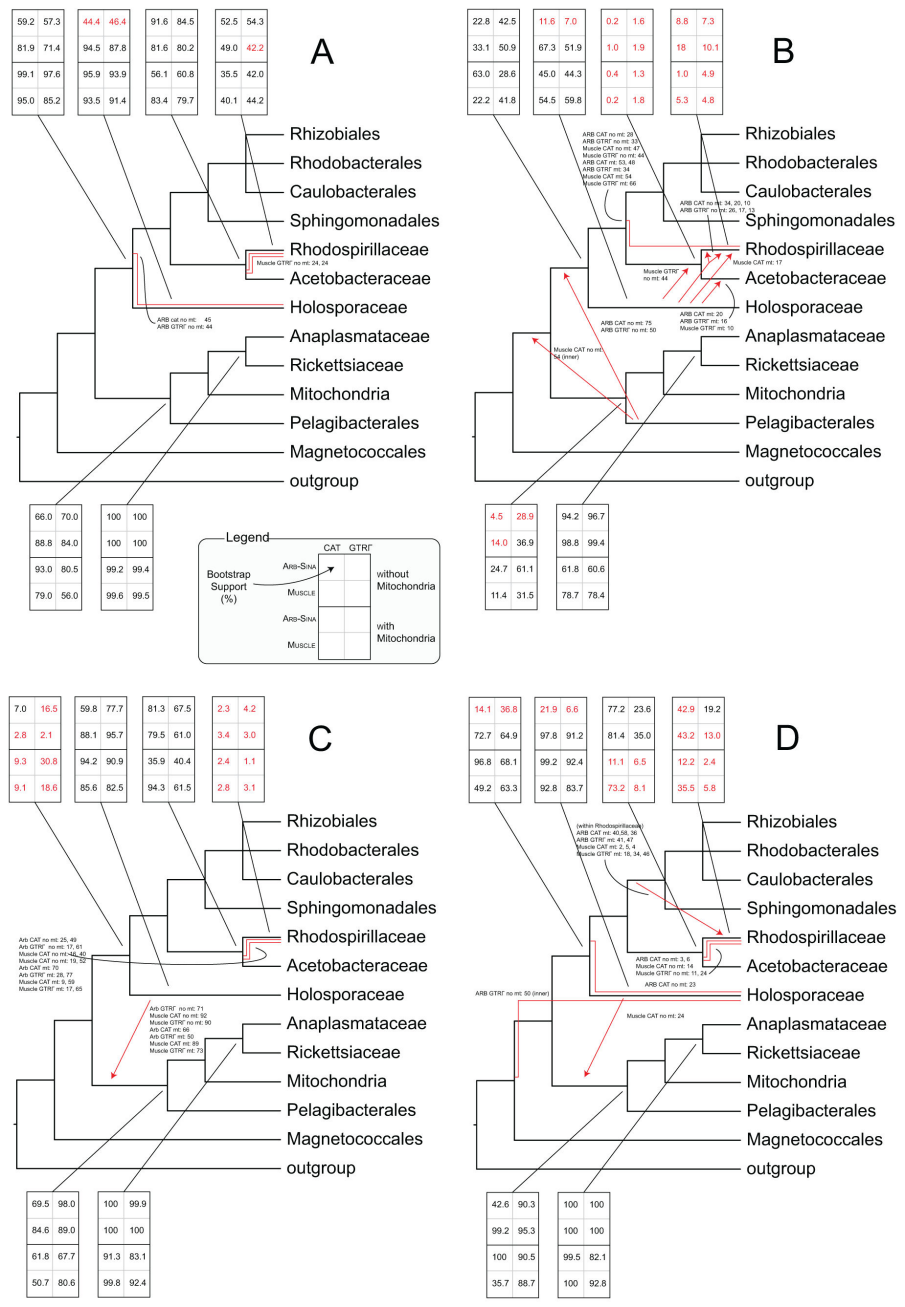
Summary of bootstrap supports. The values in the boxes are the bootstrap support percentages for the indicated bipartitions, from each of the eight inferences for each set, as described in the legend (i.e. with or without mitochondria, aligned with Arb-Sina or Muscle, GTRΓ or GTRCAT rate model). In the cases where the final tree did not agree with the proposed topology on the location of a clade, the bootstrap support for the proposed bipartition is represented in red, despite being absent in the final tree. **A**. Regular-coded complete datasets. **B**. RY-recoded complete datasets. **C**. MK-recoded complete datasets. **D**. RYMK-recoded complete datasets.

To increase resolution we concatenated the 16S and 23S rRNA genes, and explored topological stability between different alignment (Arb-Sina, Muscle) and rate (GRTΓ, GTRCAT) methods, as well as the variable inclusion of mitochondrial sequences. The complete trees included a representative of each defined *Alphaproteobacteria* species present in IMG v350 and NCBI Genbank, for which 16S and 23S rRNA gene sequences were available (Table S1). Specifically, only a single strain was picked for each species, whereas in the cases of unclassified strain, all were chosen. In this set of eight trees (Figure S12), the *Alphaproteobacteria* is divided into three primary clades (representative final tree in Figure 2; bootstrap summary in Figure 3A). The earliest diverging clade is the *Magnetococcales*, represented by *Magnetococcus marinus*. One of the subsequent clades has the *Pelagibacterales* subtending the *Anaplasmataceae*, *Midichloria mitochondrii*, the *Rickettsiaceae* and the mitochondria (if present). The other clade has the *Holosporaceae* at the base, the *Rhodospirillales* as the next clade branching out, followed by the *Sphingomonadales*, then the remaining orders. The *Holosporaceae* is represented by *Odyssella thessalonicensis* and *Caedibacter caryophilus*, and is monophyletic in six out of the eight trees, but in two it resolves paraphyletically at the same location. The *Holosporaceae* family is currently classified as a member of the *Rickettsiales* [10,11], but this topology (with monophyletic *Holosporaceae*) was not seen in our final tree and it is only supported in a very small fraction of the bootstrap trees (2.0%, 2.0%, 0.3%, 0.4%, 0.4%, 6.8%, 4%, 1.3%). The support of the monophyly of the *Rhodobacterales*, *Caulobacterales* and *Rhizobiales* increased greatly when some species were reclassified, namely when *Maricaulis maris* was removed from the *Hyphomonadaceae* (*Caulobacterales*) and when the clade formed by *Labrenzia* and *Roseibium* species was moved from the *Rhodobacteraceae* (*Rhodobacterales*) to the *Rhizobiales*. These reclassifications are listed in Table S1, with associated changes in support shown in Figure S5. The support for the monophyly of the *Rhodospirillales* was not high, varying from 56% to 92% in the complete trees, and the monophyly of the *Rhodospirillaceae* had even less support (42–52%).

### The complete RY-, MK- and RYMK-recoded trees

**Figure 4:**
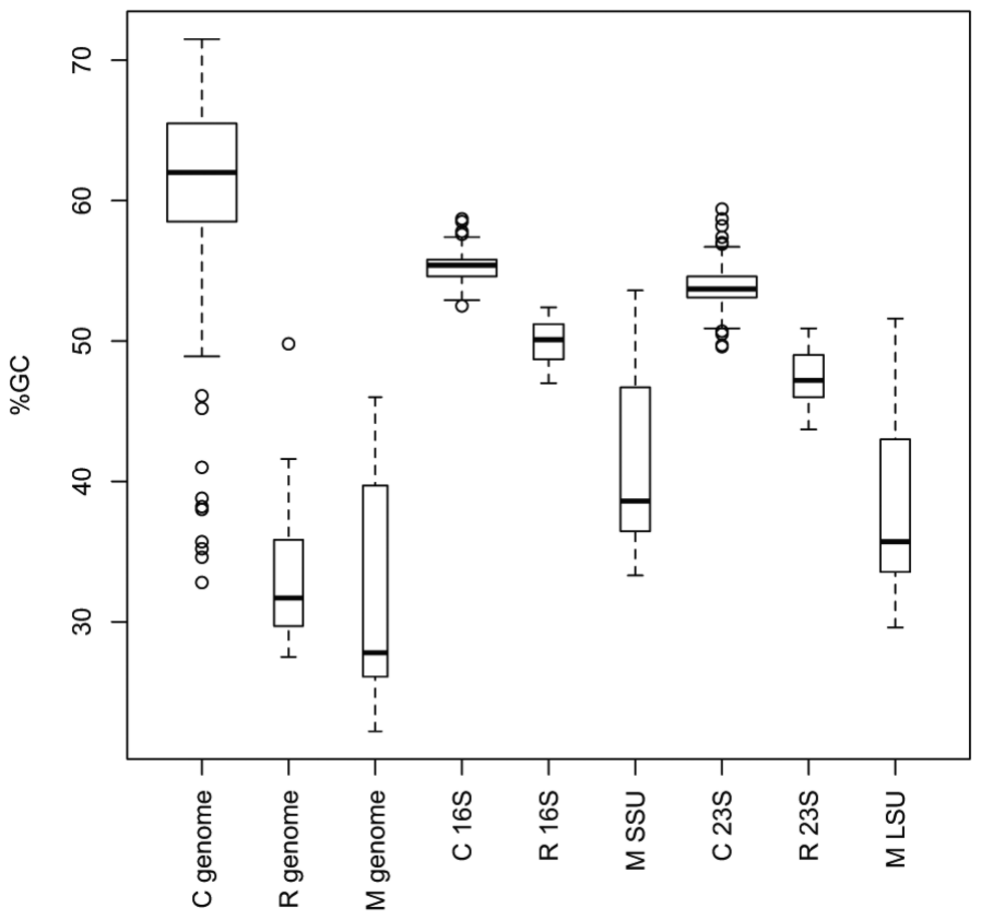
Box plot of the distributions of GC content for the genomes, SSU and LSU rRNA genes for members of the primary clade headed by *Caulobacter* (C), the clade headed by *Rickettsia* (R) and the mitochondria (M). Boxes indicate the interquartile range, with adjacent values as whiskers, outlying values as circles; median indicated by the horizontal black line. Box width is proportional to the total number of samples.

Accepting the topology formed with the regular-coding complete datasets, we compared the GC content of the crown group to that of the *Rickettsiales*–*Pelagibacterales* and to that of the mitochondria, separately. As expected based on the data in Figure 1, GC content of the SSU and LSU rRNA genes exhibited much smaller variation than that of the genomes (Figure 4). Nevertheless, there were small, statistically significant differences when the GC contents of the crown group SSU and LSU sequences were compared to the *Rickettsiales*–*Pelagibacterales* sequences (Wilcoxon rank-sum *p*-values = 2.34 x 10^-15^ and 4.70 x 10^-15^, for SSU and LSU sequences respectively). This analysis indicated that using the rRNA genes mitigated GC bias substantially, but did not eliminate it completely.

In light of this analysis, together with previous concerns surrounding the AT richness in the *Alphaproteobacteria* [5-7], we tested RY-recoding of the dataset. This approach [26,27] masks GC content bias by recoding adenosine (A) and guanosine (G) as purines (R), and cytidine (C) and thymidine (T) as pyrimidines (Y). For consistency with our other analyses, we analyzed the recoded dataset with the GTR model. This approach has also been taken by others [7]. Support for most of the orders and families dropped in the RY-recoded datasets, compared to those with regular coding (bootstrap summary in Figure 3B; summary statistics in Table 1). There was a large decrease in support for the monophyly of the *Rhodospirillales*, *Rhizobiales* and *Caulobacterales*. However, support for the monophyly of some orders, such as the *Pelagibacterales* and *Sphingomonadales*, did not change greatly. The trees of the RY-recoded datasets that included mitochondria resolved the *Pelagibacterales* as a sister clade to the mitochondria–Rickettsiaceae–*Midichloria mitochondrii*–*Acetobacteraceae* clade (without the *Holosporaceae*). Conversely, three of the four trees without the mitochondria placed the *Pelagibacterales* as paraphyletic with the *Rickettsiales* (Figure S13). 

As a consequence of the poor resolution obtained with the RY-recoded dataset, we utilized a novel variant: each RY-recoded dataset was concatenated to an MK-recoded dataset (“RYMK”). In the MK recoding, adenosine (A) and cytosine (C) were recoded as amino bases (M), and guanosine (G) and thymidine (T) as keto bases (K). Thus, the GC bias information is lost in the same way as RY-recoding, however the concatenated RYMK dataset retains the overall number of characters. This approach was designed as a tool to improve phylogenetic analyses of datasets with GC bias, rather than reflecting any biological process(es). We note that RY-recoding has been used similarly [27]. The site-independent nature of the GTR model ensured that it remained appropriate for analyzing the concatenated RYMK dataset.

Used on its own, MK-recoding suffered from similar issues to RY-recoding (Figure 3C and Figure S13). In particular, the MK-recoded datasets do not resolve the *Rhodospirillales* correctly; consequently the basal *Holosporaceae* is destabilized to the point that it clusters with the *Rickettsiales*. However, average support values and Robinson-Foulds metrics (Table 1) showed that the MK-recoded trees were more consistent than the RY-recoded trees, with respect to alignment method and rate model. On the other hand, in the RYMK-recoded datasets the grouping of the *Pelagibacterales* with the *Rickettsiaceae*, *Midichloria mitochondrii*, *Anaplasmataceae* and mitochondria, and the grouping of *Holosporaceae* with the remaining orders, are both more supported than in either the RY-recoded datasets or the MK-recoded datasets (bootstrap summary in Figure 3D; trees in Figure S14). These groupings also have more consistent topologies (7 of 8 trees; compare Figure 3B-C with Figure 3D).

### The trimmed concatenated trees

We have asserted that it is advantageous to focus on LSU and SSU sequences because it maximizes the number of taxa that can be included. In contrast, genome concatenation studies rely on a smaller number of (fully sequenced) taxa. In order to test the impact of reduced taxon selection on overall topology, we constructed trimmed datasets with fewer bacterial taxa. By reducing the number of taxa on short branches, in clades that had more than 95% bootstrap support in the trees from the complete datasets, we reduced the number of *Alphaproteobacteria* sequences from 166 to 86, while leaving the number of mitochondrial sequences at 24. The 32 resulting trees (‘trimmed’ dataset in Table 1) are shown in Figure S15 (regular coding), Figure S16 (RY- and MK-recoded) and Figure S17 (RYMK-recoded). Despite the high inclusion threshold, the trees differed substantially from the complete phylogenies. Bootstrap summaries are provided in Figure S6 (regular coding), Figure S7 (RY- and MK-recoding) and Figure S8 (RYMK-recoding). None of the trimmed trees with regular coding resolved the *Rhodospirillaceae* monophyletically, the location of the *Holosporaceae* within the clade containing *Caulobacterales* varied substantially, and the Arb-Sina-aligned trees without mitochondria placed the *Pelagibacterales* paraphyletic with the *Rickettsiales*, albeit with poor bootstrap support (< 40%). The trees inferred from the RY-, MK- and RYMK-recoded datasets had even more diverse topologies (bootstrap summaries in Figures S6–S8). The *Rhodospirillales* were either paraphyletic with respect to the clade composed of *Sphingomonadales*, *Rhizobiales*, *Rhodobacterales* and *Caulobacterales*, or paraphyletic or polyphyletic in respect to the *Holosporales*. The *Pelagibacterales* was found basal to the remaining *Alphaproteobacteria* (except for *Magnetococcus marinus*) or basal to the clade containing the *Caulobacterales* in all but three trees. Despite the differences, the mitochondria resolved consistently in the trimmed and complete trees.

### Trees with single clades removed

In an effort to identify the particular taxa or groups that may be critical to the observed instability of the complete phylogeny, we tested the effects of clade-specific jackknifing on topology. Several orders or families were removed, and trees were constructed with each of these single clades removed. The six orders and families removed were the *Rhodospirillales* (‘rhodoless’ dataset in Table 1), *Magnetococcus marinus* (‘magnetoless’ dataset in Table 1), the *Rickettsiales* including the *Holosporaceae* (‘exorickettsialess’ dataset), the *Rickettsiales* excluding the *Holosporaceae* (‘rickettsialess’ dataset), the *Holosporaceae* (‘hololess’ dataset), and the *Pelagibacterales* (‘pelagiless’ dataset). For each of the six datasets, eight trees were constructed (Arb-Sina and Muscle alignments; GRTΓ and GTRCAT rate models; plus and minus mitochondrial sequences). The 48 trees are shown in Figure S19. Removal of most orders had little effect on the tree. An exception was the problematic resolution of the *Holosporaceae* (e.g. paraphyletic in six trees out of 48, basal to the *Pelagibacterales* in two trees, basal to the *Rhodospirillales* in three trees, or nested in the *Rhodospirillales* in one). Removing the *Rhodospirillales* had the largest effects, destabilising the *Holosporaceae* and the *Pelagibacteraceae* (*Holosporaceae* basal to *Pelagibacterales* and *Rickettsiales* in two trees; *Pelagibacterales* basal to *Holosporaceae* and the clade of various orders in four trees).

### Mitochondrial placement

Trees were made with and without mitochondrial sequences (Table 1). Despite the longer branches of the mitochondrial clade, the trees inferred from the various datasets with mitochondria better resolved the overall topology shown in Figure 2, as opposed to the trees without mitochondria. For example, the primary clade composed of *Rickettsiaceae*, *Anaplasmataceae*, *Midichloria mitochondrii* and *Pelagibacterales* – in which mitochondrial sequences also cluster – and the clade with a basal *Holosporaceae* and various orders are supported much more strongly in the datasets with mitochondria, especially the Arb-Sina-aligned datasets. This is true not only for the regularly coded full datasets (Figure 3A), but also for the RY-, MK- and RYMK-recoded ones (Figure 3B-D), and the jackknifed datasets. However, this is not the case for the trimmed datasets, where the changes in support with and without mitochondria vary between alignment method and inference model. 

In contrast to the increase in support for the primary clade containing the mitochondria in the full trees, the monophyly of the *Rhodospirillales* and the *Rhodospirillaceae* loses support when the mitochondria are added, especially in the RYMK-recoded dataset. The internal topology of the *Rhizobiales* also changes. In the primary trees without mitochondria, the *Hyphomicrobiaceae* is basal to a clade formed by the *Beijerinckiaceae*, *Methylocystaceae*, *Methylobacteriaceae* and *Xanthobacteraceae*, while in those trees with mitochondria the family is basal to the sister subclade of the *Rhizobiales*, composed of the remaining families bar *Parvibaculum lavamentivorans* (basal to the two subclades) and with the addition of *Labrenzia* spp. and *Roseibium* *sp.* (Figure 2). 

The datasets with and without mitochondria were separately aligned and trimmed. One hypothesis for the variations in topology between trees, with and without mitochondria, was that the trees with mitochondria were influenced by having fewer characters as a result of the Gblocks editing criteria. We tested this hypothesis by pruning mitochondrial sequences post-alignment, from all datasets that included mitochondria (‘complete mtDel’ dataset in Table 1). These new alignments were processed with both rate models, and the resulting trees (Figure S18) were compared to those of the alignments that were initially done without mitochondria. In order to compare the trees with mitochondria and the trees without, the mitochondrial leaves were pruned from the former group. Overall, the locations of the *Pelagibacterales*, the *Holosporaceae* and other groups of interest are the same in the ‘mtDel’ trees as seen in the other trees. However, the bootstrap support of all groups is lower (Figure S9), except for the support for the monophyly of the *Holosporales* in the trees from the Arb-Sina aligned dataset. Overall, the presence of the mitochondrial sequences adds support to the trees, but at the same time results in a smaller number of characters (due to Gblocks editing).

### 16S-23S dataset with several 16S-only sequences

There are a vast number of species represented in the databases solely by their 16S rRNA gene sequences. Within the *Alphaproteobacteria*, there are 975 validly-described type strains [28], against approximately 150 genotyped species. Several of the species for which we have only 16S rRNA gene sequences branch deeply within the tree, to the point that some are reported to be part of their own orders. As a consequence, a group of deep-branching taxa with only 16S sequences was aligned to the other 16S sequences, and the 23S rRNA gene positions left as missing data in the concatenated alignments. The result was the ‘combo’ dataset (Table 1).

In the resulting trees (summary in Figure S10, all trees in Figure S20), the deep-branching species that were added reduce the support for several nodes, indicating that this approach is not ideal. However, it does offer a snapshot into the diversity of the *Alphaproteobacteria* that is not covered by the genome databases. Several species classified as *Rhodospirillales* do not cluster with the *Rhodospirillales*, but instead cluster with the orders currently represented by a single genus, some of which appear to be synonyms. More specifically, in the trees, the order *Kiloniellales* contains *Kiloniella laminariae*, *Kopriimonas byunsanensis*, *Rhodovibrio salinarum* and *Pelagibius litoralis*. The order *Kordiimonadales* includes *Kordiimonas gwangyangensis*, *Rhodothalassium salexigens* and a diverse group of iodine-oxidising bacteria. The order *Sneathiales* contains only *Sneathiella chinensis*. However, in the *Rhodospirillales*, several species with only 16S sequences are found in basal positions. The *Acetobacteraceae* can be expanded to include basally *Elioraea tepidiphila*, *Alysiosphaera europeae* and *Geminicoccus roseus*. Furthermore, the genus *Tistrella* may be basal to all of the *Rhodospirillales*. Full genome sequences of these species may therefore increase the resolving power of future studies.

### Approximately Unbiased tests

Trees from the 16S, 23S, complete with regular encoding, complete RY-recoded and complete RYMK-recoded datasets were assessed against the various alignments with the Approximately Unbiased (AU) test [29]. A *p*-value of 0.05 was used as the cut-off, such that trees with *p*-values below this number could be rejected based on an alignment. Generally, trees from a given dataset were unable to be rejected by their respective alignments from either Arb-Sina or Muscle. On the other hand, they could be rejected by most of the alignments that were based on other datasets (Table S2). For the complete datasets without mitochondria, none of the four regularly coded primary trees (GTRCAT and GTRΓ with Arb-Sina and Muscle alignments) could be rejected based on the Arb-Sina or Muscle alignments. For the complete datasets with mitochondria, of the four regularly-coded trees, two could be rejected by one of the alignments. The four trees with mitochondria agree on the topology of the orders and families and differ by a Robinson-Foulds distance of less than 22 different bipartitions (Table S2) while the four trees without mitochondria disagree in some instances regarding families, but differ by less than 18 bipartitions. Whereas all of the trees from the 16S, 23S and RY-recoded datasets could be rejected based on the two regularly-coded complete alignments, the RYMK-recoded trees could not be rejected based on 11 of the 16 comparisons with the regularly coded alignments.

## Discussion

In this study, we updated the alphaproteobacterial rRNA gene tree and explored its topological stability by systematically varying a series of parameters: alignment method; rate model; character encoding; and taxon sampling. We focused on rRNA genes, rather than protein-encoding genes, in order to recover phylogenetic signals that may have been obscured due to the genomic AT-richness and ancient divergence events that are hallmarks of the *Alphaproteobacteria*. We focused on concatenated 16S-23S rRNA gene sequences to give better support than 16S alone, while also allowing the inclusion of many more taxa than what is currently feasible with available genomes for concatenation studies. One downside of our approach is the limited number of full-length 23S sequences, relative to 16S. However on balance, and given the controversy that surrounds the phylogeny of the *Alphaproteobacteria* [4-8], we hypothesized that concatenated 16S-23S rRNA gene sequences would offer the greatest insights into accurate placement of the major groups.

### GC bias

The debate over alphaproteobacterial phylogeny focuses mainly on the location of the *Pelagibacterales* (SAR11 clade), and particularly whether it is a sister clade to the *Rickettsiales* or whether they are artefactually attracted due to shared low GC content [5-7]. Despite concerns about the effects of such compositional biases on tree topologies [26], little is known about the effect of genomic GC composition on 16S and 23S sequences. Here, we found that for the *Alphaproteobacteria* sequences that we analyzed, the GC content of rRNA genes was 5- to 6-fold less variable than the genomic GC content (Figure 1). This validated the choice of rRNA genes for minimizing artefacts that were due to shared AT richness, although further analysis showed that the GC bias was not completely eliminated in our rRNA gene datasets (Figure 4).

Consistent with the result in Figure 1, several of our further tests argued against a GC content-driven artefact affecting the position of the *Pelagibacterales*. Using the jackknifing approach, we tested the hypothesis that the *Pelagibacterales* and the *Rickettsiales* attract each other. Under this hypothesis, the AT-rich *Rickettsiales* should mask the true phylogenetic signal by attracting the AT-rich *Pelagibacterales*, and therefore the removal of the *Rickettsiales* from the dataset should reveal the location of the *Pelagibacterales* independently of this attraction. However, the *Pelagibacterales* were placed in a similar position, both in the complete dataset (Figure 2) and when *Rickettsiales* were missing (Figure S19). Additional evidence against compositional bias-driven attraction comes from the recoded datasets. The RY-recoded trees were less supported on many nodes, which may be due to the presence of two instead of four character states. We introduced RYMK-recoding to help overcome the limitation caused by the reduced number of character states and we found that the resulting trees concurred with the regular dataset, regarding the clustering of the *Pelagibacterales* with the mitochondria, *Rickettsiaceae* and *Anaplasmataceae*. Approximate Unbiased tests confirmed that RYMK-encoding (which eliminates GC bias) was in greater agreement with regular encoding than either RY- or MK-encoding. Therefore we recommend RYMK-recoding as a superior alternative to RY-recoding for assessing artefactual attractions that may arise due to GC content biases. 

Overall, in datasets of concatenated 16S and 23S sequences, we found no evidence that GC content may be contributing to a topology where the *Pelagibacterales* cluster with the *Rickettsiales*.

### Effects of alignment method, rate model and taxon sampling

In general, the Muscle and Arb-Sina alignments generated trees that agreed well with each other when used in combination with Gblocks. Also, the trees generated with the two different rate models (GTRΓ and GTRCAT) did not differ substantially and had similar AU scores. Therefore it can be concluded that in the case of rRNA gene-based trees computed with RAxML, neither the choice of alignment program, nor the rate model, significantly affect the final topology.

In contrast, taxon selection greatly affected the final tree. The number of taxa present was reduced by trimming the dataset of leaves that were assumed to be contributing little to the overall tree topology. Contrary to expectations, the trees from the trimmed datasets had lower support values and were much less consistent with each other compared to the complete trees (compare Figure 3 with Figures S9-S11). Extra species, represented by 16S sequences only, were also added to yield the ‘combo’ dataset. Trees constructed with this enlarged dataset also showed reduced support (Figure S20), consistent with the reduction in characters that came from not having 23S sequences for these species. Our strategy of aligning concatenated 16S-23S sequences, using at least one representative of each species, yielded trees with the highest possible support (Figure 2).

### Taxonomic observations

The divergence of the clade with *Rickettsia* and the clade with *Caulobacter* has been estimated to have occurred 1,650–2,390 million years ago [30]. Therefore, it is clear that several taxa may find themselves on long branches, which may spuriously attract [31]. To minimise the quantity of long branches, one representative of each alphaproteobacterial species, represented by both 16S and 23S sequences, was chosen in this study. Moreover, by analyzing only the 16S and 23S markers it was also straightforward to screen manually for misannotation errors and to correct or exclude problematic taxa. For example, the contig NZ_AAAP01003712 for *Magnetospirillum magnetotacticum* was found to be contaminated with sequence from *Methylobacterium populi*, from position 3,624 to 8,812 (data not shown).

In contrast to previous analyses, this study covered all of the known diversity within *Pelagibacterales* (i.e. subgroups I, III, IV and V, as classified by Grote et al. [9]). The monophyly of the group was confirmed with more than 95% support in all trees inferred. Therefore, our data provided no evidence that *Pelagibacterales* may be polyphyletic, in agreement with one recent study [9], but not another [7]. The *Pelagibacterales* fall basal to a clade composed of mitochondria and a *Rickettsiales* subclade without the *Holosporaceae*, in all of the full trees. This grouping has a moderate bootstrap support, but this is most likely due to the instability of the *Holosporales* (*vide infra*) and not due to an AT-attractional bias (*vide supra*).

In our four complete, regularly coded trees the mitochondria are a sister group to a clade formed by *Anaplasmataceae* and *Rickettsiaceae*, with high support. This result is consistent with several studies that used concatenated protein phylogenies [3,4,8], although slightly different to Georgiades et al. [8] and Thrash et al. [4], which found support for the *Pelagibacteraceae* as the sister clade to the mitochondria. However, the placement of the *Pelagibacterales* near the branch point of the mitochondria and the *Rickettsiales*, regardless of the order, is in greater agreement with all three of the above studies than with the results presented by Brindefalk et al. [5], Viklund et al. [6], and Rodríguez-Ezpeleta and Embley [7], where the *Pelagibacterales* are placed elsewhere in the *Alphaproteobacteria* entirely.

*Magnetococcus marinus* is the sole genome-sequenced representative of the *Magnetococcales*, a clade that is basal to the remaining *Alphaproteobacteria* [12,32]. At the outset of this study, its membership in the *Alphaproteobacteria* was unclear, although it has since been proven correct [12]. Having chosen our outgroup to include the most diverse members of the *Betaproteobacteria*, *Gammaproteobacteria* and *Zetaproteobacteria*, we can also confirm the membership of the *Magnetococcales* within *Alphaproteobacteria*. Its inclusion in our phylogenetic analyses greatly reduced the length of the branch leading to the remaining alphaproteobacterial orders. This enabled us to circumvent an earlier problem, in which the absence of *Magnetococcus marinus* meant that the choice of outgroup affected the topology of the alphaproteobacterial tree [4].

Similarly, in our trees the *Holosporaceae* are basal to a large clade of alphaproteobacterial orders, yet they are represented solely by *Odyssella thessalonicensis* and *Caedibacter caryophilus* (Figure 2). The former was only sequenced recently [8], while the latter is represented solely by 16S and 23S sequences. Previously, *Holosporaceae* has been classified as a family in the *Rickettsiales* [10,11]; however, in our datasets this clade does not cluster within this order. Instead, it lies basal to the clade that comprises several orders, but not the *Rickettsiales* and *Magnetococcales*. In light of our new analysis, we propose to remove the *Holosporaceae* from the *Rickettsiales* and to create a new order, the *Holosporales* *ord. nov*. Consequently, under this revised classification only the *Rickettsiaceae*, the *Midichloriaceae* and the *Anaplasmataceae* comprise the order *Rickettsiales* (*sensu novo*).

There are several peculiarities involving the *Rhodospirillales* in this work and in other studies [7,10], albeit generally reported without comment. There seems to be an instability within the *Rhodospirillales* clade: whereas the *Acetobacteriaceae* resolves with high support, the *Rhodospirillaceae* rarely resolves monophyletically or with high support (this study and [7,10]). In this study the deletion of the *Rhodospirillales* profoundly reduces the support of the location of the *Holosporaceae*. The *Holosporaceae* is located in a clade with the *Rhodospirillales*, *Sphingomonadales*, *Rhizobiales*, *Caulobacterales* and *Rhodobacterales* with an average support of 82% in the trees from the complete dataset, but with only 42% support (on average) in the trees without the *Rhodospirillales*. It can be concluded that the *Rhodospirillales* play a large role in supporting the topology at the internal node of the tree. Poor support, or even paraphyly [7,10], of the *Rhodospirillales* could be indicative of an incorrect resolution of the internal node separating the clade composed of *Rickettsiales* and *Pelagibacterales* from the clade that includes the *Caulobacterales, Rhodospirillales* and several other orders. 

The problematic internal organisation of the *Rhodospirillales* is even more aggravated in the datasets that include species represented only by 16S rRNA gene sequences (Figure S20). Some species classified as *Rhodospirillales* actually belong to the separate orders *Kiloniellales*, *Kordiimonadales* and *Sneathiellales*, while several *Rhodospirillales* species, such *Tistrella mobilis* (whose genome was published too recently to be included in this study, [33]), form clades that are basal to the other *Rhodospirillales*. The implication is that the current family-level classification of the *Rhodospirillales* will need revisiting in the future.

### Proposal of new subclasses

**Figure 5:**
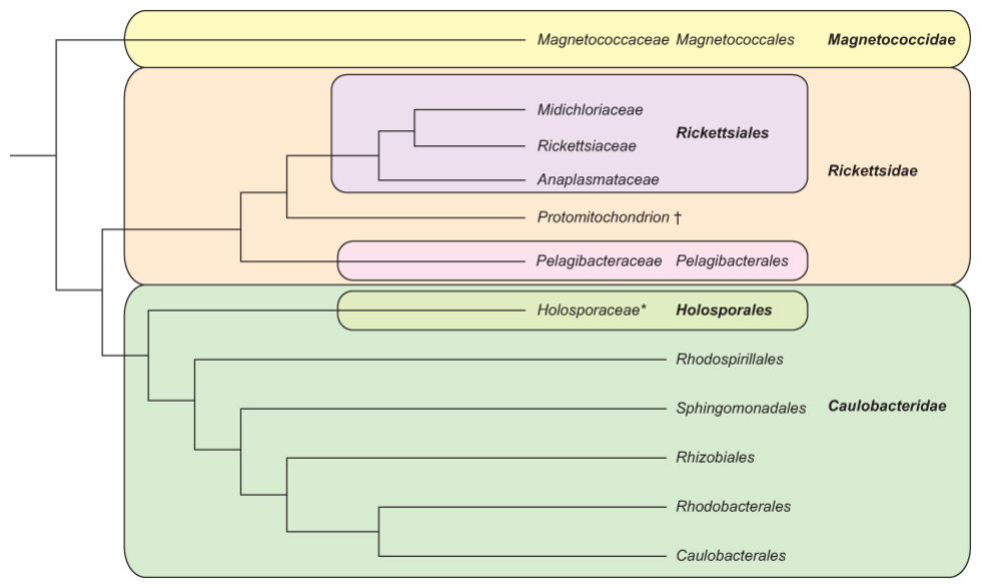
Proposed subclasses of the *Alphaproteobacteria*. The three proposed subdivisions are the *Magnetococcidae*, the *Rickettsidae* and the *Caulobacteridae*. Furthermore, the *Holosporaceae* should be removed from the *Rickettsiales*, however the identities of the family-level subdivisions of the *Holosporales*, such as the *Holosporaceae* (marked with an asterisk), are beyond the scope of this work. Under this scheme the *Rickettsiales* are comprised solely of the *Rickettsiaceae*, *Anaplasmataceae* and *Midichloriaceae*. The protomitochondrion (†) is an extinct organism that gave rise to the mitochondrial organelles of eukaryotes.

In light of the number of orders present in the *Alphaproteobacteria*, we propose the creation of three subclasses that are based on the tree topology found here (Figure 2), and that aid in the description of the groups. In particular, we propose to distinguish the two clades that are apical to the *Magnetococcales*. Therefore we propose to place: (i) the *Magnetococcales* in the *Magnetococcidae subcl. nov.*; (ii) the *Rickettsiales* (*sensu novo*), the protomitochondrion (i.e. the bacterial ancestor of the eukaryotic organelle) and *Pelagibacterales* *ord. nov.* in the *Rickettsidae subcl. nov*.; and (iii) the *Holosporales* *ord. nov.*, *Rhodospirillales*, *Sphingomonadales*, *Rhizobiales*, *Caulobacterales* and other orders in the *Caulobacteridae subcl. nov*. Our proposal is summarized in Figure 5. Overall, our updated analysis supports the position of the *Pelagibacterales* as a sister group to the composite clade containing *Rickettsiales* and the mitochondrial branch. Our analysis has also provided support for taxonomic assignment of several recently sequenced species, including *Odyssella thessalonicensis* and *Magnetococcus marinus*.

## Materials and Methods

### Taxon selection

The 16S and 23S rRNA genes used to construct the concatenated datasets were obtained from IMG (v350). The perl scripts *list_clustermaker.pl* and *fasta_acceptor.pl* were used to select a sole sequence as a representative for each species present while checking whether the sequence matched that of the other paralogs. In negative cases, the most common sequence was chosen. To supplement the trees with additional *Alphaproteobacteria* without sequenced genomes, 16S or both 16S and 23S rRNA gene sequences were obtained from GenBank.

### GC content

A perl script, *GC-counter.pl*, was used to determine the GC content of the rRNA gene sequences; genomic GC content was obtained from IMG. Scatter plots were created in Prism 4. The slopes of the linear regressions of both the SSU and the LSU rRNA gene GC content over genomic GC content for the *Alphaproteobacteria* and for the mitochondria were calculated in Excel with the *slope* function. Box plots were created in R using *boxplot*, and statistical significance was evaluated using *wilcox*.*test*.

### Sequence QC

The quality of the sequences was corrected by trimming the ends according to the match from the Arb-Sina aligner [24]. The length was checked with a perl script, *impostor* *checker.pl*, and all sequences under 1200 (for 16S) or 2000 (for 23S) bases were removed. One outcome of imparting this size cut-off was to remove numerous mitochondrial sequences (listed in Table S1); however, including these mitochondrial sequences made no difference to the observed topologies (data not shown). The presence of multiple copies of rDNA in most organisms allowed sequencing errors and contaminations to be identified.

### Alignment/gap-removal

The sequences were aligned either with Muscle [23] under default settings or with Sina [24] set to remove terminal unaligned bases and not to reverse complement sequences (due to issues with some mitochondrial sequences). Poorly aligned sites were curated with Gblocks [34] using the settings from [35]: -b1=(n/2)+1 -b2=(n/2)+1 -b3=n/2 -b4=2 -b5=h. All dataset variants were created by deleting the targeted taxa before the alignment step. 

### RY-, MK- and RYMK-recoded datasets

For the RY-recoded datasets, the completed (aligned and trimmed) datasets were recoded, by converting all A and G bases to R and all T and C bases to Y. For the MK-recoded datasets, all A and C bases were converted to M and all T and G bases were converted to K. For the RYMK-recoded datasets, the complete dataset was duplicated and one copy was RY-recoded, while the second was MK-recoded; the two differentially recoded variants were then concatenated. 

### Maximum likelihood trees

Maximum likelihood inferences were performed with RAxML 7.2.9 [25] with 1,000 bootstrap replicates under either a GTRΓ or a GTRCAT model. The trees were displayed in FigTree3.1 [36]. Figure 2 was further annotated with Adobe Illustrator CS4. The supplementary tree summary figures were made with Illustrator CS4, with the aid of Newick utilities [37] for data extraction. All alignment and tree files are available from the authors upon request.

### Topology evaluation

The bootstrap support for various groupings of interest were obtained via the perl script *descriptor.pl*, using Newick utilities [37]. First the bootstrap trees were rooted to the outgroup (*nw_reroot*), then the leaves in these were renamed by mapping the names to the groups of interest (*nw_rename*), after which the clades composed of a single name were condensed (*nw_condense*) and finally the trees were ordered (*nw_order*). Once this was done, the script determined the support for monophyly of the groupings by simply counting the frequency of the names in each of the resulting trees, where a single instance would indicate monophyly.

### Approximately Unbiased tests

The final trees of the full datasets were concatenated into a single file and the per site log-likelihoods were calculated with both the Arb-Sina-aligned and Muscle-aligned datasets (RAxML via option -*f g*), in order to be converted into a matrix (*makermt --puzzle*) that could be interpreted by Consel (*consel* and *catpv*). Robinson-Foulds distances were calculated between each tree in a set with *HashRF* (https://code.google.com/p/hashrf/).

## Supporting Information

Figure S1 **Relationship between the GC-content of the genome and that of the rRNA gene sequences in the *Alphaproteobacteria* and the mitochondria after alignment and removal of gap-rich regions.** The removal of the highly variable stretches of the rRNA gene sequences did not substantially alter the difference between the GC-content of these sequences and that of the genome.
(EPS)

Figure S2 **Relationship between the GC-content of the genome and that of the rRNA gene sequences in the *Alphaproteobacteria* and the mitochondria after alignment and removal of gap-rich regions and invariant sites.** The removal of the invariant sites does not substantially alter the relationship, therefore the reduced GC-content difference for the rRNA gene sequences is not a product of the invariant sites.
(EPS)

Figures S3 **Summary of bootstrap supports.** The values in the boxes are the bootstrap support percentages for the given bipartitions from each of the eight inferences for each set (with or without mitochondria, aligned with Arb-Sina or Muscle, GTRΓ or GTRCAT model). In the cases where the final tree did not agree with the proposed topology on the location of a clade the bootstrap support for the proposed bipartition is represented in red, despite being absent in the final tree.
(EPS)

Figures S4 **Summary of bootstrap supports.** The values in the boxes are the bootstrap support percentages for the given bipartitions from each of the eight inferences for each set (with or without mitochondria, aligned with Arb-Sina or Muscle, GTRΓ or GTRCAT model). In the cases where the final tree did not agree with the proposed topology on the location of a clade the bootstrap support for the proposed bipartition is represented in red, despite being absent in the final tree.
(EPS)

Figure S5 **Taxonomic misallocation.** The number of bootstrap trees with polyphyletic *Rhodobacterales* is greatly reduced when species of *Labrenzia*, *Roseibium* and *Pseudovibrio* are moved from the *Rhodobacterales* to the *Rhizobiales*. The values were calculated by pruning the other suspect leaves from the replicate trees. Furthermore all eight primary trees resolve this clade in the *Rhizobiales*.
(TIF)

Figure S6 **Summary of bootstrap supports.** The values in the boxes are the bootstrap support percentages for the given bipartitions from each of the eight inferences for each set (with or without mitochondria, aligned with Arb-Sina or Muscle, GTRΓ or GTRCAT model). In the cases where the final tree did not agree with the proposed topology on the location of a clade the bootstrap support for the proposed bipartition is represented in red, despite being absent in the final tree.
(EPS)

Figure S7 **Summary of bootstrap supports.** The values in the boxes are the bootstrap support percentages for the given bipartitions from each of the eight inferences for each set (with or without mitochondria, aligned with Arb-Sina or Muscle, GTRΓ or GTRCAT model). In the cases where the final tree did not agree with the proposed topology on the location of a clade the bootstrap support for the proposed bipartition is represented in red, despite being absent in the final tree.
(EPS)

Figure S8 **Summary of bootstrap supports.** The values in the boxes are the bootstrap support percentages for the given bipartitions from each of the eight inferences for each set (with or without mitochondria, aligned with Arb-Sina or Muscle, GTRΓ or GTRCAT model). In the cases where the final tree did not agree with the proposed topology on the location of a clade the bootstrap support for the proposed bipartition is represented in red, despite being absent in the final tree.
(EPS)

Figure S9 **Summary of bootstrap supports.** The values in the boxes are the bootstrap support percentages for the given bipartitions from each of the eight inferences for each set (with or without mitochondria, aligned with Arb-Sina or Muscle, GTRΓ or GTRCAT model). In the cases where the final tree did not agree with the proposed topology on the location of a clade the bootstrap support for the proposed bipartition is represented in red, despite being absent in the final tree.
(EPS)

Figure S10 **Tree inferred with the Arb-sina aligned ‘combo’ dataset under a GTRΓ model.** This dataset included strains that were represented solely by a 16S sequence, therefore revealing the diversity that is not covered by genome sequences. Bootstrap values (n = 1,000) are indicated at the nodes. Red arrows indicate how a taxon or clade differs in the other trees. Insets show clades which are particularly rich in unsequenced genomes (black text) compared to sequenced genomes (green text). The internal topology of the *Rhodospirillales* differs between trees, but the six constant subdivisions are highlighted.
(EPS)

Figure S11 **16S and 23S rRNA gene trees for the complete dataset, with and without mitochondria.**
(PDF)

Figure S12 **Regular-coded complete dataset trees, with and without mitochondria.**
(PDF)

Figure S13 **A.** RY-coded complete dataset trees, with and without mitochondria. **B**. MK-coded complete dataset trees, with and without mitochondria.
(PDF)

Figure S14 **RYMK-coded complete dataset trees, with and without mitochondria.**
(PDF)

Figure S15 **Regular-coded trimmed dataset trees, with and without mitochondria.**
(PDF)

Figure S16 **A.** RY-coded trimmed dataset trees, with and without mitochondria. **B**. MK-coded trimmed dataset trees, with and without mitochondria.
(PDF)

Figure S17 **RYMK-coded trimmed dataset trees, with and without mitochondria.**
(PDF)

Figure S18 **Regular-coded complete dataset mtDel trees.**
(PDF)

Figure S19 **Jackknifing trees, with and without mitochondria.**
(PDF)

Figure S20 **Trees including 16S only sequences, with and without mitochondria.**
(PDF)

Table S1 **Summary of taxa used in this study.**
(DOCX)

Table S2 **Approximately Unbiased (AU) test results.**
(XLSX)
