# Supplementary figures and images for: New rRNA Gene-Based Phylogenies of the Alphaproteobacteria Provide Perspective on Major Groups, Mitochondrial Ancestry and Phylogenetic Instability
Source: PLoS One. 2013 Dec 11;8(12):e83383. doi: 10.1371/journal.pone.0083383 (PMC3859672; doi:10.1371/journal.pone.0083383)

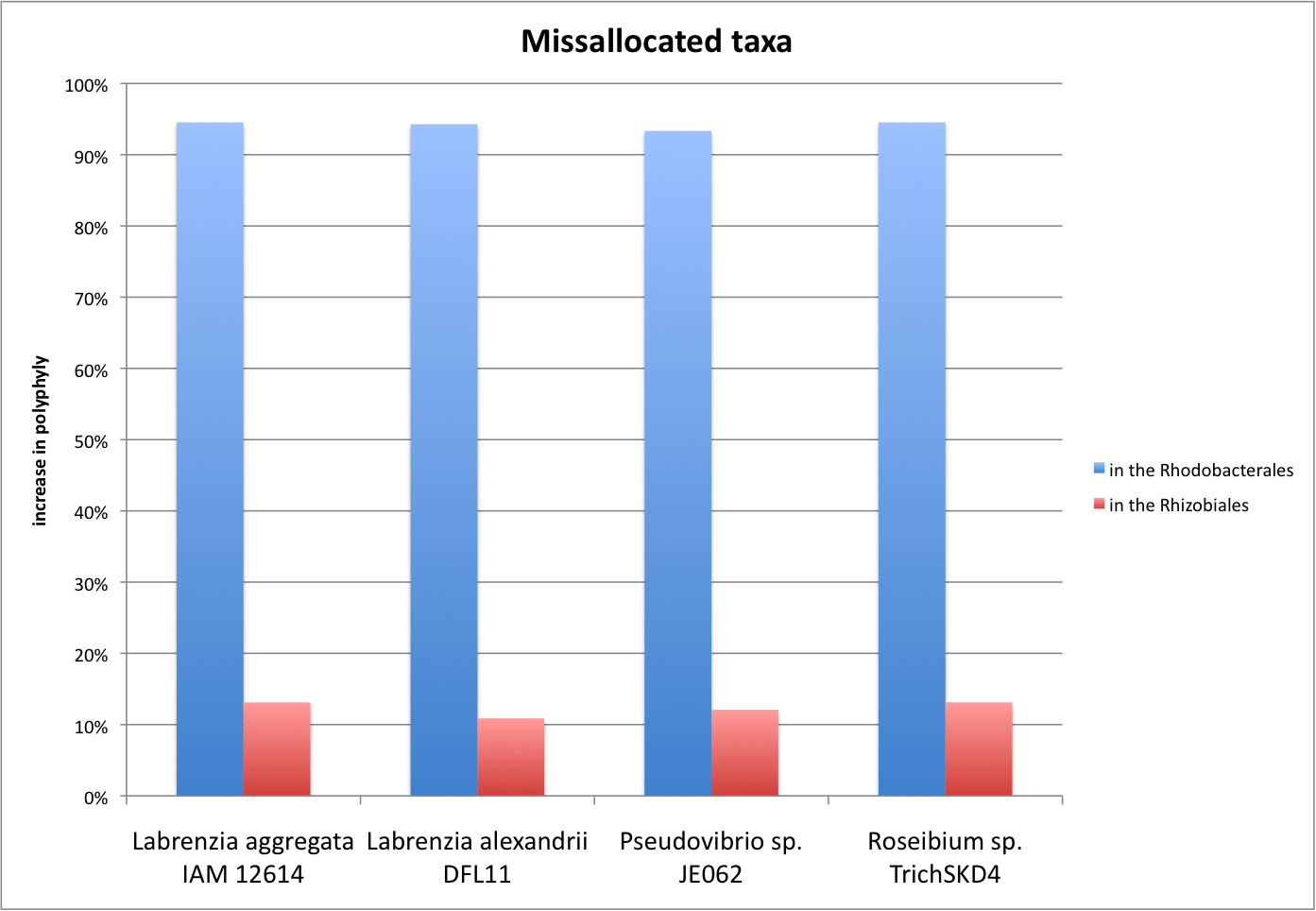

Supplement: Figure S5 — Taxonomic misallocation. The number of bootstrap trees with polyphyletic Rhodobacterales is greatly reduced when species of Labrenzia, Roseibium and Pseudovibrio are moved from the Rhodobacterales to the Rhizobiales. The values were calculated by pruning the other suspect leaves from the replicate trees. Furthermore all eight primary trees resolve this clade in the Rhizobiales. (TIF) [file pone.0083383.s005.tif]

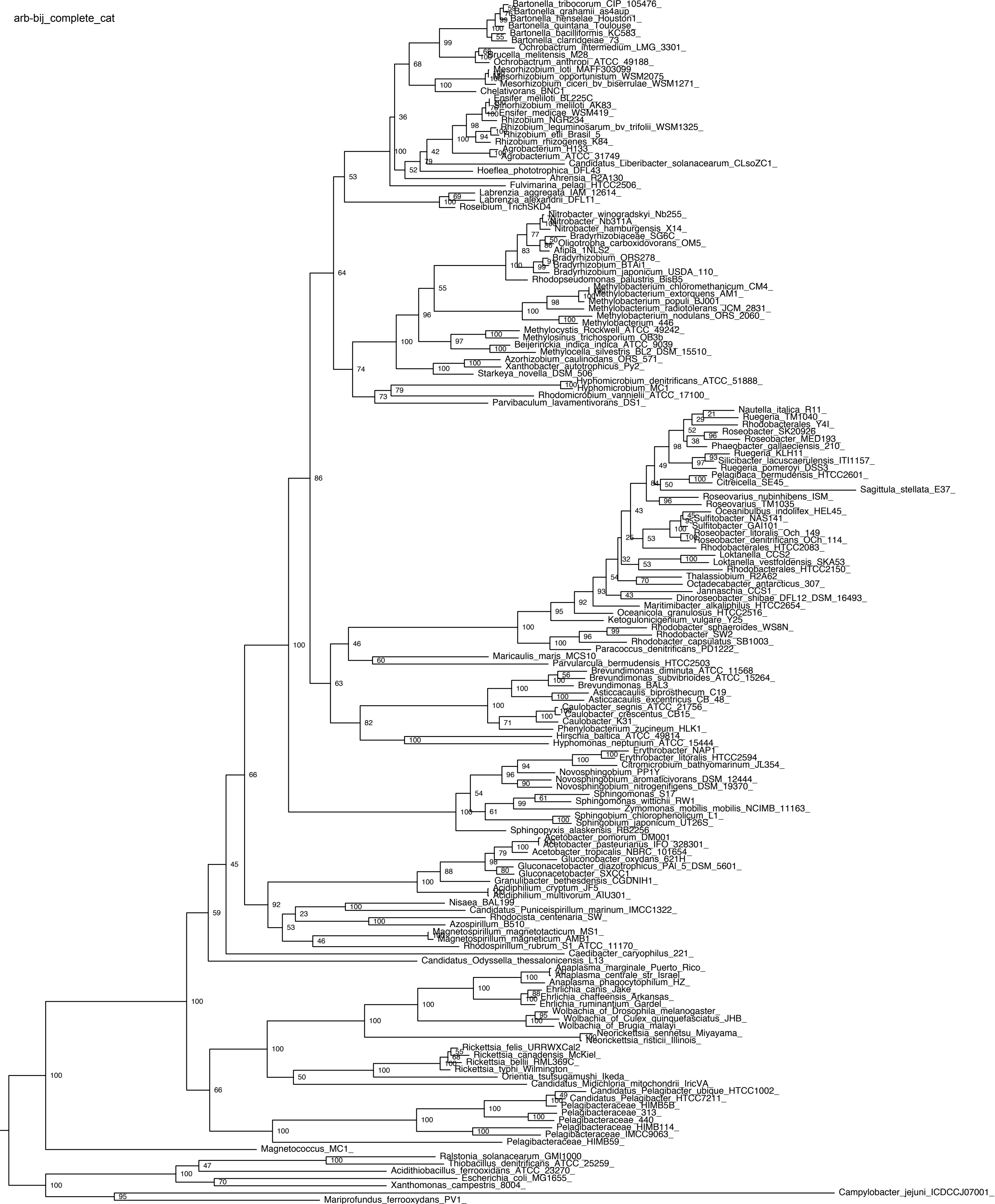

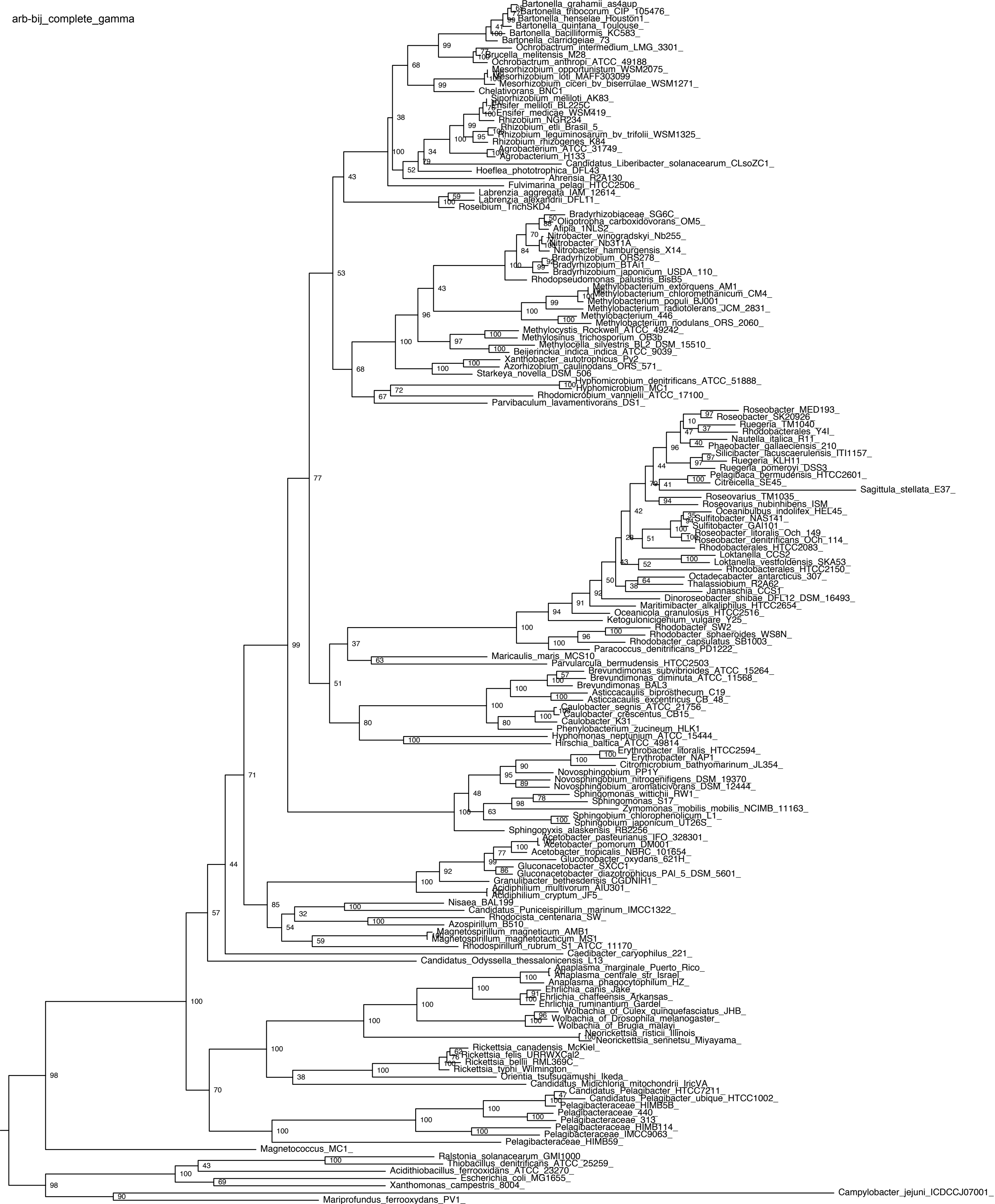

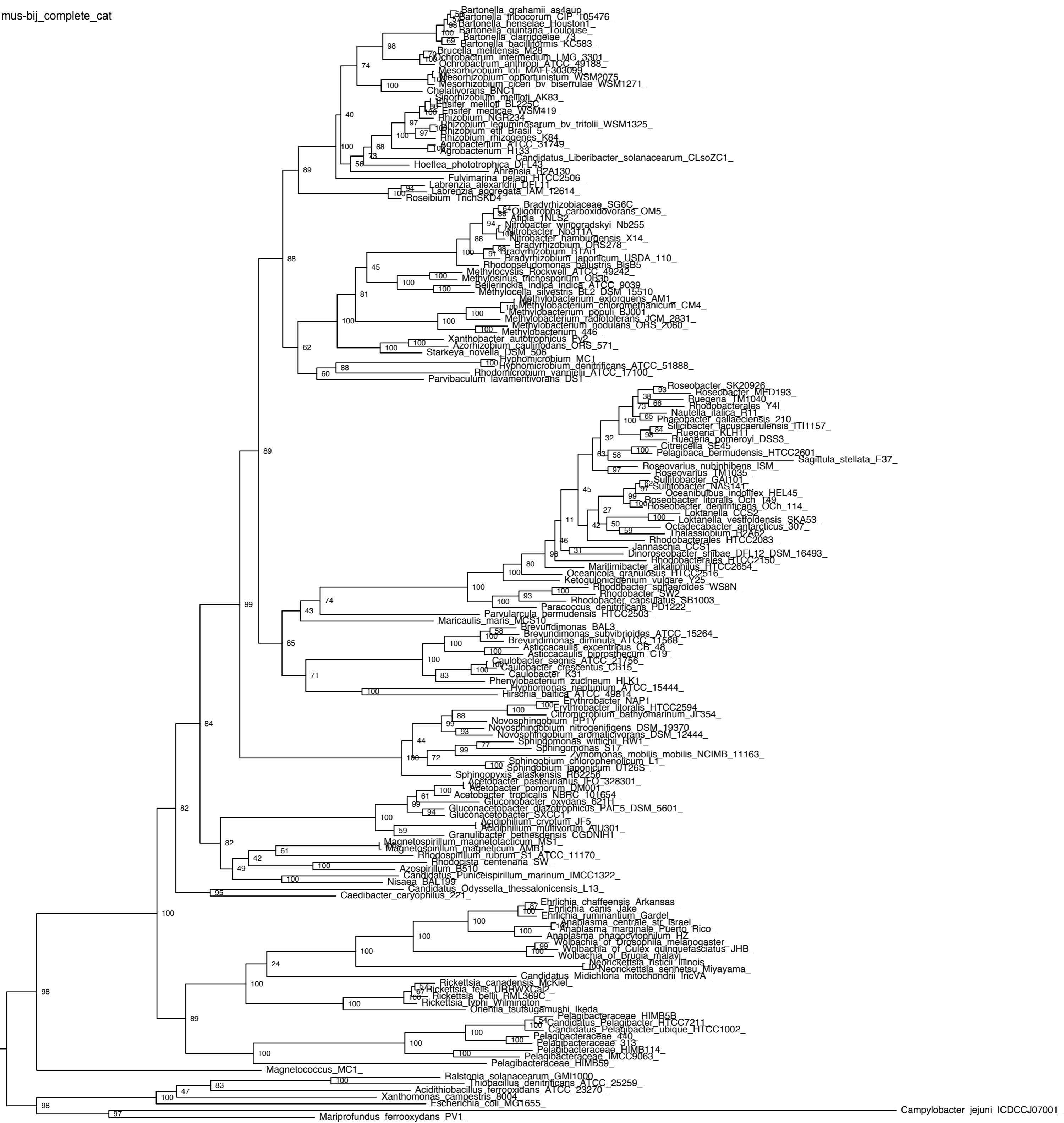

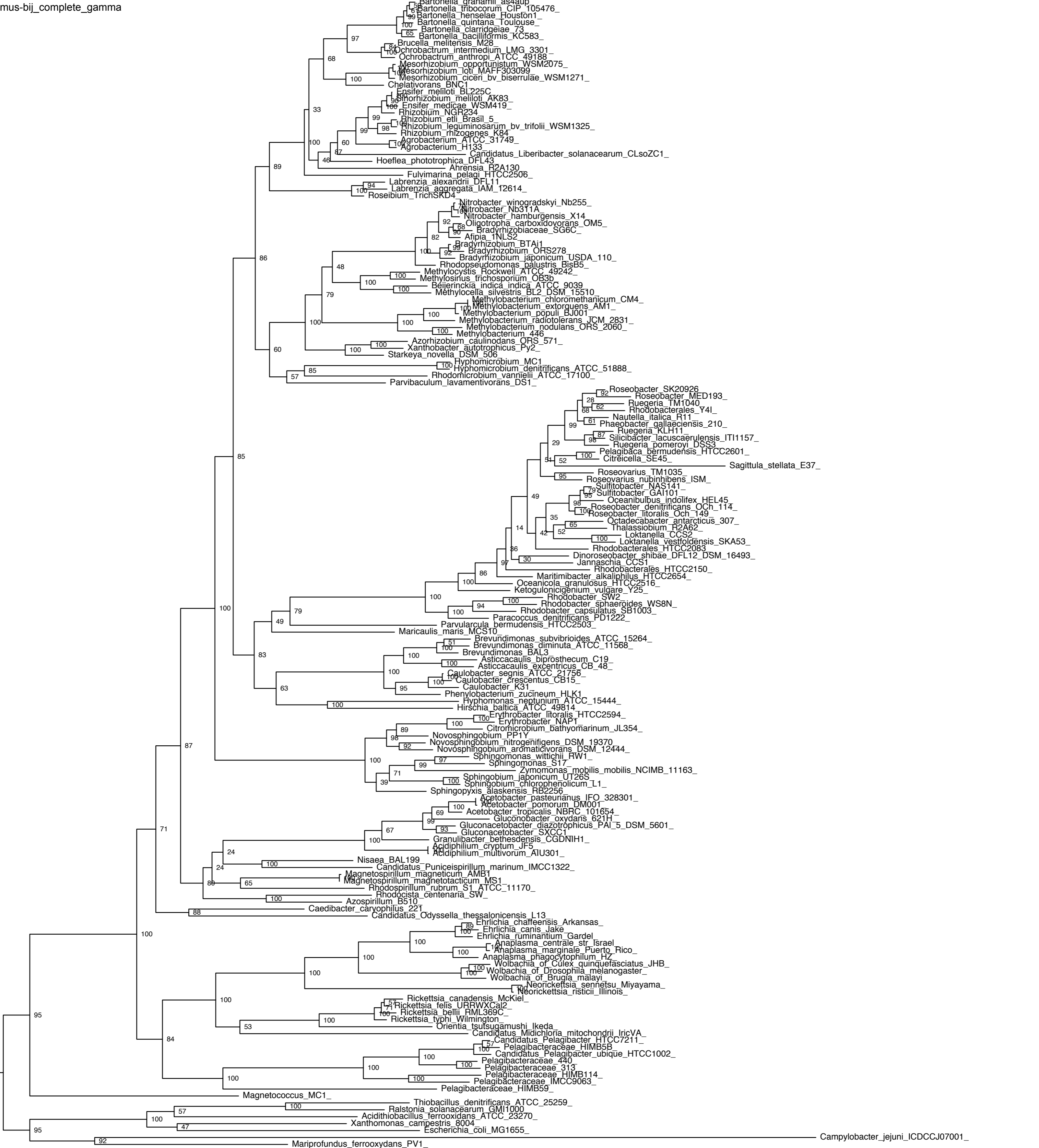

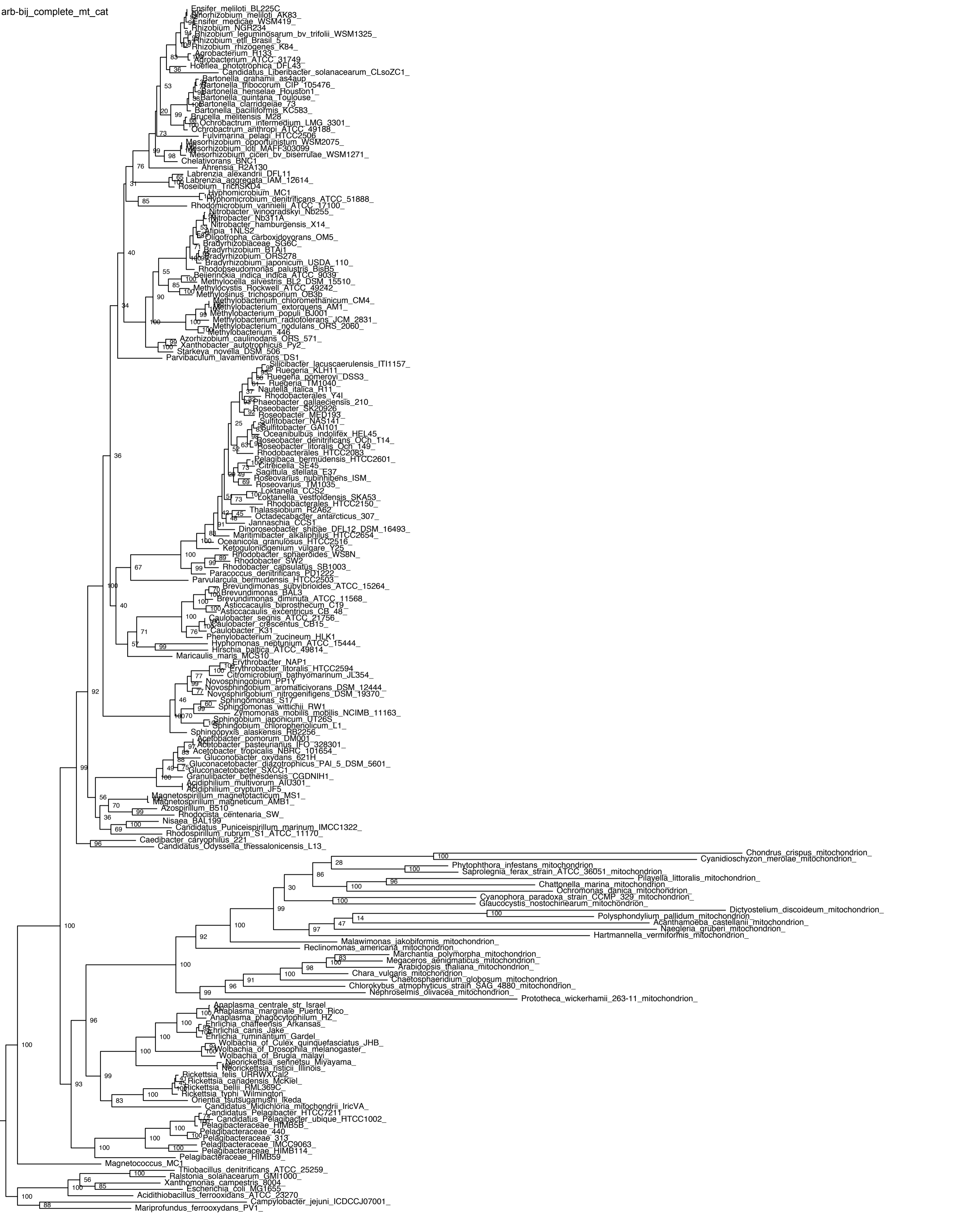

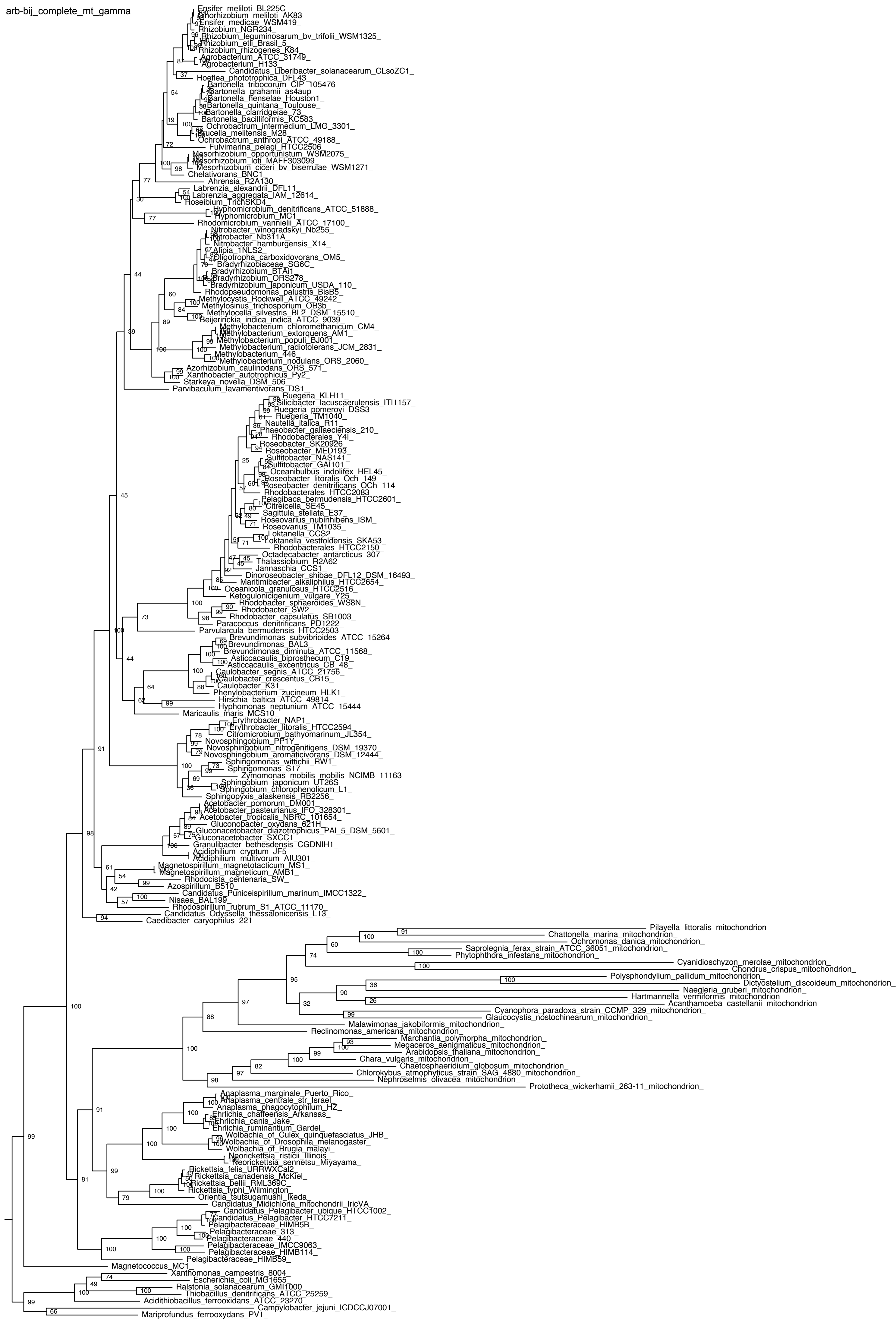



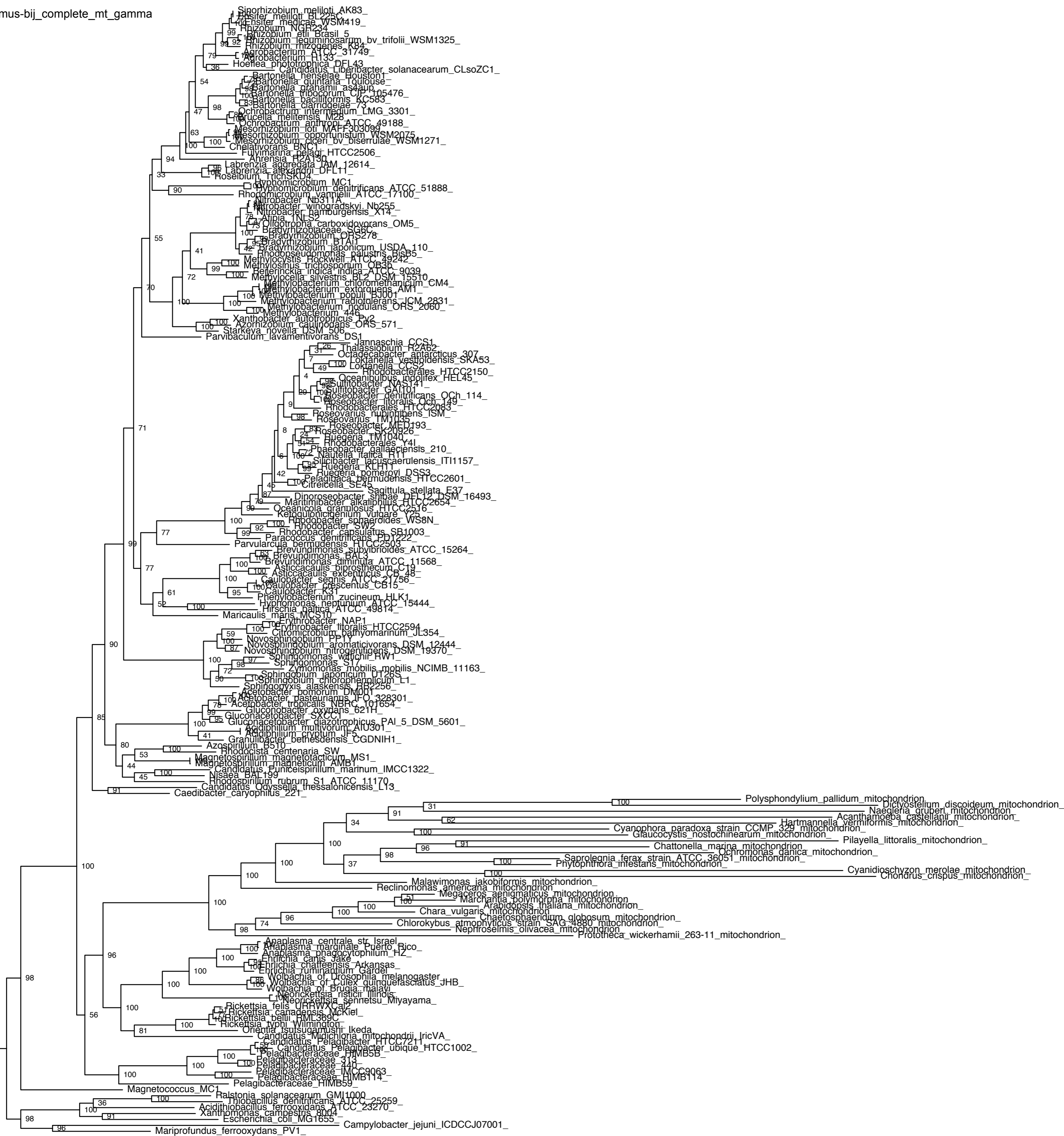

Supplement: Figure S12 — Regular-coded complete dataset trees, with and without mitochondria. (PDF) [file pone.0083383.s012.pdf]

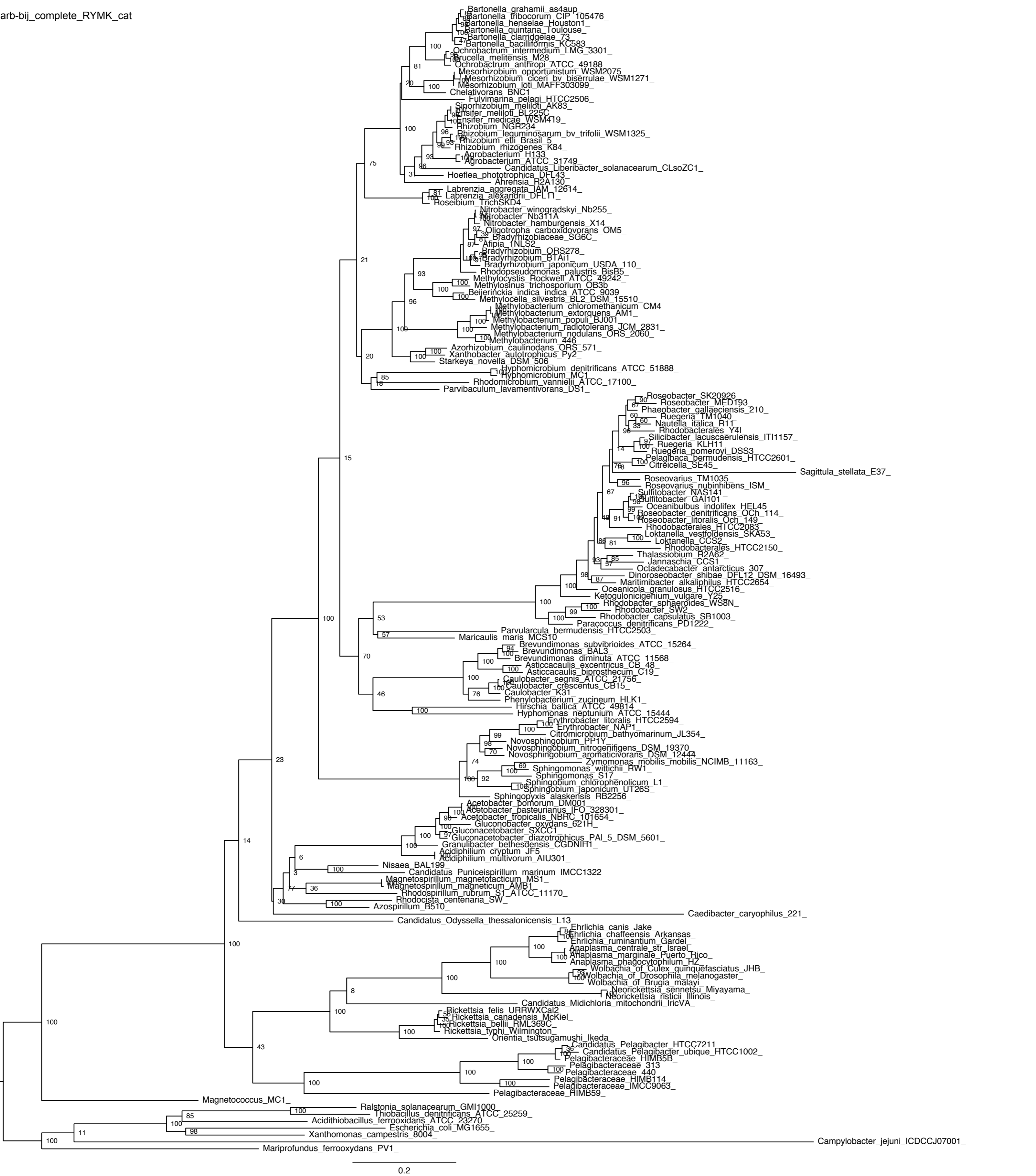

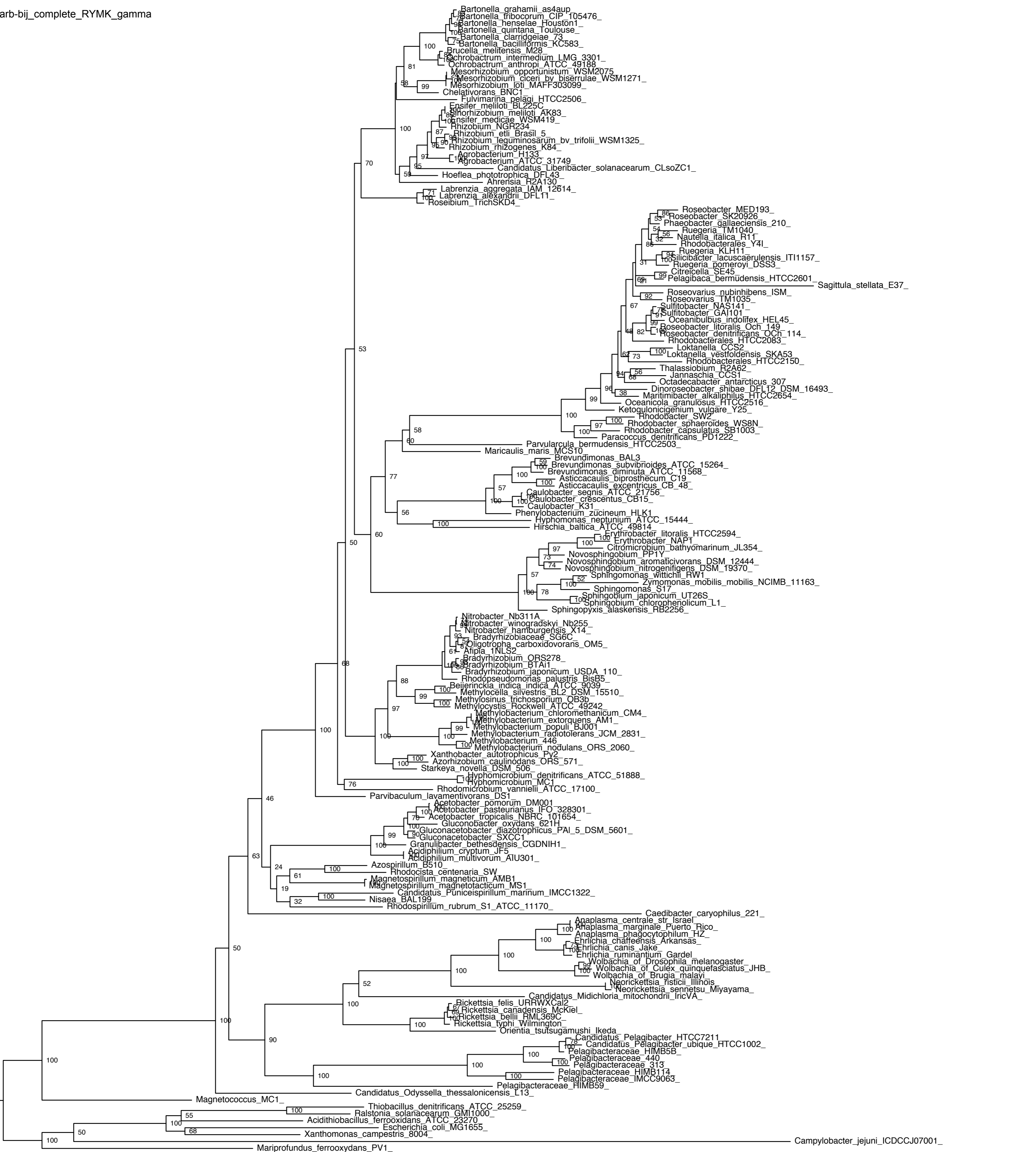

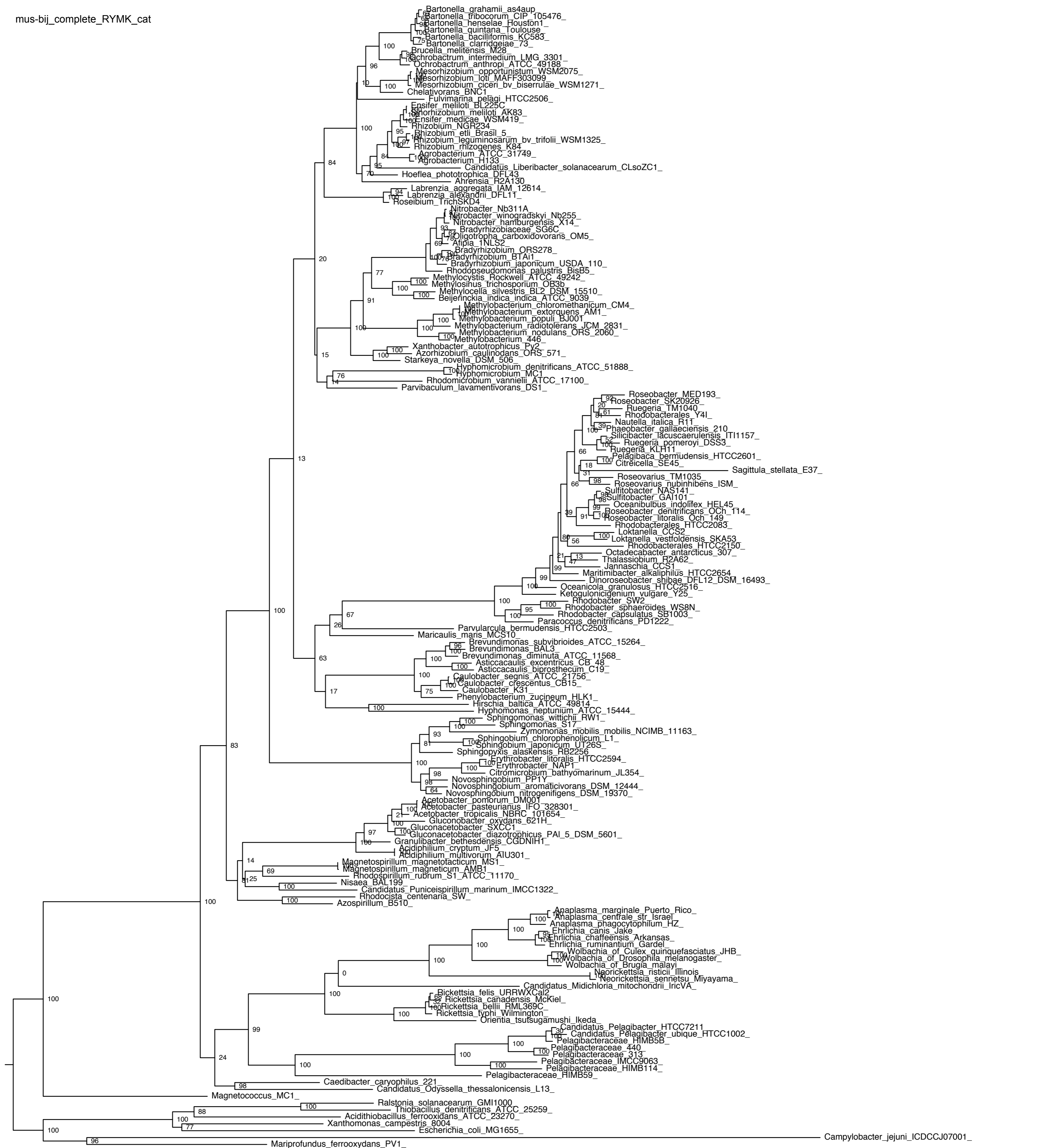

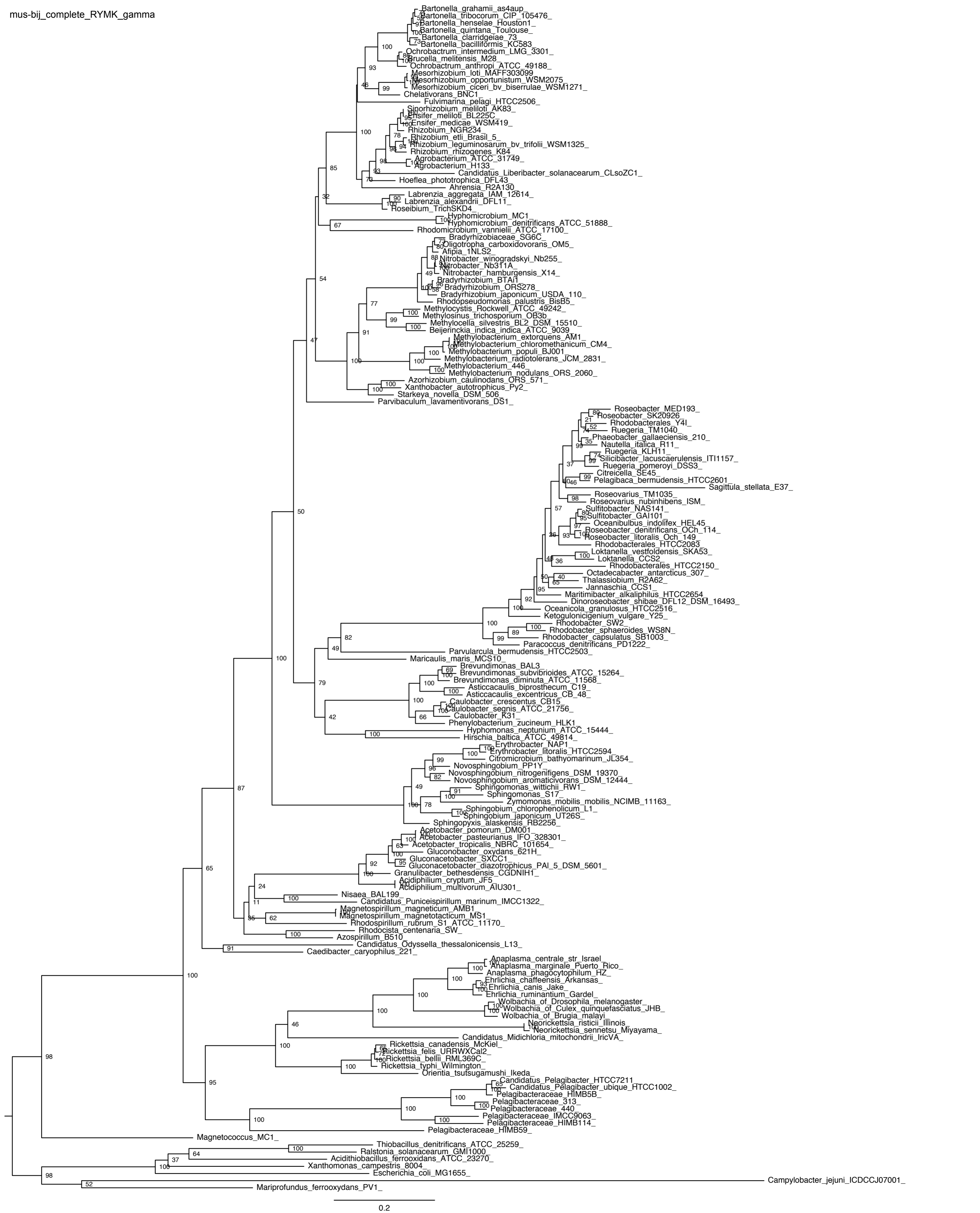

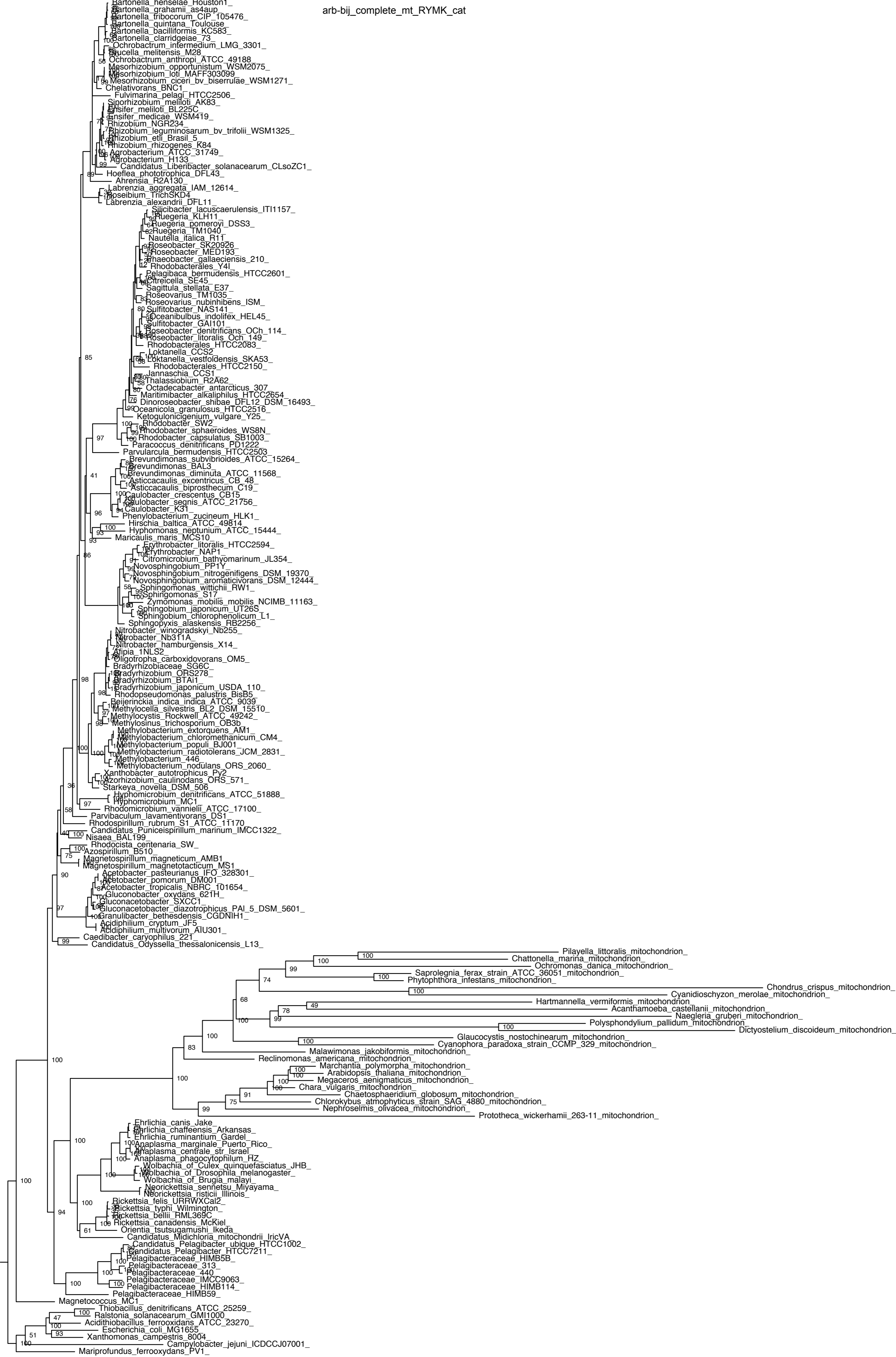

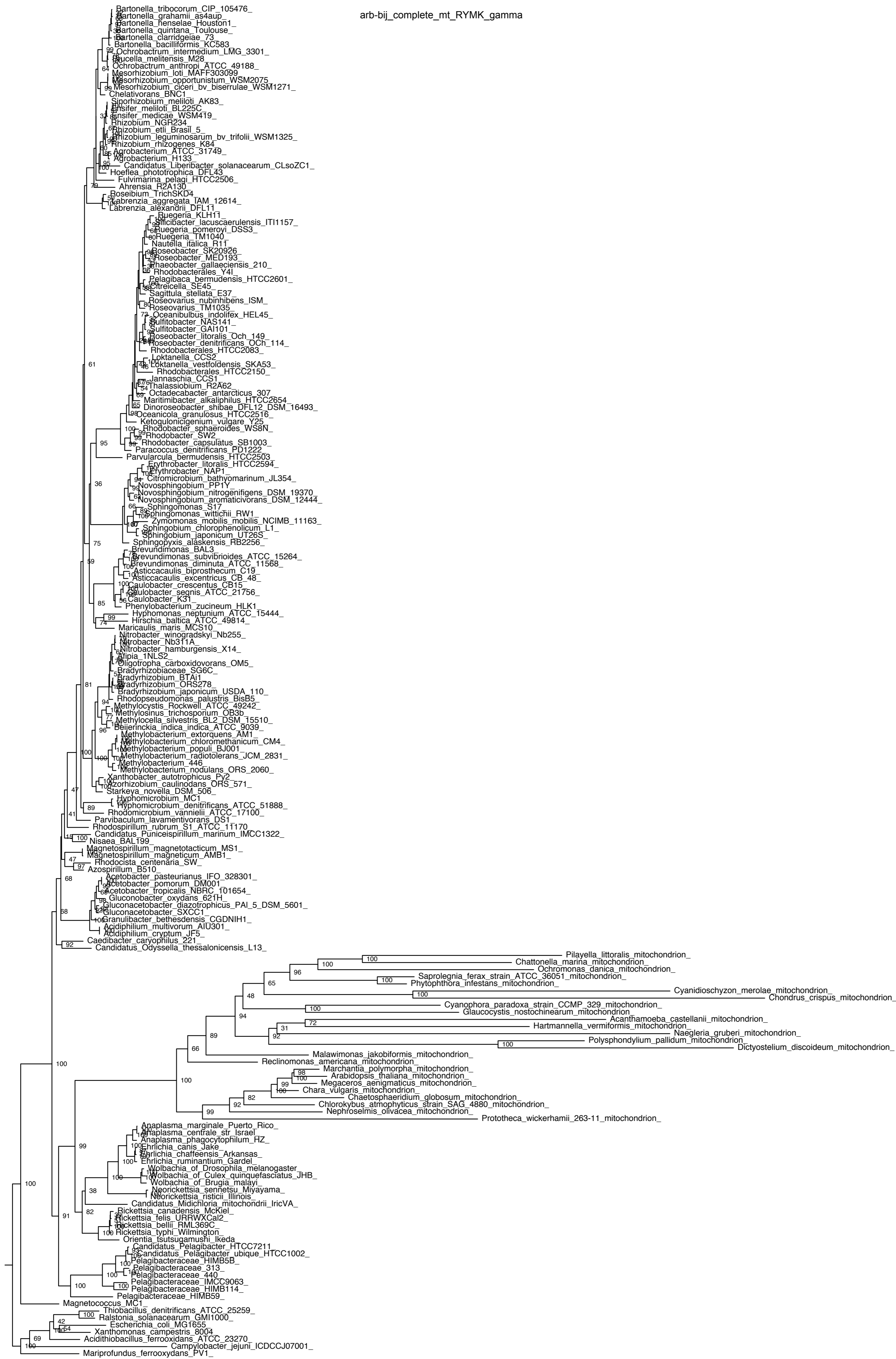

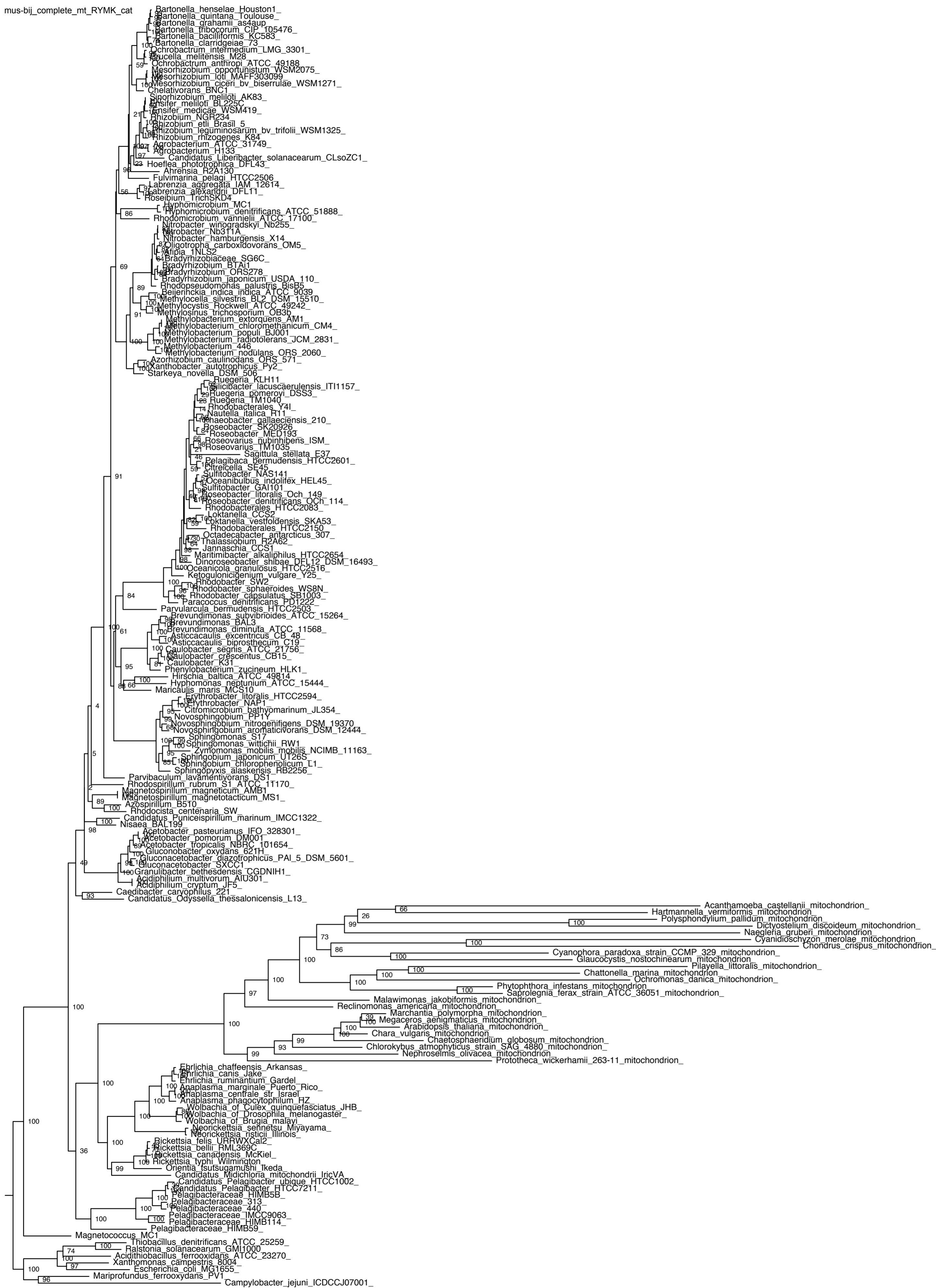

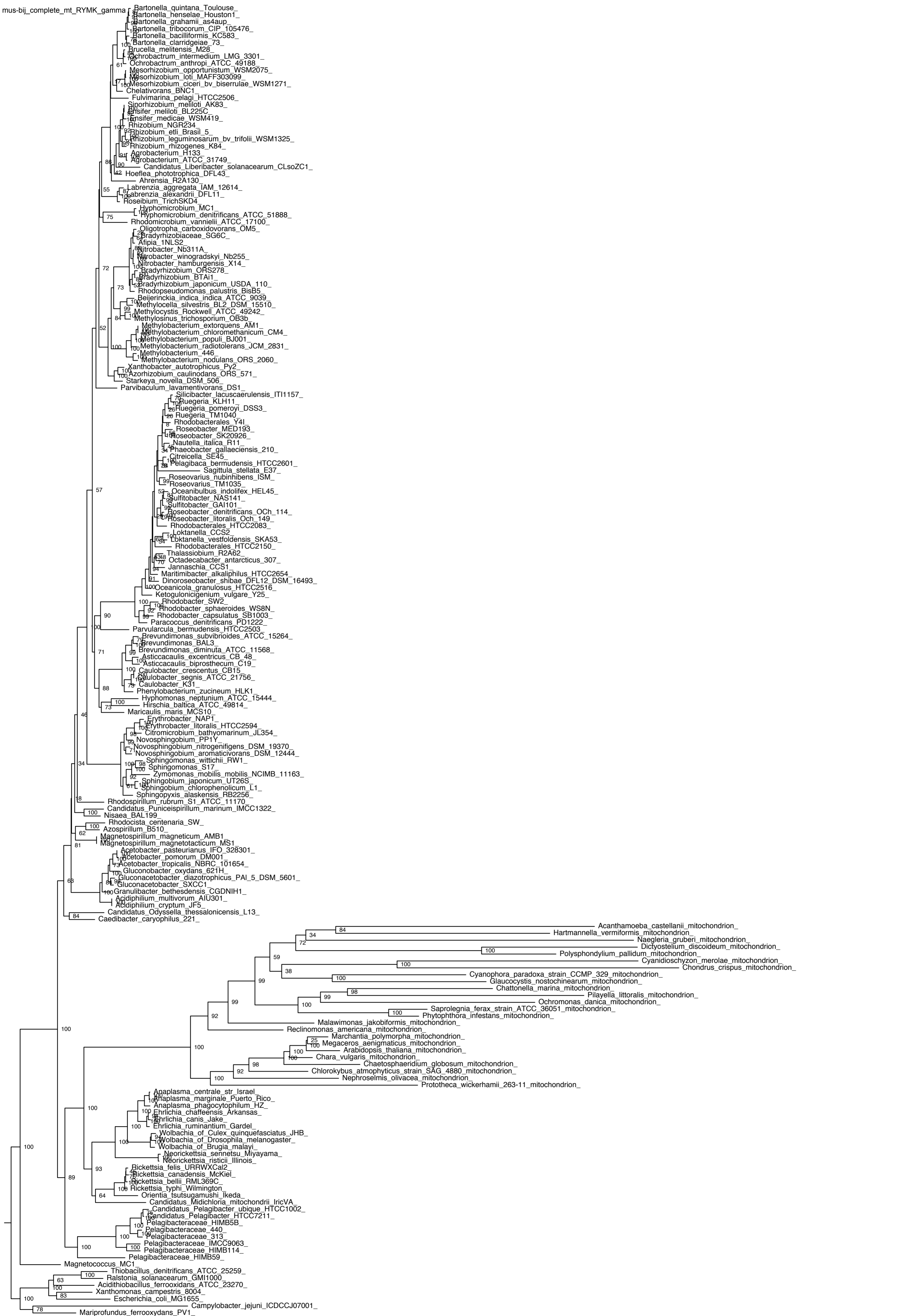

Supplement: Figure S14 — RYMK-coded complete dataset trees, with and without mitochondria. (PDF) [file pone.0083383.s014.pdf]

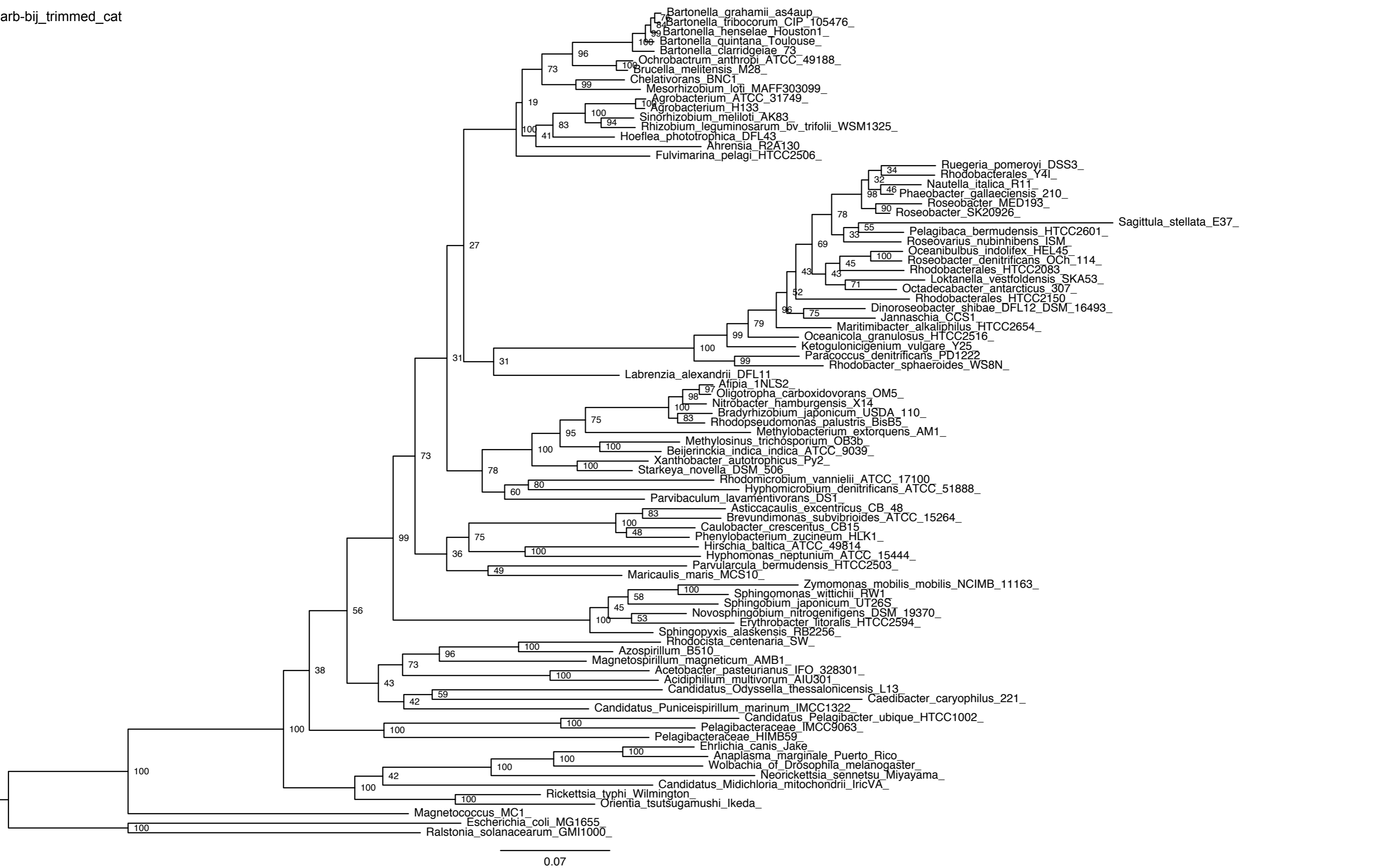

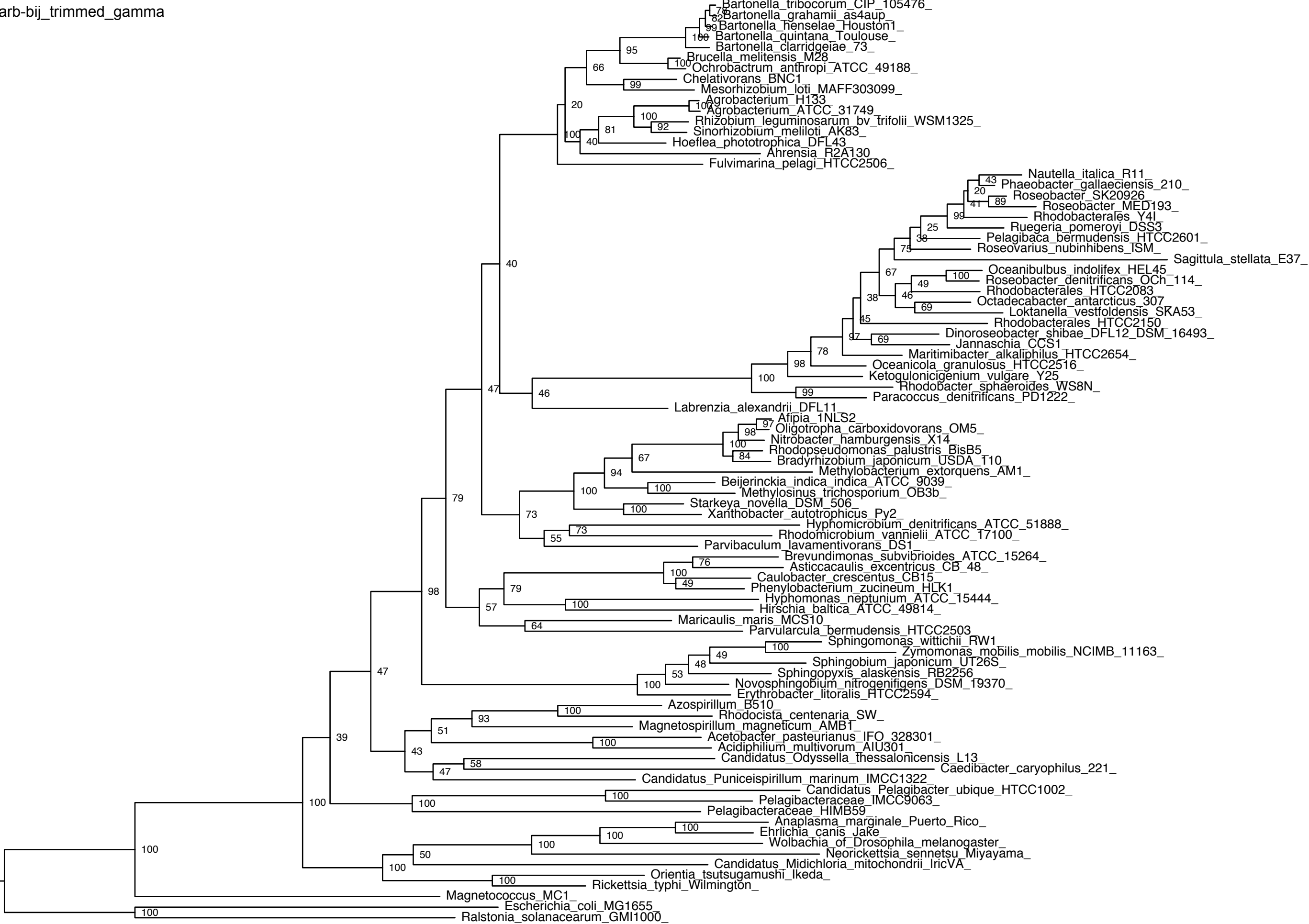

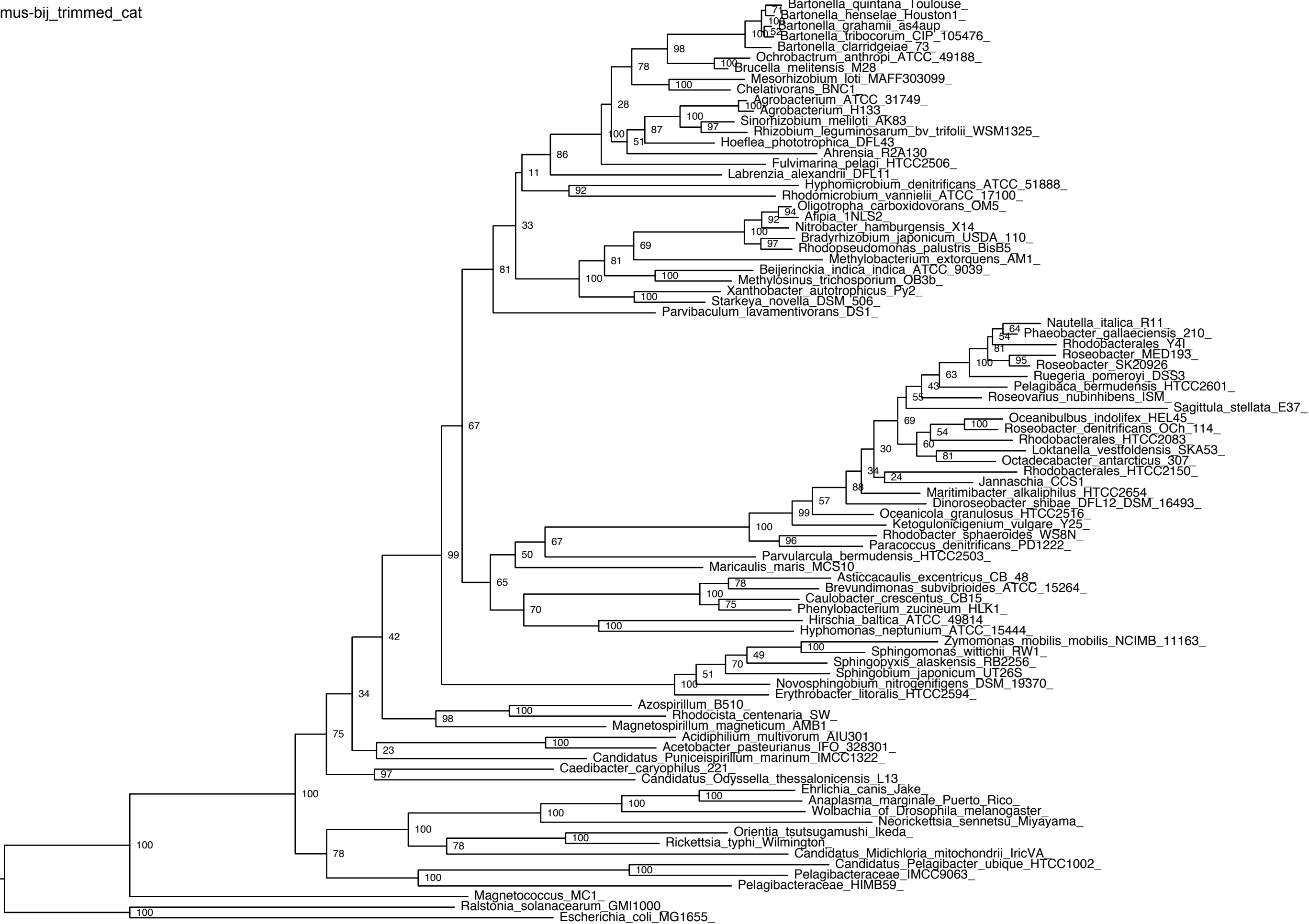

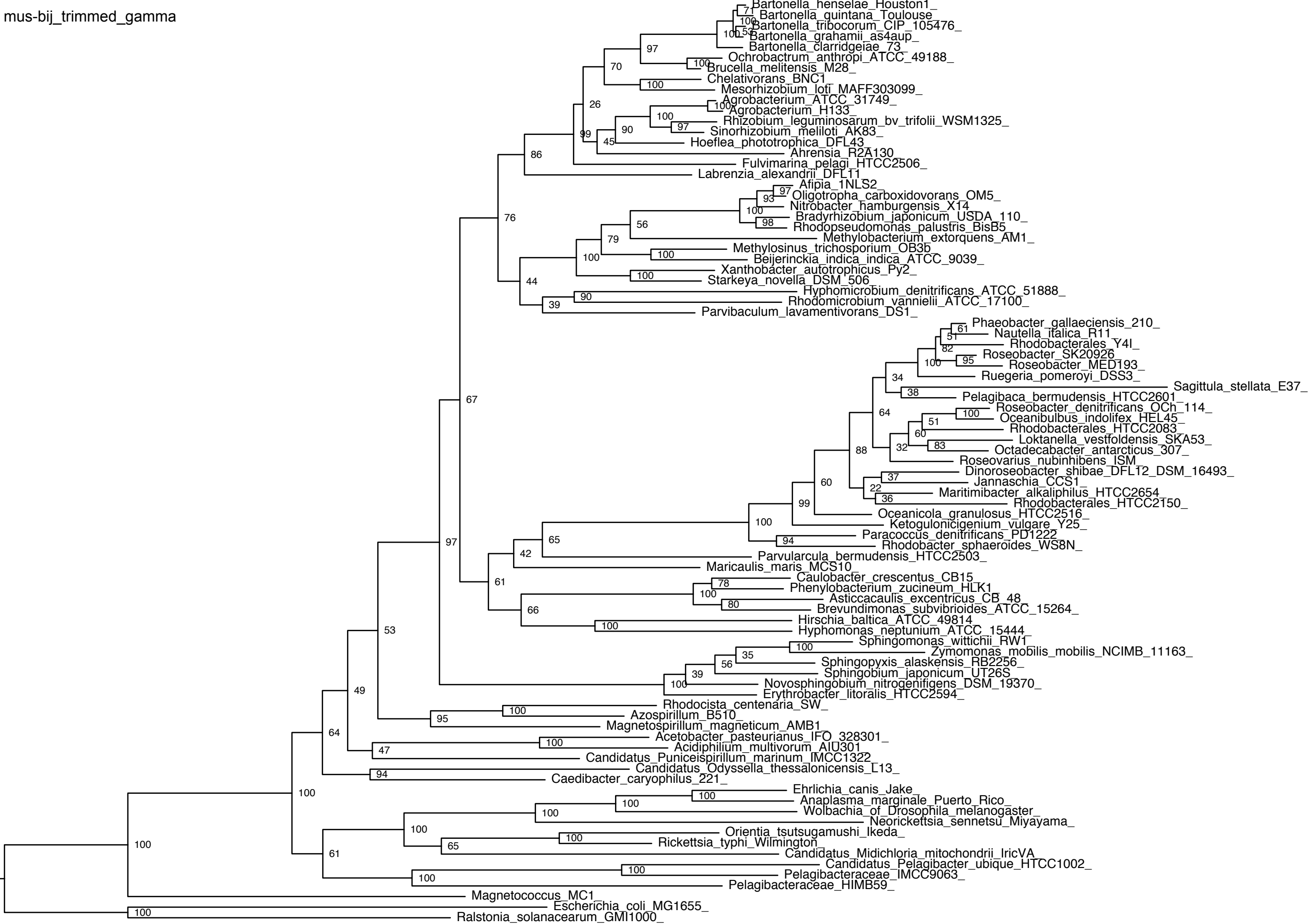

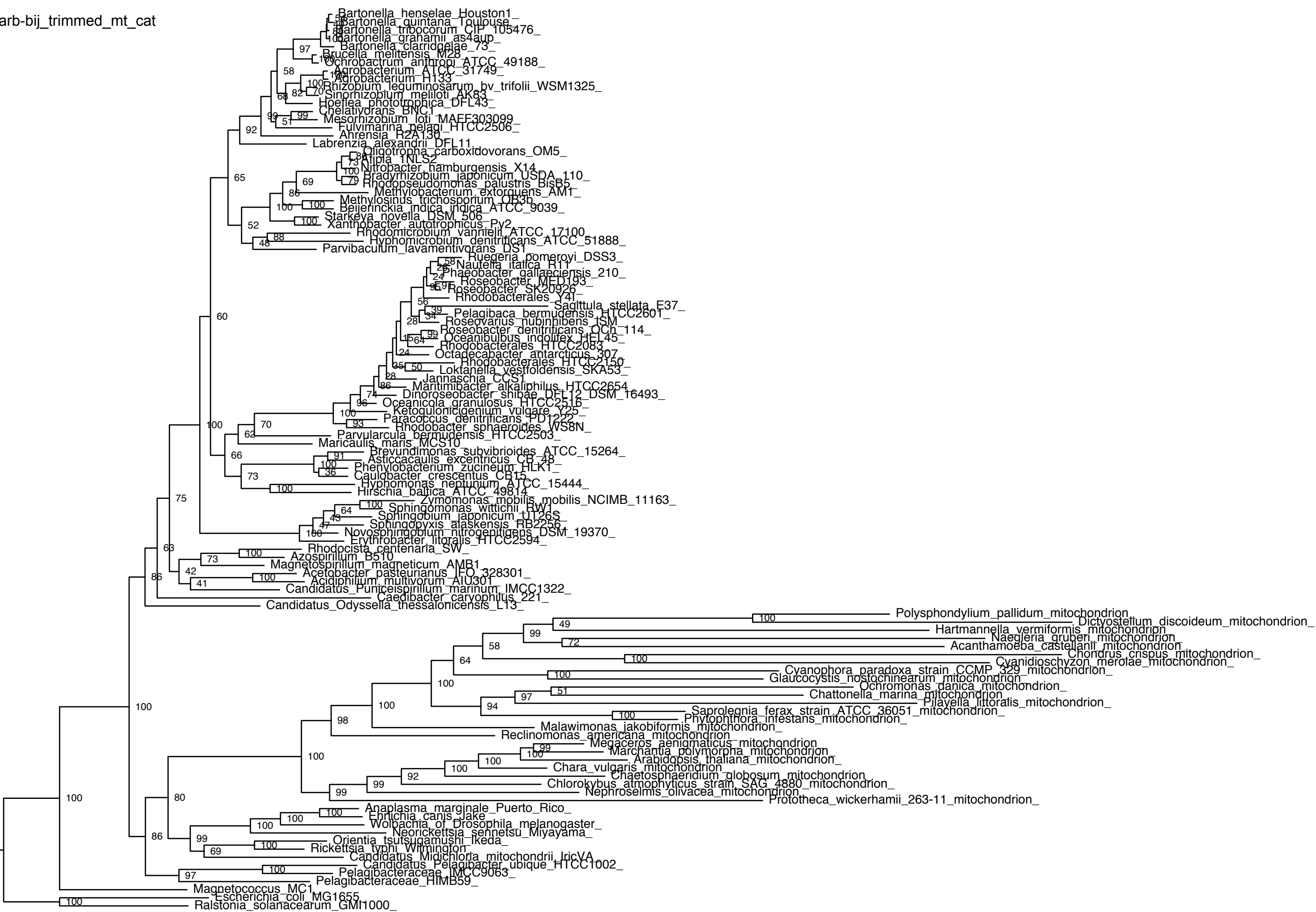

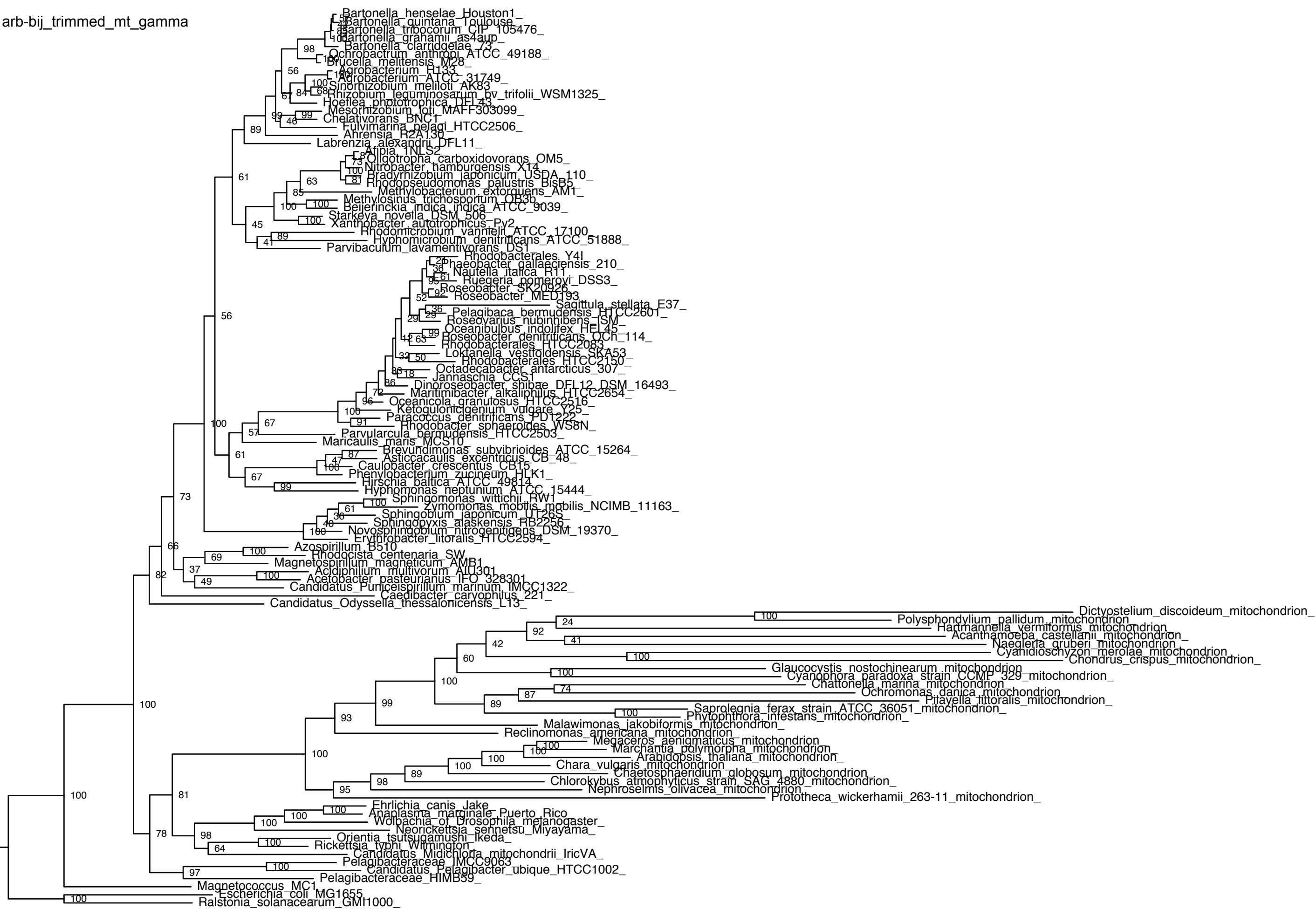

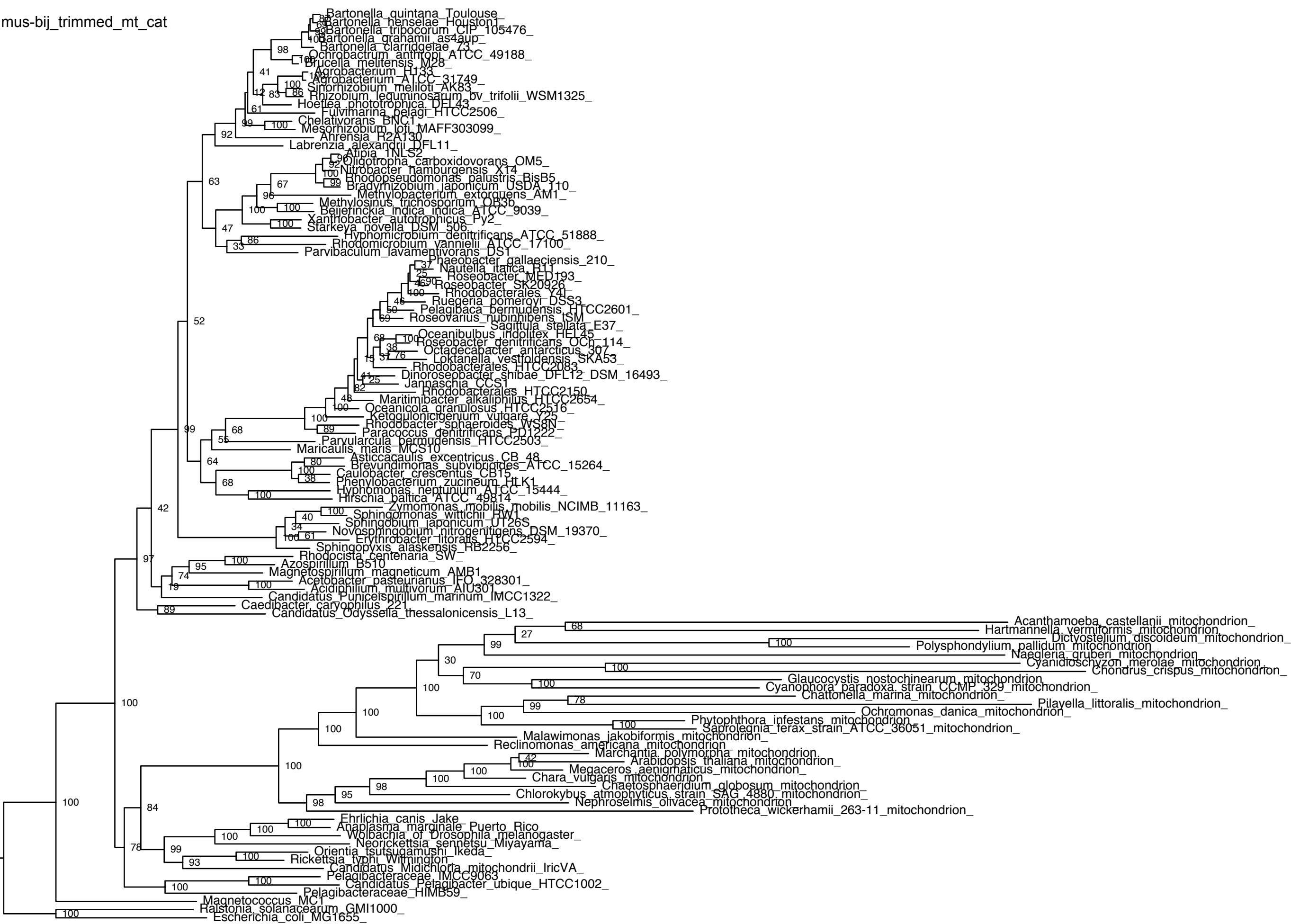

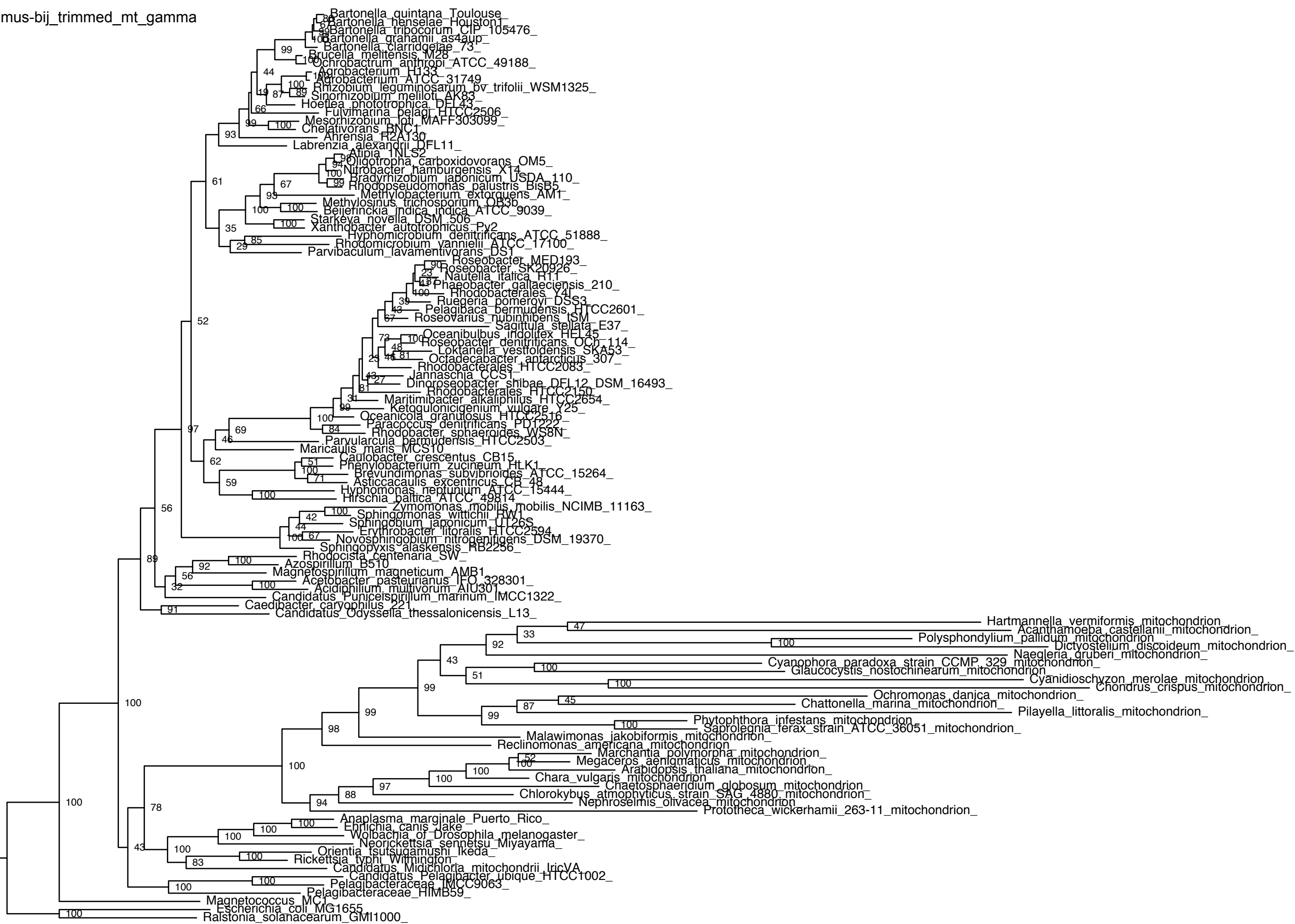

Supplement: Figure S15 — Regular-coded trimmed dataset trees, with and without mitochondria. (PDF) [file pone.0083383.s015.pdf]

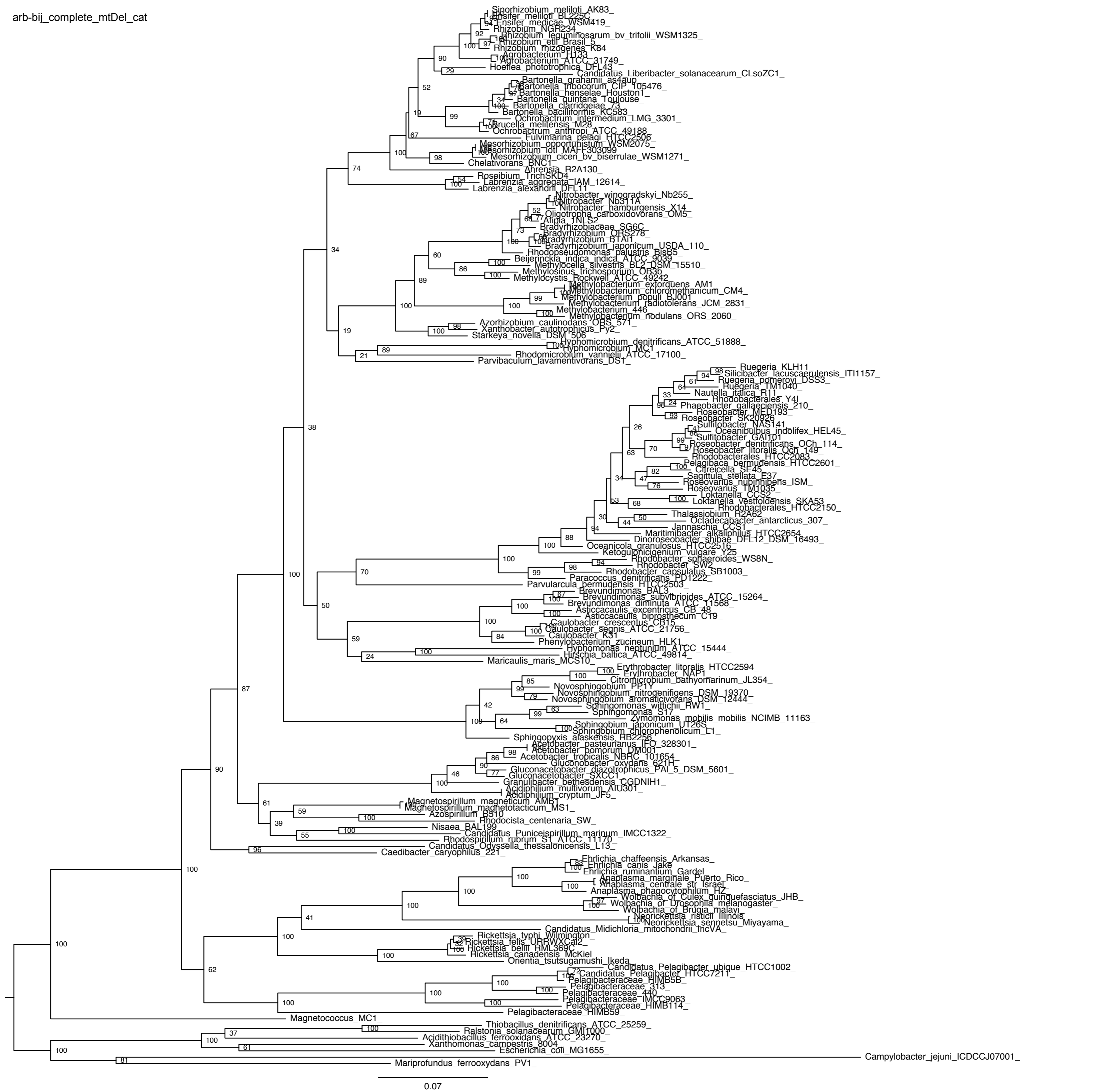

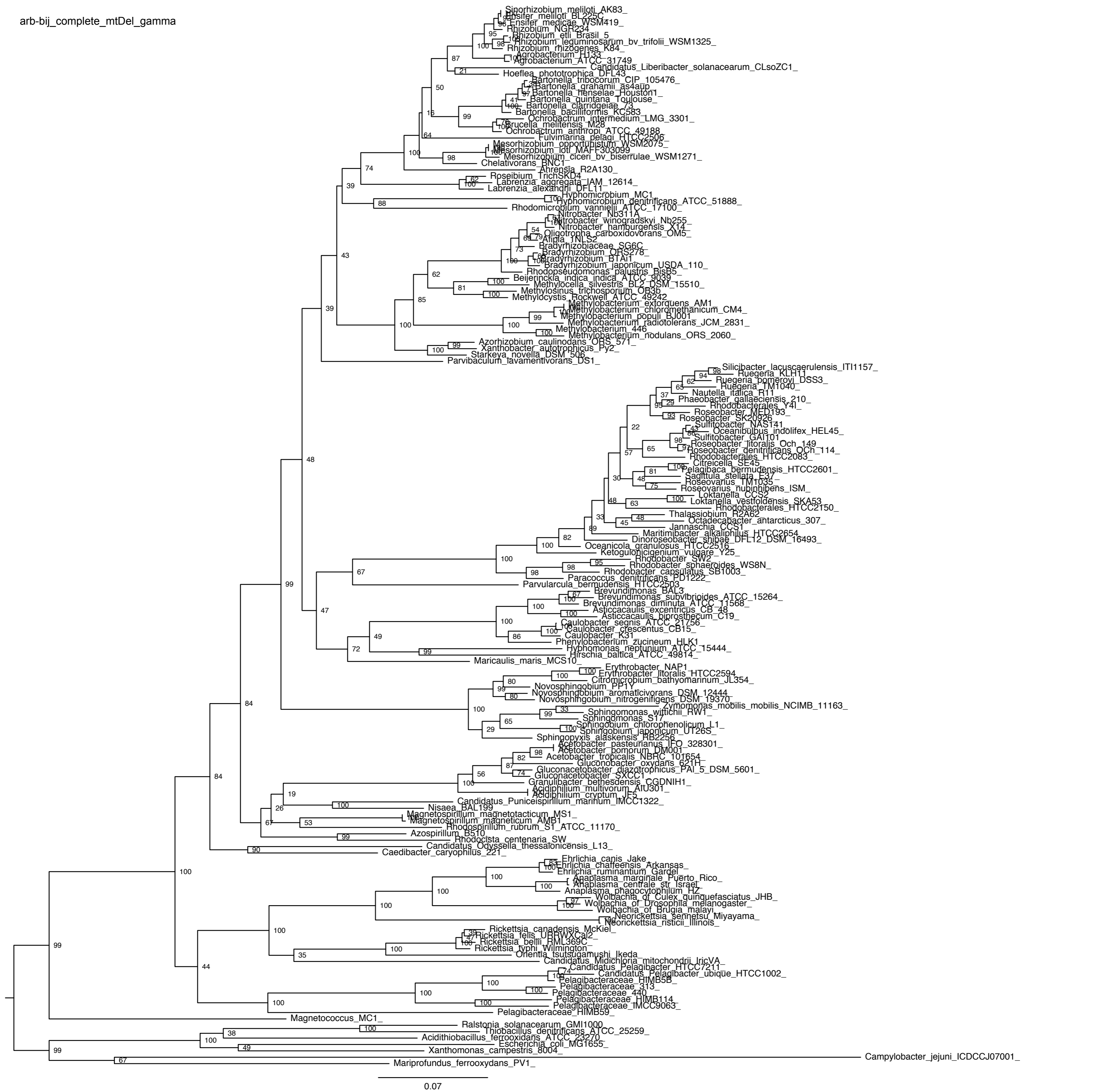

mus-bij\_complete\_mtDel\_cat

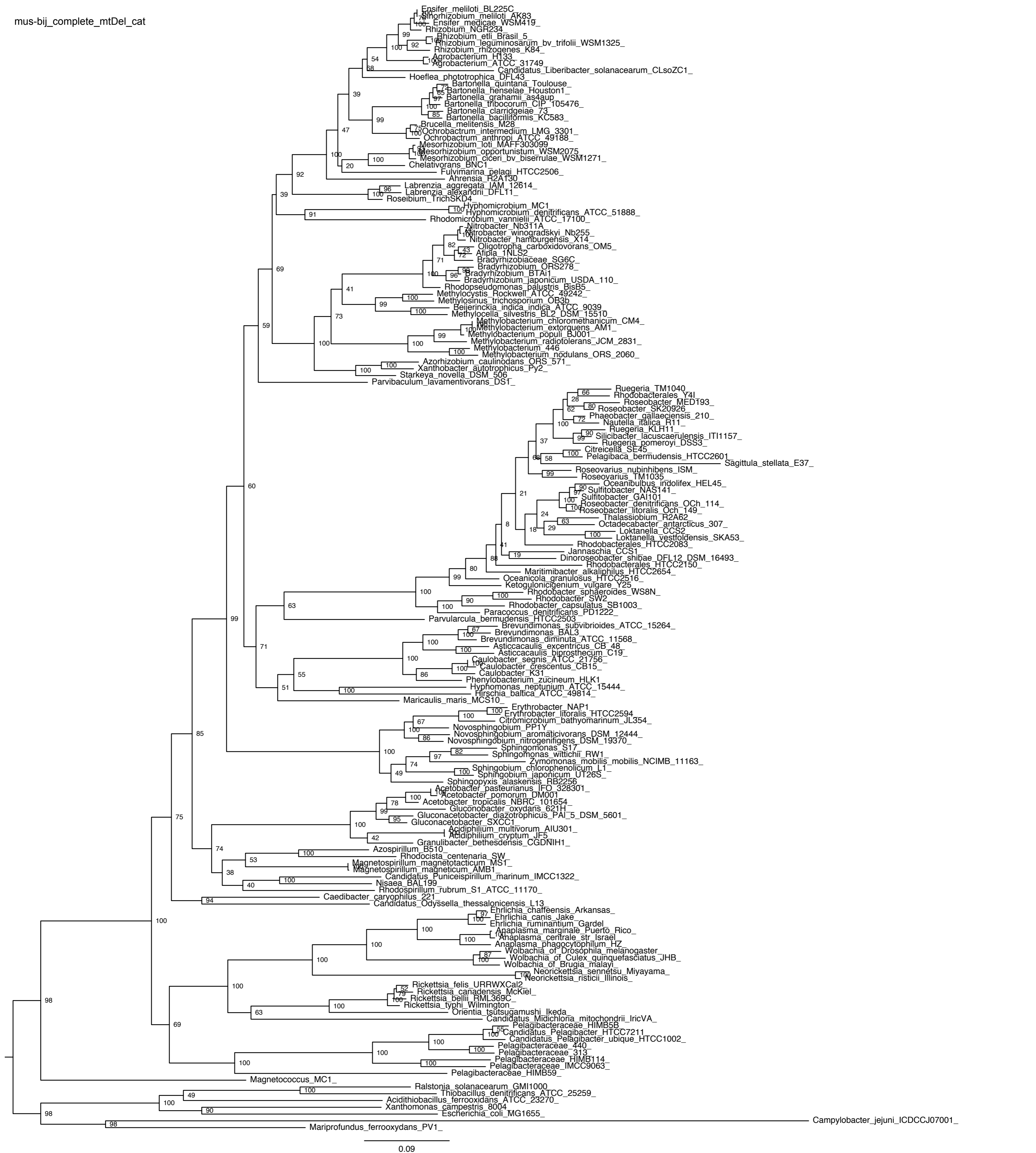

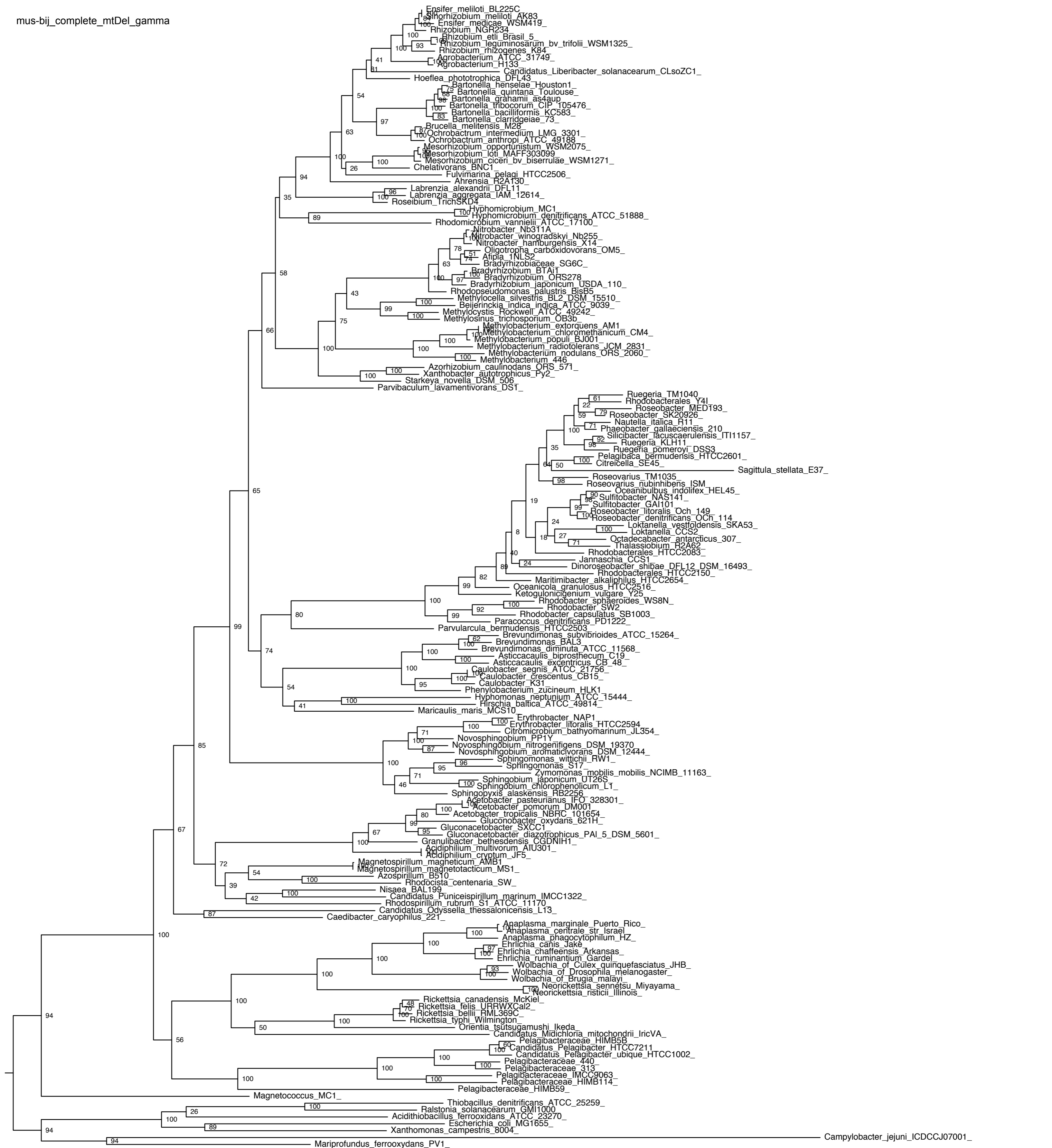

Supplement: Figure S18 — Regular-coded complete dataset mtDel trees. (PDF) [file pone.0083383.s018.pdf]

Arb\_combo\_cat

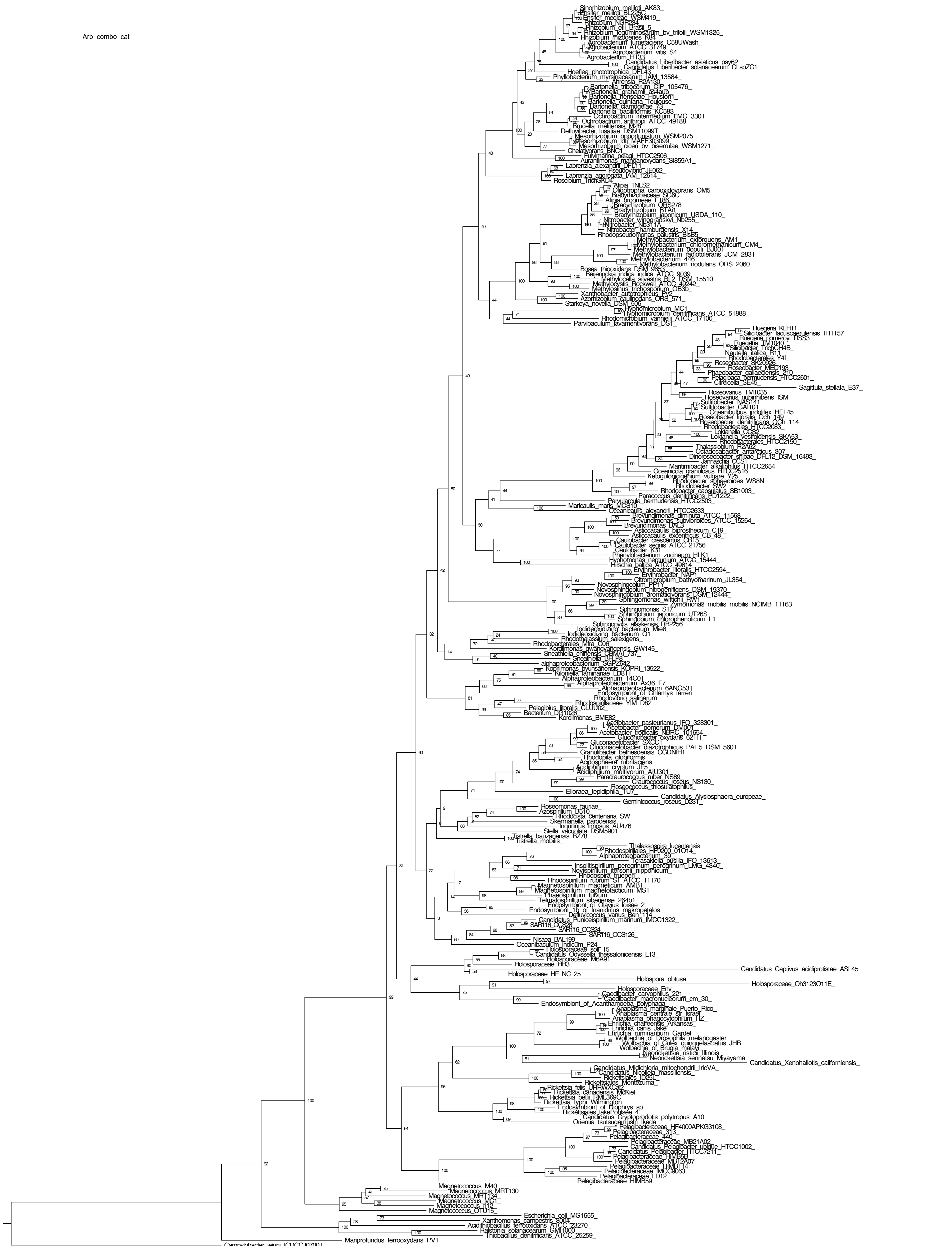

Arb\_combo\_gamma

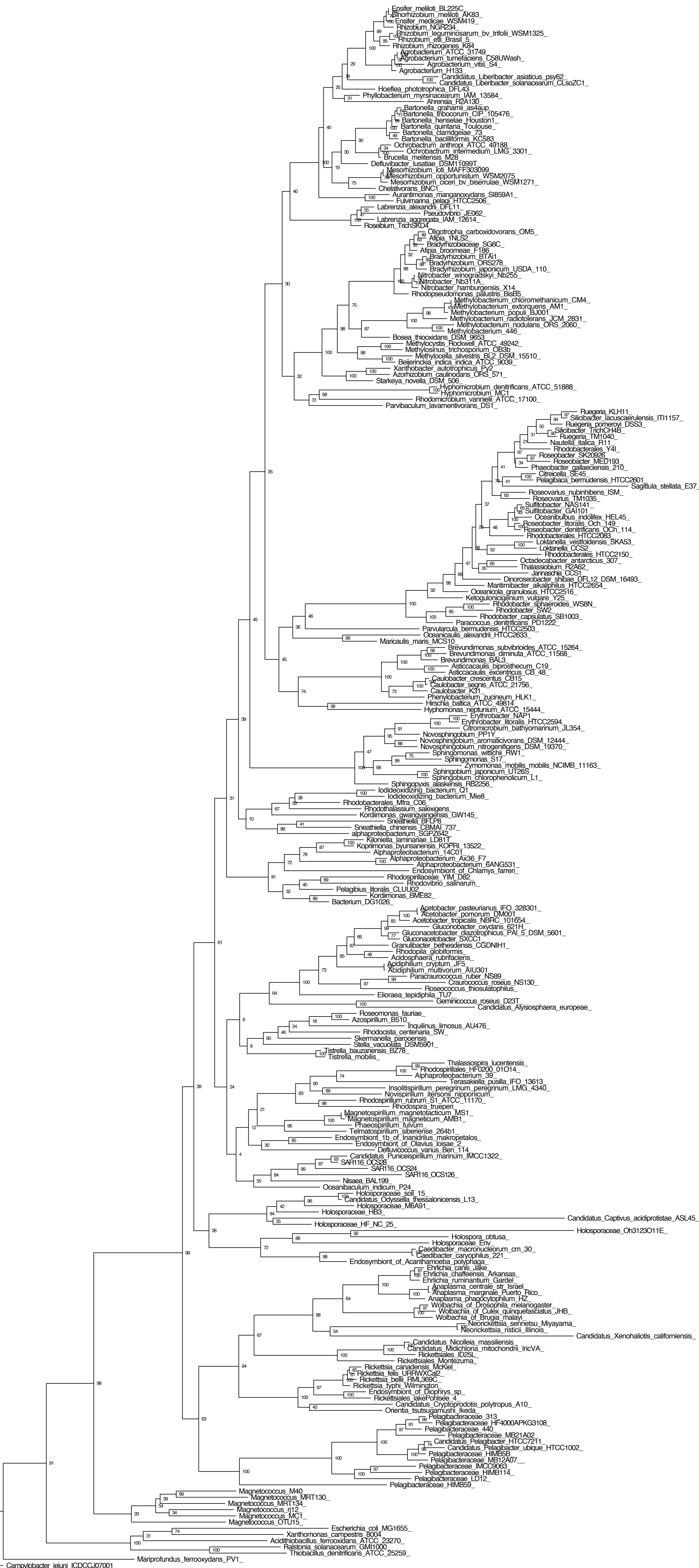

### Q.1

Mus\_combo\_cat

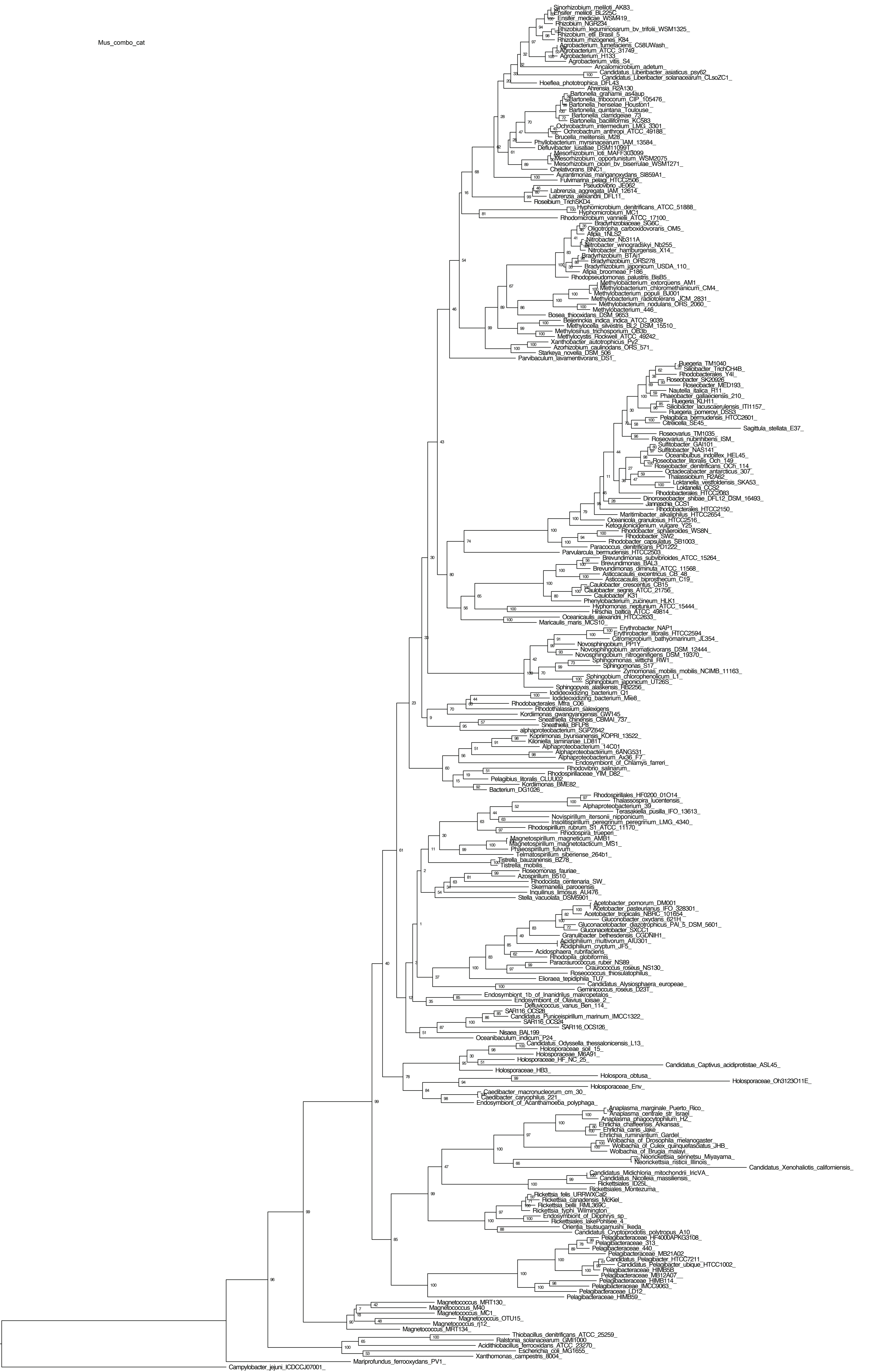

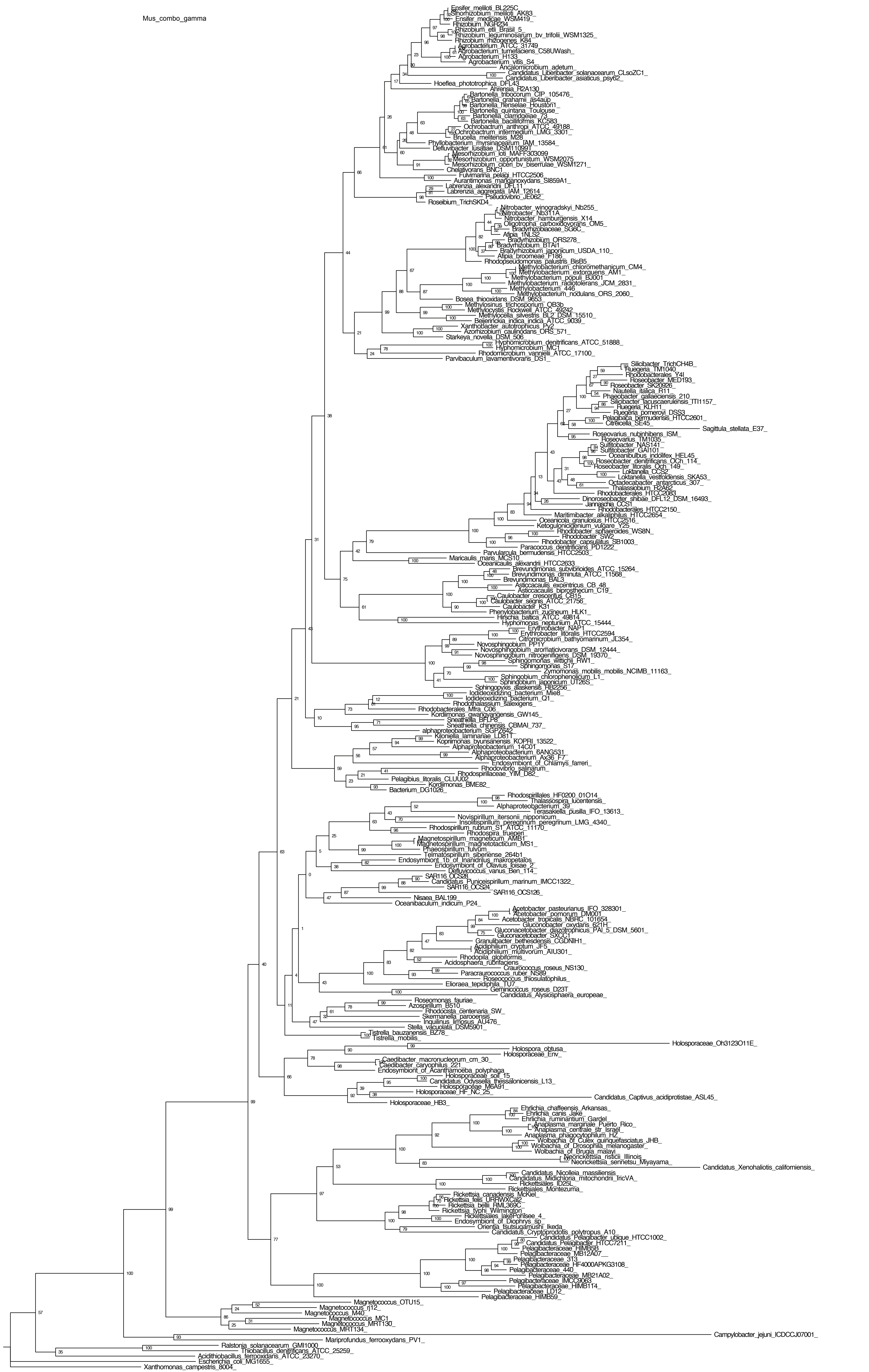

Arb\_combo\_mt\_cat

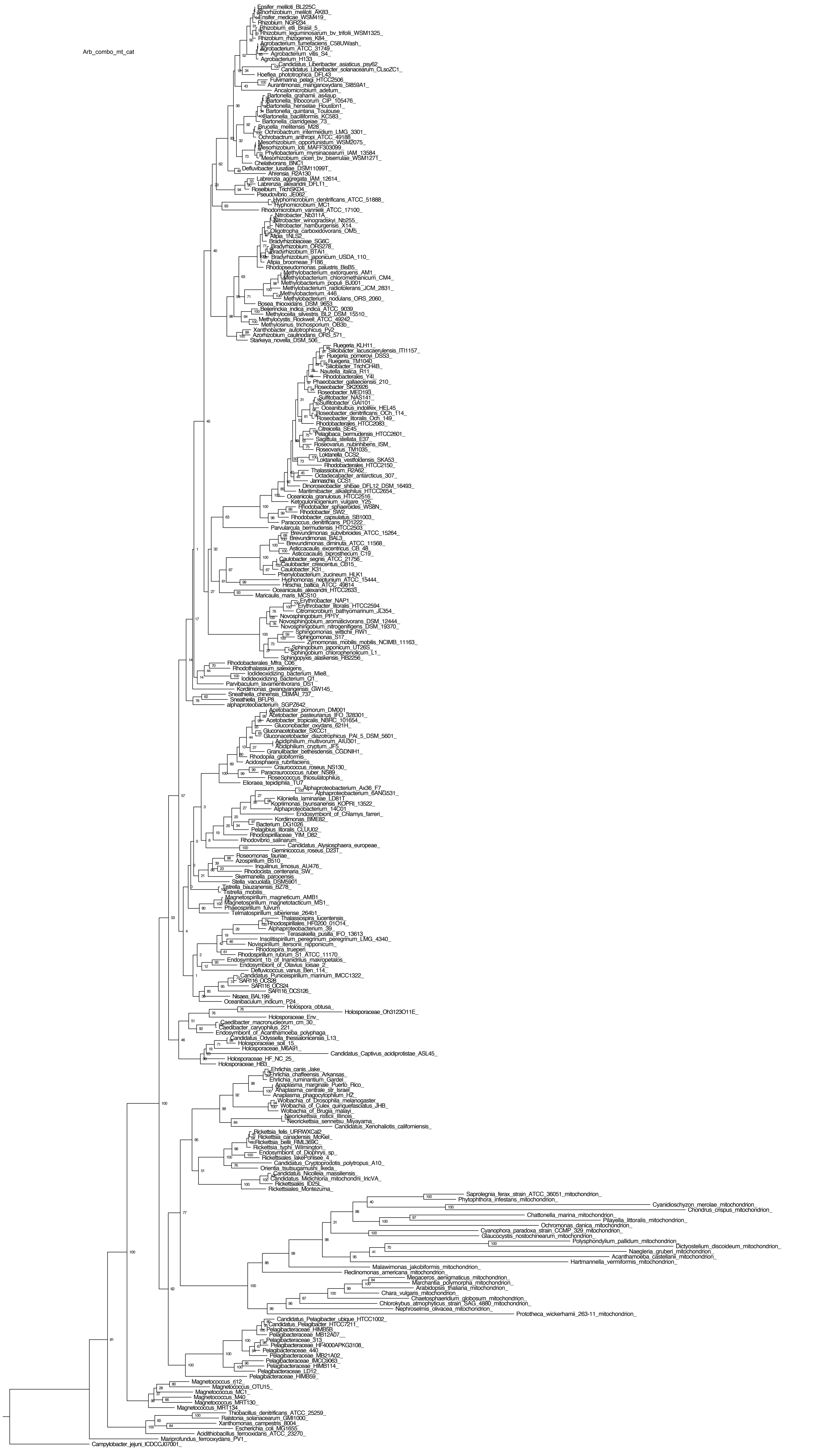

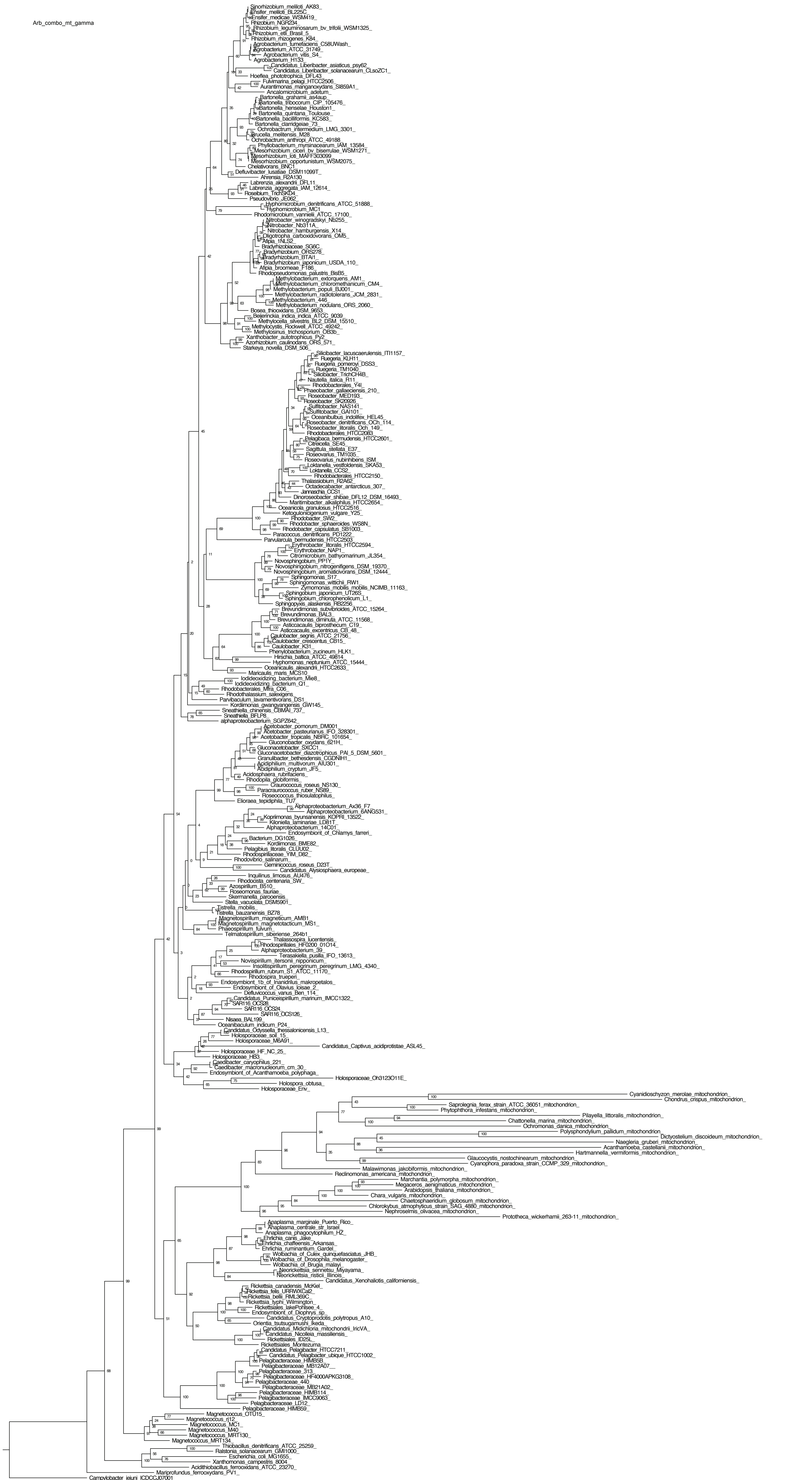

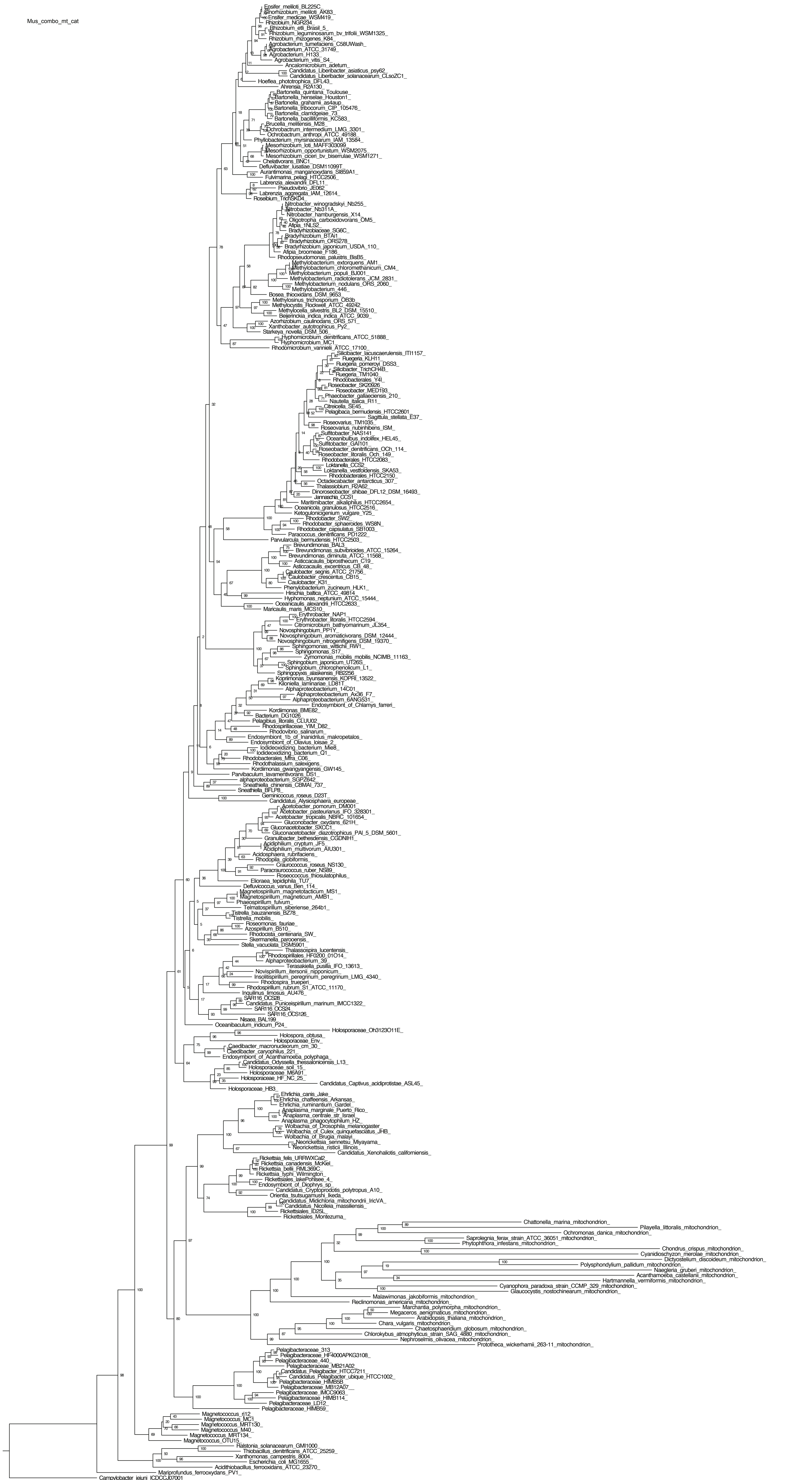

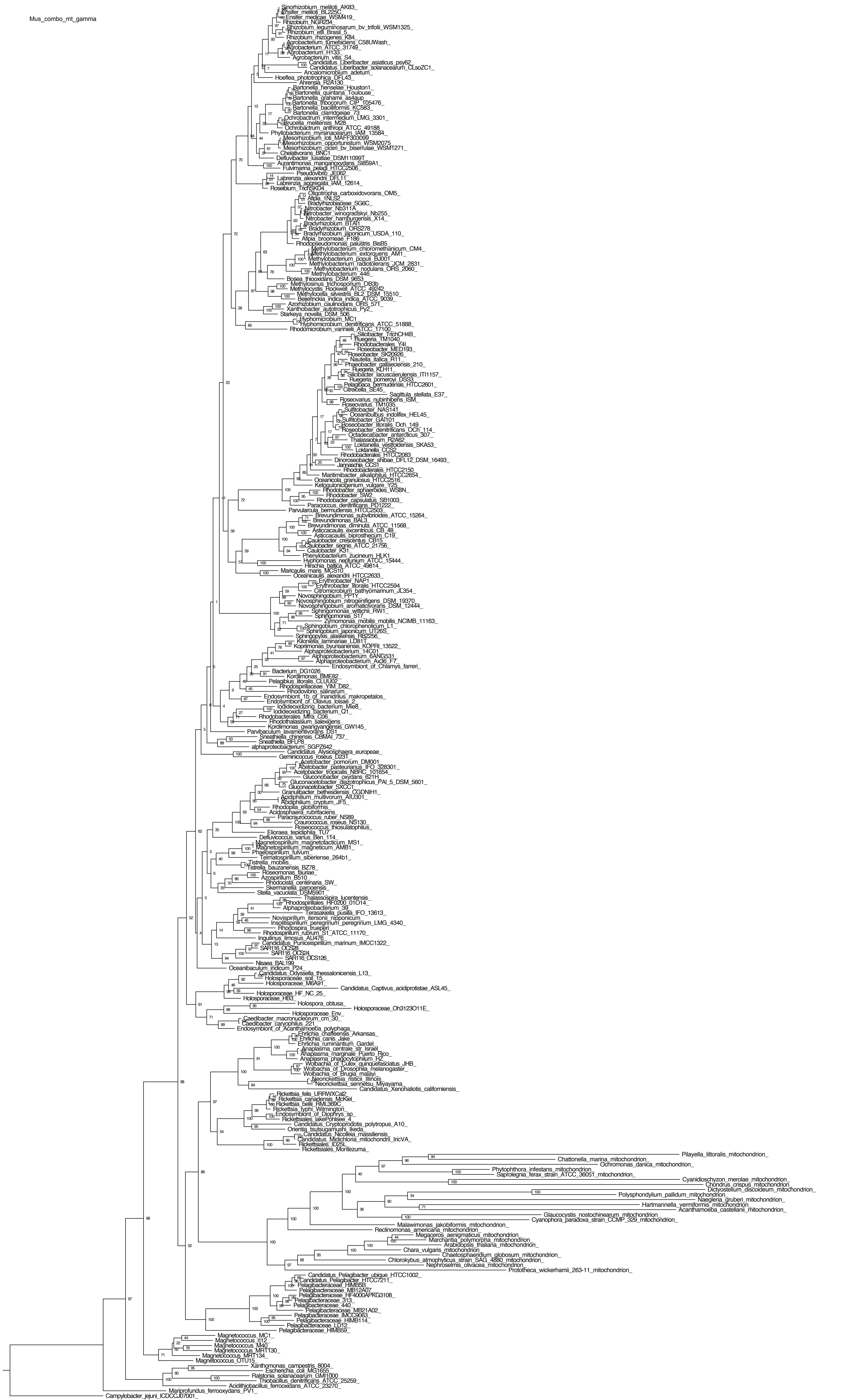

Supplement: Figure S20 — Trees including 16S only sequences, with and without mitochondria. (PDF) [file pone.0083383.s020.pdf]
