# Supplementary material for: New rRNA Gene-Based Phylogenies of the Alphaproteobacteria Provide Perspective on Major Groups, Mitochondrial Ancestry and Phylogenetic Instability
Source: PLoS One. 2013 Dec 11;8(12):e83383. doi: 10.1371/journal.pone.0083383 (PMC3859672; doi:10.1371/journal.pone.0083383)

arb-16S_complete_cat

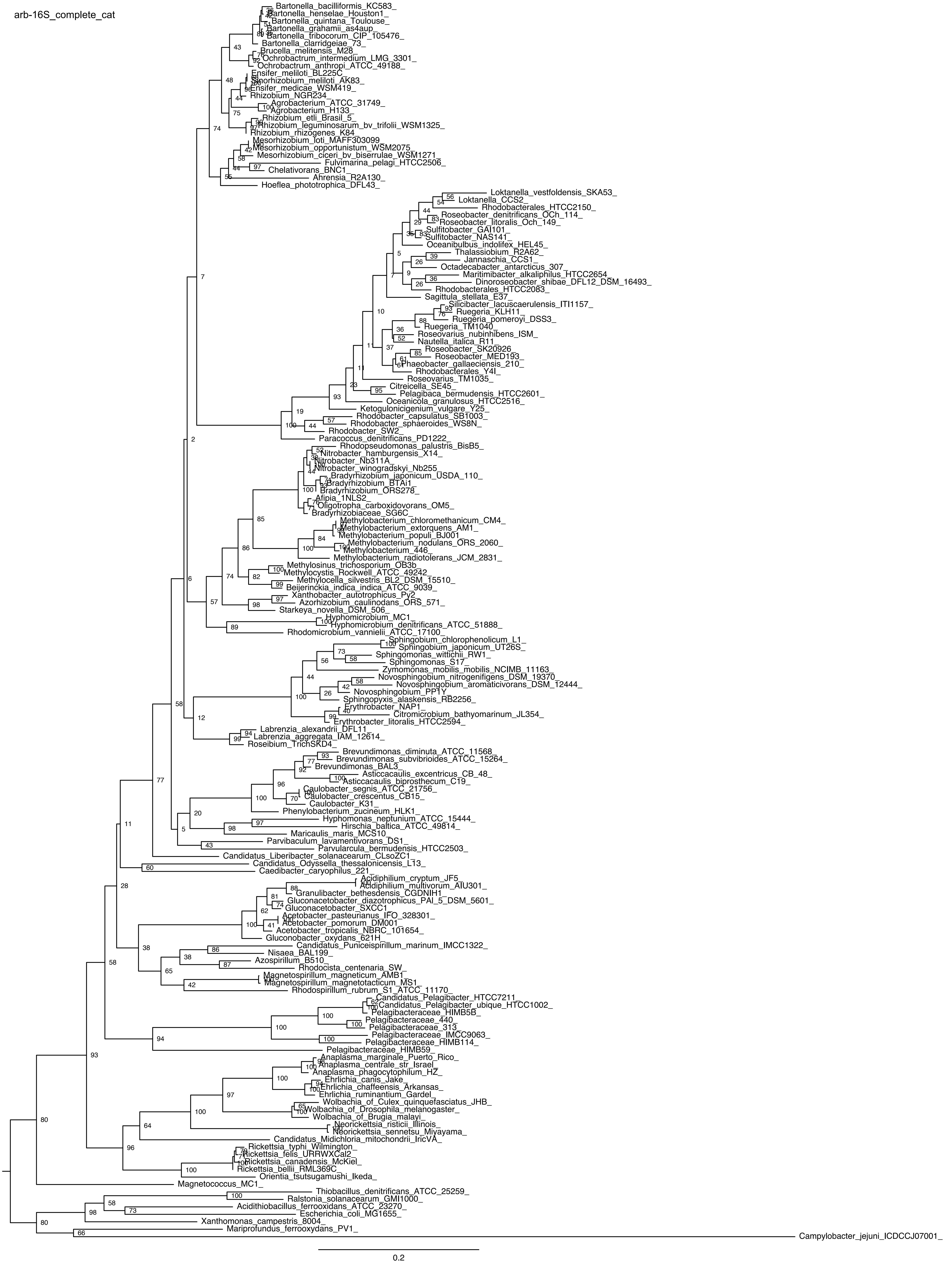

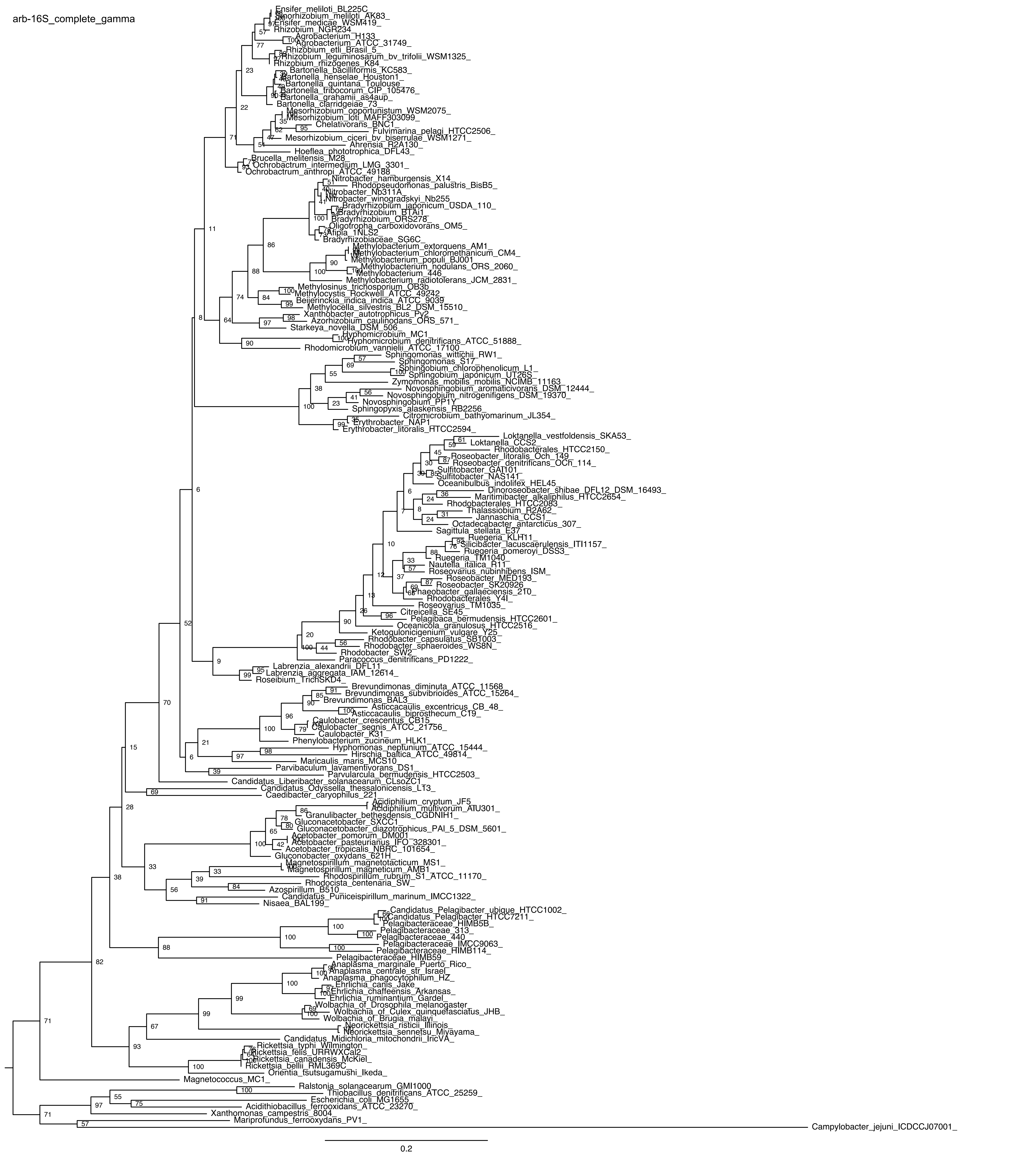

arb-16S_complete_gamma
Ensifer_meliloti_BL225C_
Sinorhizobium_meliloti_AK83_
Ensifer_medicae_WSM419_
Rhizobium_NGR234_
Agrobacterium_H133_
Agrobacterium_ATCC_31749_
Rhizobium_etli_Brasil_5_
Rhizobium_leguminosarum_bv_trifolii_WSM1325_
Rhizobium_rhizogenes_K84_
Bartonella_bacilliformis_KC583_
Bartonella_henselae_Houston1_
Bartonella_quintana_Toulouse_
Bartonella_tribocorum_CIP_105476_
Bartonella_grahamii_as4aup_
Bartonella_clarridgeiae_73_
Mesorhizobium_opportunistum_WSM2075_
Mesorhizobium_loti_MAFF303099_
Chelativorans_BNC1_
Fulvimarina_pelagi_HTCC2506_
Mesorhizobium_ciceri_bv_biserrulae_WSM1271_
Ahrensia_R2A130_
Hoeflea_phototrophica_DFL43_
Brucella_melitensis_M28_
Ochrobactrum_intermedium_LMG_3301_
Ochrobactrum_anthropi_ATCC_49188_
Nitrobacter_hamburgensis_X14_
Rhodopseudomonas_palustris_BisB5_
Nitrobacter_Nb311A_
Nitrobacter_winogradskyi_Nb255_
Bradyrhizobium_japonicum_USDA_110_
Bradyrhizobium_BTAi1_
Bradyrhizobium_ORS278_
Oligotropha_carboxidovorans_OM5_
Afipia_1NLS2_
Bradyrhizobiaceae_SG6C_
Methylobacterium_extorquens_AM1_
Methylobacterium_chloromethanicum_CM4_
Methylobacterium_populi_BJ001_
Methylobacterium_nodulans_ORS_2060_
Methylobacterium_446_
Methylobacterium_radiotolerans_JCM_2831_
Methylosinus_trichosporium_OB3b_
Methylocystis_Rockwell_ATCC_49242_
Beijerinckia_indica_indica_ATCC_9039_
Methylocella_silvestris_BL2_DSM_15510_
Xanthobacter_autotrophicus_Py2_
Azorhizobium_caulinodans_ORS_571_
Starkeya_novella_DSM_506_
Hyphomicrobium_MC1_
Hyphomicrobium_denitrificans_ATCC_51888_
Rhodomicrobium_vannielii_ATCC_17100_
Sphingomonas_wittichii_RW1_
Sphingomonas_S17_
Sphingobium_chlorophenolicum_L1_
Sphingobium_japonicum_UT26S_
Zymomonas_mobilis_mobilis_NCIMB_11163_
Novosphingobium_aromaticivorans_DSM_12444_
Novosphingobium_nitrogenifigens_DSM_19370_
Novosphingobium_PP1Y_
Sphingopyxis_alaskensis_RB2256_
Citromicrobium_bathyomarinum_JL354_
Erythrobacter_NAP1_
Erythrobacter_litoralis_HTCC2594_
Loktanella_vestfoldensis_SKA53_
Loktanella_CCS2_
Rhodobacterales_HTCC2150_
Roseobacter_litoralis_Och_149_
Roseobacter_denitrificans_OCh_114_
Sulfitobacter_GAI101_
Sulfitobacter_NAS141_
Oceanibulbus_indolifex_HEL45_
Dinoroseobacter_shibae_DFL12_DSM_16493_
Maritimibacter_alkaliphilus_HTCC2654_
Rhodobacterales_HTCC2083_
Thalassiobium_R2A62_
Jannaschia_CCS1_
Octadecabacter_antarcticus_307_
Sagittula_stellata_E37_
Ruegeria_KLH11_
Silicibacter_lacuscaerulensis_ITI1157_
Ruegeria_pomeroyi_DSS3_
Ruegeria_TM1040_
Nautella_italica_R11_
Roseovarius_nubinhibens_ISM_
Roseobacter_MED193_
Roseobacter_SK20926_
Phaeobacter_gallaeciensis_210_
Rhodobacterales_Y4I_
Roseovarius_TM1035_
Citreicella_SE45_
Pelagibaca_bermudensis_HTCC2601_
Oceanicola_granulosus_HTCC2516_
Ketogulonicigenium_vulgare_Y25_
Rhodobacter_capsulatus_SB1003_
Rhodobacter_sphaeroides_WS8N_
Rhodobacter_SW2_
Paracoccus_denitrificans_PD1222_
Labrenzia_alexandrii_DFL11_
Labrenzia_aggregata_IAM_12614_
Roseibium_TrichSKD4_
Brevundimonas_diminuta_ATCC_11568
Brevundimonas_subvibrioides_ATCC_15264_
Brevundimonas_BAL3_
Asticcacaulis_excentricus_CB_48_
Asticcacaulis_biprosthecum_C19_
Caulobacter_crescentus_CB15_
Caulobacter_segnis_ATCC_21756_
Caulobacter_K31_
Phenylobacterium_zucineum_HLK1_
Hyphomonas_neptunium_ATCC_15444_
Hirschia_baltica_ATCC_49814_
Maricaulis_maris_MCS10_
Parvibaculum_lavamentivorans_DS1_
Parvularcula_bermudensis_HTCC2503_
Candidatus_Liberibacter_solanacearum_CLsoZC1_
Candidatus_Odyssella_thessalonicensis_L13_
Caedibacter_caryophilus_221_
Acidiphilium_cryptum_JF5
Acidiphilium_multivorum_AIU301_
Granulibacter_bethesdensis_CGDNIH1_
Gluconacetobacter_SXCC1_
Gluconacetobacter_diazotrophicus_PAI_5_DSM_5601_
Acetobacter_pomorum_DM001_
Acetobacter_pasteurianus_IFO_328301_
Acetobacter_tropicalis_NBRC_101654_
Gluconobacter_oxydans_621H_
Magnetospirillum_magnetotacticum_MS1_
Magnetospirillum_magneticum_AMB1_
Rhodospirillum_rubrum_S1_ATCC_11170_
Rhodocista_centenaria_SW_
Azospirillum_B510_
Candidatus_Puniceispirillum_marinum_IMCC1322_
Nisaea_BAL199_
Candidatus_Pelagibacter_ubique_HTCC1002_
Candidatus_Pelagibacter_HTCC7211_
Pelagibacteraceae_HIMB5B_
Pelagibacteraceae_313_
Pelagibacteraceae_440_
Pelagibacteraceae_IMCC9063_
Pelagibacteraceae_HIMB114_
Pelagibacteraceae_HIMB59_
Anaplasma_marginale_Puerto_Rico_
Anaplasma_centrale_str_Israel_
Anaplasma_phagocytophilum_HZ_
Ehrlichia_canis_Jake_
Ehrlichia_chaffeensis_Arkansas_
Ehrlichia_ruminantium_Gardel_
Wolbachia_of_Drosophila_melanogaster_
Wolbachia_of_Culex_quinquefasciatus_JHB_
Wolbachia_of_Brugia_malayi_
Neorickettsia_risticii_Illinois_
Neorickettsia_sennetsu_Miyayama_
Candidatus_Midichloria_mitochondrii_IricVA_
Rickettsia_typhi_Wilmington_
Rickettsia_felis_URRWXCal2_
Rickettsia_canadensis_McKiel_
Rickettsia_bellii_RML369C_
Orientia_tsutsugamushi_Ikeda_
Magnetococcus_MC1_
Ralstonia_solanacearum_GMI1000_
Thiobacillus_denitrificans_ATCC_25259_
Escherichia_coli_MG1655_
Acidithiobacillus_ferrooxidans_ATCC_23270_
Xanthomonas_campestris_8004_
Mariprofundus_ferrooxydans_PV1_
Campylobacter_jejuni_ICDCCJ07001_
0.2

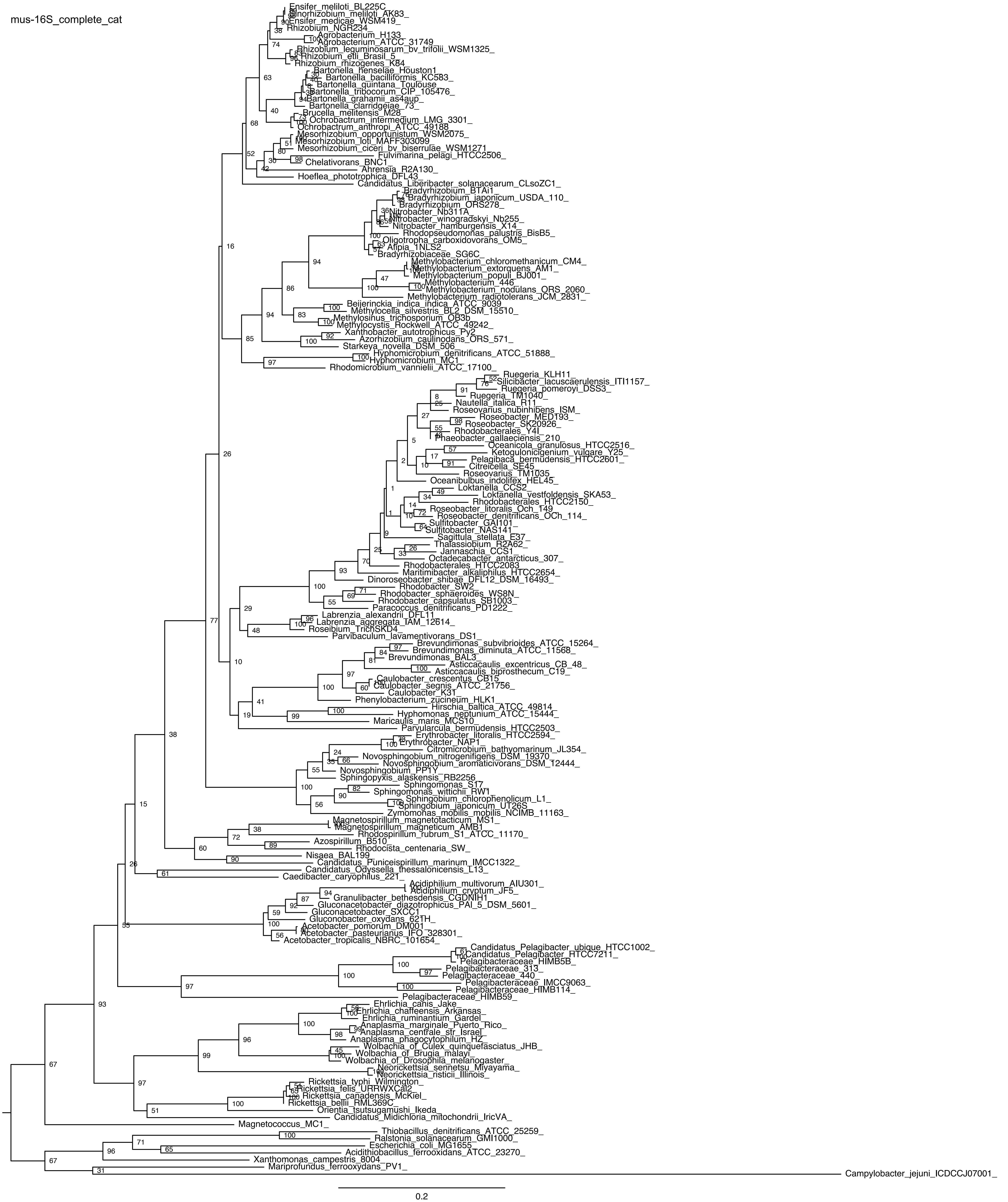
mus-16S_complete_cat
Ensifer_meliloti_BL225C
Sinorhizobium_meliloti_AK83_
Ensifer_medicae_WSM419_
Rhizobium_NGR234_
Agrobacterium_H133_
Agrobacterium_ATCC_31749
Rhizobium_leguminosarum_bv_trifolii_WSM1325_
Rhizobium_etli_Brasil_5
Rhizobium_rhizogenes_K84
Bartonella_henselae_Houston1
Bartonella_bacilliformis_KC583_
Bartonella_quintana_Toulouse
Bartonella_tribocorum_CIP_105476_
Bartonella_grahamii_as4aup_
Bartonella_clarridgeiae_73_
Brucella_melitensis_M28
Ochrobactrum_intermedium_LMG_3301_
Ochrobactrum_anthropi_ATCC_49188
Mesorhizobium_opportunistum_WSM2075_
Mesorhizobium_loti_MAFF303099_
Mesorhizobium_ciceri_bv_biserrulae_WSM1271
Fulvimarina_pelagi_HTCC2506_
Chelativorans_BNC1
Ahrensia_R2A130_
Hoeflea_phototrophica_DFL43_
Candidatus_Liberibacter_solanacearum_CLsoZC1_
Bradyrhizobium_BTAi1_
Bradyrhizobium_japonicum_USDA_110_
Bradyrhizobium_ORS278_
Nitrobacter_Nb311A
Nitrobacter_winogradskyi_Nb255_
Nitrobacter_hamburgensis_X14_
Rhodopseudomonas_palustris_BisB5_
Oligotropha_carboxidovorans_OM5_
Afipia_1NLS2_
Bradyrhizobiaceae_SG6C_
Methylobacterium_chloromethanicum_CM4_
Methylobacterium_extorquens_AM1_
Methylobacterium_populi_BJ001_
Methylobacterium_446
Methylobacterium_nodulans_ORS_2060_
Methylobacterium_radiotolerans_JCM_2831_
Beijerinckia_indica_indica_ATCC_9039
Methylocella_silvestris_BL2_DSM_15510_
Methylosinus_trichosporium_OB3b
Methylocystis_Rockwell_ATCC_49242_
Xanthobacter_autotrophicus_Py2
Azorhizobium_caulinodans_ORS_571_
Starkeya_novella_DSM_506
Hyphomicrobium_denitrificans_ATCC_51888_
Hyphomicrobium_MC1
Rhodomicrobium_vannielii_ATCC_17100_
Ruegeria_KLH11_
Silicibacter_lacuscaerulensis_ITI1157_
Ruegeria_pomeroyi_DSS3_
Ruegeria_TM1040_
Nautella_italica_R11_
Roseovarius_nubinhibens_ISM_
Roseobacter_MED193_
Roseobacter_SK20926_
Rhodobacterales_Y4I_
Phaeobacter_gallaeciensis_210_
Oceanicola_granulosus_HTCC2516_
Ketogulonicigenium_vulgare_Y25_
Pelagibaca_bermudensis_HTCC2601_
Citreicella_SE45
Roseovarius_TM1035_
Oceanibulbus_indolifex_HEL45_
Loktanella_CCS2_
Loktanella_vestfoldensis_SKA53_
Rhodobacterales_HTCC2150_
Roseobacter_litoralis_Och_149
Roseobacter_denitrificans_OCh_114_
Sulfitobacter_GAI101
Sulfitobacter_NAS141
Sagittula_stellata_E37_
Thalassiobium_R2A62_
Jannaschia_CCS1_
Octadecabacter_antarcticus_307_
Rhodobacterales_HTCC2083
Maritimibacter_alkaliphilus_HTCC2654_
Dinoroseobacter_shibae_DFL12_DSM_16493_
Rhodobacter_SW2_
Rhodobacter_sphaeroides_WS8N_
Rhodobacter_capsulatus_SB1003_
Paracoccus_denitrificans_PD1222_
Labrenzia_alexandrii_DFL11
Labrenzia_aggregata_IAM_12614_
Roseibium_TrichSKD4_
Parvibaculum_lavamentivorans_DS1_
Brevundimonas_subvibrioides_ATCC_15264_
Brevundimonas_diminuta_ATCC_11568_
Brevundimonas_BAL3_
Asticcacaulis_excentricus_CB_48_
Asticcacaulis_biprosthecum_C19_
Caulobacter_crescentus_CB15
Caulobacter_segnis_ATCC_21756_
Caulobacter_K31_
Phenylobacterium_zucineum_HLK1_
Hirschia_baltica_ATCC_49814
Hyphomonas_neptunium_ATCC_15444_
Maricaulis_maris_MCS10_
Parvularcula_bermudensis_HTCC2503_
Erythrobacter_litoralis_HTCC2594_
Erythrobacter_NAP1_
Citromicrobium_bathyomarinum_JL354_
Novosphingobium_nitrogenifigens_DSM_19370_
Novosphingobium_aromaticivorans_DSM_12444_
Novosphingobium_PP1Y
Sphingopyxis_alaskensis_RB2256
Sphingomonas_S17
Sphingomonas_wittichii_RW1
Sphingobium_chlorophenolicum_L1_
Sphingobium_japonicum_UT26S
Zymomonas_mobilis_mobilis_NCIMB_11163_
Magnetospirillum_magnetotacticum_MS1_
Magnetospirillum_magneticum_AMB1
Rhodospirillum_rubrum_S1_ATCC_11170_
Azospirillum_B510_
Rhodocista_centenaria_SW_
Nisaea_BAL199
Candidatus_Puniceispirillum_marinum_IMCC1322_
Candidatus_Odyssella_thessalonicensis_L13_
Caedibacter_caryophilus_221_
Acidiphilium_multivorum_AIU301_
Acidiphilium_cryptum_JF5_
Granulibacter_bethesdensis_CGDNIH1
Gluconacetobacter_diazotrophicus_PAl_5_DSM_5601_
Gluconacetobacter_SXCC1
Gluconobacter_oxydans_621H_
Acetobacter_pomorum_DM001
Acetobacter_pasteurianus_IFO_328301_
Acetobacter_tropicalis_NBRC_101654_
Candidatus_Pelagibacter_ubique_HTCC1002_
Candidatus_Pelagibacter_HTCC7211_
Pelagibacteraceae_HIMB5B_
Pelagibacteraceae_313_
Pelagibacteraceae_440_
Pelagibacteraceae_IMCC9063_
Pelagibacteraceae_HIMB114_
Pelagibacteraceae_HIMB59_
Ehrlichia_canis_Jake_
Ehrlichia_chaffeensis_Arkansas_
Ehrlichia_ruminantium_Gardel_
Anaplasma_marginale_Puerto_Rico_
Anaplasma_centrale_str_Israel_
Anaplasma_phagocytophilum_HZ_
Wolbachia_of_Culex_quinquefasciatus_JHB_
Wolbachia_of_Brugia_malayi_
Wolbachia_of_Drosophila_melanogaster_
Neorickettsia_sennetsu_Miyayama_
Neorickettsia_risticii_Illinois_
Rickettsia_typhi_Wilmington_
Rickettsia_felis_URRWXCal2_
Rickettsia_canadensis_McKiel_
Rickettsia_bellii_RML369C_
Orientia_tsutsugamushi_Ikeda_
Candidatus_Midichloria_mitochondrii_IricVA_
Magnetococcus_MC1_
Thiobacillus_denitrificans_ATCC_25259_
Ralstonia_solanacearum_GMI1000_
Escherichia_coli_MG1655_
Acidithiobacillus_ferrooxidans_ATCC_23270_
Xanthomonas_campestris_8004_
Mariprofundus_ferrooxydans_PV1_
Campylobacter_jejuni_ICDCCJ07001_
0.2

mus-16S_complete_gamma

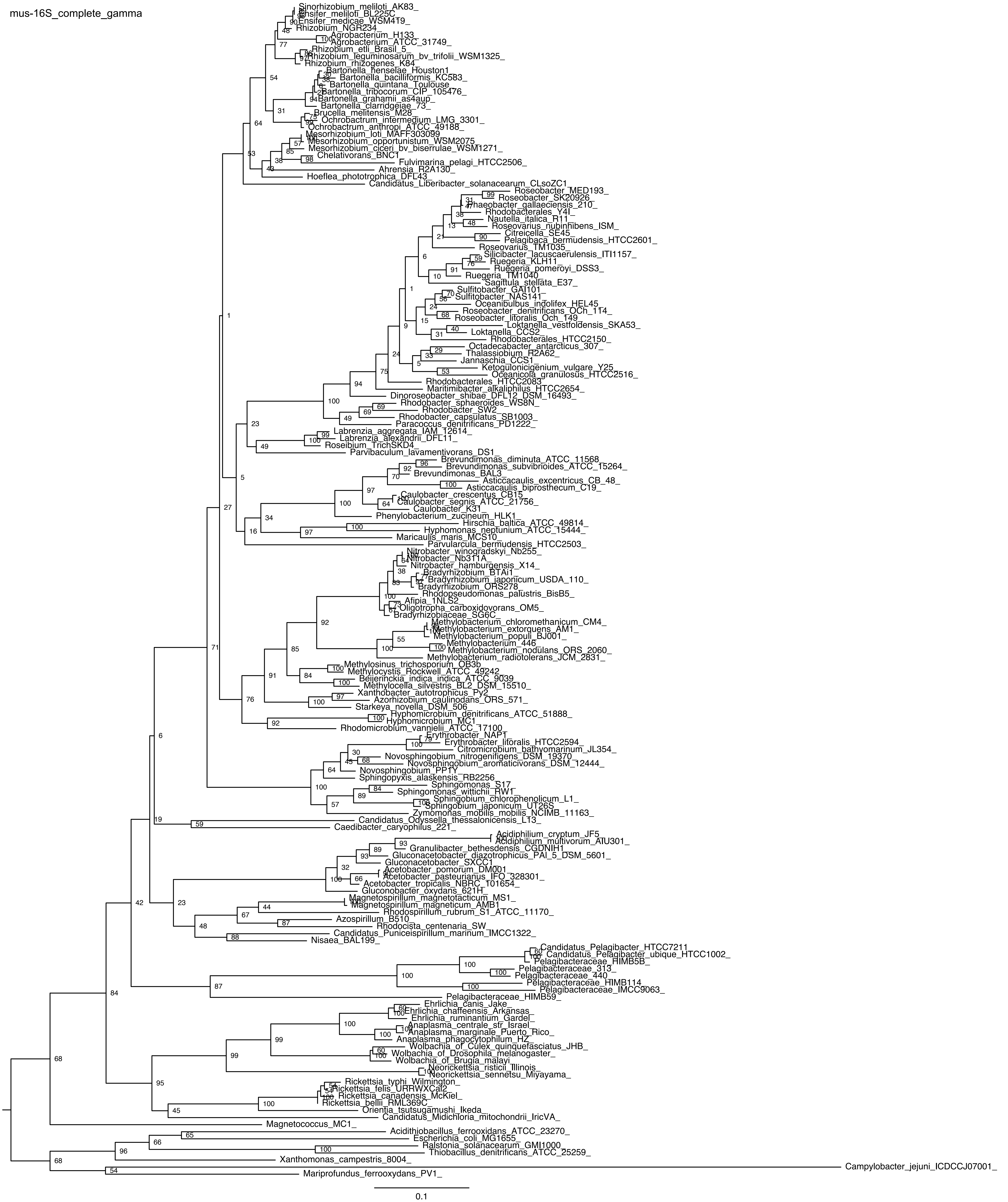

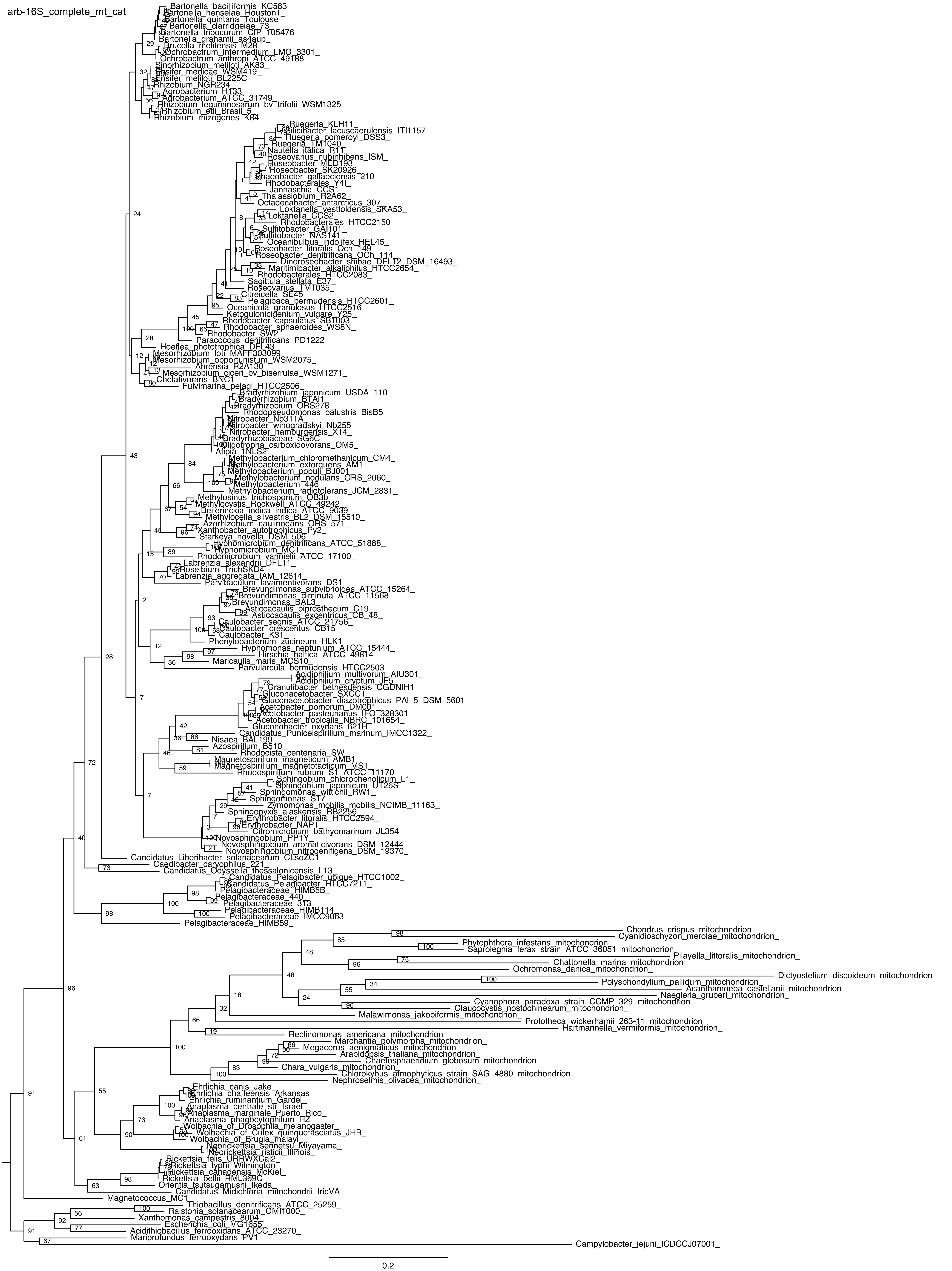

arb-16S_complete_mt_cat
Bartonella_bacilliformis_KC583_
Bartonella_henselae_Houston1_
Bartonella_quintana_Toulouse_
Bartonella_clarridgeiae_73_
Bartonella_tribocorum_CIP_105476_
Bartonella_grahamii_as4aup_
Brucella_melitensis_M28_
Ochrobactrum_intermedium_LMG_3301_
Ochrobactrum_anthropi_ATCC_49188_
Sinorhizobium_meliloti_AK83_
Ensifer_medicae_WSM419_
Ensifer_meliloti_BL225C_
Rhizobium_NGR234_
Agrobacterium_H13_
Agrobacterium_ATCC_31749_
Rhizobium_leguminosarum_bv_trifolii_WSM1325_
Rhizobium_etli_Brasil_5_
Rhizobium_rhizogenes_K84_
Ruegeria_KLH11_
Silicibacter_lacuscaerulensis_ITI1157_
Ruegeria_pomeroyi_DSS3_
Ruegeria_TM1040_
Nautella_italica_R11_
Roseovarius_nubinhibens_ISM_
Roseobacter_MED193_
Roseobacter_SK20926_
Phaeobacter_gallaeciensis_210_
Rhodobacterales_Y4I_
Jannaschia_CCS1_
Thalassiobium_R2A62_
Octadecabacter_antarcticus_307_
Loktanella_vestfoldensis_SKA53_
Loktanella_CCS2_
Rhodobacterales_HTCC2150_
Sulfitobacter_GAI101_
Sulfitobacter_NAS141_
Oceanibulbus_indolifex_HEL45_
Roseobacter_litoralis_Och_149_
Roseobacter_denitrificans_OCh_114_
Dinoroseobacter_shibae_DFL12_DSM_16493_
Maritimibacter_alkaliphilus_HTCC2654_
Rhodobacterales_HTCC2083_
Sagittula_stellata_E37_
Roseovarius_TM1035_
Citreicella_SE45_
Pelagibaca_bermudensis_HTCC2601_
Oceanicola_granulosus_HTCC2516_
Ketogulonicigenium_vulgare_Y25_
Rhodobacter_capsulatus_SB1003_
Rhodobacter_sphaeroides_WS8N_
Rhodobacter_SW2_
Paracoccus_denitrificans_PD1222_
Hoeflea_phototrophica_DFL43_
Mesorhizobium_loti_MAFF303099_
Mesorhizobium_opportunistum_WSM2075_
Ahrensia_R2A130_
Mesorhizobium_ciceri_bv_biserrulae_WSM1271_
Chelativorans_BNC1_
Fulvimarina_pelagi_HTCC2506_
Bradyrhizobium_japonicum_USDA_110_
Bradyrhizobium_BTAi1_
Bradyrhizobium_ORS278_
Rhodopseudomonas_palustris_BisB5_
Nitrobacter_Nb311A_
Nitrobacter_winogradskyi_Nb255_
Nitrobacter_hamburgensis_X14_
Bradyrhizobiaceae_SG6C_
Oligotropha_carboxidovorans_OM5_
Afipia_1NLS2_
Methylobacterium_chloromethanicum_CM4_
Methylobacterium_extorquens_AM1_
Methylobacterium_populi_BJ001_
Methylobacterium_nodulans_ORS_2060_
Methylobacterium_446_
Methylobacterium_radiotolerans_JCM_2831_
Methylosinus_trichosporium_OB3b_
Methylocystis_Rockwell_ATCC_49242_
Beijerinckia_indica_indica_ATCC_9039_
Methylocella_silvestris_BL2_DSM_15510_
Azorhizobium_caulinodans_ORS_571_
Xanthobacter_autotrophicus_Py2_
Starkeya_novella_DSM_506_
Hyphomicrobium_denitrificans_ATCC_51888_
Hyphomicrobium_MC1_
Rhodomicrobium_vannielii_ATCC_17100_
Labrenzia_alexandrii_DFL11_
Roseibium_TrichSKD4_
Labrenzia_aggregata_IAM_12614_
Parvibaculum_lavamentivorans_DS1_
Brevundimonas_subvibrioides_ATCC_15264_
Brevundimonas_diminuta_ATCC_11568_
Brevundimonas_BAL3_
Asticcacaulis_biprosthecum_C19_
Asticcacaulis_excentricus_CB_48_
Caulobacter_segnis_ATCC_21756_
Caulobacter_crescentus_CB15_
Caulobacter_K31_
Phenylobacterium_zucineum_HLK1_
Hyphomonas_neptunium_ATCC_15444_
Hirschia_baltica_ATCC_49814_
Maricaulis_maris_MCS10_
Parvularcula_bermudensis_HTCC2503_
Acidiphilium_multivorum_AIU301_
Acidiphilium_cryptum_JF5_
Granulibacter_bethesdensis_CGDNIH1_
Gluconacetobacter_SXCC1_
Gluconacetobacter_diazotrophicus_PAl_5_DSM_5601_
Acetobacter_pomorum_DM001_
Acetobacter_pasteurianus_IFO_328301_
Acetobacter_tropicalis_NBRC_101654_
Gluconobacter_oxydans_621H_
Candidatus_Puniceispirillum_marinum_IMCC1322_
Nisaea_BAL199_
Azospirillum_B510_
Rhodocista_centenaria_SW_
Magnetospirillum_magneticum_AMB1_
Magnetospirillum_magnetotacticum_MS1_
Rhodospirillum_rubrum_S1_ATCC_11170_
Sphingobium_chlorophenolicum_L1_
Sphingobium_japonicum_UT26S_
Sphingomonas_wittichii_RW1_
Sphingomonas_S17_
Zymomonas_mobilis_mobilis_NCIMB_11163_
Sphingopyxis_alaskensis_RB2256_
Erythrobacter_litoralis_HTCC2594_
Erythrobacter_NAP1_
Citromicrobium_bathyomarinum_JL354_
Novosphingobium_PP1Y_
Novosphingobium_aromaticivorans_DSM_12444_
Novosphingobium_nitrogenifigens_DSM_19370_
Candidatus_Liberibacter_solanacearum_CLsoZC1_
Caedibacter_caryophilus_221_
Candidatus_Odyssella_thessalonicensis_L13_
Candidatus_Pelagibacter_ubique_HTCC1002_
Candidatus_Pelagibacter_HTCC7211_
Pelagibacteraceae_HIMB5B_
Pelagibacteraceae_440_
Pelagibacteraceae_313_
Pelagibacteraceae_HIMB114_
Pelagibacteraceae_IMCC9063_
Pelagibacteraceae_HIMB59_
Chondrus_crispus_mitochondrion_
Cyanidioschyzon_merolae_mitochondrion_
Phytophthora_infestans_mitochondrion_
Saprolegnia_ferax_strain_ATCC_36051_mitochondrion_
Pilayella_littoralis_mitochondrion_
Chattonella_marina_mitochondrion_
Ochromonas_danica_mitochondrion_
Dictyostelium_discoideum_mitochondrion_
Polysphondylium_pallidum_mitochondrion_
Acanthamoeba_castellanii_mitochondrion_
Naegleria_gruberi_mitochondrion_
Cyanophora_paradoxa_strain_CCMP_329_mitochondrion_
Glaucocystis_nostochinearum_mitochondrion_
Malawimonas_jakobiformis_mitochondrion_
Prototheca_wickerhamii_263-11_mitochondrion_
Hartmannella_vermiformis_mitochondrion_
Reclinomonas_americana_mitochondrion_
Marchantia_polymorpha_mitochondrion_
Megaceros_aenigmaticus_mitochondrion_
Arabidopsis_thaliana_mitochondrion_
Chaetosphaeridium_globosum_mitochondrion_
Chara_vulgaris_mitochondrion_
Chlorokybus_atmophyticus_strain_SAG_4880_mitochondrion_
Nephroselmis_olivacea_mitochondrion_
Ehrlichia_canis_Jake_
Ehrlichia_chaffeensis_Arkansas_
Ehrlichia_ruminantium_Gardel_
Anaplasma_centrale_str_Israel_
Anaplasma_marginale_Puerto_Rico_
Anaplasma_phagocytophilum_HZ_
Wolbachia_of_Drosophila_melanogaster_
Wolbachia_of_Culex_quinquefasciatus_JHB_
Wolbachia_of_Brugia_malayi_
Neorickettsia_sennetsu_Miyayama_
Neorickettsia_risticii_Illinois_
Rickettsia_felis_URRWXCal2_
Rickettsia_typhi_Wilmington_
Rickettsia_canadensis_McKiel_
Rickettsia_bellii_RML369C_
Orientia_tsutsugamushi_Ikeda_
Candidatus_Midichloria_mitochondrii_IricVA_
Magnetococcus_MC1_
Thiobacillus_denitrificans_ATCC_25259_
Ralstonia_solanacearum_GMI1000_
Xanthomonas_campestris_8004_
Escherichia_coli_MG1655_
Acidithiobacillus_ferrooxidans_ATCC_23270_
Mariprofundus_ferrooxydans_PV1_
Campylobacter_jejuni_ICDCCJ07001_
0.2

arb-16S_complete_mt_gamma

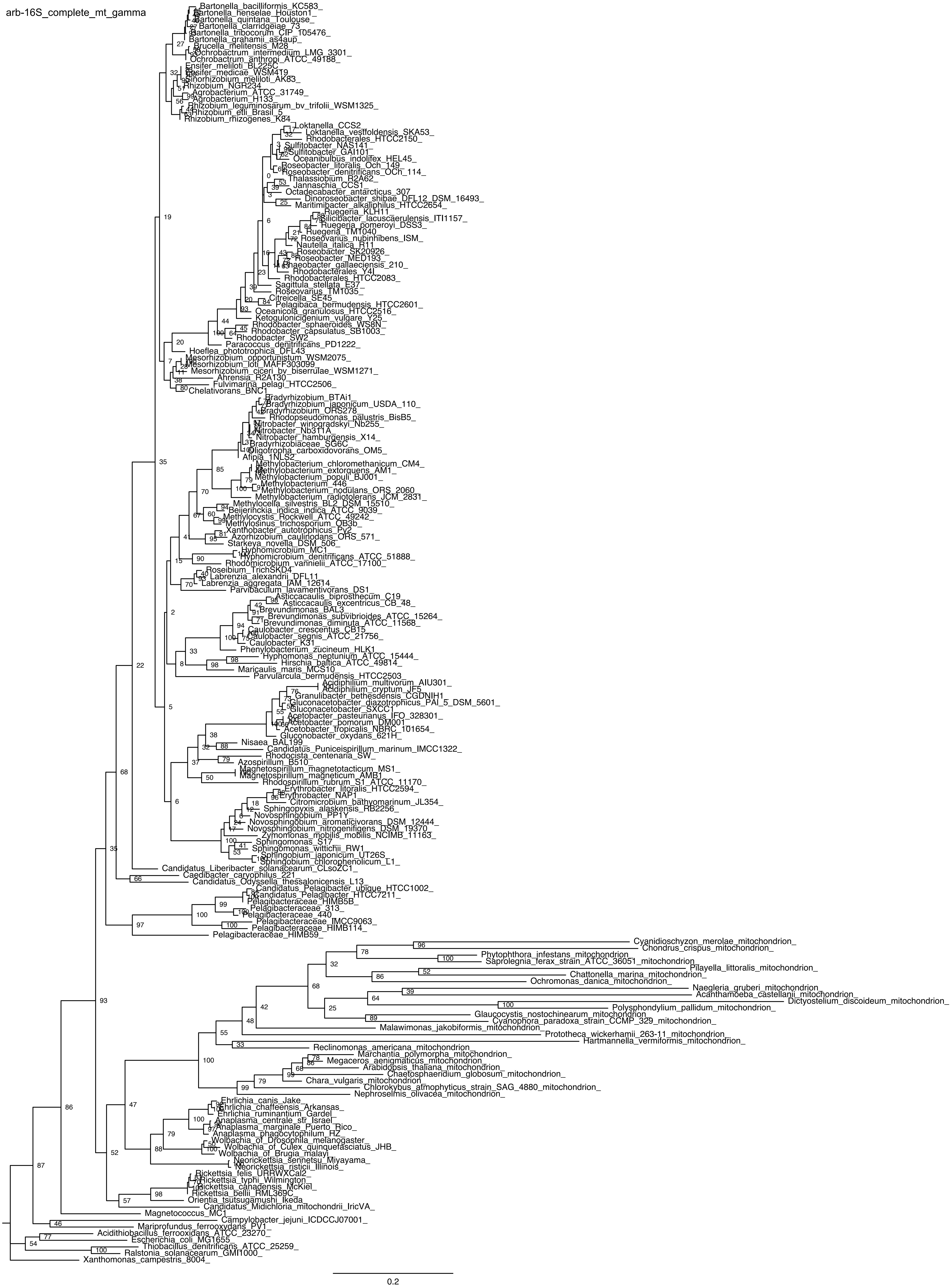

mus-16S_complete_mt_cat

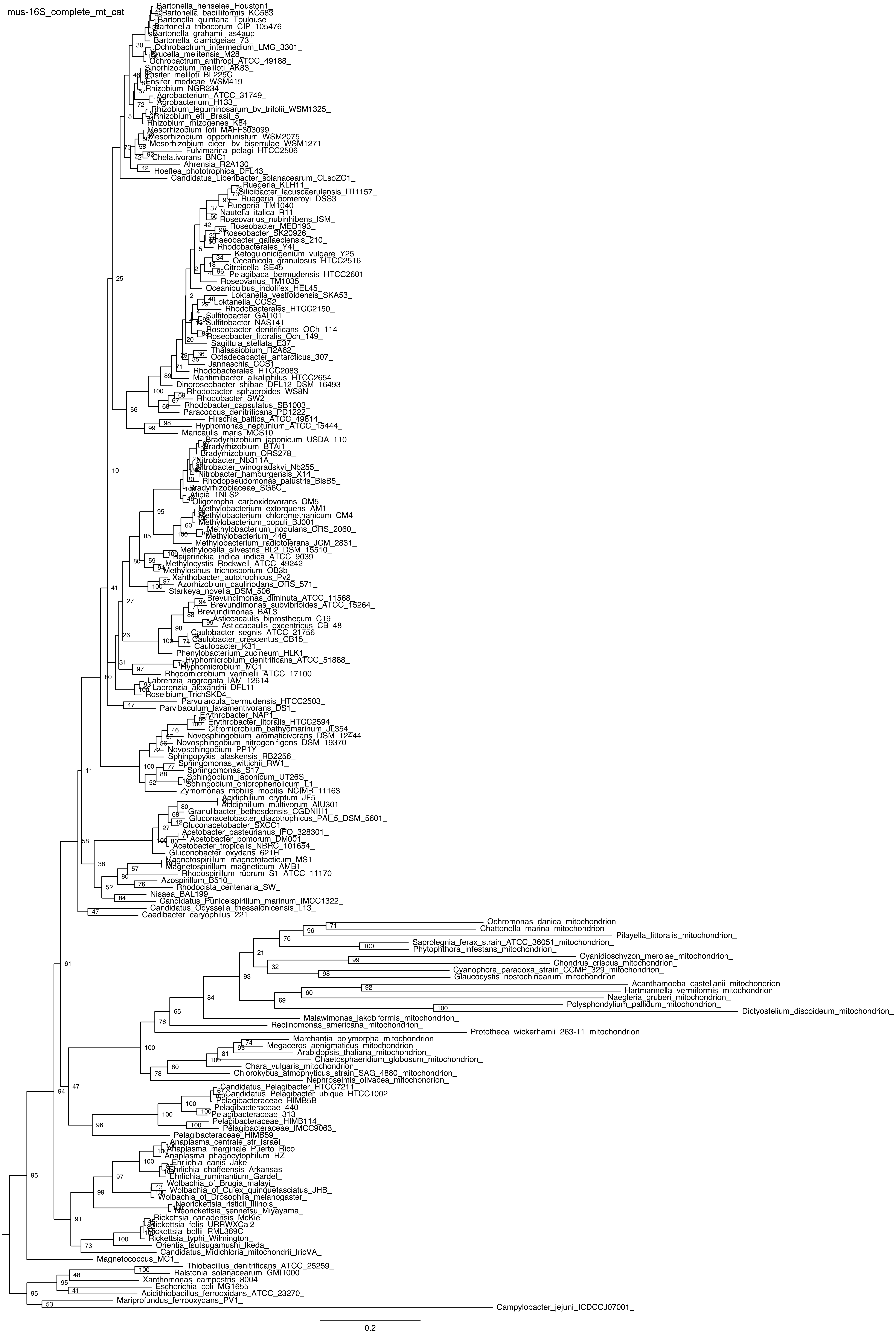

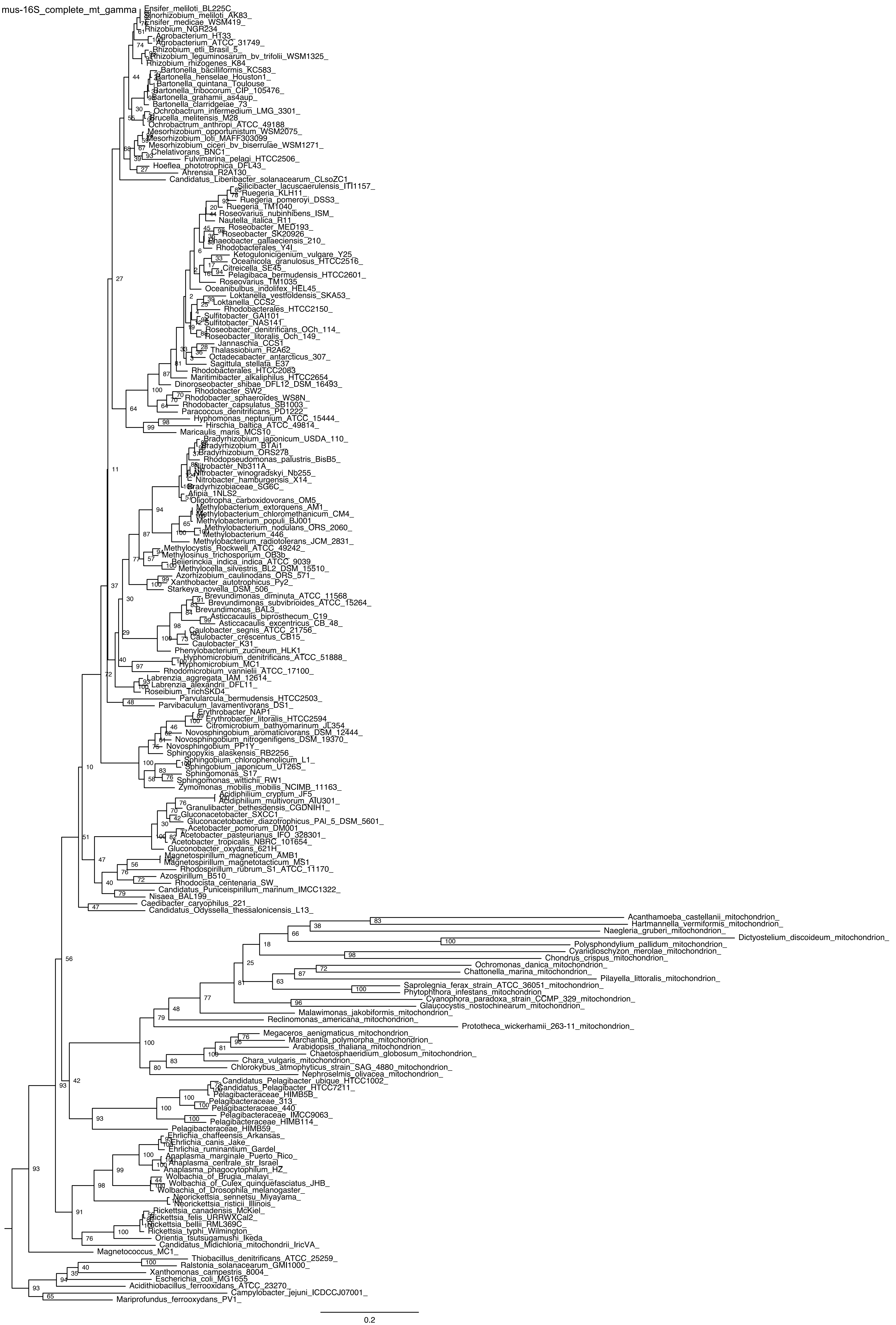
mus-16S_complete_mt_gamma
Ensifer_meliloti_BL225C_
Sinorhizobium_meliloti_AK83_
Ensifer_medicae_WSM419_
Rhizobium_NGR234_
Agrobacterium_H133_
Agrobacterium_ATCC_31749_
Rhizobium_etli_Brasil_5_
Rhizobium_leguminosarum_bv_trifolii_WSM1325_
Rhizobium_rhizogenes_K84_
Bartonella_bacilliformis_KC583_
Bartonella_henselae_Houston1_
Bartonella_quintana_Toulouse_
Bartonella_tribocorum_CIP_105476_
Bartonella_grahamii_as4aup_
Bartonella_clarridgeiae_73_
Ochrobactrum_intermedium_LMG_3301_
Brucella_melitensis_M28_
Ochrobactrum_anthropi_ATCC_49188_
Mesorhizobium_opportunistum_WSM2075_
Mesorhizobium_loti_MAFF303099_
Mesorhizobium_ciceri_bv_biserrulae_WSM1271_
Chelativorans_BNC1_
Fulvimarina_pelagi_HTCC2506_
Hoeflea_phototrophica_DFL43_
Ahrensia_R2A130_
Candidatus_Liberibacter_solanacearum_CLsoZC1_
Silicibacter_lacuscaerulensis_ITI1157_
Ruegeria_KLH11_
Ruegeria_pomeroyi_DSS3_
Ruegeria_TM1040_
Roseovarius_nubinhibens_ISM_
Nautella_italica_R11_
Roseobacter_MED193_
Roseobacter_SK20926_
Phaeobacter_gallaeciensis_210_
Rhodobacterales_Y4I_
Ketogulonicigenium_vulgare_Y25_
Oceanicola_granulosus_HTCC2516_
Citreicella_SE45_
Pelagibaca_bermudensis_HTCC2601_
Roseovarius_TM1035_
Oceanibulbus_indolifex_HEL45_
Loktanella_vestfoldensis_SKA53_
Loktanella_CCS2_
Rhodobacterales_HTCC2150_
Sulfitobacter_GAI101_
Sulfitobacter_NAS141_
Roseobacter_denitrificans_OCh_114_
Roseobacter_litoralis_Och_149_
Jannaschia_CCS1_
Thalassiobium_R2A62_
Octadecabacter_antarcticus_307_
Sagittula_stellata_E37_
Rhodobacterales_HTCC2083_
Maritimibacter_alkaliphilus_HTCC2654_
Dinoroseobacter_shibae_DFL12_DSM_16493_
Rhodobacter_SW2_
Rhodobacter_sphaeroides_WS8N_
Rhodobacter_capsulatus_SB1003_
Paracoccus_denitrificans_PD1222_
Hyphomonas_neptunium_ATCC_15444_
Hirschia_baltica_ATCC_49814_
Maricaulis_maris_MCS10_
Bradyrhizobium_japonicum_USDA_110_
Bradyrhizobium_BTAi1_
Bradyrhizobium_ORS278_
Rhodopseudomonas_palustris_BisB5_
Nitrobacter_Nb311A_
Nitrobacter_winogradskyi_Nb255_
Nitrobacter_hamburgensis_X14_
Bradyrhizobiaceae_SG6C_
Afipia_1NLS2_
Oligotropha_carboxidovorans_OM5_
Methylobacterium_extorquens_AM1_
Methylobacterium_chloromethanicum_CM4_
Methylobacterium_populi_BJ001_
Methylobacterium_nodulans_ORS_2060_
Methylobacterium_446_
Methylobacterium_radiotolerans_JCM_2831_
Methylocystis_Rockwell_ATCC_49242_
Methylosinus_trichosporium_OB3b_
Beijerinckia_indica_indica_ATCC_9039_
Methylocella_silvestris_BL2_DSM_15510_
Azorhizobium_caulinodans_ORS_571_
Xanthobacter_autotrophicus_Py2_
Starkeya_novella_DSM_506_
Brevundimonas_diminuta_ATCC_11568_
Brevundimonas_subvibrioides_ATCC_15264_
Brevundimonas_BAL3_
Asticcacaulis_biprosthecum_C19_
Asticcacaulis_excentricus_CB_48_
Caulobacter_segnis_ATCC_21756_
Caulobacter_crescentus_CB15_
Caulobacter_K31_
Phenylobacterium_zucineum_HLK1_
Hyphomicrobium_denitrificans_ATCC_51888_
Hyphomicrobium_MC1_
Rhodomicrobium_vannielii_ATCC_17100_
Labrenzia_aggregata_IAM_12614_
Labrenzia_alexandrii_DFL11_
Roseibium_TrichSKD4_
Parvularcula_bermudensis_HTCC2503_
Parvibaculum_lavamentivorans_DS1_
Erythrobacter_NAP1_
Erythrobacter_litoralis_HTCC2594_
Citromicrobium_bathyomarinum_JL354_
Novosphingobium_aromaticivorans_DSM_12444_
Novosphingobium_nitrogenifigens_DSM_19370_
Novosphingobium_PP1Y_
Sphingopyxis_alaskensis_RB2256_
Sphingobium_chlorophenolicum_L1_
Sphingobium_japonicum_UT26S_
Sphingomonas_S17_
Sphingomonas_wittichii_RW1_
Zymomonas_mobilis_mobilis_NCIMB_11163_
Acidiphilium_cryptum_JF5_
Acidiphilium_multivorum_AIU301_
Granulibacter_bethesdensis_CGDNIH1_
Gluconacetobacter_SXCC1_
Gluconacetobacter_diazotrophicus_PAI_5_DSM_5601_
Acetobacter_pomorum_DM001_
Acetobacter_pasteurianus_IFO_328301_
Acetobacter_tropicalis_NBRC_101654_
Gluconobacter_oxydans_621H_
Magnetospirillum_magneticum_AMB1_
Magnetospirillum_magnetotacticum_MS1_
Rhodospirillum_rubrum_S1_ATCC_11170_
Azospirillum_B510_
Rhodocista_centenaria_SW_
Candidatus_Puniceispirillum_marinum_IMCC1322_
Nisaea_BAL199_
Caedibacter_caryophilus_221_
Candidatus_Odyssella_thessalonicensis_L13_
Acanthamoeba_castellanii_mitochondrion_
Hartmannella_vermiformis_mitochondrion_
Naegleria_gruberi_mitochondrion_
Dictyostelium_discoideum_mitochondrion_
Polysphondylium_pallidum_mitochondrion_
Cyanidioschyzon_merolae_mitochondrion_
Chondrus_crispus_mitochondrion_
Ochromonas_danica_mitochondrion_
Chattonella_marina_mitochondrion_
Pilayella_littoralis_mitochondrion_
Saprolegnia_ferax_strain_ATCC_36051_mitochondrion_
Phytophthora_infestans_mitochondrion_
Cyanophora_paradoxa_strain_CCMP_329_mitochondrion_
Glaucocystis_nostochinearum_mitochondrion_
Malawimonas_jakobiformis_mitochondrion_
Reclinomonas_americana_mitochondrion_
Prototheca_wickerhamii_263-11_mitochondrion_
Megaceros_aenigmaticus_mitochondrion_
Marchantia_polymorpha_mitochondrion_
Arabidopsis_thaliana_mitochondrion_
Chaetosphaeridium_globosum_mitochondrion_
Chara_vulgaris_mitochondrion_
Chlorokybus_atmophyticus_strain_SAG_4880_mitochondrion_
Nephroselmis_olivacea_mitochondrion_
Candidatus_Pelagibacter_ubique_HTCC1002_
Candidatus_Pelagibacter_HTCC7211_
Pelagibacteraceae_HIMB5B_
Pelagibacteraceae_313_
Pelagibacteraceae_440_
Pelagibacteraceae_IMCC9063_
Pelagibacteraceae_HIMB114_
Pelagibacteraceae_HIMB59_
Ehrlichia_chaffeensis_Arkansas_
Ehrlichia_canis_Jake_
Ehrlichia_ruminantium_Gardel_
Anaplasma_marginale_Puerto_Rico_
Anaplasma_centrale_str_Israel_
Anaplasma_phagocytophilum_HZ_
Wolbachia_of_Brugia_malayi_
Wolbachia_of_Culex_quinquefasciatus_JHB_
Wolbachia_of_Drosophila_melanogaster_
Neorickettsia_sennetsu_Miyayama_
Neorickettsia_risticii_Illinois_
Rickettsia_canadensis_McKiel_
Rickettsia_felis_URRWXCal2_
Rickettsia_bellii_RML369C_
Rickettsia_typhi_Wilmington_
Orientia_tsutsugamushi_Ikeda_
Candidatus_Midichloria_mitochondrii_IricVA_
Magnetococcus_MC1_
Thiobacillus_denitrificans_ATCC_25259_
Ralstonia_solanacearum_GMI1000_
Xanthomonas_campestris_8004_
Escherichia_coli_MG1655_
Acidithiobacillus_ferrooxidans_ATCC_23270_
Campylobacter_jejuni_ICDCCJ07001_
Mariprofundus_ferrooxydans_PV1_
0.2

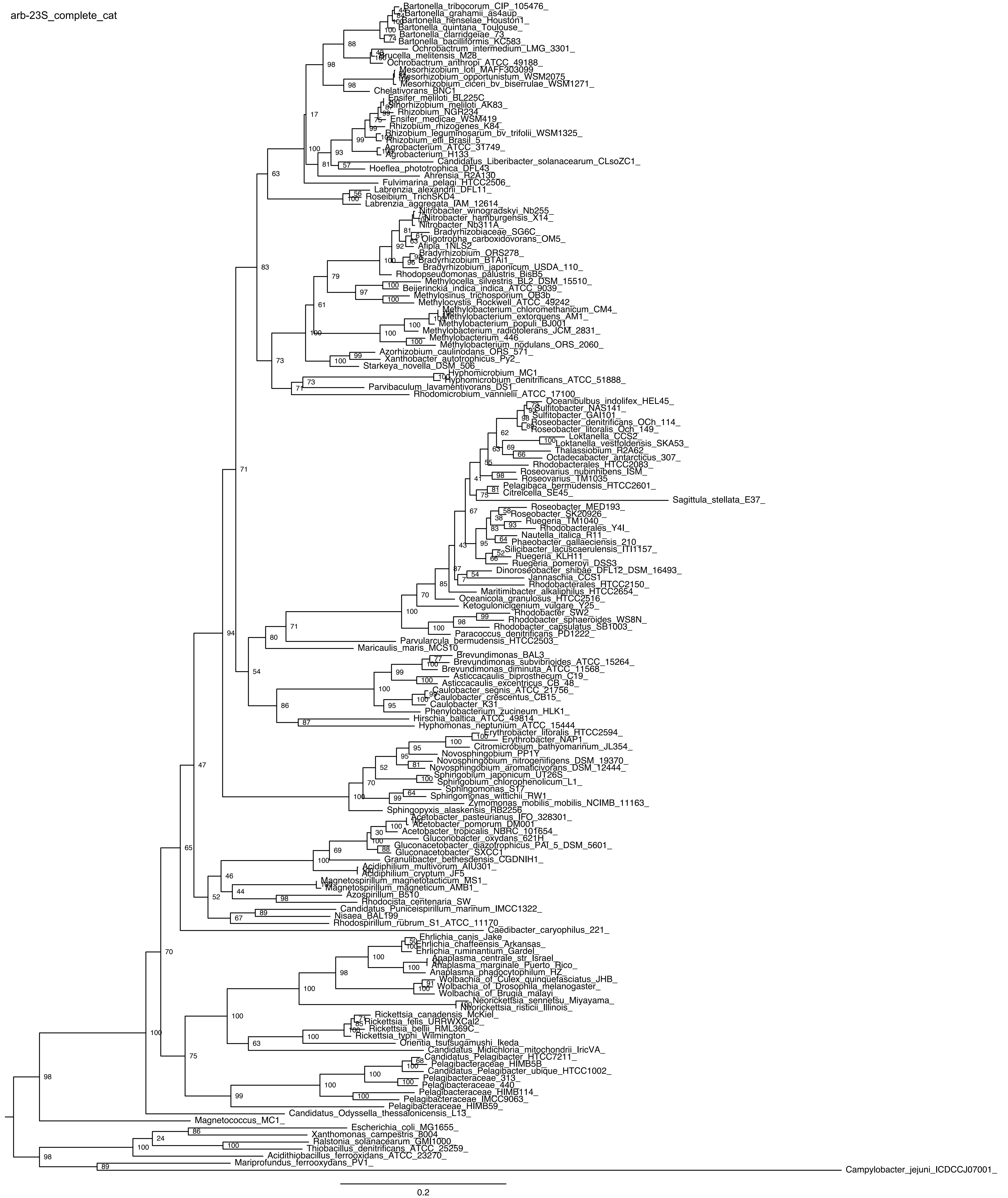
arb-23S_complete_cat
Bartonella_tribocorum_CIP_105476_
Bartonella_grahamii_as4aup_
Bartonella_henselae_Houston1_
Bartonella_quintana_Toulouse_
Bartonella_clarridgeiae_73_
Bartonella_bacilliformis_KC583_
Ochrobactrum_intermedium_LMG_3301_
Brucella_melitensis_M28_
Ochrobactrum_anthropi_ATCC_49188_
Mesorhizobium_loti_MAFF303099_
Mesorhizobium_opportunistum_WSM2075_
Mesorhizobium_ciceri_bv_biserrulae_WSM1271_
Chelativorans_BNC1_
Ensifer_meliloti_BL225C_
Sinorhizobium_meliloti_AK83_
Rhizobium_NGR234_
Ensifer_medicae_WSM419_
Rhizobium_rhizogenes_K84_
Rhizobium_leguminosarum_bv_trifolii_WSM1325_
Rhizobium_etli_Brasil_5_
Agrobacterium_ATCC_31749_
Agrobacterium_H13_
Candidatus_Liberibacter_solanacearum_CLsoZC1_
Hoeflea_phototrophica_DFL43_
Ahrensia_R2A130_
Fulvimarina_pelagi_HTCC2506_
Labrenzia_alexandrii_DFL11_
Roseibium_TrichSKD4_
Labrenzia_aggregata_IAM_12614_
Nitrobacter_winogradskyi_Nb255_
Nitrobacter_hamburgensis_X14_
Nitrobacter_Nb311A_
Bradyrhizobiaceae_SG6C_
Oligotropha_carboxidovorans_OM5_
Afipia_1NLS2_
Bradyrhizobium_ORS278_
Bradyrhizobium_BTAi1_
Bradyrhizobium_japonicum_USDA_110_
Rhodopseudomonas_palustris_BisB5_
Methylocella_silvestris_BL2_DSM_15510_
Beijerinckia_indica_indica_ATCC_9039_
Methylosinus_trichosporium_OB3b_
Methylocystis_Rockwell_ATCC_49242_
Methylobacterium_chloromethanicum_CM4_
Methylobacterium_extorquens_AM1_
Methylobacterium_populi_BJ001_
Methylobacterium_radiotolerans_JCM_2831_
Methylobacterium_446_
Methylobacterium_nodulans_ORS_2060_
Azorhizobium_caulinodans_ORS_571_
Xanthobacter_autotrophicus_Py2_
Starkeya_novella_DSM_506_
Hyphomicrobium_MC1_
Hyphomicrobium_denitrificans_ATCC_51888_
Parvibaculum_lavamentivorans_DS1_
Rhodomicrobium_vannielii_ATCC_17100_
Oceanibulbus_indolifex_HEL45_
Sulfitobacter_NAS141_
Sulfitobacter_GAI101_
Roseobacter_denitrificans_OCh_114_
Roseobacter_litoralis_Och_149_
Loktanella_CCS2_
Loktanella_vestfoldensis_SKA53_
Thalassiobium_R2A62_
Octadecabacter_antarcticus_307_
Rhodobacterales_HTCC2083_
Roseovarius_nubinhibens_ISM_
Roseovarius_TM1035_
Pelagibaca_bermudensis_HTCC2601_
Citreicella_SE45_
Sagittula_stellata_E37_
Roseobacter_MED193_
Roseobacter_SK20926_
Ruegeria_TM1040_
Rhodobacterales_Y4I_
Nautella_italica_R11_
Phaeobacter_gallaeciensis_210_
Silicibacter_lacuscaerulensis_ITI1157_
Ruegeria_KLH11_
Ruegeria_pomeroyi_DSS3_
Dinoroseobacter_shibae_DFL12_DSM_16493_
Jannaschia_CCS1_
Rhodobacterales_HTCC2150_
Maritimibacter_alkaliphilus_HTCC2654_
Oceanicola_granulosus_HTCC2516_
Ketogulonicigenium_vulgare_Y25_
Rhodobacter_SW2_
Rhodobacter_sphaeroides_WS8N_
Rhodobacter_capsulatus_SB1003_
Paracoccus_denitrificans_PD1222_
Parvularcula_bermudensis_HTCC2503_
Maricaulis_maris_MCS10_
Brevundimonas_BAL3_
Brevundimonas_subvibrioides_ATCC_15264_
Brevundimonas_diminuta_ATCC_11568_
Asticcacaulis_biprosthecum_C19_
Asticcacaulis_excentricus_CB_48_
Caulobacter_segnis_ATCC_21756_
Caulobacter_crescentus_CB15_
Caulobacter_K31_
Phenylobacterium_zucineum_HLK1_
Hirschia_baltica_ATCC_49814_
Hyphomonas_neptunium_ATCC_15444_
Erythrobacter_litoralis_HTCC2594_
Erythrobacter_NAP1_
Citromicrobium_bathyomarinum_JL354_
Novosphingobium_PP1Y_
Novosphingobium_nitrogenifigens_DSM_19370_
Novosphingobium_aromaticivorans_DSM_12444_
Sphingobium_japonicum_UT26S_
Sphingobium_chlorophenolicum_L1_
Sphingomonas_S17_
Sphingomonas_wittichii_RW1_
Zymomonas_mobilis_mobilis_NCIMB_11163_
Sphingopyxis_alaskensis_RB2256_
Acetobacter_pasteurianus_IFO_328301_
Acetobacter_pomorum_DM001_
Acetobacter_tropicalis_NBRC_101654_
Gluconobacter_oxydans_621H_
Gluconacetobacter_diazotrophicus_PAl_5_DSM_5601_
Gluconacetobacter_SXCC1_
Granulibacter_bethesdensis_CGDNIH1_
Acidiphilium_multivorum_AIU301_
Acidiphilium_cryptum_JF5_
Magnetospirillum_magnetotacticum_MS1_
Magnetospirillum_magneticum_AMB1_
Azospirillum_B510_
Rhodocista_centenaria_SW_
Candidatus_Puniceispirillum_marinum_IMCC1322_
Nisaea_BAL199_
Rhodospirillum_rubrum_S1_ATCC_11170_
Caedibacter_caryophilus_221_
Ehrlichia_canis_Jake_
Ehrlichia_chaffeensis_Arkansas_
Ehrlichia_ruminantium_Gardel_
Anaplasma_centrale_str_Israel_
Anaplasma_marginale_Puerto_Rico_
Anaplasma_phagocytophilum_HZ_
Wolbachia_of_Culex_quinquefasciatus_JHB_
Wolbachia_of_Drosophila_melanogaster_
Wolbachia_of_Brugia_malayi_
Neorickettsia_sennetsu_Miyayama_
Neorickettsia_risticii_Illinois_
Rickettsia_canadensis_McKiel_
Rickettsia_felis_URRWXCal2_
Rickettsia_bellii_RML369C_
Rickettsia_typhi_Wilmington_
Orientia_tsutsugamushi_Ikeda_
Candidatus_Midichloria_mitochondrii_IricVA_
Candidatus_Pelagibacter_HTCC7211_
Pelagibacteraceae_HIMB5B_
Candidatus_Pelagibacter_ubique_HTCC1002_
Pelagibacteraceae_313_
Pelagibacteraceae_440_
Pelagibacteraceae_HIMB114_
Pelagibacteraceae_IMCC9063_
Pelagibacteraceae_HIMB59_
Candidatus_Odyssella_thessalonicensis_L13_
Magnetococcus_MC1_
Escherichia_coli_MG1655_
Xanthomonas_campestris_8004_
Ralstonia_solanacearum_GMI1000_
Thiobacillus_denitrificans_ATCC_25259_
Acidithiobacillus_ferrooxidans_ATCC_23270_
Mariprofundus_ferrooxydans_PV1_
Campylobacter_jejuni_ICDCCJ07001_
0.2

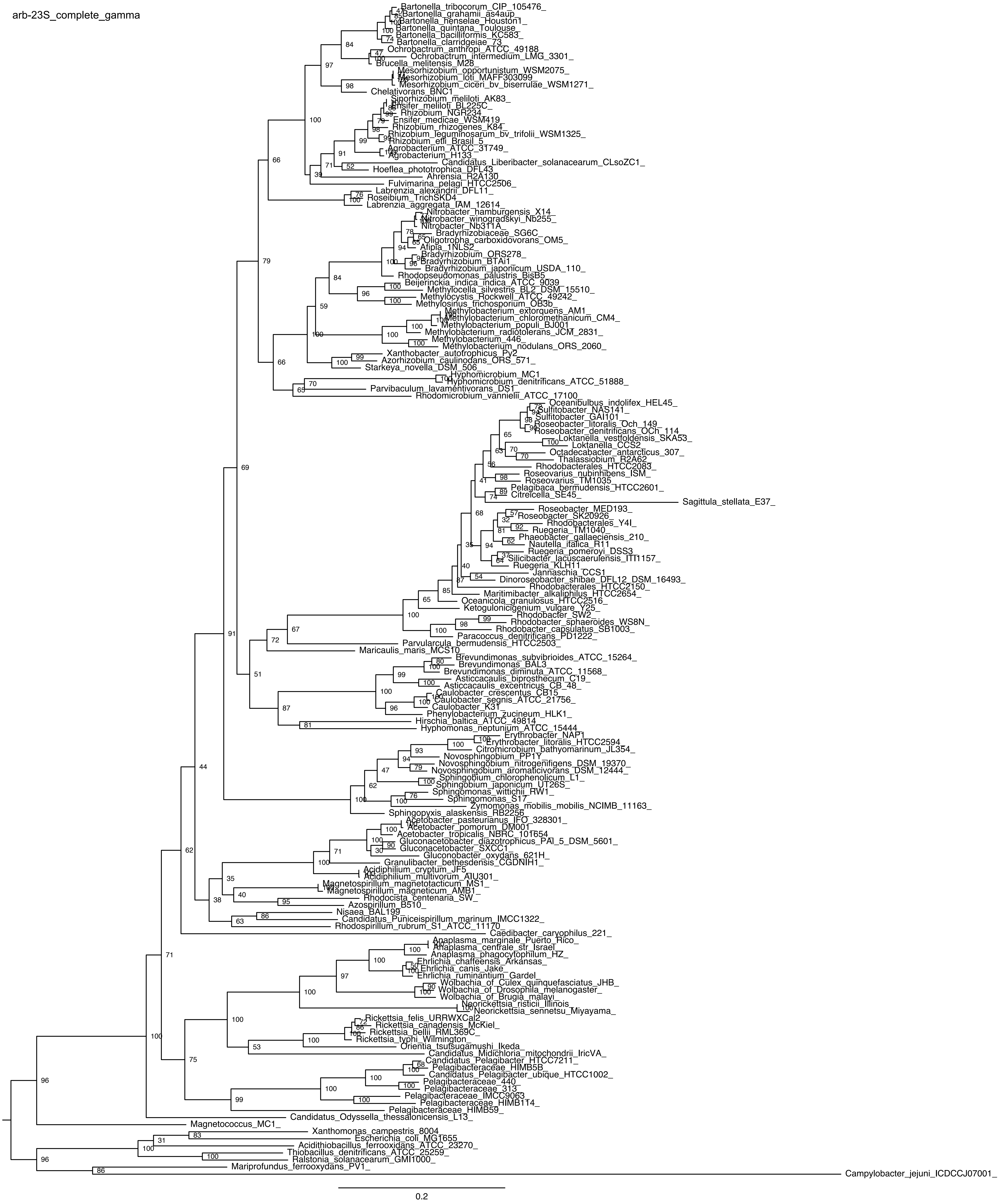
arb-23S_complete_gamma
Bartonella_tribocorum_CIP_105476_
Bartonella_grahamii_as4aup_
Bartonella_henselae_Houston1_
Bartonella_quintana_Toulouse_
Bartonella_bacilliformis_KC583_
Bartonella_clarridgeiae_73_
Ochrobactrum_anthropi_ATCC_49188_
Ochrobactrum_intermedium_LMG_3301_
Brucella_melitensis_M28_
Mesorhizobium_opportunistum_WSM2075_
Mesorhizobium_loti_MAFF303099_
Mesorhizobium_ciceri_bv_biserrulae_WSM1271_
Chelativorans_BNC1_
Sinorhizobium_meliloti_AK83_
Ensifer_meliloti_BL225C_
Rhizobium_NGR234_
Ensifer_medicae_WSM419_
Rhizobium_rhizogenes_K84_
Rhizobium_leguminosarum_bv_trifolii_WSM1325_
Rhizobium_etli_Brasil_5_
Agrobacterium_ATCC_31749_
Agrobacterium_H133_
Candidatus_Liberibacter_solanacearum_CLsoZC1_
Hoeflea_phototrophica_DFL43_
Ahrensia_R2A130_
Fulvimarina_pelagi_HTCC2506_
Labrenzia_alexandrii_DFL11_
Roseibium_TrichSKD4_
Labrenzia_aggregata_IAM_12614_
Nitrobacter_hamburgensis_X14_
Nitrobacter_winogradskyi_Nb255_
Nitrobacter_Nb311A_
Bradyrhizobiaceae_SG6C_
Oligotropha_carboxidovorans_OM5_
Afipia_1NLS2_
Bradyrhizobium_ORS278_
Bradyrhizobium_BTAi1_
Bradyrhizobium_japonicum_USDA_110_
Rhodopseudomonas_palustris_BisB5_
Beijerinckia_indica_indica_ATCC_9039_
Methylocella_silvestris_BL2_DSM_15510_
Methylocystis_Rockwell_ATCC_49242_
Methylosinus_trichosporium_OB3b_
Methylobacterium_extorquens_AM1_
Methylobacterium_chloromethanicum_CM4_
Methylobacterium_populi_BJ001_
Methylobacterium_radiotolerans_JCM_2831_
Methylobacterium_446_
Methylobacterium_nodulans_ORS_2060_
Xanthobacter_autotrophicus_Py2_
Azorhizobium_caulinodans_ORS_571_
Starkeya_novella_DSM_506_
Hyphomicrobium_MC1_
Hyphomicrobium_denitrificans_ATCC_51888_
Parvibaculum_lavamentivorans_DS1_
Rhodomicrobium_vannielii_ATCC_17100_
Oceanibulbus_indolifex_HEL45_
Sulfitobacter_NAS141_
Sulfitobacter_GAI101_
Roseobacter_litoralis_Och_149_
Roseobacter_denitrificans_OCh_114_
Loktanella_vestfoldensis_SKA53_
Loktanella_CCS2_
Octadecabacter_antarcticus_307_
Thalassiobium_R2A62_
Rhodobacterales_HTCC2083_
Roseovarius_nubinhibens_ISM_
Roseovarius_TM1035_
Pelagibaca_bermudensis_HTCC2601_
Citreicella_SE45_
Sagittula_stellata_E37_
Roseobacter_MED193_
Roseobacter_SK20926_
Rhodobacterales_Y4I_
Ruegeria_TM1040_
Phaeobacter_gallaeciensis_210_
Nautella_italica_R11_
Ruegeria_pomeroyi_DSS3_
Silicibacter_lacuscaerulensis_ITI1157_
Ruegeria_KLH11_
Jannaschia_CCS1_
Dinoroseobacter_shibae_DFL12_DSM_16493_
Rhodobacterales_HTCC2150_
Maritimibacter_alkaliphilus_HTCC2654_
Oceanicola_granulosus_HTCC2516_
Ketogulonicigenium_vulgare_Y25_
Rhodobacter_SW2_
Rhodobacter_sphaeroides_WS8N_
Rhodobacter_capsulatus_SB1003_
Paracoccus_denitrificans_PD1222_
Parvularcula_bermudensis_HTCC2503_
Maricaulis_maris_MCS10_
Brevundimonas_subvibrioides_ATCC_15264_
Brevundimonas_BAL3_
Brevundimonas_diminuta_ATCC_11568_
Asticcacaulis_biprosthecum_C19_
Asticcacaulis_excentricus_CB_48_
Caulobacter_crescentus_CB15_
Caulobacter_segnis_ATCC_21756_
Caulobacter_K31_
Phenylobacterium_zucineum_HLK1_
Hirschia_baltica_ATCC_49814_
Hyphomonas_neptunium_ATCC_15444_
Erythrobacter_NAP1_
Erythrobacter_litoralis_HTCC2594_
Citromicrobium_bathyomarinum_JL354_
Novosphingobium_PP1Y_
Novosphingobium_nitrogenifigens_DSM_19370_
Novosphingobium_aromaticivorans_DSM_12444_
Sphingobium_chlorophenolicum_L1_
Sphingobium_japonicum_UT26S_
Sphingomonas_wittichii_RW1_
Sphingomonas_S17_
Zymomonas_mobilis_mobilis_NCIMB_11163_
Sphingopyxis_alaskensis_RB2256_
Acetobacter_pasteurianus_IFO_328301_
Acetobacter_pomorum_DM001_
Acetobacter_tropicalis_NBRC_101654_
Gluconacetobacter_diazotrophicus_PAl_5_DSM_5601_
Gluconacetobacter_SXCC1_
Gluconobacter_oxydans_621H_
Granulibacter_bethesdensis_CGDNIH1_
Acidiphilium_cryptum_JF5_
Acidiphilium_multivorum_AIU301_
Magnetospirillum_magnetotacticum_MS1_
Magnetospirillum_magneticum_AMB1_
Rhodocista_centenaria_SW_
Azospirillum_B510_
Nisaea_BAL199_
Candidatus_Puniceispirillum_marinum_IMCC1322_
Rhodospirillum_rubrum_S1_ATCC_11170_
Caedibacter_caryophilus_221_
Anaplasma_marginale_Puerto_Rico_
Anaplasma_centrale_str_Israel_
Anaplasma_phagocytophilum_HZ_
Ehrlichia_chaffeensis_Arkansas_
Ehrlichia_canis_Jake_
Ehrlichia_ruminantium_Gardel_
Wolbachia_of_Culex_quinquefasciatus_JHB_
Wolbachia_of_Drosophila_melanogaster_
Wolbachia_of_Brugia_malayi_
Neorickettsia_risticii_Illinois_
Neorickettsia_sennetsu_Miyayama_
Rickettsia_felis_URRWXCal2_
Rickettsia_canadensis_McKiel_
Rickettsia_bellii_RML369C_
Rickettsia_typhi_Wilmington_
Orientia_tsutsugamushi_Ikeda_
Candidatus_Midichloria_mitochondrii_IricVA_
Candidatus_Pelagibacter_HTCC7211_
Pelagibacteraceae_HIMB5B_
Candidatus_Pelagibacter_ubique_HTCC1002_
Pelagibacteraceae_440_
Pelagibacteraceae_313_
Pelagibacteraceae_IMCC9063_
Pelagibacteraceae_HIMB114_
Pelagibacteraceae_HIMB59_
Candidatus_Odyssella_thessalonicensis_L13_
Magnetococcus_MC1_
Xanthomonas_campestris_8004_
Escherichia_coli_MG1655_
Acidithiobacillus_ferrooxidans_ATCC_23270_
Thiobacillus_denitrificans_ATCC_25259_
Ralstonia_solanacearum_GMI1000_
Mariprofundus_ferrooxydans_PV1_
Campylobacter_jejuni_ICDCCJ07001_
0.2

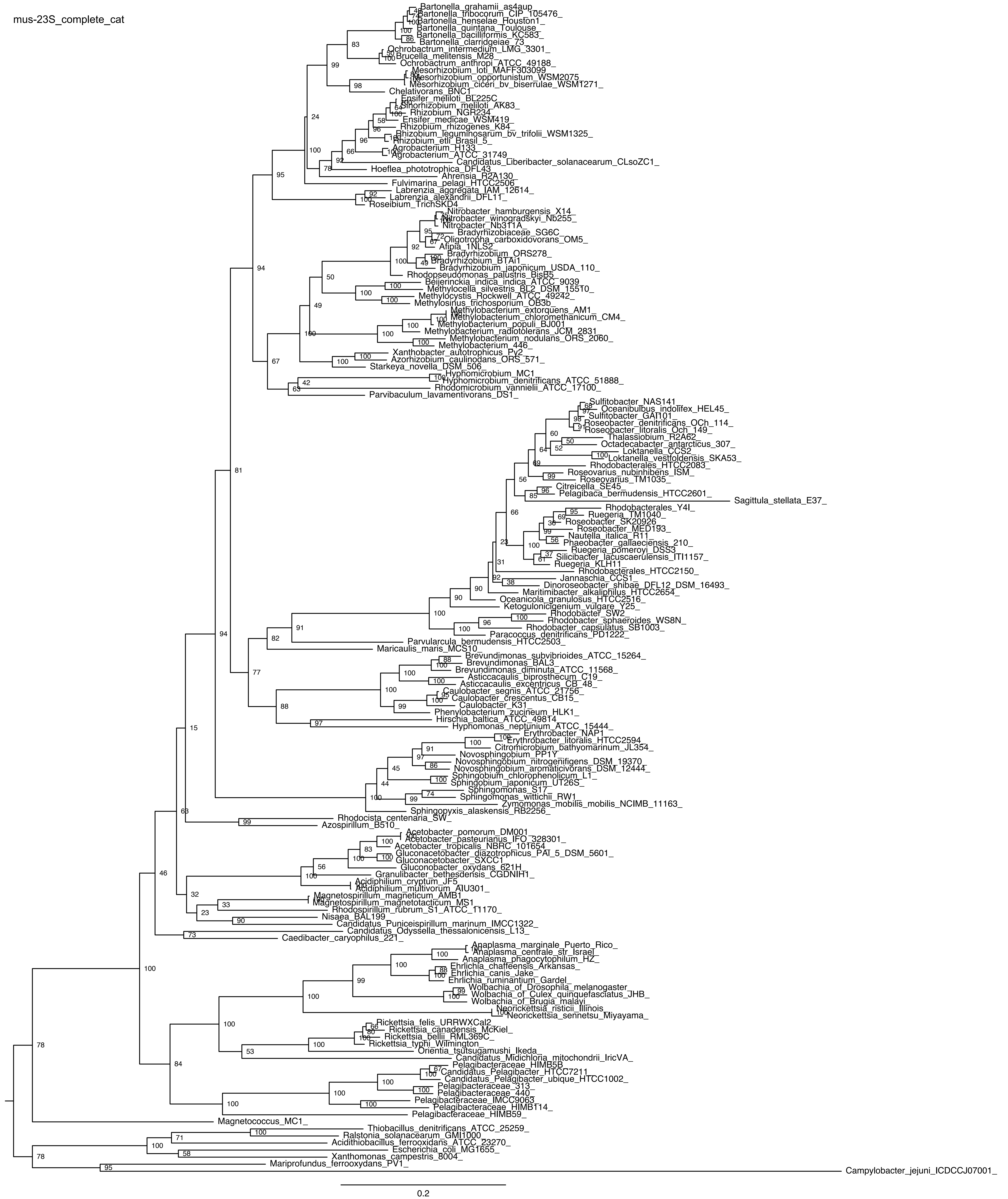
mus-23S_complete_cat
Bartonella_grahamii_as4aup_
Bartonella_tribocorum_CIP_105476_
Bartonella_henselae_Houston1_
Bartonella_quintana_Toulouse_
Bartonella_bacilliformis_KC583_
Bartonella_clarridgeiae_73_
Ochrobactrum_intermedium_LMG_3301_
Brucella_melitensis_M28_
Ochrobactrum_anthropi_ATCC_49188_
Mesorhizobium_loti_MAFF303099_
Mesorhizobium_opportunistum_WSM2075_
Mesorhizobium_ciceri_bv_biserrulae_WSM1271_
Chelativorans_BNC1_
Ensifer_meliloti_BL225C_
Sinorhizobium_meliloti_AK83_
Rhizobium_NGR234_
Ensifer_medicae_WSM419_
Rhizobium_rhizogenes_K84_
Rhizobium_leguminosarum_bv_trifolii_WSM1325_
Rhizobium_etli_Brasil_5_
Agrobacterium_H13-3_
Agrobacterium_ATCC_31749_
Candidatus_Liberibacter_solanacearum_CLsoZC1_
Hoeflea_phototrophica_DFL43_
Ahrensia_R2A130_
Fulvimarina_pelagi_HTCC2506_
Labrenzia_aggregata_IAM_12614_
Labrenzia_alexandrii_DFL11_
Roseibium_TrichSKD4_
Nitrobacter_hamburgensis_X14_
Nitrobacter_winogradskyi_Nb255_
Nitrobacter_Nb311A_
Bradyrhizobiaceae_SG6C_
Oligotropha_carboxidovorans_OM5_
Afipia_1NLS2_
Bradyrhizobium_ORS278_
Bradyrhizobium_BTAi1_
Bradyrhizobium_japonicum_USDA_110_
Rhodopseudomonas_palustris_BisB5_
Beijerinckia_indica_indica_ATCC_9039_
Methylocella_silvestris_BL2_DSM_15510_
Methylocystis_Rockwell_ATCC_49242_
Methylosinus_trichosporium_OB3b_
Methylobacterium_extorquens_AM1_
Methylobacterium_chloromethanicum_CM4_
Methylobacterium_populi_BJ001_
Methylobacterium_radiotolerans_JCM_2831_
Methylobacterium_nodulans_ORS_2060_
Methylobacterium_4-46_
Xanthobacter_autotrophicus_Py2_
Azorhizobium_caulinodans_ORS_571_
Starkeya_novella_DSM_506_
Hyphomicrobium_MC1_
Hyphomicrobium_denitrificans_ATCC_51888_
Rhodomicrobium_vannielii_ATCC_17100_
Parvibaculum_lavamentivorans_DS1_
Sulfitobacter_NAS141_
Oceanibulbus_indolifex_HEL45_
Sulfitobacter_GAI101_
Roseobacter_denitrificans_OCh_114_
Roseobacter_litoralis_Och_149_
Thalassiobium_R2A62_
Octadecabacter_antarcticus_307_
Loktanella_CCS2_
Loktanella_vestfoldensis_SKA53_
Rhodobacterales_HTCC2083_
Roseovarius_nubinhibens_ISM_
Roseovarius_TM1035_
Citreicella_SE45_
Pelagibaca_bermudensis_HTCC2601_
Sagittula_stellata_E37_
Rhodobacterales_Y4I_
Ruegeria_TM1040_
Roseobacter_SK209-2-6_
Roseobacter_MED193_
Nautella_italica_R11_
Phaeobacter_gallaeciensis_2-10_
Ruegeria_pomeroyi_DSS3_
Silicibacter_lacuscaerulensis_ITI1157_
Ruegeria_KLH11_
Rhodobacterales_HTCC2150_
Jannaschia_CCS1_
Dinoroseobacter_shibae_DFL12_DSM_16493_
Maritimibacter_alkaliphilus_HTCC2654_
Oceanicola_granulosus_HTCC2516_
Ketogulonicigenium_vulgare_Y25_
Rhodobacter_SW2_
Rhodobacter_sphaeroides_WS8N_
Rhodobacter_capsulatus_SB1003_
Paracoccus_denitrificans_PD1222_
Parvularcula_bermudensis_HTCC2503_
Maricaulis_maris_MCS10_
Brevundimonas_subvibrioides_ATCC_15264_
Brevundimonas_BAL3_
Brevundimonas_diminuta_ATCC_11568_
Asticcacaulis_biprosthecum_C19_
Asticcacaulis_excentricus_CB_48_
Caulobacter_segnis_ATCC_21756_
Caulobacter_crescentus_CB15_
Caulobacter_K31_
Phenylobacterium_zucineum_HLK1_
Hirschia_baltica_ATCC_49814_
Hyphomonas_neptunium_ATCC_15444_
Erythrobacter_NAP1_
Erythrobacter_litoralis_HTCC2594_
Citromicrobium_bathyomarinum_JL354_
Novosphingobium_PP1Y_
Novosphingobium_nitrogenifigens_DSM_19370_
Novosphingobium_aromaticivorans_DSM_12444_
Sphingobium_chlorophenolicum_L-1_
Sphingobium_japonicum_UT26S_
Sphingomonas_S17_
Sphingomonas_wittichii_RW1_
Zymomonas_mobilis_mobilis_NCIMB_11163_
Sphingopyxis_alaskensis_RB2256_
Rhodocista_centenaria_SW_
Azospirillum_B510_
Acetobacter_pomorum_DM001_
Acetobacter_pasteurianus_IFO_3283-01_
Acetobacter_tropicalis_NBRC_101654_
Gluconacetobacter_diazotrophicus_PAl_5_DSM_5601_
Gluconacetobacter_SXCC-1_
Gluconobacter_oxydans_621H_
Granulibacter_bethesdensis_CGDNIH1_
Acidiphilium_cryptum_JF-5_
Acidiphilium_multivorum_AIU301_
Magnetospirillum_magneticum_AMB-1_
Magnetospirillum_magnetotacticum_MS-1_
Rhodospirillum_rubrum_S1_ATCC_11170_
Nisaea_BAL199_
Candidatus_Puniceispirillum_marinum_IMCC1322_
Candidatus_Odyssella_thessalonicensis_L13_
Caedibacter_caryophilus_221_
Anaplasma_marginale_Puerto_Rico_
Anaplasma_centrale_str_Israel_
Anaplasma_phagocytophilum_HZ_
Ehrlichia_chaffeensis_Arkansas_
Ehrlichia_canis_Jake_
Ehrlichia_ruminantium_Gardel_
Wolbachia_of_Drosophila_melanogaster_
Wolbachia_of_Culex_quinquefasciatus_JHB_
Wolbachia_of_Brugia_malayi_
Neorickettsia_risticii_Illinois_
Neorickettsia_sennetsu_Miyayama_
Rickettsia_felis_URRWXCal2_
Rickettsia_canadensis_McKiel_
Rickettsia_bellii_RML369C_
Rickettsia_typhi_Wilmington_
Orientia_tsutsugamushi_Ikeda_
Candidatus_Midichloria_mitochondrii_IricVA_
Pelagibacteraceae_HIMB5B_
Candidatus_Pelagibacter_HTCC7211_
Candidatus_Pelagibacter_ubique_HTCC1002_
Pelagibacteraceae_313_
Pelagibacteraceae_440_
Pelagibacteraceae_IMCC9063_
Pelagibacteraceae_HIMB114_
Pelagibacteraceae_HIMB59_
Magnetococcus_MC-1_
Thiobacillus_denitrificans_ATCC_25259_
Ralstonia_solanacearum_GMI1000_
Acidithiobacillus_ferrooxidans_ATCC_23270_
Escherichia_coli_MG1655_
Xanthomonas_campestris_8004_
Mariprofundus_ferrooxydans_PV-1_
Campylobacter_jejuni_ICDCCJ07001_
0.2

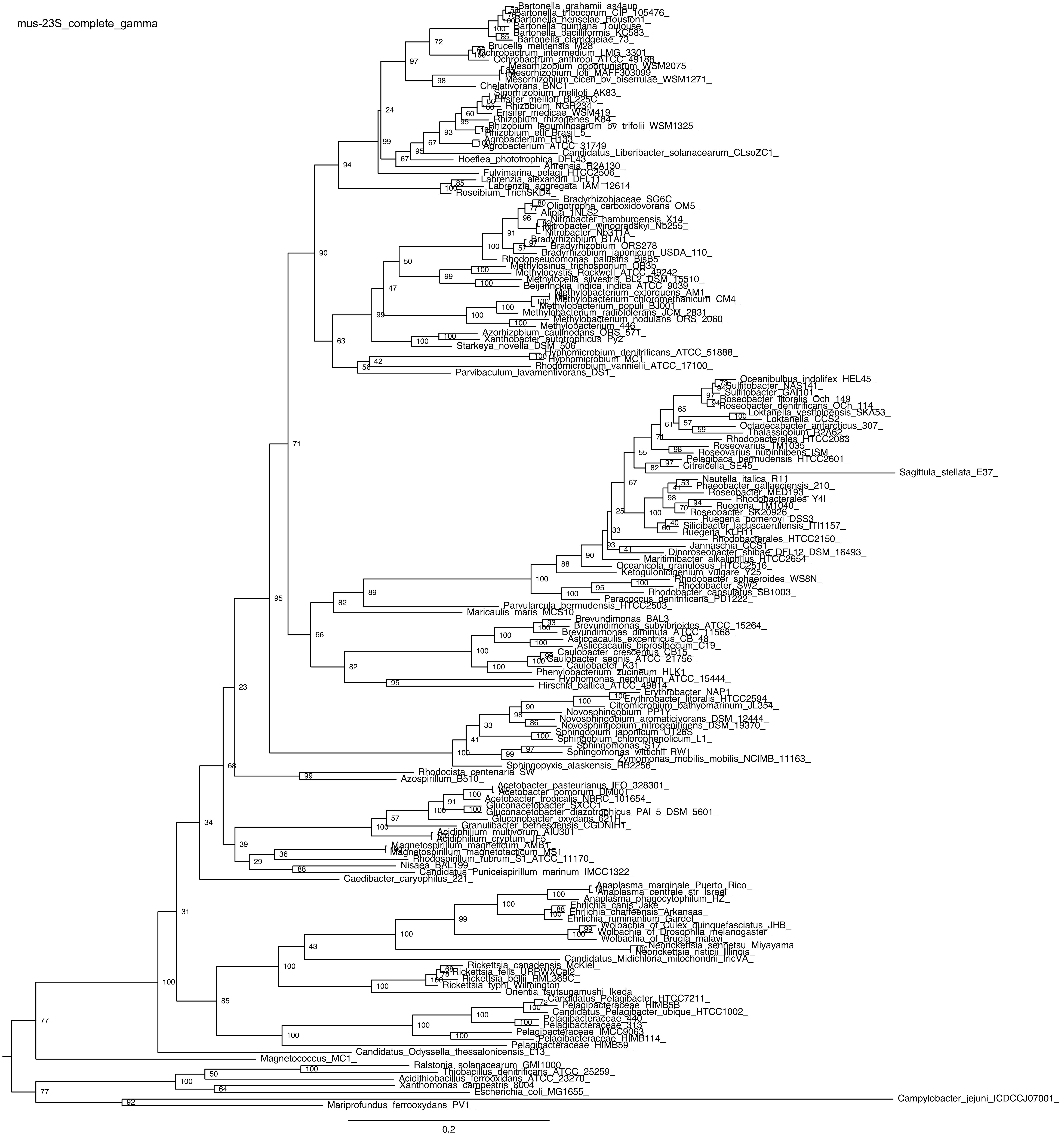
mus-23S_complete_gamma
Bartonella_grahamii_as4aup
Bartonella_tribocorum_CIP_105476_
Bartonella_henselae_Houston1_
Bartonella_quintana_Toulouse_
Bartonella_bacilliformis_KC583_
Bartonella_clarridgeiae_73_
Brucella_melitensis_M28_
Ochrobactrum_intermedium_LMG_3301_
Ochrobactrum_anthropi_ATCC_49188_
Mesorhizobium_opportunistum_WSM2075_
Mesorhizobium_loti_MAFF303099_
Mesorhizobium_ciceri_bv_biserrulae_WSM1271_
Chelativorans_BNC1_
Sinorhizobium_meliloti_AK83_
Ensifer_meliloti_BL225C_
Rhizobium_NGR234_
Ensifer_medicae_WSM419_
Rhizobium_rhizogenes_K84_
Rhizobium_leguminosarum_bv_trifolii_WSM1325_
Rhizobium_etli_Brasil_5_
Agrobacterium_H13_3_
Agrobacterium_ATCC_31749_
Candidatus_Liberibacter_solanacearum_CLsoZC1_
Hoeflea_phototrophica_DFL43_
Ahrensia_R2A130_
Fulvimarina_pelagi_HTCC2506_
Labrenzia_alexandrii_DFL11_
Labrenzia_aggregata_IAM_12614_
Roseibium_TrichSKD4_
Bradyrhizobiaceae_SG6C_
Oligotropha_carboxidovorans_OM5_
Afipia_1NLS2_
Nitrobacter_hamburgensis_X14_
Nitrobacter_winogradskyi_Nb255_
Nitrobacter_Nb311A_
Bradyrhizobium_BTAi1_
Bradyrhizobium_ORS278_
Bradyrhizobium_japonicum_USDA_110_
Rhodopseudomonas_palustris_BisB5_
Methylosinus_trichosporium_OB3b_
Methylocystis_Rockwell_ATCC_49242_
Methylocella_silvestris_BL2_DSM_15510_
Beijerinckia_indica_indica_ATCC_9039_
Methylobacterium_extorquens_AM1_
Methylobacterium_chloromethanicum_CM4_
Methylobacterium_populi_BJ001_
Methylobacterium_radiotolerans_JCM_2831_
Methylobacterium_nodulans_ORS_2060_
Methylobacterium_446_
Azorhizobium_caulinodans_ORS_571_
Xanthobacter_autotrophicus_Py2_
Starkeya_novella_DSM_506_
Hyphomicrobium_denitrificans_ATCC_51888_
Hyphomicrobium_MC1_
Rhodomicrobium_vannielii_ATCC_17100_
Parvibaculum_lavamentivorans_DS1_
Oceanibulbus_indolifex_HEL45_
Sulfitobacter_NAS141_
Sulfitobacter_GAI101_
Roseobacter_litoralis_Och_149_
Roseobacter_denitrificans_OCh_114_
Loktanella_vestfoldensis_SKA53_
Loktanella_CCS2_
Octadecabacter_antarcticus_307_
Thalassiobium_R2A62_
Rhodobacterales_HTCC2083_
Roseovarius_TM1035_
Roseovarius_nubinhibens_ISM_
Pelagibaca_bermudensis_HTCC2601_
Citreicella_SE45_
Sagittula_stellata_E37_
Nautella_italica_R11_
Phaeobacter_gallaeciensis_210_
Roseobacter_MED193_
Rhodobacterales_Y4I_
Ruegeria_TM1040_
Roseobacter_SK20926_
Ruegeria_pomeroyi_DSS3_
Silicibacter_lacuscaerulensis_ITI1157_
Ruegeria_KLH11_
Rhodobacterales_HTCC2150_
Jannaschia_CCS1_
Dinoroseobacter_shibae_DFL12_DSM_16493_
Maritimibacter_alkaliphilus_HTCC2654_
Oceanicola_granulosus_HTCC2516_
Ketogulonicigenium_vulgare_Y25_
Rhodobacter_sphaeroides_WS8N_
Rhodobacter_SW2_
Rhodobacter_capsulatus_SB1003_
Paracoccus_denitrificans_PD1222_
Parvularcula_bermudensis_HTCC2503_
Maricaulis_maris_MCS10_
Brevundimonas_BAL3_
Brevundimonas_subvibrioides_ATCC_15264_
Brevundimonas_diminuta_ATCC_11568_
Asticcacaulis_excentricus_CB_48_
Asticcacaulis_biprosthecum_C19_
Caulobacter_crescentus_CB15_
Caulobacter_segnis_ATCC_21756_
Caulobacter_K31_
Phenylobacterium_zucineum_HLK1_
Hyphomonas_neptunium_ATCC_15444_
Hirschia_baltica_ATCC_49814_
Erythrobacter_NAP1_
Erythrobacter_litoralis_HTCC2594_
Citromicrobium_bathyomarinum_JL354_
Novosphingobium_PP1Y_
Novosphingobium_aromaticivorans_DSM_12444_
Novosphingobium_nitrogenifigens_DSM_19370_
Sphingobium_japonicum_UT26S_
Sphingobium_chlorophenolicum_L1_
Sphingomonas_S17_
Sphingomonas_wittichii_RW1_
Zymomonas_mobilis_mobilis_NCIMB_11163_
Sphingopyxis_alaskensis_RB2256_
Rhodocista_centenaria_SW_
Azospirillum_B510_
Acetobacter_pasteurianus_IFO_328301_
Acetobacter_pomorum_DM001_
Acetobacter_tropicalis_NBRC_101654_
Gluconacetobacter_SXCC1_
Gluconacetobacter_diazotrophicus_PAI_5_DSM_5601_
Gluconobacter_oxydans_621H_
Granulibacter_bethesdensis_CGDNIH1_
Acidiphilium_multivorum_AIU301_
Acidiphilium_cryptum_JF5_
Magnetospirillum_magneticum_AMB1_
Magnetospirillum_magnetotacticum_MS1_
Rhodospirillum_rubrum_S1_ATCC_11170_
Nisaea_BAL199_
Candidatus_Puniceispirillum_marinum_IMCC1322_
Caedibacter_caryophilus_221_
Anaplasma_marginale_Puerto_Rico_
Anaplasma_centrale_str_Israel_
Anaplasma_phagocytophilum_HZ_
Ehrlichia_canis_Jake_
Ehrlichia_chaffeensis_Arkansas_
Ehrlichia_ruminantium_Gardel_
Wolbachia_of_Culex_quinquefasciatus_JHB_
Wolbachia_of_Drosophila_melanogaster_
Wolbachia_of_Brugia_malayi_
Neorickettsia_sennetsu_Miyayama_
Neorickettsia_risticii_Illinois_
Candidatus_Midichloria_mitochondrii_IricVA_
Rickettsia_canadensis_McKiel_
Rickettsia_felis_URRWXCal2_
Rickettsia_bellii_RML369C_
Rickettsia_typhi_Wilmington_
Orientia_tsutsugamushi_Ikeda_
Candidatus_Pelagibacter_HTCC7211_
Pelagibacteraceae_HIMB5B_
Candidatus_Pelagibacter_ubique_HTCC1002_
Pelagibacteraceae_440_
Pelagibacteraceae_313_
Pelagibacteraceae_IMCC9063_
Pelagibacteraceae_HIMB114_
Pelagibacteraceae_HIMB59_
Candidatus_Odyssella_thessalonicensis_L13_
Magnetococcus_MC1_
Ralstonia_solanacearum_GMI1000_
Thiobacillus_denitrificans_ATCC_25259_
Acidithiobacillus_ferrooxidans_ATCC_23270_
Xanthomonas_campestris_8004_
Escherichia_coli_MG1655_
Campylobacter_jejuni_ICDCCJ07001_
Mariprofundus_ferrooxydans_PV1_
0.2

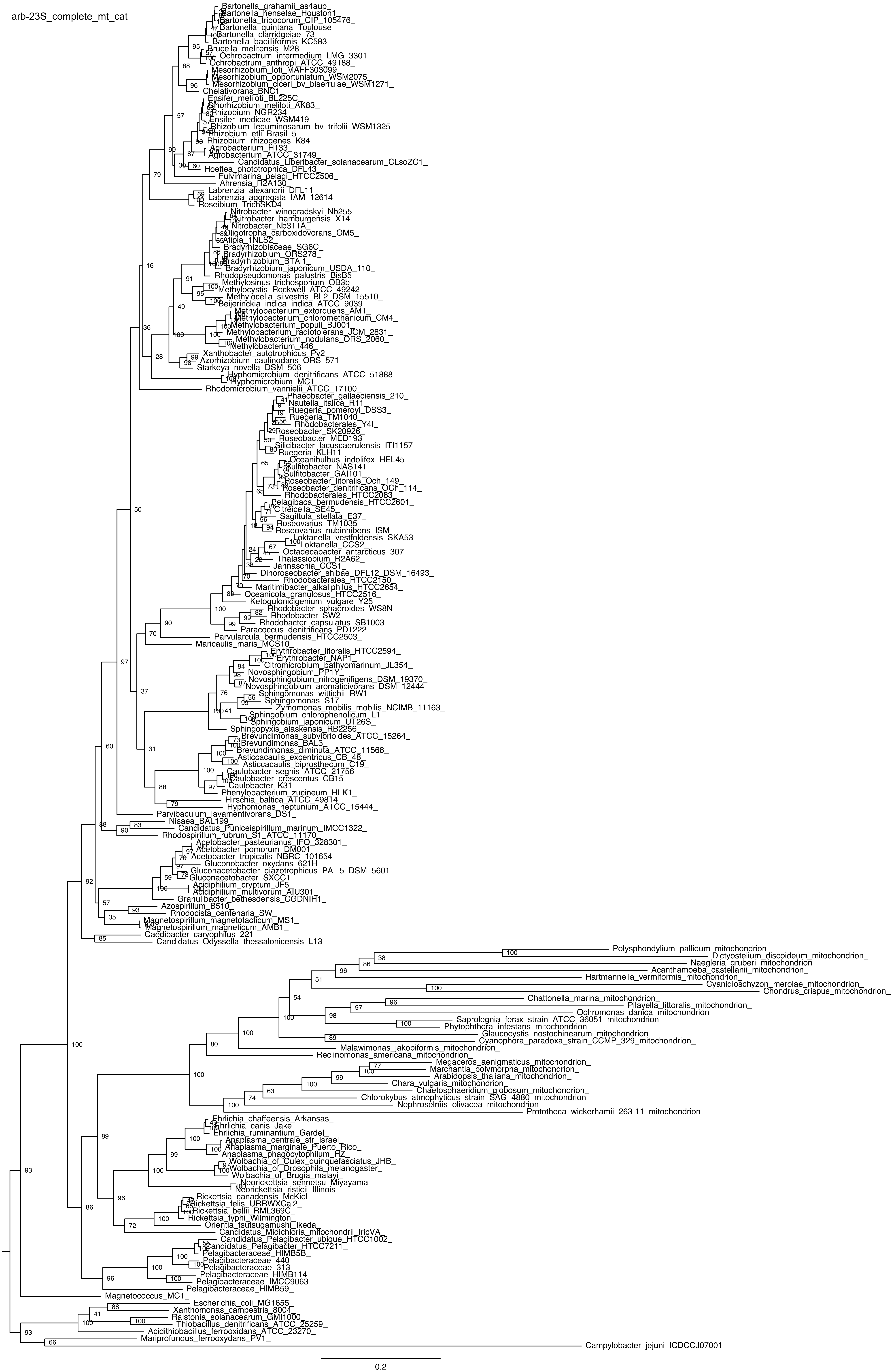
arb-23S_complete_mt_cat
Bartonella_grahamii_as4aup_
Bartonella_henselae_Houston1_
Bartonella_tribocorum_CIP_105476_
Bartonella_quintana_Toulouse_
Bartonella_clarridgeiae_73_
Bartonella_bacilliformis_KC583_
Brucella_melitensis_M28_
Ochrobactrum_intermedium_LMG_3301_
Ochrobactrum_anthropi_ATCC_49188_
Mesorhizobium_loti_MAFF303099_
Mesorhizobium_opportunistum_WSM2075_
Mesorhizobium_ciceri_bv_biserrulae_WSM1271_
Chelativorans_BNC1_
Ensifer_meliloti_BL225C_
Sinorhizobium_meliloti_AK83_
Rhizobium_NGR234_
Ensifer_medicae_WSM419_
Rhizobium_leguminosarum_bv_trifolii_WSM1325_
Rhizobium_etli_Brasil_5_
Rhizobium_rhizogenes_K84_
Agrobacterium_H133_
Agrobacterium_ATCC_31749_
Candidatus_Liberibacter_solanacearum_CLsoZC1_
Hoeflea_phototrophica_DFL43_
Fulvimarina_pelagi_HTCC2506_
Ahrensia_R2A130_
Labrenzia_alexandrii_DFL11_
Labrenzia_aggregata_IAM_12614_
Roseibium_TrichSKD4_
Nitrobacter_winogradskyi_Nb255_
Nitrobacter_hamburgensis_X14_
Nitrobacter_Nb311A_
Oligotropha_carboxidovorans_OM5_
Afipia_1NLS2_
Bradyrhizobiaceae_SG6C_
Bradyrhizobium_ORS278_
Bradyrhizobium_BTAi1_
Bradyrhizobium_japonicum_USDA_110_
Rhodopseudomonas_palustris_BisB5_
Methylosinus_trichosporium_OB3b_
Methylocystis_Rockwell_ATCC_49242_
Methylocella_silvestris_BL2_DSM_15510_
Beijerinckia_indica_indica_ATCC_9039_
Methylobacterium_extorquens_AM1_
Methylobacterium_chloromethanicum_CM4_
Methylobacterium_populi_BJ001_
Methylobacterium_radiotolerans_JCM_2831_
Methylobacterium_nodulans_ORS_2060_
Methylobacterium_446_
Xanthobacter_autotrophicus_Py2_
Azorhizobium_caulinodans_ORS_571_
Starkeya_novella_DSM_506_
Hyphomicrobium_denitrificans_ATCC_51888_
Hyphomicrobium_MC1_
Rhodomicrobium_vannielii_ATCC_17100_
Phaeobacter_gallaeciensis_210_
Nautella_italica_R11_
Ruegeria_pomeroyi_DSS3_
Ruegeria_TM1040_
Rhodobacterales_Y4I_
Roseobacter_SK20926_
Roseobacter_MED193_
Silicibacter_lacuscaerulensis_ITI1157_
Ruegeria_KLH11_
Oceanibulbus_indolifex_HEL45_
Sulfitobacter_NAS141_
Sulfitobacter_GAI101_
Roseobacter_litoralis_Och_149_
Roseobacter_denitrificans_OCh_114_
Rhodobacterales_HTCC2083_
Pelagibaca_bermudensis_HTCC2601_
Citreicella_SE45_
Sagittula_stellata_E37_
Roseovarius_TM1035_
Roseovarius_nubinhibens_ISM_
Loktanella_vestfoldensis_SKA53_
Loktanella_CCS2_
Octadecabacter_antarcticus_307_
Thalassiobium_R2A62_
Jannaschia_CCS1_
Dinoroseobacter_shibae_DFL12_DSM_16493_
Rhodobacterales_HTCC2150_
Maritimibacter_alkaliphilus_HTCC2654_
Oceanicola_granulosus_HTCC2516_
Ketogulonicigenium_vulgare_Y25_
Rhodobacter_sphaeroides_WS8N_
Rhodobacter_SW2_
Rhodobacter_capsulatus_SB1003_
Paracoccus_denitrificans_PD1222_
Parvularcula_bermudensis_HTCC2503_
Maricaulis_maris_MCS10_
Erythrobacter_litoralis_HTCC2594_
Erythrobacter_NAP1_
Citromicrobium_bathyomarinum_JL354_
Novosphingobium_PP1Y_
Novosphingobium_nitrogenifigens_DSM_19370_
Novosphingobium_aromaticivorans_DSM_12444_
Sphingomonas_wittichii_RW1_
Sphingomonas_S17_
Zymomonas_mobilis_mobilis_NCIMB_11163_
Sphingobium_chlorophenolicum_L1_
Sphingobium_japonicum_UT26S_
Sphingopyxis_alaskensis_RB2256_
Brevundimonas_subvibrioides_ATCC_15264_
Brevundimonas_BAL3_
Brevundimonas_diminuta_ATCC_11568_
Asticcacaulis_excentricus_CB_48_
Asticcacaulis_biprosthecum_C19_
Caulobacter_segnis_ATCC_21756_
Caulobacter_crescentus_CB15_
Caulobacter_K31_
Phenylobacterium_zucineum_HLK1_
Hirschia_baltica_ATCC_49814_
Hyphomonas_neptunium_ATCC_15444_
Parvibaculum_lavamentivorans_DS1_
Nisaea_BAL199_
Candidatus_Puniceispirillum_marinum_IMCC1322_
Rhodospirillum_rubrum_S1_ATCC_11170_
Acetobacter_pasteurianus_IFO_328301_
Acetobacter_pomorum_DM001_
Acetobacter_tropicalis_NBRC_101654_
Gluconobacter_oxydans_621H_
Gluconacetobacter_diazotrophicus_PAl_5_DSM_5601_
Gluconacetobacter_SXCC1_
Acidiphilium_cryptum_JF5_
Acidiphilium_multivorum_AIU301_
Granulibacter_bethesdensis_CGDNIH1_
Azospirillum_B510_
Rhodocista_centenaria_SW_
Magnetospirillum_magnetotacticum_MS1_
Magnetospirillum_magneticum_AMB1_
Caedibacter_caryophilus_221_
Candidatus_Odyssella_thessalonicensis_L13_
Polysphondylium_pallidum_mitochondrion_
Dictyostelium_discoideum_mitochondrion_
Naegleria_gruberi_mitochondrion_
Acanthamoeba_castellanii_mitochondrion_
Hartmannella_vermiformis_mitochondrion_
Cyanidioschyzon_merolae_mitochondrion_
Chondrus_crispus_mitochondrion_
Chattonella_marina_mitochondrion_
Pilayella_littoralis_mitochondrion_
Ochromonas_danica_mitochondrion_
Saprolegnia_ferax_strain_ATCC_36051_mitochondrion_
Phytophthora_infestans_mitochondrion_
Glaucocystis_nostochinearum_mitochondrion_
Cyanophora_paradoxa_strain_CCMP_329_mitochondrion_
Malawimonas_jakobiformis_mitochondrion_
Reclinomonas_americana_mitochondrion_
Megaceros_aenigmaticus_mitochondrion_
Marchantia_polymorpha_mitochondrion_
Arabidopsis_thaliana_mitochondrion_
Chara_vulgaris_mitochondrion_
Chaetosphaeridium_globosum_mitochondrion_
Chlorokybus_atmophyticus_strain_SAG_4880_mitochondrion_
Nephroselmis_olivacea_mitochondrion_
Prototheca_wickerhamii_263-11_mitochondrion_
Ehrlichia_chaffeensis_Arkansas_
Ehrlichia_canis_Jake_
Ehrlichia_ruminantium_Gardel_
Anaplasma_centrale_str_Israel_
Anaplasma_marginale_Puerto_Rico_
Anaplasma_phagocytophilum_HZ_
Wolbachia_of_Culex_quinquefasciatus_JHB_
Wolbachia_of_Drosophila_melanogaster_
Wolbachia_of_Brugia_malayi_
Neorickettsia_sennetsu_Miyayama_
Neorickettsia_risticii_Illinois_
Rickettsia_canadensis_McKiel_
Rickettsia_felis_URRWXCal2_
Rickettsia_bellii_RML369C_
Rickettsia_typhi_Wilmington_
Orientia_tsutsugamushi_Ikeda_
Candidatus_Midichloria_mitochondrii_IricVA_
Candidatus_Pelagibacter_ubique_HTCC1002_
Candidatus_Pelagibacter_HTCC7211_
Pelagibacteraceae_HIMB5B_
Pelagibacteraceae_440_
Pelagibacteraceae_313_
Pelagibacteraceae_HIMB114_
Pelagibacteraceae_IMCC9063_
Pelagibacteraceae_HIMB59_
Magnetococcus_MC1_
Escherichia_coli_MG1655_
Xanthomonas_campestris_8004_
Ralstonia_solanacearum_GMI1000_
Thiobacillus_denitrificans_ATCC_25259_
Acidithiobacillus_ferrooxidans_ATCC_23270_
Mariprofundus_ferrooxydans_PV1_
Campylobacter_jejuni_ICDCCJ07001_
0.2

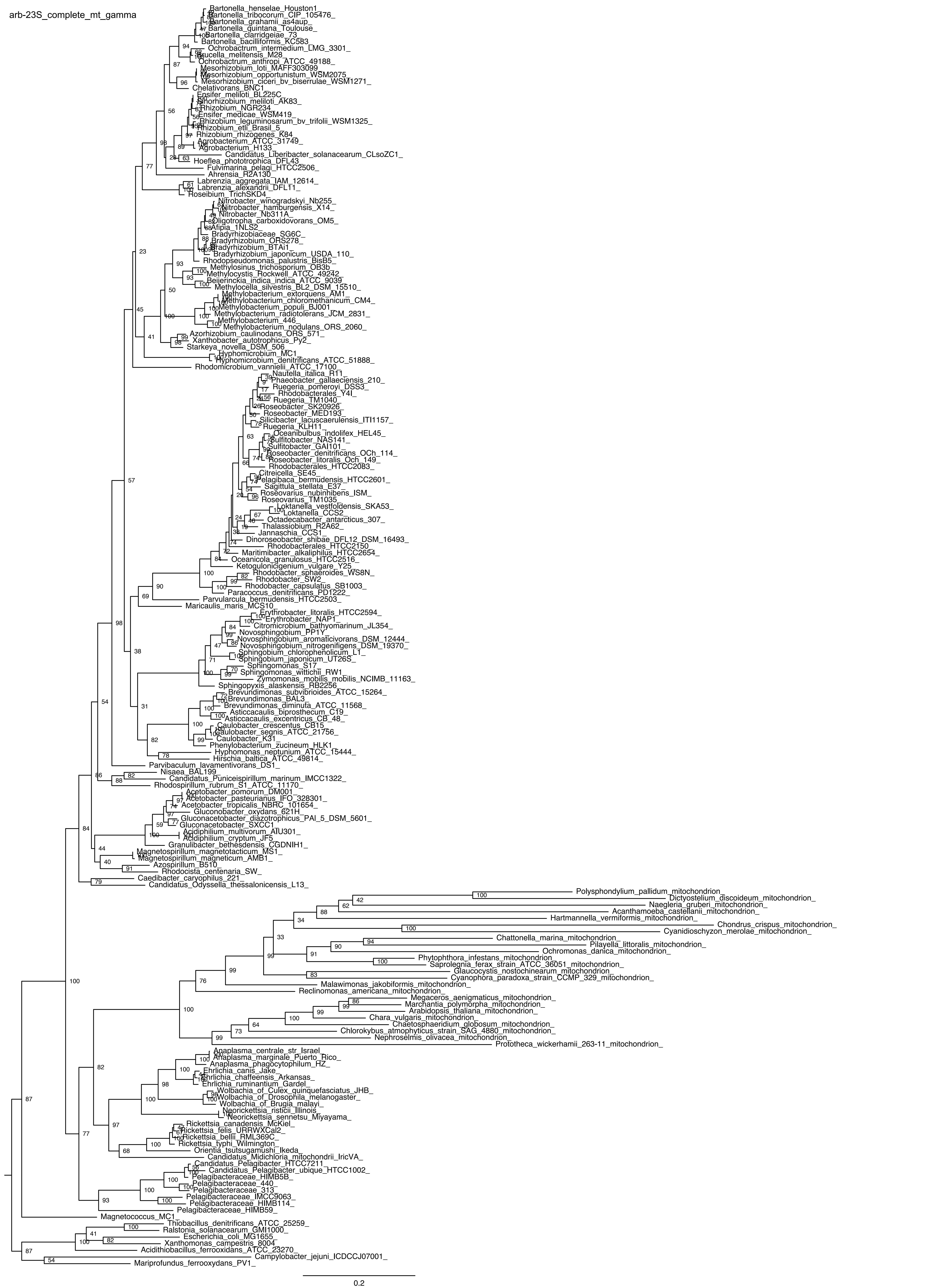
arb-23S_complete_mt_gamma
Bartonella_henselae_Houston1_
Bartonella_tribocorum_CIP_105476_
Bartonella_grahamii_as4aup_
Bartonella_quintana_Toulouse_
Bartonella_clarridgeiae_73_
Bartonella_bacilliformis_KC583_
Ochrobactrum_intermedium_LMG_3301_
Brucella_melitensis_M28_
Ochrobactrum_anthropi_ATCC_49188_
Mesorhizobium_loti_MAFF303099_
Mesorhizobium_opportunistum_WSM2075_
Mesorhizobium_ciceri_bv_biserrulae_WSM1271_
Chelativorans_BNC1_
Ensifer_meliloti_BL225C_
Sinorhizobium_meliloti_AK83_
Rhizobium_NGR234_
Ensifer_medicae_WSM419_
Rhizobium_leguminosarum_bv_trifolii_WSM1325_
Rhizobium_etli_Brasil_5_
Rhizobium_rhizogenes_K84_
Agrobacterium_ATCC_31749_
Agrobacterium_H133_
Candidatus_Liberibacter_solanacearum_CLsoZC1_
Hoeflea_phototrophica_DFL43_
Fulvimarina_pelagi_HTCC2506_
Ahrensia_R2A130_
Labrenzia_aggregata_IAM_12614_
Labrenzia_alexandrii_DFL11_
Roseibium_TrichSKD4_
Nitrobacter_winogradskyi_Nb255_
Nitrobacter_hamburgensis_X14_
Nitrobacter_Nb311A_
Oligotropha_carboxidovorans_OM5_
Afipia_1NLS2_
Bradyrhizobiaceae_SG6C_
Bradyrhizobium_ORS278_
Bradyrhizobium_BTAi1_
Bradyrhizobium_japonicum_USDA_110_
Rhodopseudomonas_palustris_BisB5_
Methylosinus_trichosporium_OB3b_
Methylocystis_Rockwell_ATCC_49242_
Beijerinckia_indica_indica_ATCC_9039_
Methylocella_silvestris_BL2_DSM_15510_
Methylobacterium_extorquens_AM1_
Methylobacterium_chloromethanicum_CM4_
Methylobacterium_populi_BJ001_
Methylobacterium_radiotolerans_JCM_2831_
Methylobacterium_446_
Methylobacterium_nodulans_ORS_2060_
Azorhizobium_caulinodans_ORS_571_
Xanthobacter_autotrophicus_Py2_
Starkeya_novella_DSM_506_
Hyphomicrobium_MC1_
Hyphomicrobium_denitrificans_ATCC_51888_
Rhodomicrobium_vannielii_ATCC_17100_
Nautella_italica_R11_
Phaeobacter_gallaeciensis_210_
Ruegeria_pomeroyi_DSS3_
Rhodobacterales_Y4I_
Ruegeria_TM1040_
Roseobacter_SK20926_
Roseobacter_MED193_
Silicibacter_lacuscaerulensis_ITI1157_
Ruegeria_KLH11_
Oceanibulbus_indolifex_HEL45_
Sulfitobacter_NAS141_
Sulfitobacter_GAI101_
Roseobacter_denitrificans_OCh_114_
Roseobacter_litoralis_Och_149_
Rhodobacterales_HTCC2083_
Citreicella_SE45_
Pelagibaca_bermudensis_HTCC2601_
Sagittula_stellata_E37_
Roseovarius_nubinhibens_ISM_
Roseovarius_TM1035_
Loktanella_vestfoldensis_SKA53_
Loktanella_CCS2_
Octadecabacter_antarcticus_307_
Thalassiobium_R2A62_
Jannaschia_CCS1_
Dinoroseobacter_shibae_DFL12_DSM_16493_
Rhodobacterales_HTCC2150_
Maritimibacter_alkaliphilus_HTCC2654_
Oceanicola_granulosus_HTCC2516_
Ketogulonicigenium_vulgare_Y25_
Rhodobacter_sphaeroides_WS8N_
Rhodobacter_SW2_
Rhodobacter_capsulatus_SB1003_
Paracoccus_denitrificans_PD1222_
Parvularcula_bermudensis_HTCC2503_
Maricaulis_maris_MCS10_
Erythrobacter_litoralis_HTCC2594_
Erythrobacter_NAP1_
Citromicrobium_bathyomarinum_JL354_
Novosphingobium_PP1Y_
Novosphingobium_aromaticivorans_DSM_12444_
Novosphingobium_nitrogenifigens_DSM_19370_
Sphingobium_chlorophenolicum_L1_
Sphingobium_japonicum_UT26S_
Sphingomonas_S17_
Sphingomonas_wittichii_RW1_
Zymomonas_mobilis_mobilis_NCIMB_11163_
Sphingopyxis_alaskensis_RB2256_
Brevundimonas_subvibrioides_ATCC_15264_
Brevundimonas_BAL3_
Brevundimonas_diminuta_ATCC_11568_
Asticcacaulis_biprosthecum_C19_
Asticcacaulis_excentricus_CB_48_
Caulobacter_crescentus_CB15_
Caulobacter_segnis_ATCC_21756_
Caulobacter_K31_
Phenylobacterium_zucineum_HLK1_
Hyphomonas_neptunium_ATCC_15444_
Hirschia_baltica_ATCC_49814_
Parvibaculum_lavamentivorans_DS1_
Nisaea_BAL199_
Candidatus_Puniceispirillum_marinum_IMCC1322_
Rhodospirillum_rubrum_S1_ATCC_11170_
Acetobacter_pomorum_DM001_
Acetobacter_pasteurianus_IFO_328301_
Acetobacter_tropicalis_NBRC_101654_
Gluconobacter_oxydans_621H_
Gluconacetobacter_diazotrophicus_PAl_5_DSM_5601_
Gluconacetobacter_SXCC1_
Acidiphilium_multivorum_AIU301_
Acidiphilium_cryptum_JF5_
Granulibacter_bethesdensis_CGDNIH1_
Magnetospirillum_magnetotacticum_MS1_
Magnetospirillum_magneticum_AMB1_
Azospirillum_B510_
Rhodocista_centenaria_SW_
Caedibacter_caryophilus_221_
Candidatus_Odyssella_thessalonicensis_L13_
Polysphondylium_pallidum_mitochondrion_
Dictyostelium_discoideum_mitochondrion_
Naegleria_gruberi_mitochondrion_
Acanthamoeba_castellanii_mitochondrion_
Hartmannella_vermiformis_mitochondrion_
Chondrus_crispus_mitochondrion_
Cyanidioschyzon_merolae_mitochondrion_
Chattonella_marina_mitochondrion_
Pilayella_littoralis_mitochondrion_
Ochromonas_danica_mitochondrion_
Phytophthora_infestans_mitochondrion_
Saprolegnia_ferax_strain_ATCC_36051_mitochondrion_
Glaucocystis_nostochinearum_mitochondrion_
Cyanophora_paradoxa_strain_CCMP_329_mitochondrion_
Malawimonas_jakobiformis_mitochondrion_
Reclinomonas_americana_mitochondrion_
Megaceros_aenigmaticus_mitochondrion_
Marchantia_polymorpha_mitochondrion_
Arabidopsis_thaliana_mitochondrion_
Chara_vulgaris_mitochondrion_
Chaetosphaeridium_globosum_mitochondrion_
Chlorokybus_atmophyticus_strain_SAG_4880_mitochondrion_
Nephroselmis_olivacea_mitochondrion_
Prototheca_wickerhamii_263-11_mitochondrion_
Anaplasma_centrale_str_Israel_
Anaplasma_marginale_Puerto_Rico_
Anaplasma_phagocytophilum_HZ_
Ehrlichia_canis_Jake_
Ehrlichia_chaffeensis_Arkansas_
Ehrlichia_ruminantium_Gardel_
Wolbachia_of_Culex_quinquefasciatus_JHB_
Wolbachia_of_Drosophila_melanogaster_
Wolbachia_of_Brugia_malayi_
Neorickettsia_risticii_Illinois_
Neorickettsia_sennetsu_Miyayama_
Rickettsia_canadensis_McKiel_
Rickettsia_felis_URRWXCal2_
Rickettsia_bellii_RML369C_
Rickettsia_typhi_Wilmington_
Orientia_tsutsugamushi_Ikeda_
Candidatus_Midichloria_mitochondrii_IricVA_
Candidatus_Pelagibacter_HTCC7211_
Candidatus_Pelagibacter_ubique_HTCC1002_
Pelagibacteraceae_HIMB5B_
Pelagibacteraceae_440_
Pelagibacteraceae_313_
Pelagibacteraceae_IMCC9063_
Pelagibacteraceae_HIMB114_
Pelagibacteraceae_HIMB59_
Magnetococcus_MC1_
Thiobacillus_denitrificans_ATCC_25259_
Ralstonia_solanacearum_GMI1000_
Escherichia_coli_MG1655_
Xanthomonas_campestris_8004_
Acidithiobacillus_ferrooxidans_ATCC_23270_
Campylobacter_jejuni_ICDCCJ07001_
Mariprofundus_ferrooxydans_PV1_
0.2

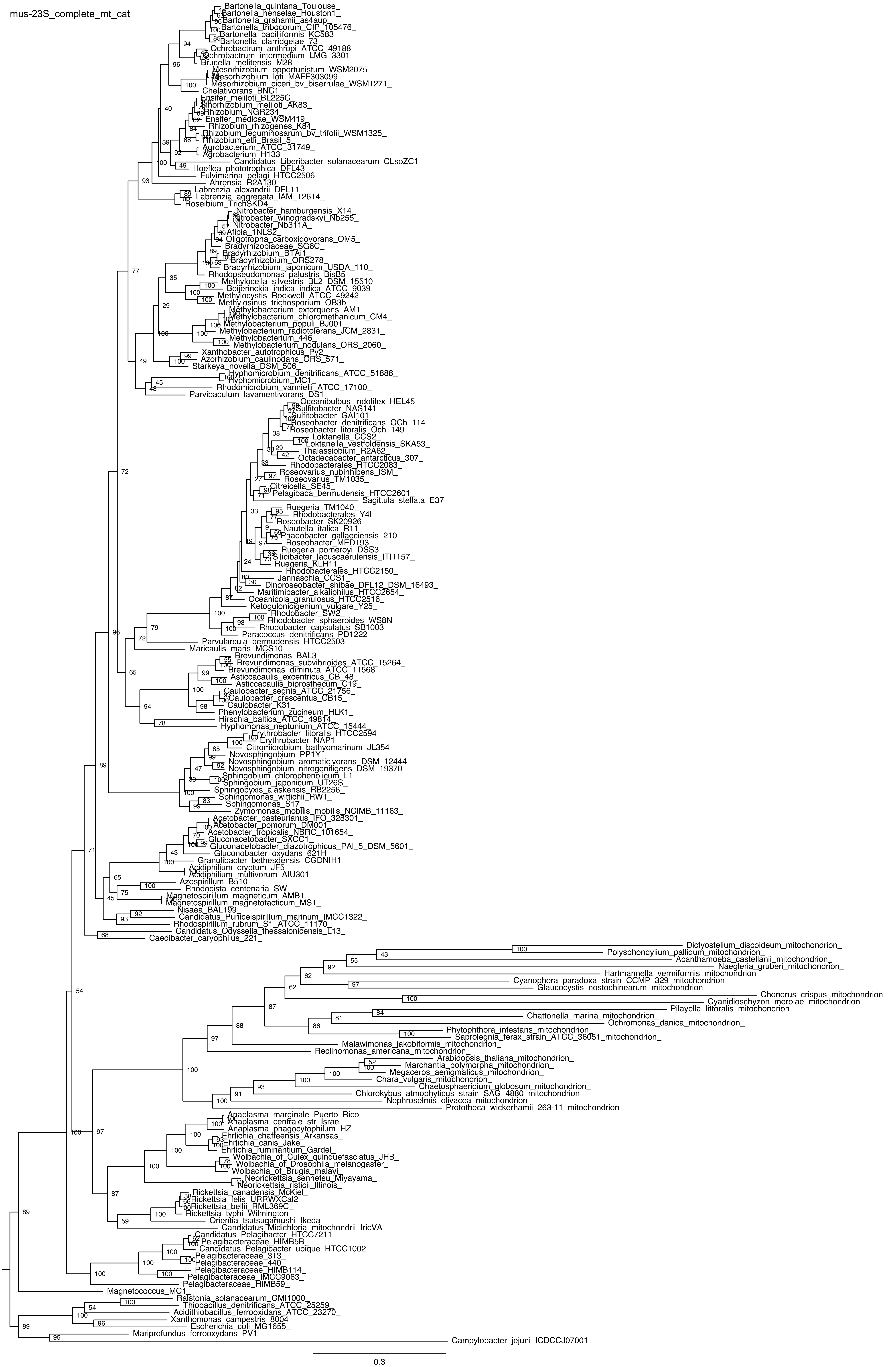
mus-23S_complete_mt_cat
Bartonella_quintana_Toulouse_
Bartonella_henselae_Houston1_
Bartonella_grahamii_as4aup_
Bartonella_tribocorum_CIP_105476_
Bartonella_bacilliformis_KC583_
Bartonella_clarridgeiae_73_
Ochrobactrum_anthropi_ATCC_49188_
Ochrobactrum_intermedium_LMG_3301_
Brucella_melitensis_M28_
Mesorhizobium_opportunistum_WSM2075_
Mesorhizobium_loti_MAFF303099_
Mesorhizobium_ciceri_bv_biserrulae_WSM1271_
Chelativorans_BNC1_
Ensifer_meliloti_BL225C_
Sinorhizobium_meliloti_AK83_
Rhizobium_NGR234_
Ensifer_medicae_WSM419_
Rhizobium_rhizogenes_K84_
Rhizobium_leguminosarum_bv_trifolii_WSM1325_
Rhizobium_etli_Brasil_5_
Agrobacterium_ATCC_31749_
Agrobacterium_H133_
Candidatus_Liberibacter_solanacearum_CLsoZC1_
Hoeflea_phototrophica_DFL43_
Fulvimarina_pelagi_HTCC2506_
Ahrensia_R2A130_
Labrenzia_alexandrii_DFL11_
Labrenzia_aggregata_IAM_12614_
Roseibium_TrichSKD4_
Nitrobacter_hamburgensis_X14_
Nitrobacter_winogradskyi_Nb255_
Nitrobacter_Nb311A_
Afipia_1NLS2_
Oligotropha_carboxidovorans_OM5_
Bradyrhizobiaceae_SG6C_
Bradyrhizobium_BTAi1_
Bradyrhizobium_ORS278_
Bradyrhizobium_japonicum_USDA_110_
Rhodopseudomonas_palustris_BisB5_
Methylocella_silvestris_BL2_DSM_15510_
Beijerinckia_indica_indica_ATCC_9039_
Methylocystis_Rockwell_ATCC_49242_
Methylosinus_trichosporium_OB3b_
Methylobacterium_extorquens_AM1_
Methylobacterium_chloromethanicum_CM4_
Methylobacterium_populi_BJ001_
Methylobacterium_radiotolerans_JCM_2831_
Methylobacterium_446_
Methylobacterium_nodulans_ORS_2060_
Xanthobacter_autotrophicus_Py2_
Azorhizobium_caulinodans_ORS_571_
Starkeya_novella_DSM_506_
Hyphomicrobium_denitrificans_ATCC_51888_
Hyphomicrobium_MC1_
Rhodomicrobium_vannielii_ATCC_17100_
Parvibaculum_lavamentivorans_DS1_
Oceanibulbus_indolifex_HEL45_
Sulfitobacter_NAS141_
Sulfitobacter_GAI101_
Roseobacter_denitrificans_OCh_114_
Roseobacter_litoralis_Och_149_
Loktanella_CCS2_
Loktanella_vestfoldensis_SKA53_
Thalassiobium_R2A62_
Octadecabacter_antarcticus_307_
Rhodobacterales_HTCC2083_
Roseovarius_nubinhibens_ISM_
Roseovarius_TM1035_
Citreicella_SE45_
Pelagibaca_bermudensis_HTCC2601_
Sagittula_stellata_E37_
Ruegeria_TM1040_
Rhodobacterales_Y4I_
Roseobacter_SK20926_
Nautella_italica_R11_
Phaeobacter_gallaeciensis_210_
Roseobacter_MED193_
Ruegeria_pomeroyi_DSS3_
Silicibacter_lacuscaerulensis_ITI1157_
Ruegeria_KLH11_
Rhodobacterales_HTCC2150_
Jannaschia_CCS1_
Dinoroseobacter_shibae_DFL12_DSM_16493_
Maritimibacter_alkaliphilus_HTCC2654_
Oceanicola_granulosus_HTCC2516_
Ketogulonicigenium_vulgare_Y25_
Rhodobacter_SW2_
Rhodobacter_sphaeroides_WS8N_
Rhodobacter_capsulatus_SB1003_
Paracoccus_denitrificans_PD1222_
Parvularcula_bermudensis_HTCC2503_
Maricaulis_maris_MCS10_
Brevundimonas_BAL3_
Brevundimonas_subvibrioides_ATCC_15264_
Brevundimonas_diminuta_ATCC_11568_
Asticcacaulis_excentricus_CB_48_
Asticcacaulis_biprosthecum_C19_
Caulobacter_segnis_ATCC_21756_
Caulobacter_crescentus_CB15_
Caulobacter_K31_
Phenylobacterium_zucineum_HLK1_
Hirschia_baltica_ATCC_49814_
Hyphomonas_neptunium_ATCC_15444_
Erythrobacter_litoralis_HTCC2594_
Erythrobacter_NAP1_
Citromicrobium_bathyomarinum_JL354_
Novosphingobium_PP1Y_
Novosphingobium_aromaticivorans_DSM_12444_
Novosphingobium_nitrogenifigens_DSM_19370_
Sphingobium_chlorophenolicum_L1_
Sphingobium_japonicum_UT26S_
Sphingopyxis_alaskensis_RB2256_
Sphingomonas_wittichii_RW1_
Sphingomonas_S17_
Zymomonas_mobilis_mobilis_NCIMB_11163_
Acetobacter_pasteurianus_IFO_328301_
Acetobacter_pomorum_DM001_
Acetobacter_tropicalis_NBRC_101654_
Gluconacetobacter_SXCC1_
Gluconacetobacter_diazotrophicus_PAl_5_DSM_5601_
Gluconobacter_oxydans_621H_
Granulibacter_bethesdensis_CGDNIH1_
Acidiphilium_cryptum_JF5_
Acidiphilium_multivorum_AIU301_
Azospirillum_B510_
Rhodocista_centenaria_SW_
Magnetospirillum_magneticum_AMB1_
Magnetospirillum_magnetotacticum_MS1_
Nisaea_BAL199_
Candidatus_Puniceispirillum_marinum_IMCC1322_
Rhodospirillum_rubrum_S1_ATCC_11170_
Candidatus_Odyssella_thessalonicensis_L13_
Caedibacter_caryophilus_221_
Dictyostelium_discoideum_mitochondrion_
Polysphondylium_pallidum_mitochondrion_
Acanthamoeba_castellanii_mitochondrion_
Naegleria_gruberi_mitochondrion_
Hartmannella_vermiformis_mitochondrion_
Cyanophora_paradoxa_strain_CCMP_329_mitochondrion_
Glaucocystis_nostochinearum_mitochondrion_
Chondrus_crispus_mitochondrion_
Cyanidioschyzon_merolae_mitochondrion_
Pilayella_littoralis_mitochondrion_
Chattonella_marina_mitochondrion_
Ochromonas_danica_mitochondrion_
Phytophthora_infestans_mitochondrion_
Saprolegnia_ferax_strain_ATCC_36051_mitochondrion_
Malawimonas_jakobiformis_mitochondrion_
Reclinomonas_americana_mitochondrion_
Arabidopsis_thaliana_mitochondrion_
Marchantia_polymorpha_mitochondrion_
Megaceros_aenigmaticus_mitochondrion_
Chara_vulgaris_mitochondrion_
Chaetosphaeridium_globosum_mitochondrion_
Chlorokybus_atmophyticus_strain_SAG_4880_mitochondrion_
Nephroselmis_olivacea_mitochondrion_
Prototheca_wickerhamii_263-11_mitochondrion_
Anaplasma_marginale_Puerto_Rico_
Anaplasma_centrale_str_Israel_
Anaplasma_phagocytophilum_HZ_
Ehrlichia_chaffeensis_Arkansas_
Ehrlichia_canis_Jake_
Ehrlichia_ruminantium_Gardel_
Wolbachia_of_Culex_quinquefasciatus_JHB_
Wolbachia_of_Drosophila_melanogaster_
Wolbachia_of_Brugia_malayi_
Neorickettsia_sennetsu_Miyayama_
Neorickettsia_risticii_Illinois_
Rickettsia_canadensis_McKiel_
Rickettsia_felis_URRWXCal2_
Rickettsia_bellii_RML369C_
Rickettsia_typhi_Wilmington_
Orientia_tsutsugamushi_Ikeda_
Candidatus_Midichloria_mitochondrii_IricVA_
Candidatus_Pelagibacter_HTCC7211_
Pelagibacteraceae_HIMB5B_
Candidatus_Pelagibacter_ubique_HTCC1002_
Pelagibacteraceae_313_
Pelagibacteraceae_440_
Pelagibacteraceae_HIMB114_
Pelagibacteraceae_IMCC9063_
Pelagibacteraceae_HIMB59_
Magnetococcus_MC1_
Ralstonia_solanacearum_GMI1000_
Thiobacillus_denitrificans_ATCC_25259_
Acidithiobacillus_ferrooxidans_ATCC_23270_
Xanthomonas_campestris_8004_
Escherichia_coli_MG1655_
Mariprofundus_ferrooxydans_PV1_
Campylobacter_jejuni_ICDCCJ07001_
0.3

mus-23S_complete_mt_gamma

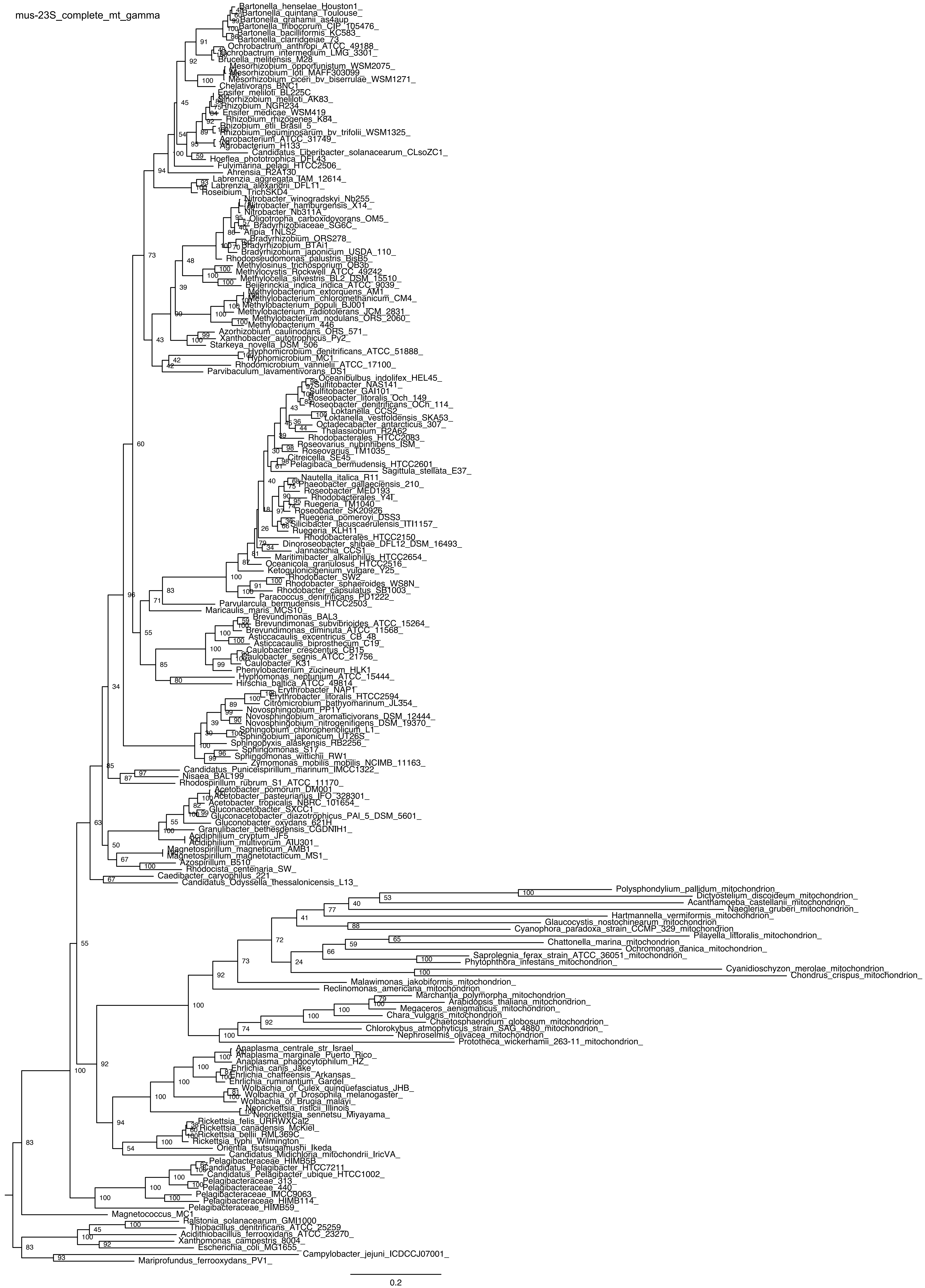

Supplement: Figure S11 — 16S and 23S rRNA gene trees for the complete dataset, with and without mitochondria. (PDF) [file pone.0083383.s011.pdf]
