# Supplementary material for: New rRNA Gene-Based Phylogenies of the Alphaproteobacteria Provide Perspective on Major Groups, Mitochondrial Ancestry and Phylogenetic Instability
Source: PLoS One. 2013 Dec 11;8(12):e83383. doi: 10.1371/journal.pone.0083383 (PMC3859672; doi:10.1371/journal.pone.0083383)
Supplement: Figure S13 — A. RY-coded complete dataset trees, with and without mitochondria. B. MK-coded complete dataset trees, with and without mitochondria. (PDF) [file pone.0083383.s013.pdf]

arb-bij_complete_RY_cat

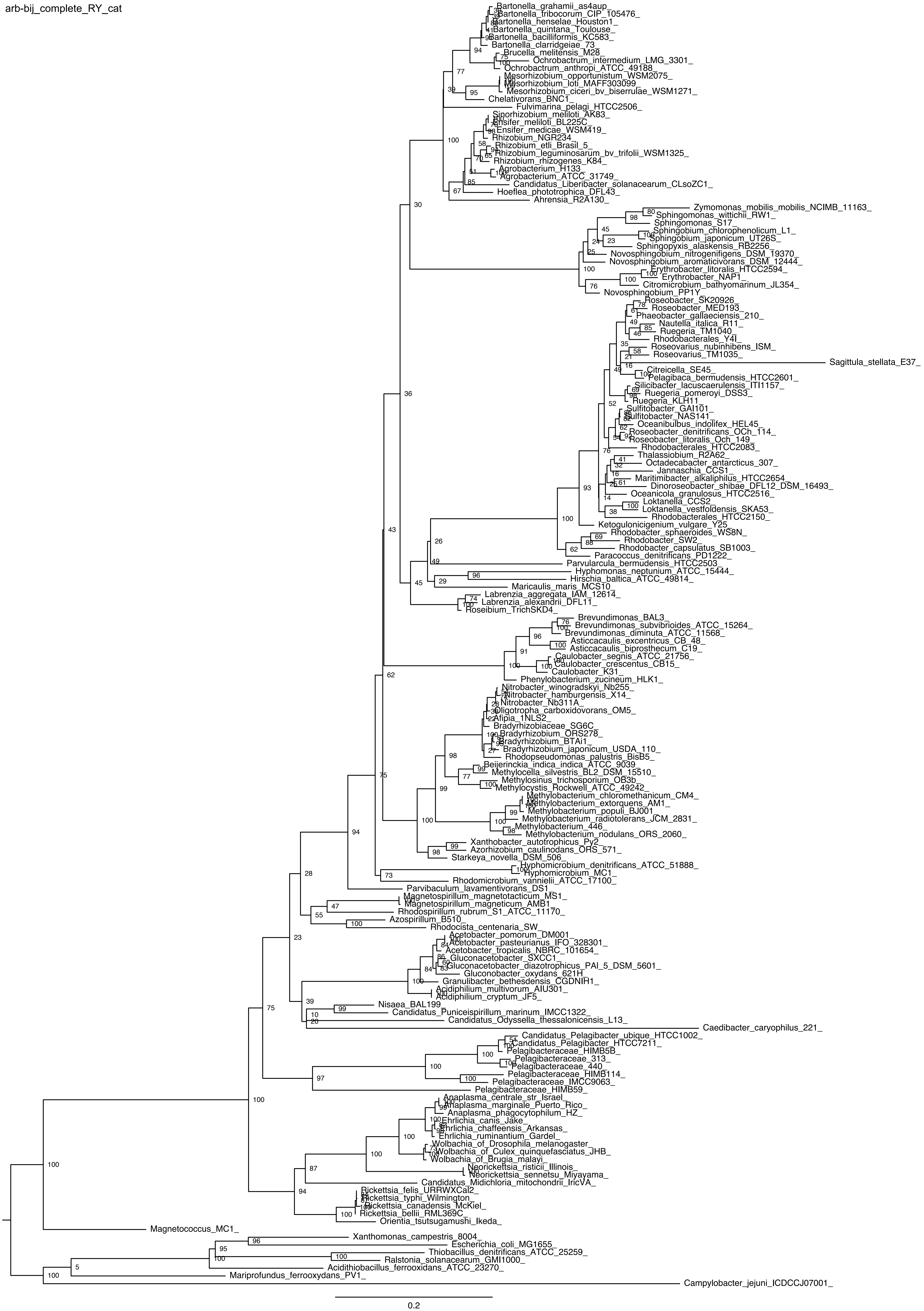

arb-bij_complete_RY_gamma

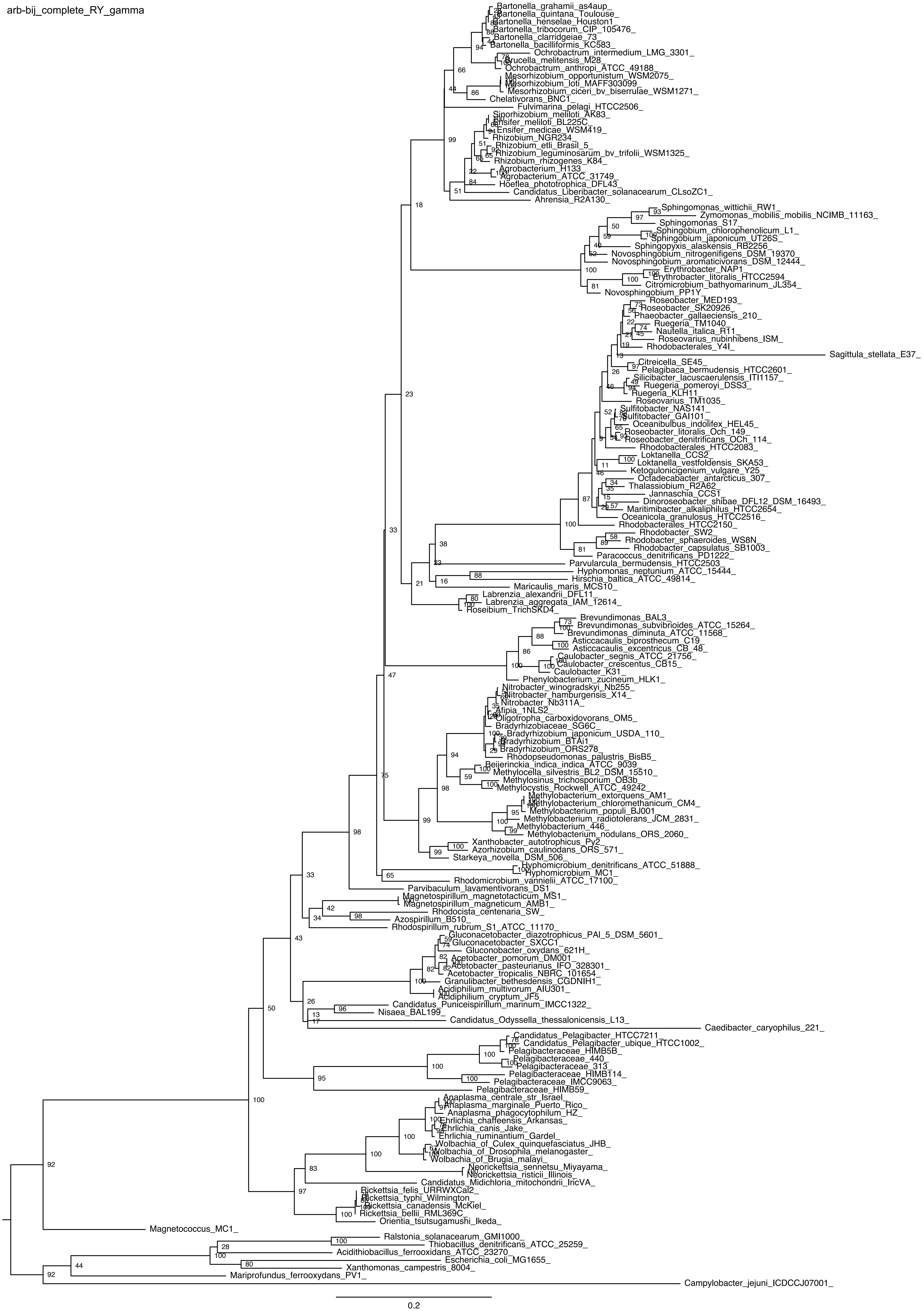

mus-bij_complete_RY_cat

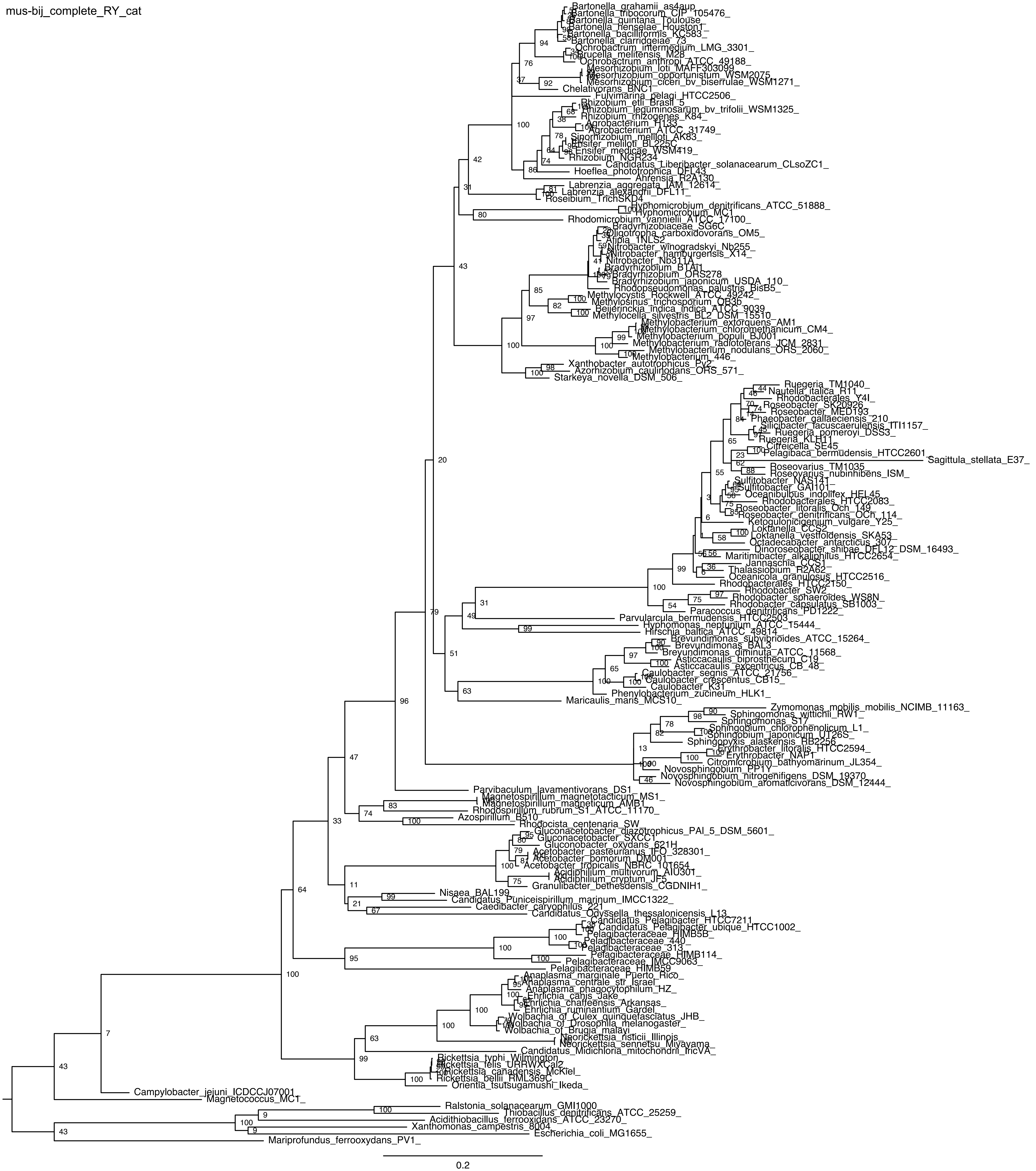

mus-bij_complete_RY_gamma

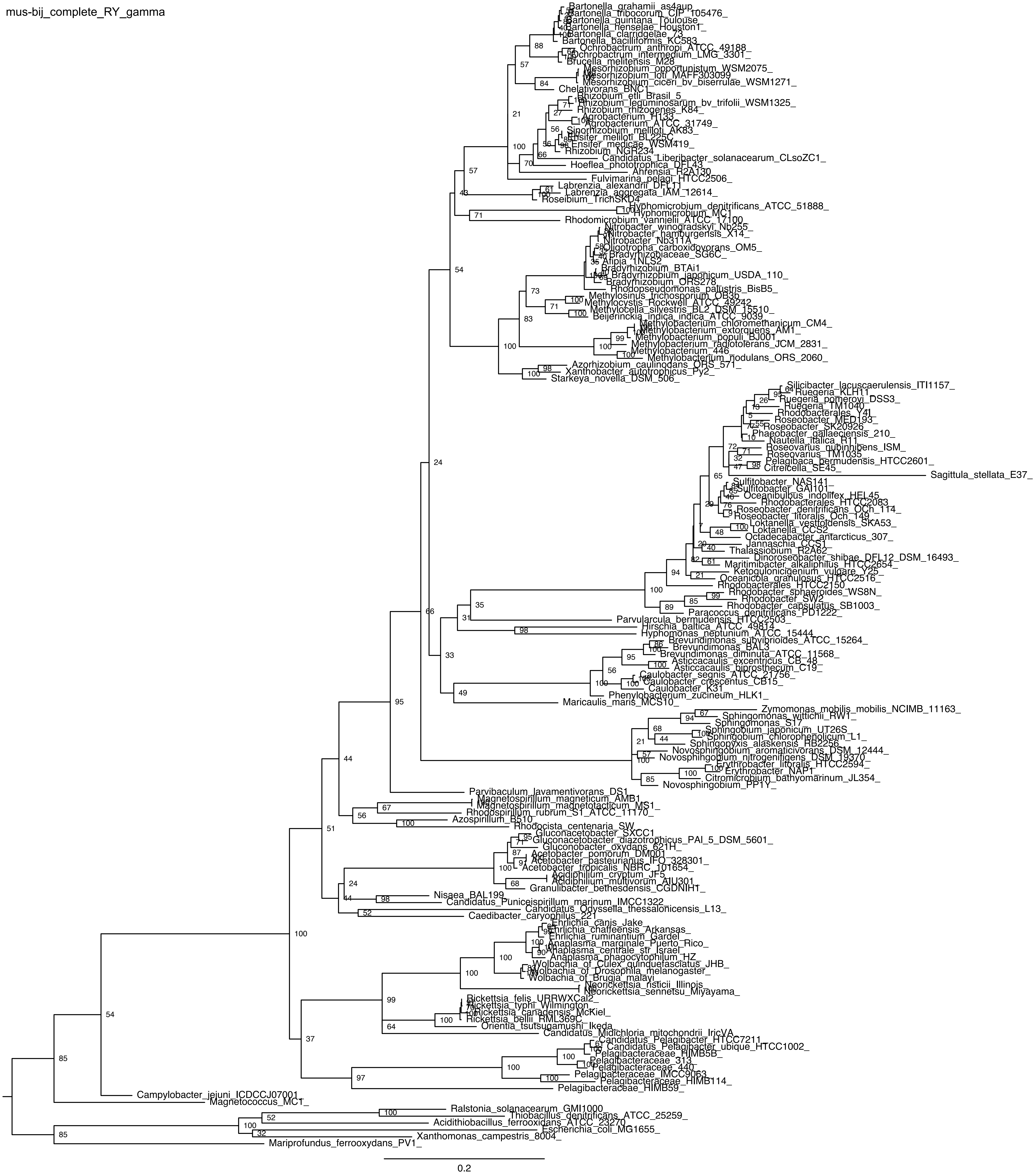

arb-bij_complete_mt_RY_cat

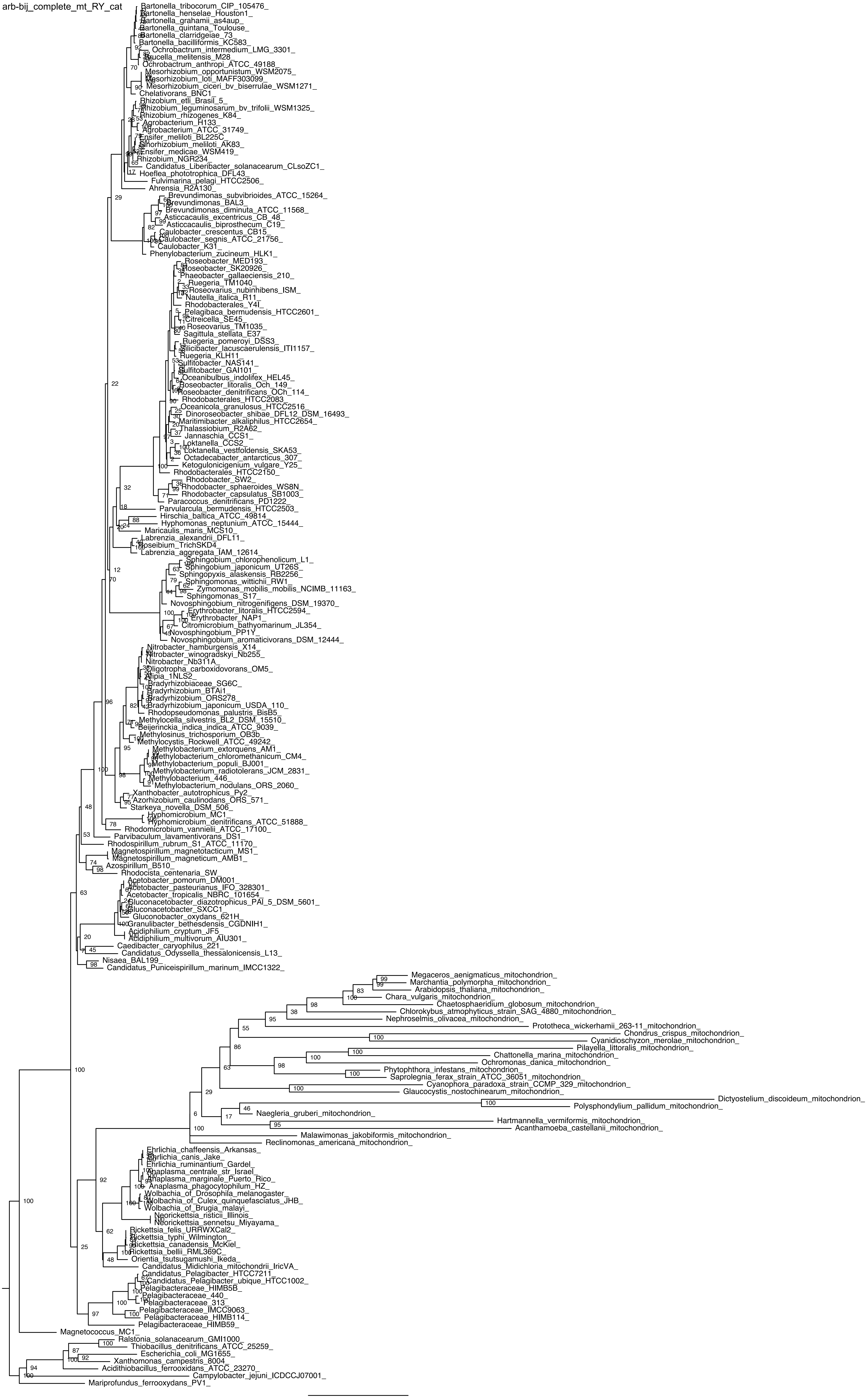

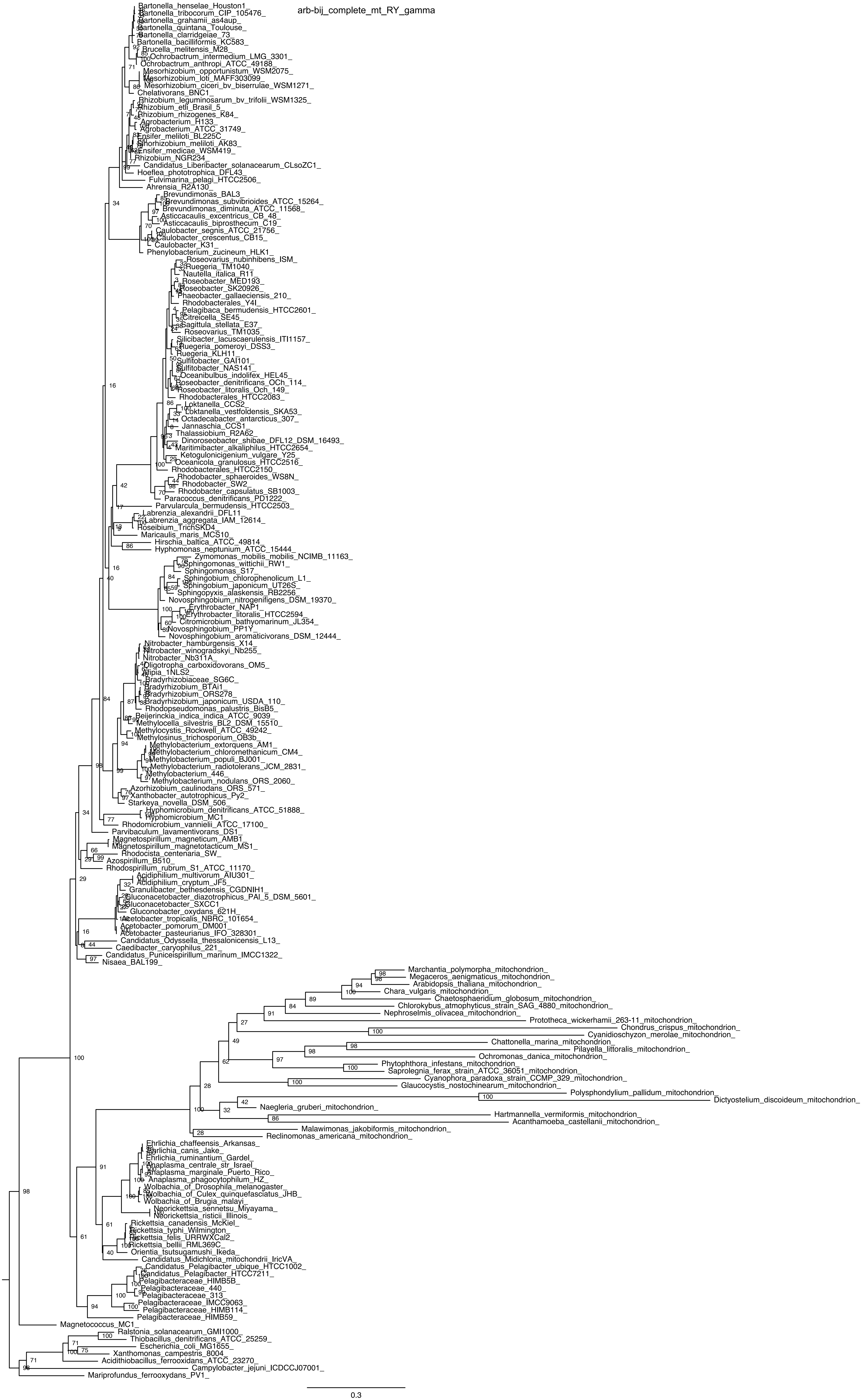
arb-bij_complete_mt_RY_gamma
Bartonella_henselae_Houston1_
Bartonella_tribocorum_CIP_105476_
Bartonella_grahamii_as4aup_
Bartonella_quintana_Toulouse_
Bartonella_clarridgeiae_73_
Bartonella_bacilliformis_KC583_
Brucella_melitensis_M28_
Ochrobactrum_intermedium_LMG_3301_
Ochrobactrum_anthropi_ATCC_49188_
Mesorhizobium_opportunistum_WSM2075_
Mesorhizobium_loti_MAFF303099_
Mesorhizobium_ciceri_bv_biserrulae_WSM1271_
Chelativorans_BNC1_
Rhizobium_leguminosarum_bv_trifolii_WSM1325_
Rhizobium_etli_Brasil_5_
Rhizobium_rhizogenes_K84_
Agrobacterium_H133_
Agrobacterium_ATCC_31749_
Ensifer_meliloti_BL225C_
Sinorhizobium_meliloti_AK83_
Ensifer_medicae_WSM419_
Rhizobium_NGR234_
Candidatus_Liberibacter_solanacearum_CLsoZC1_
Hoeflea_phototrophica_DFL43_
Fulvimarina_pelagi_HTCC2506_
Ahrensia_R2A130_
Brevundimonas_BAL3_
Brevundimonas_subvibrioides_ATCC_15264_
Brevundimonas_diminuta_ATCC_11568_
Asticcacaulis_excentricus_CB_48_
Asticcacaulis_biprosthecum_C19_
Caulobacter_segnis_ATCC_21756_
Caulobacter_crescentus_CB15_
Caulobacter_K31_
Phenylobacterium_zucineum_HLK1_
Roseovarius_nubinhibens_ISM_
Ruegeria_TM1040_
Nautella_italica_R11_
Roseobacter_MED193_
Roseobacter_SK20926_
Phaeobacter_gallaeciensis_210_
Rhodobacterales_Y4I_
Pelagibaca_bermudensis_HTCC2601_
Citreicella_SE45_
Sagittula_stellata_E37_
Roseovarius_TM1035_
Silicibacter_lacuscaerulensis_ITI1157_
Ruegeria_pomeroyi_DSS3_
Ruegeria_KLH11_
Sulfitobacter_GAI101_
Sulfitobacter_NAS141_
Oceanibulbus_indolifex_HEL45_
Roseobacter_denitrificans_OCh_114_
Roseobacter_litoralis_Och_149_
Rhodobacterales_HTCC2083_
Loktanella_CCS2_
Loktanella_vestfoldensis_SKA53_
Octadecabacter_antarcticus_307_
Jannaschia_CCS1_
Thalassiobium_R2A62_
Dinoroseobacter_shibae_DFL12_DSM_16493_
Maritimibacter_alkaliphilus_HTCC2654_
Ketogulonicigenium_vulgare_Y25_
Oceanicola_granulosus_HTCC2516_
Rhodobacterales_HTCC2150_
Rhodobacter_sphaeroides_WS8N_
Rhodobacter_SW2_
Rhodobacter_capsulatus_SB1003_
Paracoccus_denitrificans_PD1222_
Parvularcula_bermudensis_HTCC2503_
Labrenzia_alexandrii_DFL11_
Labrenzia_aggregata_IAM_12614_
Roseibium_TrichSKD4_
Maricaulis_maris_MCS10_
Hirschia_baltica_ATCC_49814_
Hyphomonas_neptunium_ATCC_15444_
Zymomonas_mobilis_mobilis_NCIMB_11163_
Sphingomonas_wittichii_RW1_
Sphingomonas_S17_
Sphingobium_chlorophenolicum_L1_
Sphingobium_japonicum_UT26S_
Sphingopyxis_alaskensis_RB2256_
Novosphingobium_nitrogenifigens_DSM_19370_
Erythrobacter_NAP1_
Erythrobacter_litoralis_HTCC2594_
Citromicrobium_bathyomarinum_JL354_
Novosphingobium_PP1Y_
Novosphingobium_aromaticivorans_DSM_12444_
Nitrobacter_hamburgensis_X14_
Nitrobacter_winogradskyi_Nb255_
Nitrobacter_Nb311A_
Oligotropha_carboxidovorans_OM5_
Afipia_1NLS2_
Bradyrhizobiaceae_SG6C_
Bradyrhizobium_BTAi1_
Bradyrhizobium_ORS278_
Bradyrhizobium_japonicum_USDA_110_
Rhodopseudomonas_palustris_BisB5_
Beijerinckia_indica_indica_ATCC_9039_
Methylocella_silvestris_BL2_DSM_15510_
Methylocystis_Rockwell_ATCC_49242_
Methylosinus_trichosporium_OB3b_
Methylobacterium_extorquens_AM1_
Methylobacterium_chloromethanicum_CM4_
Methylobacterium_populi_BJ001_
Methylobacterium_radiotolerans_JCM_2831_
Methylobacterium_446_
Methylobacterium_nodulans_ORS_2060_
Azorhizobium_caulinodans_ORS_571_
Xanthobacter_autotrophicus_Py2_
Starkeya_novella_DSM_506_
Hyphomicrobium_denitrificans_ATCC_51888_
Hyphomicrobium_MC1_
Rhodomicrobium_vannielii_ATCC_17100_
Parvibaculum_lavamentivorans_DS1_
Magnetospirillum_magneticum_AMB1_
Magnetospirillum_magnetotacticum_MS1_
Rhodocista_centenaria_SW_
Azospirillum_B510_
Rhodospirillum_rubrum_S1_ATCC_11170_
Acidiphilium_multivorum_AIU301_
Acidiphilium_cryptum_JF5_
Granulibacter_bethesdensis_CGDNIH1_
Gluconacetobacter_diazotrophicus_PAI_5_DSM_5601_
Gluconacetobacter_SXCC1_
Gluconobacter_oxydans_621H_
Acetobacter_tropicalis_NBRC_101654_
Acetobacter_pomorum_DM001_
Acetobacter_pasteurianus_IFO_328301_
Candidatus_Odyssella_thessalonicensis_L13_
Caedibacter_caryophilus_221_
Candidatus_Puniceispirillum_marinum_IMCC1322_
Nisaea_BAL199_
Marchantia_polymorpha_mitochondrion_
Megaceros_aenigmaticus_mitochondrion_
Arabidopsis_thaliana_mitochondrion_
Chara_vulgaris_mitochondrion_
Chaetosphaeridium_globosum_mitochondrion_
Chlorokybus_atmophyticus_strain_SAG_4880_mitochondrion_
Nephroselmis_olivacea_mitochondrion_
Prototheca_wickerhamii_263-11_mitochondrion_
Chondrus_crispus_mitochondrion_
Cyanidioschyzon_merolae_mitochondrion_
Chattonella_marina_mitochondrion_
Pilayella_littoralis_mitochondrion_
Ochromonas_danica_mitochondrion_
Phytophthora_infestans_mitochondrion_
Saprolegnia_ferax_strain_ATCC_36051_mitochondrion_
Cyanophora_paradoxa_strain_CCMP_329_mitochondrion_
Glaucocystis_nostochinearum_mitochondrion_
Polysphondylium_pallidum_mitochondrion_
Dictyostelium_discoideum_mitochondrion_
Naegleria_gruberi_mitochondrion_
Hartmannella_vermiformis_mitochondrion_
Acanthamoeba_castellanii_mitochondrion_
Malawimonas_jakobiformis_mitochondrion_
Reclinomonas_americana_mitochondrion_
Ehrlichia_chaffeensis_Arkansas_
Ehrlichia_canis_Jake_
Ehrlichia_ruminantium_Gardel_
Anaplasma_centrale_str_Israel_
Anaplasma_marginale_Puerto_Rico_
Anaplasma_phagocytophilum_HZ_
Wolbachia_of_Drosophila_melanogaster_
Wolbachia_of_Culex_quinquefasciatus_JHB_
Wolbachia_of_Brugia_malayi_
Neorickettsia_sennetsu_Miyayama_
Neorickettsia_risticii_Illinois_
Rickettsia_canadensis_McKiel_
Rickettsia_typhi_Wilmington_
Rickettsia_felis_URRWXCal2_
Rickettsia_bellii_RML369C_
Orientia_tsutsugamushi_Ikeda_
Candidatus_Midichloria_mitochondrii_IricVA_
Candidatus_Pelagibacter_ubique_HTCC1002_
Candidatus_Pelagibacter_HTCC7211_
Pelagibacteraceae_HIMB5B_
Pelagibacteraceae_440_
Pelagibacteraceae_313_
Pelagibacteraceae_IMCC9063_
Pelagibacteraceae_HIMB114_
Pelagibacteraceae_HIMB59_
Magnetococcus_MC1_
Ralstonia_solanacearum_GMI1000_
Thiobacillus_denitrificans_ATCC_25259_
Escherichia_coli_MG1655_
Xanthomonas_campestris_8004_
Acidithiobacillus_ferrooxidans_ATCC_23270_
Campylobacter_jejuni_ICDCCJ07001_
Mariprofundus_ferrooxydans_PV1_
0.3

mus-bij_complete_mt_RY_cat

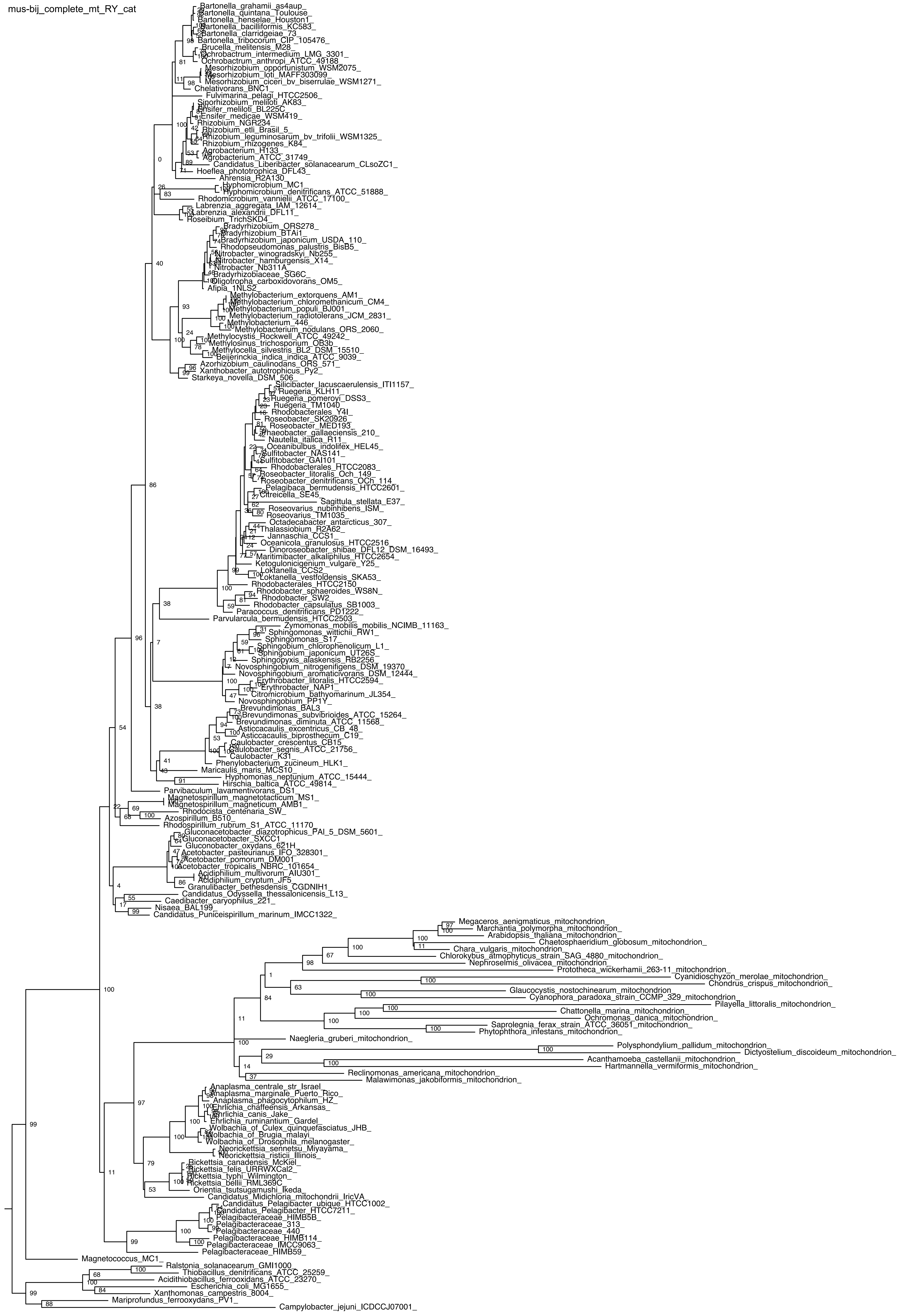

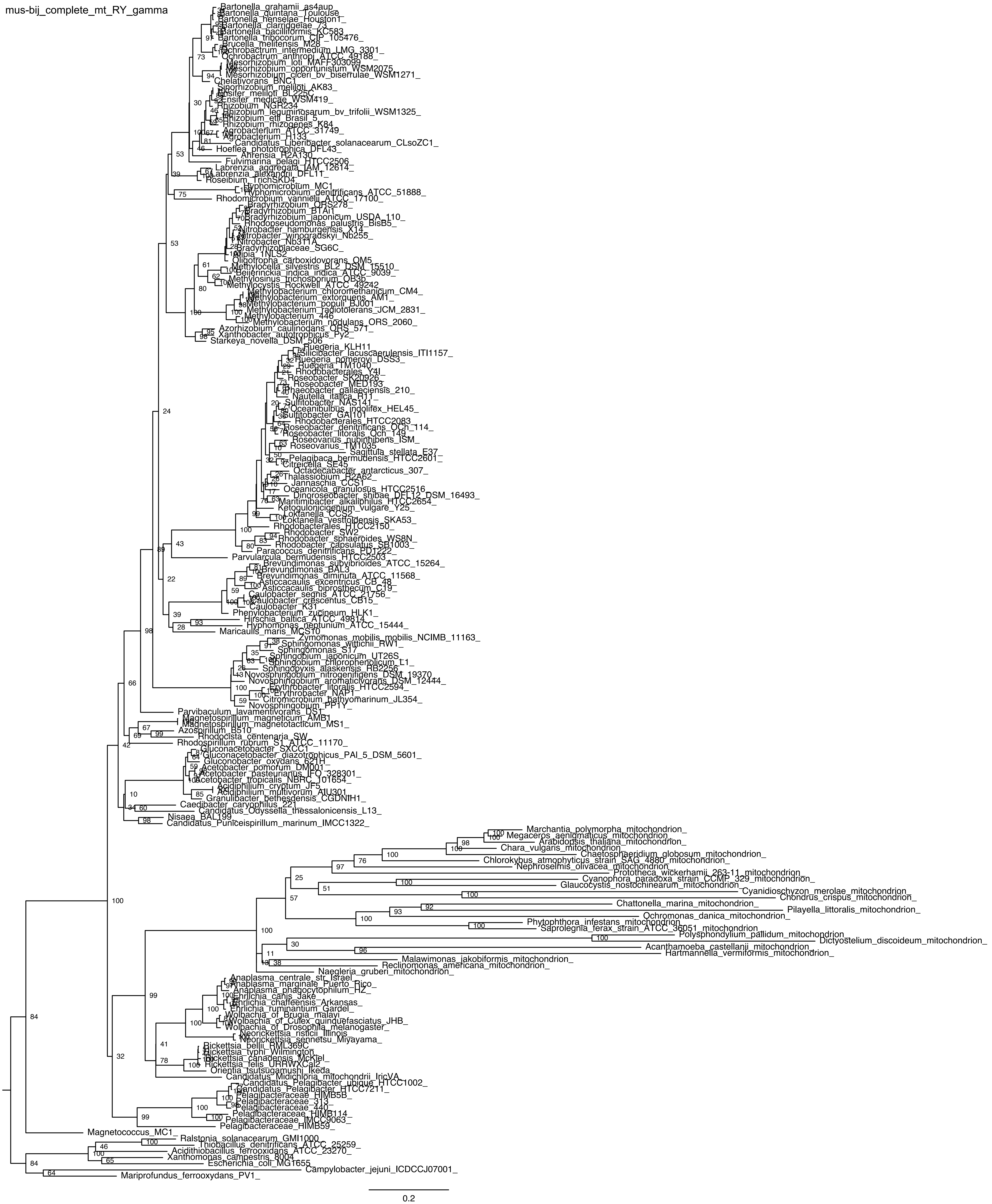
mus-bij_complete_mt_RY_gamma
Bartonella_grahamii_as4aup
Bartonella_quintana_Toulouse
Bartonella_henselae_Houston1_
Bartonella_clarridgeiae_73
Bartonella_bacilliformis_KC583
Bartonella_tribocorum_CIP_105476_
Brucella_melitensis_M28
Ochrobactrum_intermedium_LMG_3301_
Ochrobactrum_anthropi_ATCC_49188_
Mesorhizobium_loti_MAFF303099
Mesorhizobium_opportunistum_WSM2075
Mesorhizobium_ciceri_bv_biserrulae_WSM1271_
Chelativorans_BNC1
Sinorhizobium_meliloti_AK83_
Ensifer_meliloti_BL225C
Ensifer_medicae_WSM419_
Rhizobium_NGR234
Rhizobium_leguminosarum_bv_trifolii_WSM1325_
Rhizobium_etli_Brasil_5
Rhizobium_rhizogenes_K84
Agrobacterium_ATCC_31749
Agrobacterium_H133
Candidatus_Liberibacter_solanacearum_CLsoZC1_
Hoeflea_phototrophica_DFL43_
Ahrensia_R2A130
Fulvimarina_pelagi_HTCC2506
Labrenzia_aggregata_IAM_12614_
Labrenzia_alexandrii_DFL11_
Roseibium_TrichSKD4
Hyphomicrobium_MC1
Hyphomicrobium_denitrificans_ATCC_51888_
Rhodomicrobium_vannielii_ATCC_17100_
Bradyrhizobium_ORS278
Bradyrhizobium_BTAi1
Bradyrhizobium_japonicum_USDA_110_
Rhodopseudomonas_palustris_BisB5_
Nitrobacter_hamburgensis_X14
Nitrobacter_winogradskyi_Nb255
Nitrobacter_Nb311A
Bradyrhizobiaceae_SG6C
Afipia_1NLS2
Oligotropha_carboxidovorans_OM5
Methylocella_silvestris_BL2_DSM_15510_
Beijerinckia_indica_indica_ATCC_9039_
Methylosinus_trichosporium_OB3b
Methylocystis_Rockwell_ATCC_49242
Methylobacterium_chloromethanicum_CM4_
Methylobacterium_extorquens_AM1
Methylobacterium_populi_BJ001
Methylobacterium_radiotolerans_JCM_2831_
Methylobacterium_446
Methylobacterium_nodulans_ORS_2060_
Azorhizobium_caulinodans_ORS_571
Xanthobacter_autotrophicus_Py2
Starkeya_novella_DSM_506
Ruegeria_KLH11
Silicibacter_lacuscaerulensis_ITI1157_
Ruegeria_pomeroyi_DSS3_
Ruegeria_TM1040
Rhodobacterales_Y4I
Roseobacter_SK20926
Roseobacter_MED193
Phaeobacter_gallaeciensis_210_
Nautella_italica_R11
Sulfitobacter_NAS141
Oceanibulbus_indolifex_HEL45_
Sulfitobacter_GAI101
Rhodobacterales_HTCC2083
Roseobacter_denitrificans_OCh_114_
Roseobacter_litoralis_Och_149
Roseovarius_nubinhibens_ISM
Roseovarius_TM1035
Sagittula_stellata_E37
Pelagibaca_bermudensis_HTCC2601_
Citreicella_SE45
Octadecabacter_antarcticus_307
Thalassiobium_R2A62
Jannaschia_CCS1
Oceanicola_granulosus_HTCC2516
Dinoroseobacter_shibae_DFL12_DSM_16493_
Maritimibacter_alkaliphilus_HTCC2654_
Ketogulonicigenium_vulgare_Y25
Loktanella_CCS2
Loktanella_vestfoldensis_SKA53_
Rhodobacterales_HTCC2150_
Rhodobacter_SW2
Rhodobacter_sphaeroides_WS8N
Rhodobacter_capsulatus_SB1003_
Paracoccus_denitrificans_PD1222
Parvularcula_bermudensis_HTCC2503
Brevundimonas_subvibrioides_ATCC_15264_
Brevundimonas_BAL3
Brevundimonas_diminuta_ATCC_11568_
Asticcacaulis_excentricus_CB_48
Asticcacaulis_biprosthecum_C19
Caulobacter_segnis_ATCC_21756
Caulobacter_crescentus_CB15
Caulobacter_K31
Phenylobacterium_zucineum_HLK1_
Hirschia_baltica_ATCC_49814
Hyphomonas_neptunium_ATCC_15444_
Maricaulis_maris_MCS10
Zymomonas_mobilis_mobilis_NCIMB_11163_
Sphingomonas_wittichii_RW1
Sphingomonas_S17
Sphingobium_japonicum_UT26S
Sphingobium_chlorophenolicum_L1
Sphingopyxis_alaskensis_RB2256
Novosphingobium_nitrogenifigens_DSM_19370
Novosphingobium_aromaticivorans_DSM_12444_
Erythrobacter_litoralis_HTCC2594
Erythrobacter_NAP1
Citromicrobium_bathyomarinum_JL354_
Novosphingobium_PP1Y
Parvibaculum_lavamentivorans_DS1
Magnetospirillum_magneticum_AMB1
Magnetospirillum_magnetotacticum_MS1_
Azospirillum_B510
Rhodocista_centenaria_SW
Rhodospirillum_rubrum_S1_ATCC_11170_
Gluconacetobacter_SXCC1
Gluconacetobacter_diazotrophicus_PAl_5_DSM_5601_
Gluconobacter_oxydans_621H
Acetobacter_pomorum_DM001
Acetobacter_pasteurianus_IFO_328301_
Acetobacter_tropicalis_NBRC_101654_
Acidiphilium_cryptum_JF5
Acidiphilium_multivorum_AIU301
Granulibacter_bethesdensis_CGDNIH1_
Caedibacter_caryophilus_221
Candidatus_Odyssella_thessalonicensis_L13_
Nisaea_BAL199
Candidatus_Puniceispirillum_marinum_IMCC1322_
Marchantia_polymorpha_mitochondrion_
Megaceros_aenigmaticus_mitochondrion
Arabidopsis_thaliana_mitochondrion_
Chara_vulgaris_mitochondrion
Chaetosphaeridium_globosum_mitochondrion_
Chlorokybus_atmophyticus_strain_SAG_4880_mitochondrion_
Nephroselmis_olivacea_mitochondrion
Prototheca_wickerhamii_263-11_mitochondrion
Cyanophora_paradoxa_strain_CCMP_329_mitochondrion_
Glaucocystis_nostochinearum_mitochondrion
Cyanidioschyzon_merolae_mitochondrion
Chondrus_crispus_mitochondrion_
Chattonella_marina_mitochondrion_
Pilayella_littoralis_mitochondrion_
Ochromonas_danica_mitochondrion_
Phytophthora_infestans_mitochondrion
Saprolegnia_ferax_strain_ATCC_36051_mitochondrion
Polysphondylium_pallidum_mitochondrion
Dictyostelium_discoideum_mitochondrion_
Acanthamoeba_castellanii_mitochondrion
Hartmannella_vermiformis_mitochondrion_
Malawimonas_jakobiformis_mitochondrion_
Reclinomonas_americana_mitochondrion_
Naegleria_gruberi_mitochondrion_
Anaplasma_centrale_str_Israel
Anaplasma_marginale_Puerto_Rico
Anaplasma_phagocytophilum_HZ
Ehrlichia_canis_Jake
Ehrlichia_chaffeensis_Arkansas_
Ehrlichia_ruminantium_Gardel
Wolbachia_of_Brugia_malayi
Wolbachia_of_Culex_quinquefasciatus_JHB_
Wolbachia_of_Drosophila_melanogaster_
Neorickettsia_risticii_Illinois
Neorickettsia_sennetsu_Miyayama_
Rickettsia_bellii_RML369C
Rickettsia_typhi_Wilmington
Rickettsia_canadensis_McKiel
Rickettsia_felis_URRWXCal2
Orientia_tsutsugamushi_Ikeda
Candidatus_Midichloria_mitochondrii_IricVA
Candidatus_Pelagibacter_ubique_HTCC1002_
Candidatus_Pelagibacter_HTCC7211
Pelagibacteraceae_HIMB5B
Pelagibacteraceae_313
Pelagibacteraceae_440
Pelagibacteraceae_HIMB114
Pelagibacteraceae_IMCC9063
Pelagibacteraceae_HIMB59
Magnetococcus_MC1
Ralstonia_solanacearum_GMI1000
Thiobacillus_denitrificans_ATCC_25259
Acidithiobacillus_ferrooxidans_ATCC_23270
Xanthomonas_campestris_8004
Escherichia_coli_MG1655
Campylobacter_jejuni_ICDCCJ07001_
Mariprofundus_ferrooxydans_PV1_
0.2

Arb bij complete MK cat

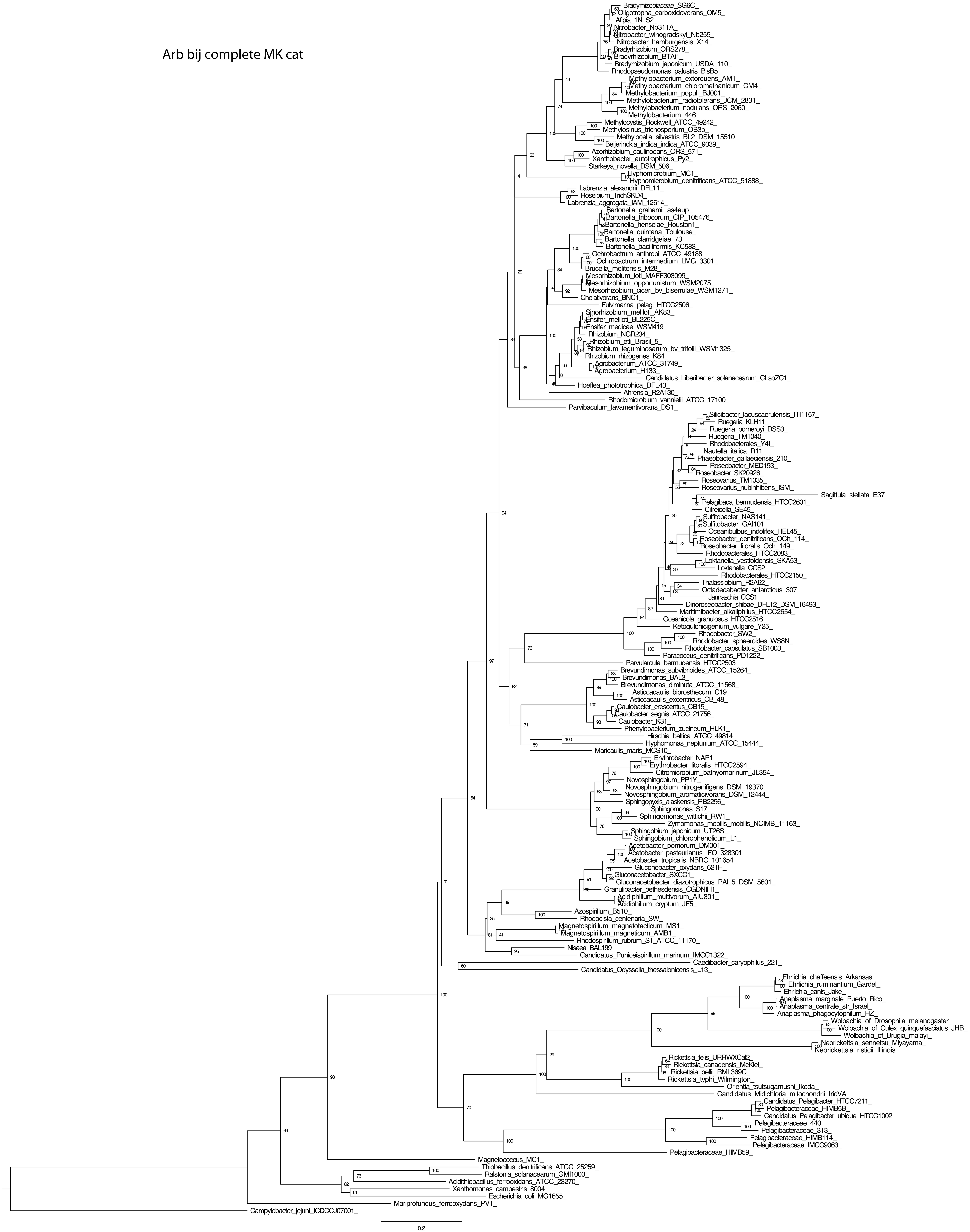

Mus bij bacteria MK gamma

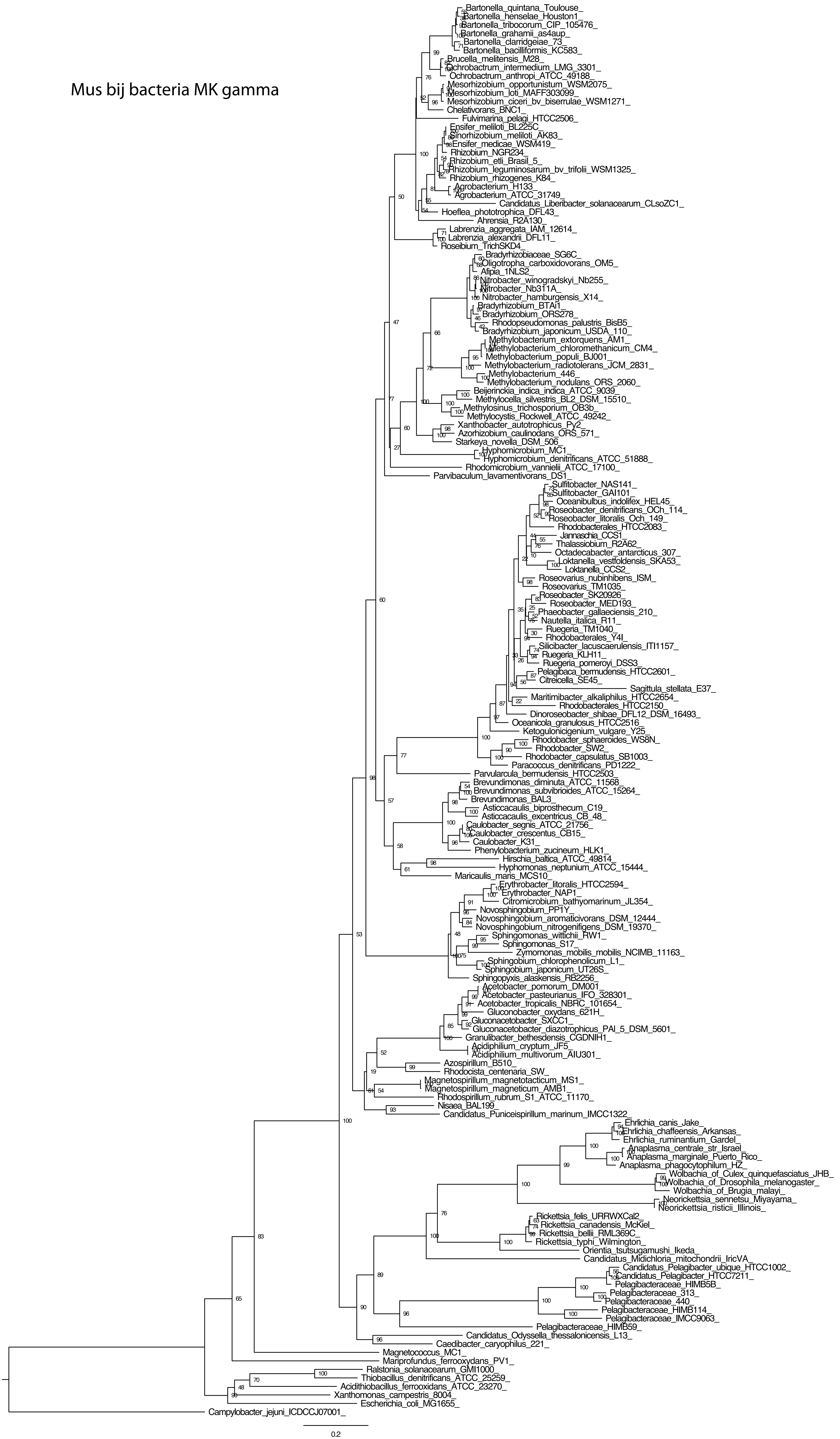

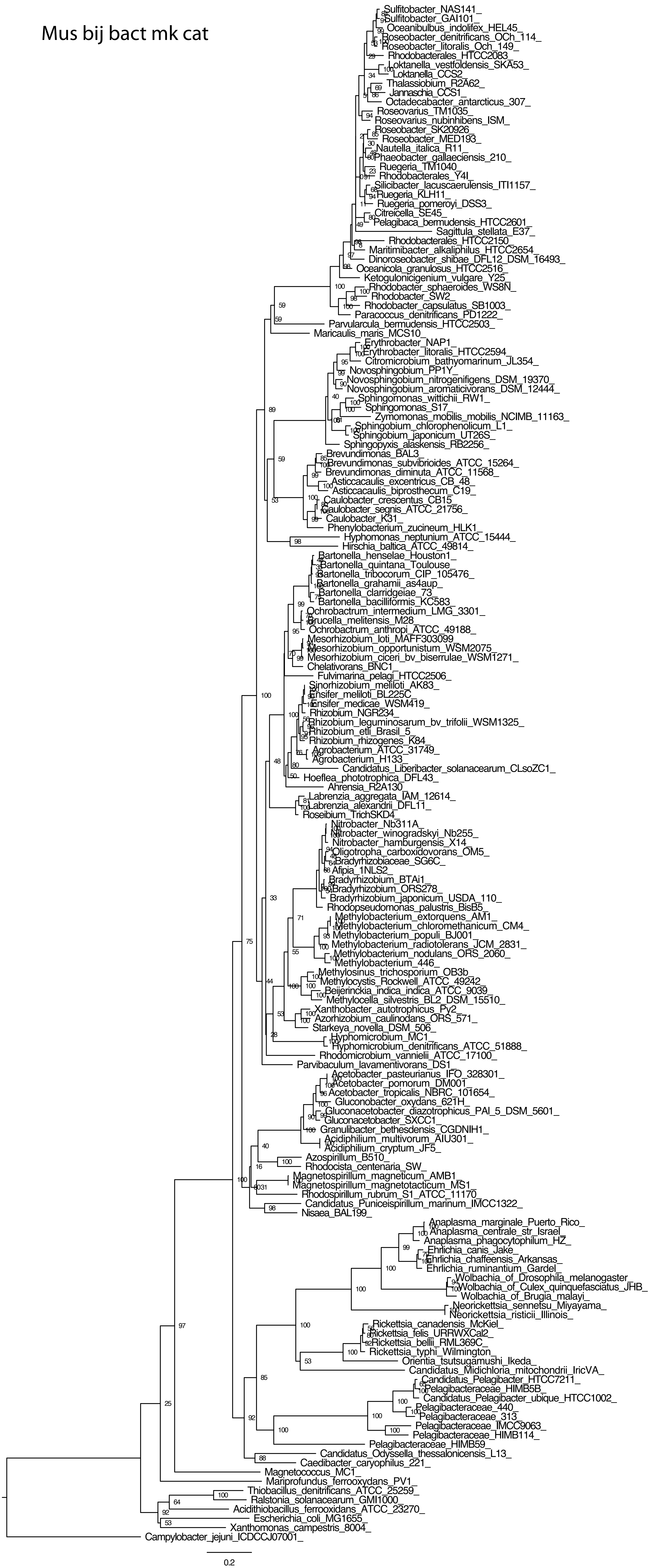
Mus bij bact mk cat
Sulfitobacter_NAS141_
Sulfitobacter_GAI101_
Oceanibulbus_indolifex_HEL45_
Roseobacter_denitrificans_OCh_114_
Roseobacter_litoralis_Och_149_
Rhodobacterales_HTCC2083_
Loktanella_vestfoldensis_SKA53_
Loktanella_CCS2_
Thalassiobium_R2A62_
Jannaschia_CCS1_
Octadecabacter_antarcticus_307_
Roseovarius_TM1035_
Roseovarius_nubinhibens_ISM_
Roseobacter_SK20926_
Roseobacter_MED193_
Nautella_italica_R11_
Phaeobacter_gallaeciensis_210_
Ruegeria_TM1040_
Rhodobacterales_Y4I_
Silicibacter_lacuscaerulensis_ITI1157_
Ruegeria_KLH11_
Ruegeria_pomeroyi_DSS3_
Citreicella_SE45_
Pelagibaca_bermudensis_HTCC2601_
Sagittula_stellata_E37_
Rhodobacterales_HTCC2150_
Maritimibacter_alkaliphilus_HTCC2654_
Dinoroseobacter_shibae_DFL12_DSM_16493_
Oceanicola_granulosus_HTCC2516_
Ketogulonicigenium_vulgare_Y25_
Rhodobacter_sphaeroides_WS8N_
Rhodobacter_SW2_
Rhodobacter_capsulatus_SB1003_
Paracoccus_denitrificans_PD1222_
Parvularcula_bermudensis_HTCC2503_
Maricaulis_maris_MCS10_
Erythrobacter_NAP1_
Erythrobacter_litoralis_HTCC2594_
Citromicrobium_bathyomarinum_JL354_
Novosphingobium_PP1Y_
Novosphingobium_nitrogenifigens_DSM_19370_
Novosphingobium_aromaticivorans_DSM_12444_
Sphingomonas_wittichii_RW1_
Sphingomonas_S17_
Zymomonas_mobilis_mobilis_NCIMB_11163_
Sphingobium_chlorophenolicum_L1_
Sphingobium_japonicum_UT26S_
Sphingopyxis_alaskensis_RB2256_
Brevundimonas_BAL3_
Brevundimonas_subvibrioides_ATCC_15264_
Brevundimonas_diminuta_ATCC_11568_
Asticcacaulis_excentricus_CB_48_
Asticcacaulis_biprosthecum_C19_
Caulobacter_crescentus_CB15_
Caulobacter_segnis_ATCC_21756_
Caulobacter_K31_
Phenylobacterium_zucineum_HLK1_
Hyphomonas_neptunium_ATCC_15444_
Hirschia_baltica_ATCC_49814_
Bartonella_henselae_Houston1_
Bartonella_quintana_Toulouse_
Bartonella_tribocorum_CIP_105476_
Bartonella_grahamii_as4aup_
Bartonella_clarridgeiae_73_
Bartonella_bacilliformis_KC583_
Ochrobactrum_intermedium_LMG_3301_
Brucella_melitensis_M28_
Ochrobactrum_anthropi_ATCC_49188_
Mesorhizobium_loti_MAFF303099_
Mesorhizobium_opportunistum_WSM2075_
Mesorhizobium_ciceri_bv_biserrulae_WSM1271_
Chelativorans_BNC1_
Fulvimarina_pelagi_HTCC2506_
Sinorhizobium_meliloti_AK83_
Ensifer_meliloti_BL225C_
Ensifer_medicae_WSM419_
Rhizobium_NGR234_
Rhizobium_leguminosarum_bv_trifolii_WSM1325_
Rhizobium_etli_Brasil_5_
Rhizobium_rhizogenes_K84_
Agrobacterium_ATCC_31749_
Agrobacterium_H133_
Candidatus_Liberibacter_solanacearum_CLsoZC1_
Hoeflea_phototrophica_DFL43_
Ahrensia_R2A130_
Labrenzia_aggregata_IAM_12614_
Labrenzia_alexandrii_DFL11_
Roseibium_TrichSKD4_
Nitrobacter_Nb311A_
Nitrobacter_winogradskyi_Nb255_
Nitrobacter_hamburgensis_X14_
Oligotropha_carboxidovorans_OM5_
Bradyrhizobiaceae_SG6C_
Afipia_1NLS2_
Bradyrhizobium_BTAi1_
Bradyrhizobium_ORS278_
Bradyrhizobium_japonicum_USDA_110_
Rhodopseudomonas_palustris_BisB5_
Methylobacterium_extorquens_AM1_
Methylobacterium_chloromethanicum_CM4_
Methylobacterium_populi_BJ001_
Methylobacterium_radiotolerans_JCM_2831_
Methylobacterium_nodulans_ORS_2060_
Methylobacterium_446_
Methylosinus_trichosporium_OB3b_
Methylocystis_Rockwell_ATCC_49242_
Beijerinckia_indica_indica_ATCC_9039_
Methylocella_silvestris_BL2_DSM_15510_
Xanthobacter_autotrophicus_Py2_
Azorhizobium_caulinodans_ORS_571_
Starkeya_novella_DSM_506_
Hyphomicrobium_MC1_
Hyphomicrobium_denitrificans_ATCC_51888_
Rhodomicrobium_vannielii_ATCC_17100_
Parvibaculum_lavamentivorans_DS1_
Acetobacter_pasteurianus_IFO_328301_
Acetobacter_pomorum_DM001_
Acetobacter_tropicalis_NBRC_101654_
Gluconobacter_oxydans_621H_
Gluconacetobacter_diazotrophicus_PAl_5_DSM_5601_
Gluconacetobacter_SXCC1_
Granulibacter_bethesdensis_CGDNIH1_
Acidiphilium_multivorum_AIU301_
Acidiphilium_cryptum_JF5_
Azospirillum_B510_
Rhodocista_centenaria_SW_
Magnetospirillum_magneticum_AMB1_
Magnetospirillum_magnetotacticum_MS1_
Rhodospirillum_rubrum_S1_ATCC_11170_
Candidatus_Puniceispirillum_marinum_IMCC1322_
Nisaea_BAL199_
Anaplasma_marginale_Puerto_Rico_
Anaplasma_centrale_str_Israel_
Anaplasma_phagocytophilum_HZ_
Ehrlichia_canis_Jake_
Ehrlichia_chaffeensis_Arkansas_
Ehrlichia_ruminantium_Gardel_
Wolbachia_of_Drosophila_melanogaster_
Wolbachia_of_Culex_quinquefasciatus_JHB_
Wolbachia_of_Brugia_malayi_
Neorickettsia_sennetsu_Miyayama_
Neorickettsia_risticii_Illinois_
Rickettsia_canadensis_McKiel_
Rickettsia_felis_URRWXCal2_
Rickettsia_bellii_RML369C_
Rickettsia_typhi_Wilmington_
Orientia_tsutsugamushi_Ikeda_
Candidatus_Midichloria_mitochondrii_IricVA_
Candidatus_Pelagibacter_HTCC7211_
Pelagibacteraceae_HIMB5B_
Candidatus_Pelagibacter_ubique_HTCC1002_
Pelagibacteraceae_440_
Pelagibacteraceae_313_
Pelagibacteraceae_IMCC9063_
Pelagibacteraceae_HIMB114_
Pelagibacteraceae_HIMB59_
Candidatus_Odyssella_thessalonicensis_L13_
Caedibacter_caryophilus_221_
Magnetococcus_MC1_
Mariprofundus_ferrooxydans_PV1_
Thiobacillus_denitrificans_ATCC_25259_
Ralstonia_solanacearum_GMI1000_
Acidithiobacillus_ferrooxidans_ATCC_23270_
Escherichia_coli_MG1655_
Xanthomonas_campestris_8004_
Campylobacter_jejuni_ICDCCJ07001_
0.2

Arb bij complete MK gamma

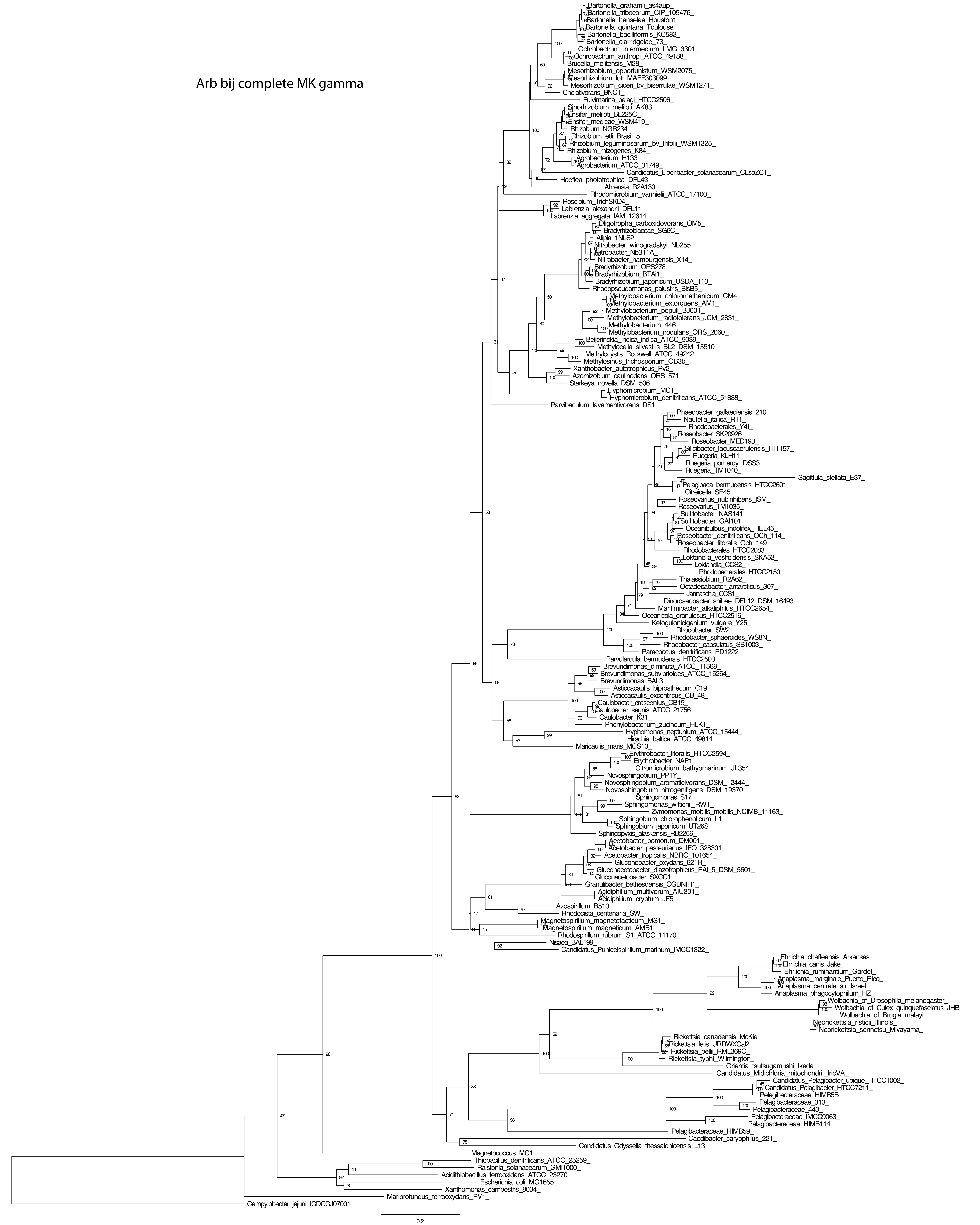

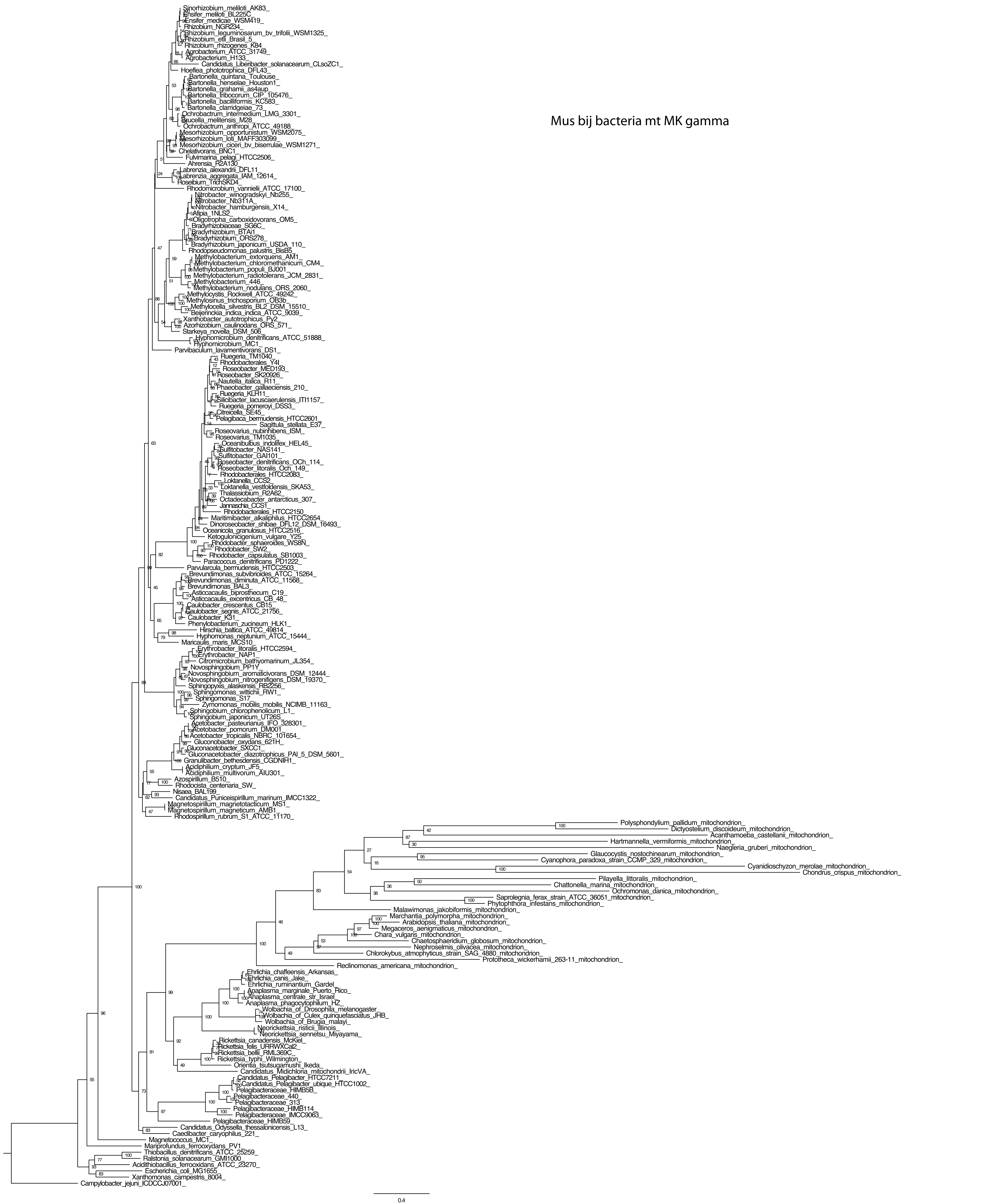
Mus bij bacteria mt MK gamma
Sinorhizobium_meliloti_AK83_
Ensifer_meliloti_BL225C_
Ensifer_medicae_WSM419_
Rhizobium_NGR234_
Rhizobium_leguminosarum_bv_trifolii_WSM1325_
Rhizobium_etli_Brasil_5_
Rhizobium_rhizogenes_K84_
Agrobacterium_ATCC_31749_
Agrobacterium_H133_
Candidatus_Liberibacter_solanacearum_CLsoZC1_
Hoeflea_phototrophica_DFL43_
Bartonella_quintana_Toulouse_
Bartonella_henselae_Houston1_
Bartonella_grahamii_as4aup_
Bartonella_tribocorum_CIP_105476_
Bartonella_bacilliformis_KC583_
Bartonella_clarridgeiae_73_
Ochrobactrum_intermedium_LMG_3301_
Brucella_melitensis_M28_
Ochrobactrum_anthropi_ATCC_49188_
Mesorhizobium_opportunistum_WSM2075_
Mesorhizobium_loti_MAFF303099_
Mesorhizobium_ciceri_bv_biserrulae_WSM1271_
Chelativorans_BNC1_
Fulvimarina_pelagi_HTCC2506_
Ahrensia_R2A130_
Labrenzia_alexandrii_DFL11_
Labrenzia_aggregata_IAM_12614_
Roseibium_TrichSKD4_
Rhodomicrobium_vannielii_ATCC_17100_
Nitrobacter_winogradskyi_Nb255_
Nitrobacter_Nb311A_
Nitrobacter_hamburgensis_X14_
Afipia_1NLS2_
Oligotropha_carboxidovorans_OM5_
Bradyrhizobiaceae_SG6C_
Bradyrhizobium_BTAi1_
Bradyrhizobium_ORS278_
Bradyrhizobium_japonicum_USDA_110_
Rhodopseudomonas_palustris_BisB5_
Methylobacterium_extorquens_AM1_
Methylobacterium_chloromethanicum_CM4_
Methylobacterium_populi_BJ001_
Methylobacterium_radiotolerans_JCM_2831_
Methylobacterium_446_
Methylobacterium_nodulans_ORS_2060_
Methylocystis_Rockwell_ATCC_49242_
Methylosinus_trichosporium_OB3b_
Methylocella_silvestris_BL2_DSM_15510_
Beijerinckia_indica_indica_ATCC_9039_
Xanthobacter_autotrophicus_Py2_
Azorhizobium_caulinodans_ORS_571_
Starkeya_novella_DSM_506_
Hyphomicrobium_denitrificans_ATCC_51888_
Hyphomicrobium_MC1_
Parvibaculum_lavamentivorans_DS1_
Ruegeria_TM1040_
Rhodobacterales_Y4I_
Roseobacter_MED193_
Roseobacter_SK20926_
Nautella_italica_R11_
Phaeobacter_gallaeciensis_210_
Ruegeria_KLH11_
Silicibacter_lacuscaerulensis_ITI1157_
Ruegeria_pomeroyi_DSS3_
Citreicella_SE45_
Pelagibaca_bermudensis_HTCC2601_
Sagittula_stellata_E37_
Roseovarius_nubinhibens_ISM_
Roseovarius_TM1035_
Oceanibulbus_indolifex_HEL45_
Sulfitobacter_NAS141_
Sulfitobacter_GAI101_
Roseobacter_denitrificans_OCh_114_
Roseobacter_litoralis_Och_149_
Rhodobacterales_HTCC2083_
Loktanella_CCS2_
Loktanella_vestfoldensis_SKA53_
Thalassiobium_R2A62_
Octadecabacter_antarcticus_307_
Jannaschia_CCS1_
Rhodobacterales_HTCC2150_
Maritimibacter_alkaliphilus_HTCC2654_
Dinoroseobacter_shibae_DFL12_DSM_16493_
Oceanicola_granulosus_HTCC2516_
Ketogulonicigenium_vulgare_Y25_
Rhodobacter_sphaeroides_WS8N_
Rhodobacter_SW2_
Rhodobacter_capsulatus_SB1003_
Paracoccus_denitrificans_PD1222_
Parvularcula_bermudensis_HTCC2503_
Brevundimonas_subvibrioides_ATCC_15264_
Brevundimonas_diminuta_ATCC_11568_
Brevundimonas_BAL3_
Asticcacaulis_biprosthecum_C19_
Asticcacaulis_excentricus_CB_48_
Caulobacter_crescentus_CB15_
Caulobacter_segnis_ATCC_21756_
Caulobacter_K31_
Phenylobacterium_zucineum_HLK1_
Hirschia_baltica_ATCC_49814_
Hyphomonas_neptunium_ATCC_15444_
Maricaulis_maris_MCS10_
Erythrobacter_litoralis_HTCC2594_
Erythrobacter_NAP1_
Citromicrobium_bathyomarinum_JL354_
Novosphingobium_PP1Y_
Novosphingobium_aromaticivorans_DSM_12444_
Novosphingobium_nitrogenifigens_DSM_19370_
Sphingopyxis_alaskensis_RB2256_
Sphingomonas_wittichii_RW1_
Sphingomonas_S17_
Zymomonas_mobilis_mobilis_NCIMB_11163_
Sphingobium_chlorophenolicum_L1_
Sphingobium_japonicum_UT26S_
Acetobacter_pasteurianus_IFO_328301_
Acetobacter_pomorum_DM001_
Acetobacter_tropicalis_NBRC_101654_
Gluconobacter_oxydans_621H_
Gluconacetobacter_SXCC1_
Gluconacetobacter_diazotrophicus_PAl_5_DSM_5601_
Granulibacter_bethesdensis_CGDNIH1_
Acidiphilium_cryptum_JF5_
Acidiphilium_multivorum_AIU301_
Azospirillum_B510_
Rhodocista_centenaria_SW_
Nisaea_BAL199_
Candidatus_Puniceispirillum_marinum_IMCC1322_
Magnetospirillum_magnetotacticum_MS1_
Magnetospirillum_magneticum_AMB1_
Rhodospirillum_rubrum_S1_ATCC_11170_
Polysphondylium_pallidum_mitochondrion_
Dictyostelium_discoideum_mitochondrion_
Acanthamoeba_castellanii_mitochondrion_
Hartmannella_vermiformis_mitochondrion_
Naegleria_gruberi_mitochondrion_
Glaucocystis_nostochinearum_mitochondrion_
Cyanophora_paradoxa_strain_CCMP_329_mitochondrion_
Cyanidioschyzon_merolae_mitochondrion_
Chondrus_crispus_mitochondrion_
Pilayella_littoralis_mitochondrion_
Chattonella_marina_mitochondrion_
Ochromonas_danica_mitochondrion_
Saprolegnia_ferax_strain_ATCC_36051_mitochondrion_
Phytophthora_infestans_mitochondrion_
Malawimonas_jakobiformis_mitochondrion_
Marchantia_polymorpha_mitochondrion_
Arabidopsis_thaliana_mitochondrion_
Megaceros_aenigmaticus_mitochondrion_
Chara_vulgaris_mitochondrion_
Chaetosphaeridium_globosum_mitochondrion_
Nephroselmis_olivacea_mitochondrion_
Chlorokybus_atmophyticus_strain_SAG_4880_mitochondrion_
Prototheca_wickerhamii_263-11_mitochondrion_
Reclinomonas_americana_mitochondrion_
Ehrlichia_chaffeensis_Arkansas_
Ehrlichia_canis_Jake_
Ehrlichia_ruminantium_Gardel_
Anaplasma_marginale_Puerto_Rico_
Anaplasma_centrale_str_Israel_
Anaplasma_phagocytophilum_HZ_
Wolbachia_of_Drosophila_melanogaster_
Wolbachia_of_Culex_quinquefasciatus_JHB_
Wolbachia_of_Brugia_malayi_
Neorickettsia_risticii_Illinois_
Neorickettsia_sennetsu_Miyayama_
Rickettsia_canadensis_McKiel_
Rickettsia_felis_URRWXCal2_
Rickettsia_bellii_RML369C_
Rickettsia_typhi_Wilmington_
Orientia_tsutsugamushi_Ikeda_
Candidatus_Midichloria_mitochondrii_IricVA_
Candidatus_Pelagibacter_HTCC7211_
Candidatus_Pelagibacter_ubique_HTCC1002_
Pelagibacteraceae_HIMB5B_
Pelagibacteraceae_440_
Pelagibacteraceae_313_
Pelagibacteraceae_HIMB114_
Pelagibacteraceae_IMCC9063_
Pelagibacteraceae_HIMB59_
Candidatus_Odyssella_thessalonicensis_L13_
Caedibacter_caryophilus_221_
Magnetococcus_MC1_
Mariprofundus_ferrooxydans_PV1_
Thiobacillus_denitrificans_ATCC_25259_
Ralstonia_solanacearum_GMI1000_
Acidithiobacillus_ferrooxidans_ATCC_23270_
Escherichia_coli_MG1655_
Xanthomonas_campestris_8004_
Campylobacter_jejuni_ICDCCJ07001_
0.4

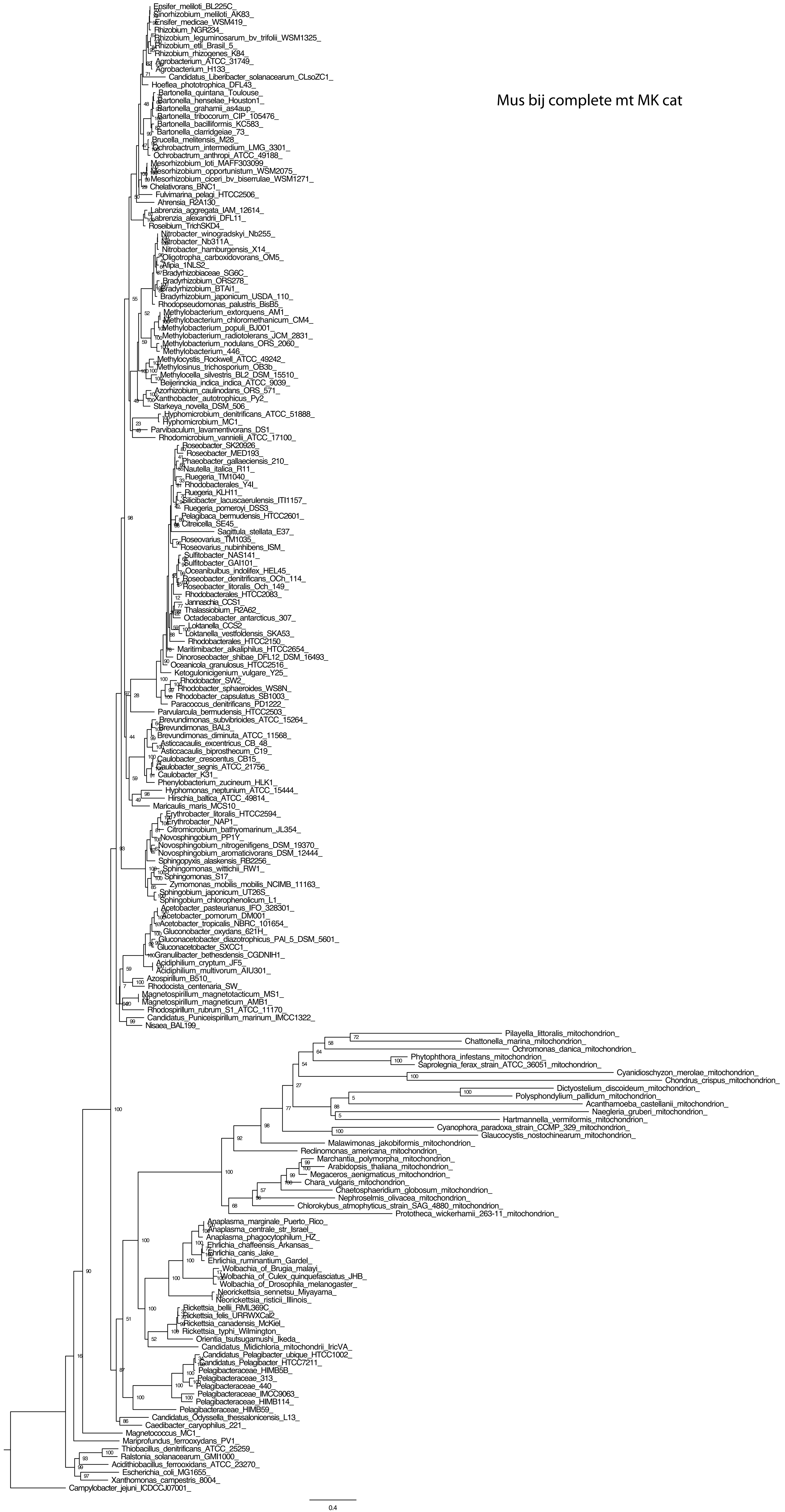

Mus bij complete mt MK cat
Ensifer_meliloti_BL225C_
Sinorhizobium_meliloti_AK83_
Ensifer_medicae_WSM419_
Rhizobium_NGR234_
Rhizobium_leguminosarum_bv_trifolii_WSM1325_
Rhizobium_etli_Brasil_5_
Rhizobium_rhizogenes_K84_
Agrobacterium_ATCC_31749_
Agrobacterium_H133_
Candidatus_Liberibacter_solanacearum_CLsoZC1_
Hoeflea_phototrophica_DFL43_
Bartonella_quintana_Toulouse_
Bartonella_henselae_Houston1_
Bartonella_grahamii_as4aup_
Bartonella_tribocorum_CIP_105476_
Bartonella_bacilliformis_KC583_
Bartonella_clarridgeiae_73_
Brucella_melitensis_M28_
Ochrobactrum_intermedium_LMG_3301_
Ochrobactrum_anthropi_ATCC_49188_
Mesorhizobium_loti_MAFF303099_
Mesorhizobium_opportunistum_WSM2075_
Mesorhizobium_ciceri_bv_biserrulae_WSM1271_
Chelativorans_BNC1_
Fulvimarina_pelagi_HTCC2506_
Ahrensia_R2A130_
Labrenzia_aggregata_IAM_12614_
Labrenzia_alexandrii_DFL11_
Roseibium_TrichSKD4_
Nitrobacter_winogradskyi_Nb255_
Nitrobacter_Nb311A_
Nitrobacter_hamburgensis_X14_
Oligotropha_carboxidovorans_OM5_
Afipia_1NLS2_
Bradyrhizobiaceae_SG6C_
Bradyrhizobium_ORS278_
Bradyrhizobium_BTAi1_
Bradyrhizobium_japonicum_USDA_110_
Rhodopseudomonas_palustris_BisB5_
Methylobacterium_extorquens_AM1_
Methylobacterium_chloromethanicum_CM4_
Methylobacterium_populi_BJ001_
Methylobacterium_radiotolerans_JCM_2831_
Methylobacterium_nodulans_ORS_2060_
Methylobacterium_446_
Methylocystis_Rockwell_ATCC_49242_
Methylosinus_trichosporium_OB3b_
Methylocella_silvestris_BL2_DSM_15510_
Beijerinckia_indica_indica_ATCC_9039_
Azorhizobium_caulinodans_ORS_571_
Xanthobacter_autotrophicus_Py2_
Starkeya_novella_DSM_506_
Hyphomicrobium_denitrificans_ATCC_51888_
Hyphomicrobium_MC1_
Parvibaculum_lavamentivorans_DS1_
Rhodomicrobium_vannielii_ATCC_17100_
Roseobacter_SK20926_
Roseobacter_MED193_
Phaeobacter_gallaeciensis_210_
Nautella_italica_R11_
Ruegeria_TM1040_
Rhodobacterales_Y4I_
Ruegeria_KLH11_
Silicibacter_lacuscaerulensis_ITI1157_
Ruegeria_pomeroyi_DSS3_
Pelagibaca_bermudensis_HTCC2601_
Citreicella_SE45_
Sagittula_stellata_E37_
Roseovarius_TM1035_
Roseovarius_nubinhibens_ISM_
Sulfitobacter_NAS141_
Sulfitobacter_GAI101_
Oceanibulbus_indolifex_HEL45_
Roseobacter_denitrificans_OCh_114_
Roseobacter_litoralis_Och_149_
Rhodobacterales_HTCC2083_
Jannaschia_CCS1_
Thalassiobium_R2A62_
Octadecabacter_antarcticus_307_
Loktanella_CCS2_
Loktanella_vestfoldensis_SKA53_
Rhodobacterales_HTCC2150_
Maritimibacter_alkaliphilus_HTCC2654_
Dinoroseobacter_shibae_DFL12_DSM_16493_
Oceanicola_granulosus_HTCC2516_
Ketogulonicigenium_vulgare_Y25_
Rhodobacter_SW2_
Rhodobacter_sphaeroides_WS8N_
Rhodobacter_capsulatus_SB1003_
Paracoccus_denitrificans_PD1222_
Parvularcula_bermudensis_HTCC2503_
Brevundimonas_subvibrioides_ATCC_15264_
Brevundimonas_BAL3_
Brevundimonas_diminuta_ATCC_11568_
Asticcacaulis_excentricus_CB_48_
Asticcacaulis_biprosthecum_C19_
Caulobacter_crescentus_CB15_
Caulobacter_segnis_ATCC_21756_
Caulobacter_K31_
Phenylobacterium_zucineum_HLK1_
Hyphomonas_neptunium_ATCC_15444_
Hirschia_baltica_ATCC_49814_
Maricaulis_maris_MCS10_
Erythrobacter_litoralis_HTCC2594_
Erythrobacter_NAP1_
Citromicrobium_bathyomarinum_JL354_
Novosphingobium_PP1Y_
Novosphingobium_nitrogenifigens_DSM_19370_
Novosphingobium_aromaticivorans_DSM_12444_
Sphingopyxis_alaskensis_RB2256_
Sphingomonas_wittichii_RW1_
Sphingomonas_S17_
Zymomonas_mobilis_mobilis_NCIMB_11163_
Sphingobium_japonicum_UT26S_
Sphingobium_chlorophenolicum_L1_
Acetobacter_pasteurianus_IFO_328301_
Acetobacter_pomorum_DM001_
Acetobacter_tropicalis_NBRC_101654_
Gluconobacter_oxydans_621H_
Gluconacetobacter_diazotrophicus_PAl_5_DSM_5601_
Gluconacetobacter_SXCC1_
Granulibacter_bethesdensis_CGDNIH1_
Acidiphilium_cryptum_JF5_
Acidiphilium_multivorum_AIU301_
Azospirillum_B510_
Rhodocista_centenaria_SW_
Magnetospirillum_magnetotacticum_MS1_
Magnetospirillum_magneticum_AMB1_
Rhodospirillum_rubrum_S1_ATCC_11170_
Candidatus_Puniceispirillum_marinum_IMCC1322_
Nisaea_BAL199_
Pilayella_littoralis_mitochondrion_
Chattonella_marina_mitochondrion_
Ochromonas_danica_mitochondrion_
Phytophthora_infestans_mitochondrion_
Saprolegnia_ferax_strain_ATCC_36051_mitochondrion_
Cyanidioschyzon_merolae_mitochondrion_
Chondrus_crispus_mitochondrion_
Dictyostelium_discoideum_mitochondrion_
Polysphondylium_pallidum_mitochondrion_
Acanthamoeba_castellanii_mitochondrion_
Naegleria_gruberi_mitochondrion_
Hartmannella_vermiformis_mitochondrion_
Cyanophora_paradoxa_strain_CCMP_329_mitochondrion_
Glaucocystis_nostochinearum_mitochondrion_
Malawimonas_jakobiformis_mitochondrion_
Reclinomonas_americana_mitochondrion_
Marchantia_polymorpha_mitochondrion_
Arabidopsis_thaliana_mitochondrion_
Megaceros_aenigmaticus_mitochondrion_
Chara_vulgaris_mitochondrion_
Chaetosphaeridium_globosum_mitochondrion_
Nephroselmis_olivacea_mitochondrion_
Chlorokybus_atmophyticus_strain_SAG_4880_mitochondrion_
Prototheca_wickerhamii_263-11_mitochondrion_
Anaplasma_marginale_Puerto_Rico_
Anaplasma_centrale_str_Israel_
Anaplasma_phagocytophilum_HZ_
Ehrlichia_chaffeensis_Arkansas_
Ehrlichia_canis_Jake_
Ehrlichia_ruminantium_Gardel_
Wolbachia_of_Brugia_malayi_
Wolbachia_of_Culex_quinquefasciatus_JHB_
Wolbachia_of_Drosophila_melanogaster_
Neorickettsia_sennetsu_Miyayama_
Neorickettsia_risticii_Illinois_
Rickettsia_bellii_RML369C_
Rickettsia_felis_URRWXCal2_
Rickettsia_canadensis_McKiel_
Rickettsia_typhi_Wilmington_
Orientia_tsutsugamushi_Ikeda_
Candidatus_Midichloria_mitochondrii_IricVA_
Candidatus_Pelagibacter_ubique_HTCC1002_
Candidatus_Pelagibacter_HTCC7211_
Pelagibacteraceae_HIMB5B_
Pelagibacteraceae_313_
Pelagibacteraceae_440_
Pelagibacteraceae_IMCC9063_
Pelagibacteraceae_HIMB114_
Pelagibacteraceae_HIMB59_
Candidatus_Odyssella_thessalonicensis_L13_
Caedibacter_caryophilus_221_
Magnetococcus_MC1_
Mariprofundus_ferrooxydans_PV1_
Thiobacillus_denitrificans_ATCC_25259_
Ralstonia_solanacearum_GMI1000_
Acidithiobacillus_ferrooxidans_ATCC_23270_
Escherichia_coli_MG1655_
Xanthomonas_campestris_8004_
Campylobacter_jejuni_ICDCCJ07001_
0.4

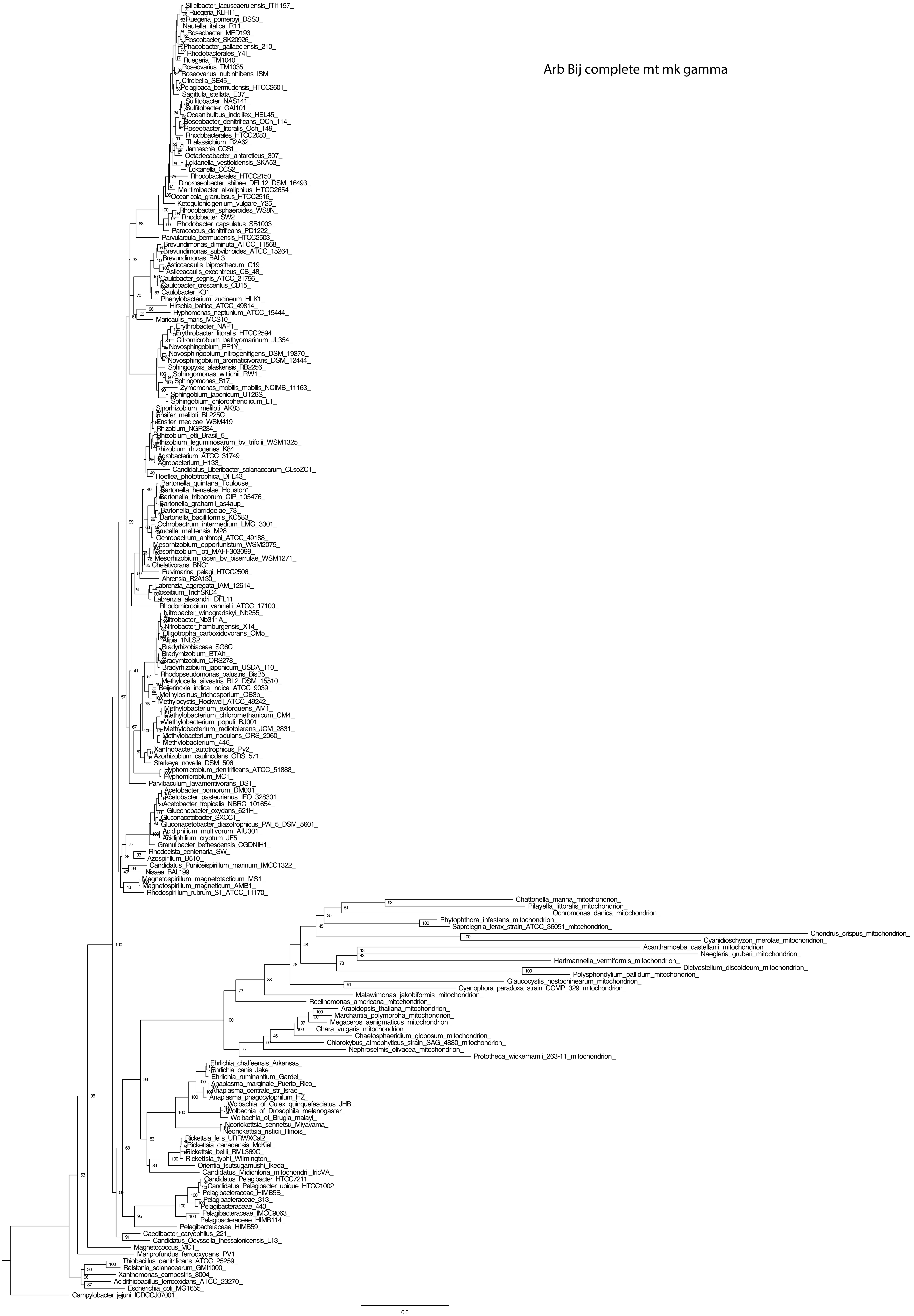

Arb Bij complete mt mk gamma
Silicibacter_lacuscaerulensis_ITI1157_
Ruegeria_KLH11_
Ruegeria_pomeroyi_DSS3_
Nautella_italica_R11_
Roseobacter_MED193_
Roseobacter_SK20926_
Phaeobacter_gallaeciensis_210_
Rhodobacterales_Y4I_
Ruegeria_TM1040_
Roseovarius_TM1035_
Roseovarius_nubinhibens_ISM_
Citreicella_SE45_
Pelagibaca_bermudensis_HTCC2601_
Sagittula_stellata_E37_
Sulfitobacter_NAS141_
Sulfitobacter_GAI101_
Oceanibulbus_indolifex_HEL45_
Roseobacter_denitrificans_OCh_114_
Roseobacter_litoralis_Och_149_
Rhodobacterales_HTCC2083_
Thalassiobium_R2A62_
Jannaschia_CCS1_
Octadecabacter_antarcticus_307_
Loktanella_vestfoldensis_SKA53_
Loktanella_CCS2_
Rhodobacterales_HTCC2150_
Dinoroseobacter_shibae_DFL12_DSM_16493_
Maritimibacter_alkaliphilus_HTCC2654_
Oceanicola_granulosus_HTCC2516_
Ketogulonicigenium_vulgare_Y25_
Rhodobacter_sphaeroides_WS8N_
Rhodobacter_SW2_
Rhodobacter_capsulatus_SB1003_
Paracoccus_denitrificans_PD1222_
Parvularcula_bermudensis_HTCC2503_
Brevundimonas_diminuta_ATCC_11568_
Brevundimonas_subvibrioides_ATCC_15264_
Brevundimonas_BAL3_
Asticcacaulis_biprosthecum_C19_
Asticcacaulis_excentricus_CB_48_
Caulobacter_segnis_ATCC_21756_
Caulobacter_crescentus_CB15_
Caulobacter_K31_
Phenylobacterium_zucineum_HLK1_
Hirschia_baltica_ATCC_49814_
Hyphomonas_neptunium_ATCC_15444_
Maricaulis_maris_MCS10_
Erythrobacter_NAP1_
Erythrobacter_litoralis_HTCC2594_
Citromicrobium_bathyomarinum_JL354_
Novosphingobium_PP1Y_
Novosphingobium_nitrogenifigens_DSM_19370_
Novosphingobium_aromaticivorans_DSM_12444_
Sphingopyxis_alaskensis_RB2256_
Sphingomonas_wittichii_RW1_
Sphingomonas_S17_
Zymomonas_mobilis_mobilis_NCIMB_11163_
Sphingobium_japonicum_UT26S_
Sphingobium_chlorophenolicum_L1_
Sinorhizobium_meliloti_AK83_
Ensifer_meliloti_BL225C_
Ensifer_medicae_WSM419_
Rhizobium_NGR234_
Rhizobium_etli_Brasil_5_
Rhizobium_leguminosarum_bv_trifolii_WSM1325_
Rhizobium_rhizogenes_K84_
Agrobacterium_ATCC_31749_
Agrobacterium_H133_
Candidatus_Liberibacter_solanacearum_CLsoZC1_
Hoeflea_phototrophica_DFL43_
Bartonella_quintana_Toulouse_
Bartonella_henselae_Houston1_
Bartonella_tribocorum_CIP_105476_
Bartonella_grahamii_as4aup_
Bartonella_clarridgeiae_73_
Bartonella_bacilliformis_KC583_
Ochrobactrum_intermedium_LMG_3301_
Brucella_melitensis_M28_
Ochrobactrum_anthropi_ATCC_49188_
Mesorhizobium_opportunistum_WSM2075_
Mesorhizobium_loti_MAFF303099_
Mesorhizobium_ciceri_bv_biserrulae_WSM1271_
Chelativorans_BNC1_
Fulvimarina_pelagi_HTCC2506_
Ahrensia_R2A130_
Labrenzia_aggregata_IAM_12614_
Roseibium_TrichSKD4_
Labrenzia_alexandrii_DFL11_
Rhodomicrobium_vannielii_ATCC_17100_
Nitrobacter_winogradskyi_Nb255_
Nitrobacter_Nb311A_
Nitrobacter_hamburgensis_X14_
Oligotropha_carboxidovorans_OM5_
Afipia_1NLS2_
Bradyrhizobiaceae_SG6C_
Bradyrhizobium_BTAi1_
Bradyrhizobium_ORS278_
Bradyrhizobium_japonicum_USDA_110_
Rhodopseudomonas_palustris_BisB5_
Methylocella_silvestris_BL2_DSM_15510_
Beijerinckia_indica_indica_ATCC_9039_
Methylosinus_trichosporium_OB3b_
Methylocystis_Rockwell_ATCC_49242_
Methylobacterium_extorquens_AM1_
Methylobacterium_chloromethanicum_CM4_
Methylobacterium_populi_BJ001_
Methylobacterium_radiotolerans_JCM_2831_
Methylobacterium_nodulans_ORS_2060_
Methylobacterium_446_
Xanthobacter_autotrophicus_Py2_
Azorhizobium_caulinodans_ORS_571_
Starkeya_novella_DSM_506_
Hyphomicrobium_denitrificans_ATCC_51888_
Hyphomicrobium_MC1_
Parvibaculum_lavamentivorans_DS1_
Acetobacter_pomorum_DM001_
Acetobacter_pasteurianus_IFO_328301_
Acetobacter_tropicalis_NBRC_101654_
Gluconobacter_oxydans_621H_
Gluconacetobacter_SXCC1_
Gluconacetobacter_diazotrophicus_PAI_5_DSM_5601_
Acidiphilium_multivorum_AIU301_
Acidiphilium_cryptum_JF5_
Granulibacter_bethesdensis_CGDNIH1_
Rhodocista_centenaria_SW_
Azospirillum_B510_
Candidatus_Puniceispirillum_marinum_IMCC1322_
Nisaea_BAL199_
Magnetospirillum_magnetotacticum_MS1_
Magnetospirillum_magneticum_AMB1_
Rhodospirillum_rubrum_S1_ATCC_11170_
Chattonella_marina_mitochondrion_
Pilayella_littoralis_mitochondrion_
Ochromonas_danica_mitochondrion_
Phytophthora_infestans_mitochondrion_
Saprolegnia_ferax_strain_ATCC_36051_mitochondrion_
Chondrus_crispus_mitochondrion_
Cyanidioschyzon_merolae_mitochondrion_
Acanthamoeba_castellanii_mitochondrion_
Naegleria_gruberi_mitochondrion_
Hartmannella_vermiformis_mitochondrion_
Dictyostelium_discoideum_mitochondrion_
Polysphondylium_pallidum_mitochondrion_
Glaucocystis_nostochinearum_mitochondrion_
Cyanophora_paradoxa_strain_CCMP_329_mitochondrion_
Malawimonas_jakobiformis_mitochondrion_
Reclinomonas_americana_mitochondrion_
Arabidopsis_thaliana_mitochondrion_
Marchantia_polymorpha_mitochondrion_
Megaceros_aenigmaticus_mitochondrion_
Chara_vulgaris_mitochondrion_
Chaetosphaeridium_globosum_mitochondrion_
Chlorokybus_atmophyticus_strain_SAG_4880_mitochondrion_
Nephroselmis_olivacea_mitochondrion_
Prototheca_wickerhamii_263-11_mitochondrion_
Ehrlichia_chaffeensis_Arkansas_
Ehrlichia_canis_Jake_
Ehrlichia_ruminantium_Gardel_
Anaplasma_marginale_Puerto_Rico_
Anaplasma_centrale_str_Israel_
Anaplasma_phagocytophilum_HZ_
Wolbachia_of_Culex_quinquefasciatus_JHB_
Wolbachia_of_Drosophila_melanogaster_
Wolbachia_of_Brugia_malayi_
Neorickettsia_sennetsu_Miyayama_
Neorickettsia_risticii_Illinois_
Rickettsia_felis_URRWXCal2_
Rickettsia_canadensis_McKiel_
Rickettsia_bellii_RML369C_
Rickettsia_typhi_Wilmington_
Orientia_tsutsugamushi_Ikeda_
Candidatus_Midichloria_mitochondrii_IricVA_
Candidatus_Pelagibacter_HTCC7211_
Candidatus_Pelagibacter_ubique_HTCC1002_
Pelagibacteraceae_HIMB5B_
Pelagibacteraceae_313_
Pelagibacteraceae_440_
Pelagibacteraceae_IMCC9063_
Pelagibacteraceae_HIMB114_
Pelagibacteraceae_HIMB59_
Caedibacter_caryophilus_221_
Candidatus_Odyssella_thessalonicensis_L13_
Magnetococcus_MC1_
Mariprofundus_ferrooxydans_PV1_
Thiobacillus_denitrificans_ATCC_25259_
Ralstonia_solanacearum_GMI1000_
Xanthomonas_campestris_8004_
Acidithiobacillus_ferrooxidans_ATCC_23270_
Escherichia_coli_MG1655_
Campylobacter_jejuni_ICDCCJ07001_
0.6

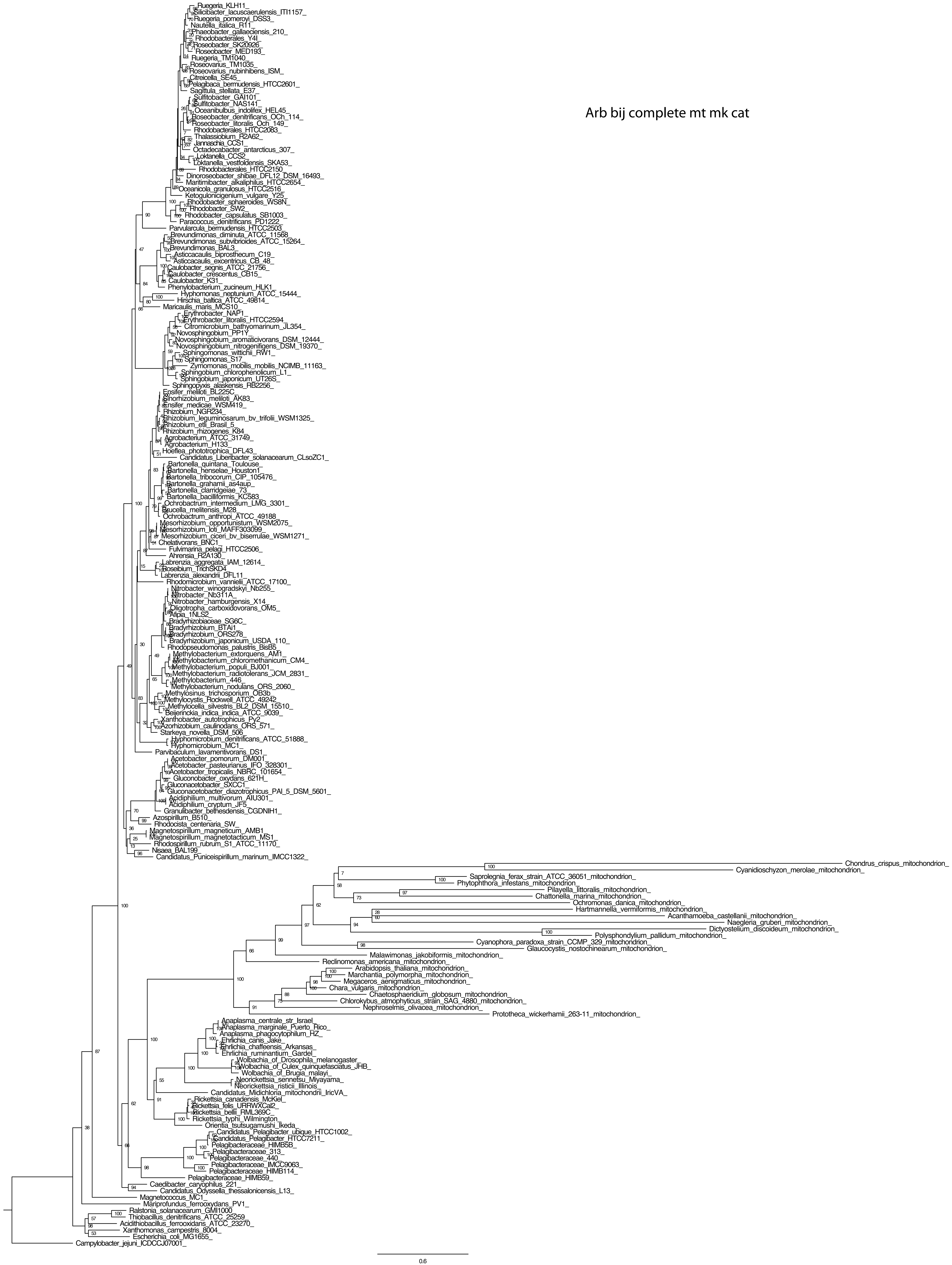

Arb bij complete mt mk cat
Ruegeria_KLH11_
Silicibacter_lacuscaerulensis_ITI1157_
Ruegeria_pomeroyi_DSS3_
Nautella_italica_R11_
Phaeobacter_gallaeciensis_210_
Rhodobacterales_Y4I_
Roseobacter_SK20926_
Roseobacter_MED193_
Ruegeria_TM1040_
Roseovarius_TM1035_
Roseovarius_nubinhibens_ISM_
Citreicella_SE45_
Pelagibaca_bermudensis_HTCC2601_
Sagittula_stellata_E37_
Sulfitobacter_GAI101_
Sulfitobacter_NAS141_
Oceanibulbus_indolifex_HEL45_
Roseobacter_denitrificans_OCh_114_
Roseobacter_litoralis_Och_149_
Rhodobacterales_HTCC2083_
Thalassiobium_R2A62_
Jannaschia_CCS1_
Octadecabacter_antarcticus_307_
Loktanella_CCS2_
Loktanella_vestfoldensis_SKA53_
Rhodobacterales_HTCC2150_
Dinoroseobacter_shibae_DFL12_DSM_16493_
Maritimibacter_alkaliphilus_HTCC2654_
Oceanicola_granulosus_HTCC2516_
Ketogulonicigenium_vulgare_Y25_
Rhodobacter_sphaeroides_WS8N_
Rhodobacter_SW2_
Rhodobacter_capsulatus_SB1003_
Paracoccus_denitrificans_PD1222_
Parvularcula_bermudensis_HTCC2503_
Brevundimonas_diminuta_ATCC_11568_
Brevundimonas_subvibrioides_ATCC_15264_
Brevundimonas_BAL3_
Asticcacaulis_biprosthecum_C19_
Asticcacaulis_excentricus_CB_48_
Caulobacter_segnis_ATCC_21756_
Caulobacter_crescentus_CB15_
Caulobacter_K31_
Phenylobacterium_zucineum_HLK1_
Hyphomonas_neptunium_ATCC_15444_
Hirschia_baltica_ATCC_49814_
Maricaulis_maris_MCS10_
Erythrobacter_NAP1_
Erythrobacter_litoralis_HTCC2594_
Citromicrobium_bathyomarinum_JL354_
Novosphingobium_PP1Y_
Novosphingobium_aromaticivorans_DSM_12444_
Novosphingobium_nitrogenifigens_DSM_19370_
Sphingomonas_wittichii_RW1_
Sphingomonas_S17_
Zymomonas_mobilis_mobilis_NCIMB_11163_
Sphingobium_chlorophenolicum_L1_
Sphingobium_japonicum_UT26S_
Sphingopyxis_alaskensis_RB2256_
Ensifer_meliloti_BL225C_
Sinorhizobium_meliloti_AK83_
Ensifer_medicae_WSM419_
Rhizobium_NGR234_
Rhizobium_leguminosarum_bv_trifolii_WSM1325_
Rhizobium_etli_Brasil_5_
Rhizobium_rhizogenes_K84_
Agrobacterium_ATCC_31749_
Agrobacterium_H133_
Hoeflea_phototrophica_DFL43_
Candidatus_Liberibacter_solanacearum_CLsoZC1_
Bartonella_quintana_Toulouse_
Bartonella_henselae_Houston1_
Bartonella_tribocorum_CIP_105476_
Bartonella_grahamii_as4aup_
Bartonella_clarridgeiae_73_
Bartonella_bacilliformis_KC583_
Ochrobactrum_intermedium_LMG_3301_
Brucella_melitensis_M28_
Ochrobactrum_anthropi_ATCC_49188_
Mesorhizobium_opportunistum_WSM2075_
Mesorhizobium_loti_MAFF303099_
Mesorhizobium_ciceri_bv_biserrulae_WSM1271_
Chelativorans_BNC1_
Fulvimarina_pelagi_HTCC2506_
Ahrensia_R2A130_
Labrenzia_aggregata_IAM_12614_
Roseibium_TrichSKD4_
Labrenzia_alexandrii_DFL11_
Rhodomicrobium_vannielii_ATCC_17100_
Nitrobacter_winogradskyi_Nb255_
Nitrobacter_Nb311A_
Nitrobacter_hamburgensis_X14_
Oligotropha_carboxidovorans_OM5_
Afipia_1NLS2_
Bradyrhizobiaceae_SG6C_
Bradyrhizobium_BTAi1_
Bradyrhizobium_ORS278_
Bradyrhizobium_japonicum_USDA_110_
Rhodopseudomonas_palustris_BisB5_
Methylobacterium_extorquens_AM1_
Methylobacterium_chloromethanicum_CM4_
Methylobacterium_populi_BJ001_
Methylobacterium_radiotolerans_JCM_2831_
Methylobacterium_446_
Methylobacterium_nodulans_ORS_2060_
Methylosinus_trichosporium_OB3b_
Methylocystis_Rockwell_ATCC_49242_
Methylocella_silvestris_BL2_DSM_15510_
Beijerinckia_indica_indica_ATCC_9039_
Xanthobacter_autotrophicus_Py2_
Azorhizobium_caulinodans_ORS_571_
Starkeya_novella_DSM_506_
Hyphomicrobium_denitrificans_ATCC_51888_
Hyphomicrobium_MC1_
Parvibaculum_lavamentivorans_DS1_
Acetobacter_pomorum_DM001_
Acetobacter_pasteurianus_IFO_328301_
Acetobacter_tropicalis_NBRC_101654_
Gluconobacter_oxydans_621H_
Gluconacetobacter_SXCC1_
Gluconacetobacter_diazotrophicus_PAI_5_DSM_5601_
Acidiphilium_multivorum_AIU301_
Acidiphilium_cryptum_JF5_
Granulibacter_bethesdensis_CGDNIH1_
Azospirillum_B510_
Rhodocista_centenaria_SW_
Magnetospirillum_magneticum_AMB1_
Magnetospirillum_magnetotacticum_MS1_
Rhodospirillum_rubrum_S1_ATCC_11170_
Nisaea_BAL199_
Candidatus_Puniceispirillum_marinum_IMCC1322_
Chondrus_crispus_mitochondrion_
Cyanidioschyzon_merolae_mitochondrion_
Saprolegnia_ferax_strain_ATCC_36051_mitochondrion_
Phytophthora_infestans_mitochondrion_
Pilayella_littoralis_mitochondrion_
Chattonella_marina_mitochondrion_
Ochromonas_danica_mitochondrion_
Hartmannella_vermiformis_mitochondrion_
Acanthamoeba_castellanii_mitochondrion_
Naegleria_gruberi_mitochondrion_
Dictyostelium_discoideum_mitochondrion_
Polysphondylium_pallidum_mitochondrion_
Cyanophora_paradoxa_strain_CCMP_329_mitochondrion_
Glaucocystis_nostochinearum_mitochondrion_
Malawimonas_jakobiformis_mitochondrion_
Reclinomonas_americana_mitochondrion_
Arabidopsis_thaliana_mitochondrion_
Marchantia_polymorpha_mitochondrion_
Megaceros_aenigmaticus_mitochondrion_
Chara_vulgaris_mitochondrion_
Chaetosphaeridium_globosum_mitochondrion_
Chlorokybus_atmophyticus_strain_SAG_4880_mitochondrion_
Nephroselmis_olivacea_mitochondrion_
Prototheca_wickerhamii_263-11_mitochondrion_
Anaplasma_centrale_str_Israel_
Anaplasma_marginale_Puerto_Rico_
Anaplasma_phagocytophilum_HZ_
Ehrlichia_canis_Jake_
Ehrlichia_chaffeensis_Arkansas_
Ehrlichia_ruminantium_Gardel_
Wolbachia_of_Drosophila_melanogaster_
Wolbachia_of_Culex_quinquefasciatus_JHB_
Wolbachia_of_Brugia_malayi_
Neorickettsia_sennetsu_Miyayama_
Neorickettsia_risticii_Illinois_
Candidatus_Midichloria_mitochondrii_IricVA_
Rickettsia_canadensis_McKiel_
Rickettsia_felis_URRWXCal2_
Rickettsia_bellii_RML369C_
Rickettsia_typhi_Wilmington_
Orientia_tsutsugamushi_Ikeda_
Candidatus_Pelagibacter_ubique_HTCC1002_
Candidatus_Pelagibacter_HTCC7211_
Pelagibacteraceae_HIMB5B_
Pelagibacteraceae_313_
Pelagibacteraceae_440_
Pelagibacteraceae_IMCC9063_
Pelagibacteraceae_HIMB114_
Pelagibacteraceae_HIMB59_
Caedibacter_caryophilus_221_
Candidatus_Odyssella_thessalonicensis_L13_
Magnetococcus_MC1_
Mariprofundus_ferrooxydans_PV1_
Ralstonia_solanacearum_GMI1000_
Thiobacillus_denitrificans_ATCC_25259_
Acidithiobacillus_ferrooxidans_ATCC_23270_
Xanthomonas_campestris_8004_
Escherichia_coli_MG1655_
Campylobacter_jejuni_ICDCCJ07001_
0.6
