# Supplementary material for: New rRNA Gene-Based Phylogenies of the Alphaproteobacteria Provide Perspective on Major Groups, Mitochondrial Ancestry and Phylogenetic Instability
Source: PLoS One. 2013 Dec 11;8(12):e83383. doi: 10.1371/journal.pone.0083383 (PMC3859672; doi:10.1371/journal.pone.0083383)
Supplement: Figure S16 — A. RY-coded trimmed dataset trees, with and without mitochondria. B. MK-coded trimmed dataset trees, with and without mitochondria. (PDF) [file pone.0083383.s016.pdf]

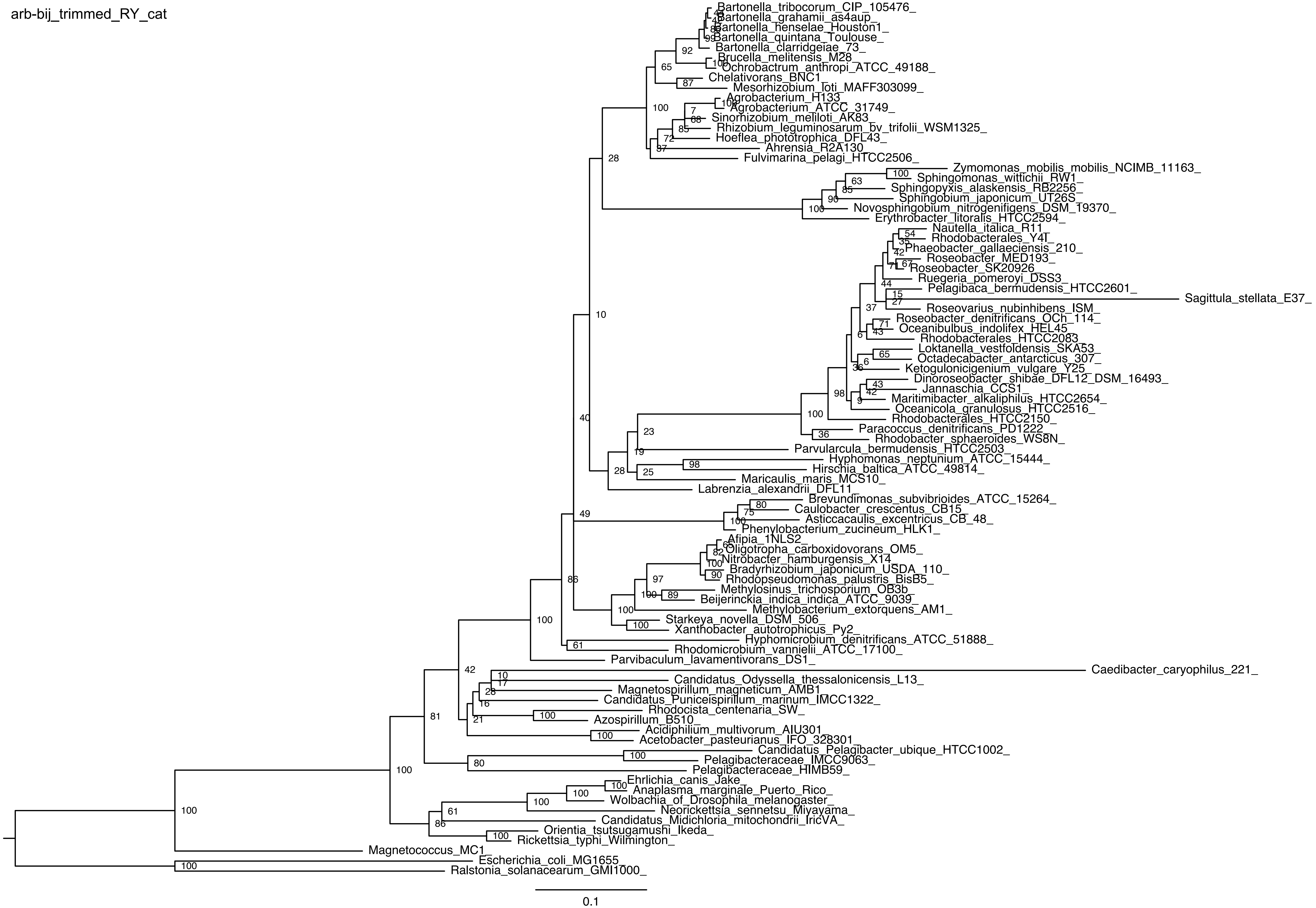
arb-bij_trimmed_RY_cat
Bartonella_tribocorum_CIP_105476_
Bartonella_grahamii_as4aup_
Bartonella_henselae_Houston1_
Bartonella_quintana_Toulouse_
Bartonella_clarridgeiae_73_
Brucella_melitensis_M28_
Ochrobactrum_anthropi_ATCC_49188_
Chelativorans_BNC1_
Mesorhizobium_loti_MAFF303099_
Agrobacterium_H133_
Agrobacterium_ATCC_31749_
Sinorhizobium_meliloti_AK83_
Rhizobium_leguminosarum_bv_trifolii_WSM1325_
Hoeflea_phototrophica_DFL43_
Ahrensia_R2A130_
Fulvimarina_pelagi_HTCC2506_
Zymomonas_mobilis_mobilis_NCIMB_11163_
Sphingomonas_wittichii_RW1_
Sphingopyxis_alaskensis_RB2256_
Sphingobium_japonicum_UT26S_
Novosphingobium_nitrogenifigens_DSM_19370_
Erythrobacter_litoralis_HTCC2594_
Nautella_italica_R11_
Rhodobacterales_Y4I_
Phaeobacter_gallaeciensis_210_
Roseobacter_MED193_
Roseobacter_SK20926_
Ruegeria_pomeroyi_DSS3_
Pelagibaca_bermudensis_HTCC2601_
Sagittula_stellata_E37_
Roseovarius_nubinhibens_ISM_
Roseobacter_denitrificans_OCh_114_
Oceanibulbus_indolifex_HEL45_
Rhodobacterales_HTCC2083_
Loktanella_vestfoldensis_SKA53_
Octadecabacter_antarcticus_307_
Ketogulonicigenium_vulgare_Y25_
Dinoroseobacter_shibae_DFL12_DSM_16493_
Jannaschia_CCS1_
Maritimibacter_alkaliphilus_HTCC2654_
Oceanicola_granulosus_HTCC2516_
Rhodobacterales_HTCC2150_
Paracoccus_denitrificans_PD1222_
Rhodobacter_sphaeroides_WS8N_
Parvularcula_bermudensis_HTCC2503_
Hyphomonas_neptunium_ATCC_15444_
Hirschia_baltica_ATCC_49814_
Maricaulis_maris_MCS10_
Labrenzia_alexandrii_DFL11_
Brevundimonas_subvibrioides_ATCC_15264_
Caulobacter_crescentus_CB15_
Asticcacaulis_excentricus_CB_48_
Phenylobacterium_zucineum_HLK1_
Afipia_1NLS2_
Oligotropha_carboxidovorans_OM5_
Nitrobacter_hamburgensis_X14_
Bradyrhizobium_japonicum_USDA_110_
Rhodopseudomonas_palustris_BisB5_
Methylosinus_trichosporium_OB3b_
Beijerinckia_indica_indica_ATCC_9039_
Methylobacterium_extorquens_AM1_
Starkeya_novella_DSM_506_
Xanthobacter_autotrophicus_Py2_
Hyphomicrobium_denitrificans_ATCC_51888_
Rhodomicrobium_vannielii_ATCC_17100_
Parvibaculum_lavamentivorans_DS1_
Caedibacter_caryophilus_221_
Candidatus_Odyssella_thessalonicensis_L13_
Magnetospirillum_magneticum_AMB1_
Candidatus_Puniceispirillum_marinum_IMCC1322_
Rhodocista_centenaria_SW_
Azospirillum_B510_
Acidiphilium_multivorum_AIU301_
Acetobacter_pasteurianus_IFO_328301_
Candidatus_Pelagibacter_ubique_HTCC1002_
Pelagibacteraceae_IMCC9063_
Pelagibacteraceae_HIMB59_
Ehrlichia_canis_Jake_
Anaplasma_marginale_Puerto_Rico_
Wolbachia_of_Drosophila_melanogaster_
Neorickettsia_sennetsu_Miyayama_
Candidatus_Midichloria_mitochondrii_IricVA_
Orientia_tsutsugamushi_Ikeda_
Rickettsia_typhi_Wilmington_
Magnetococcus_MC1_
Escherichia_coli_MG1655_
Ralstonia_solanacearum_GMI1000_
0.1

arb-bij_trimmed_RY_gamma

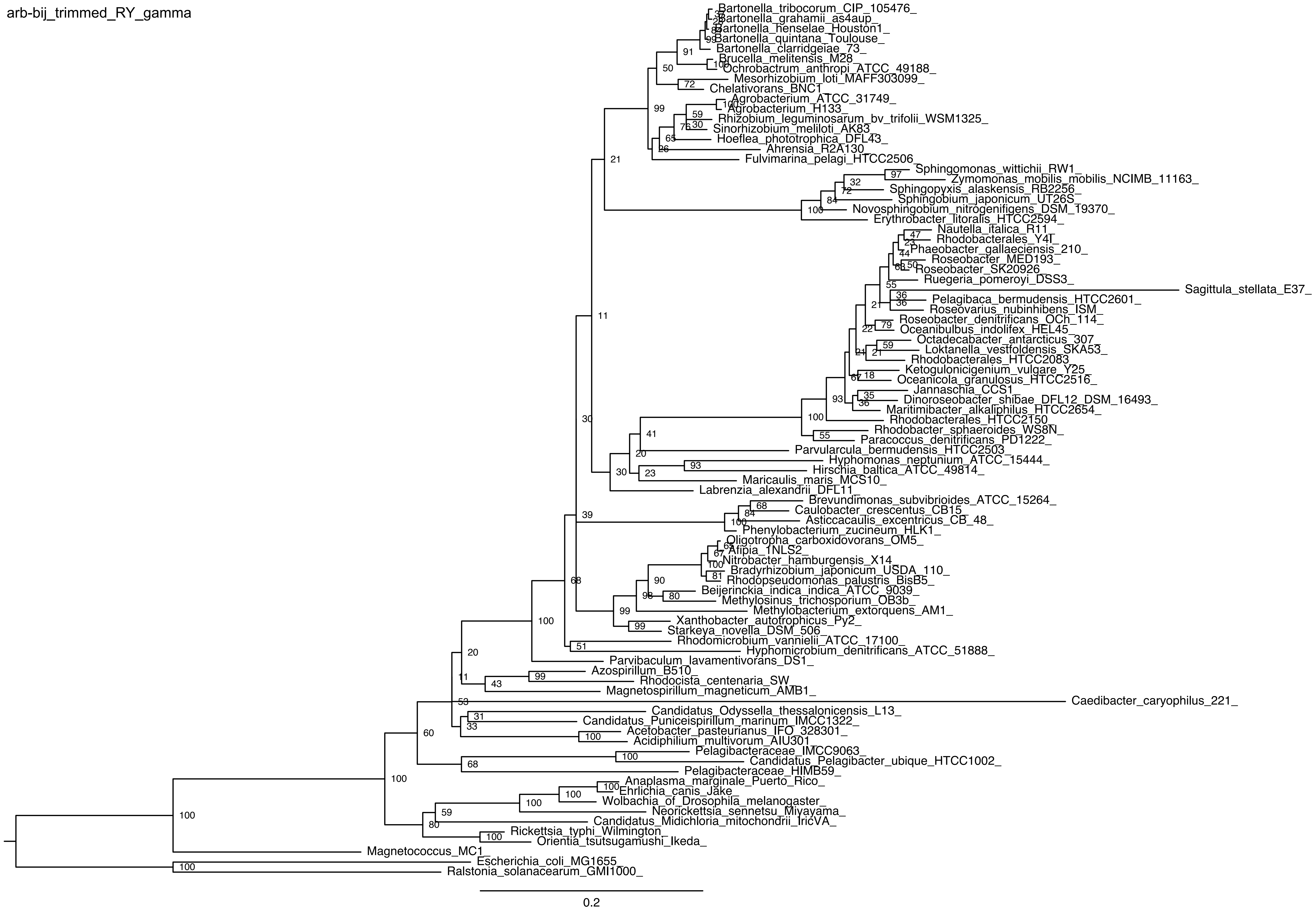

mus-bij_trimmed_RY_cat

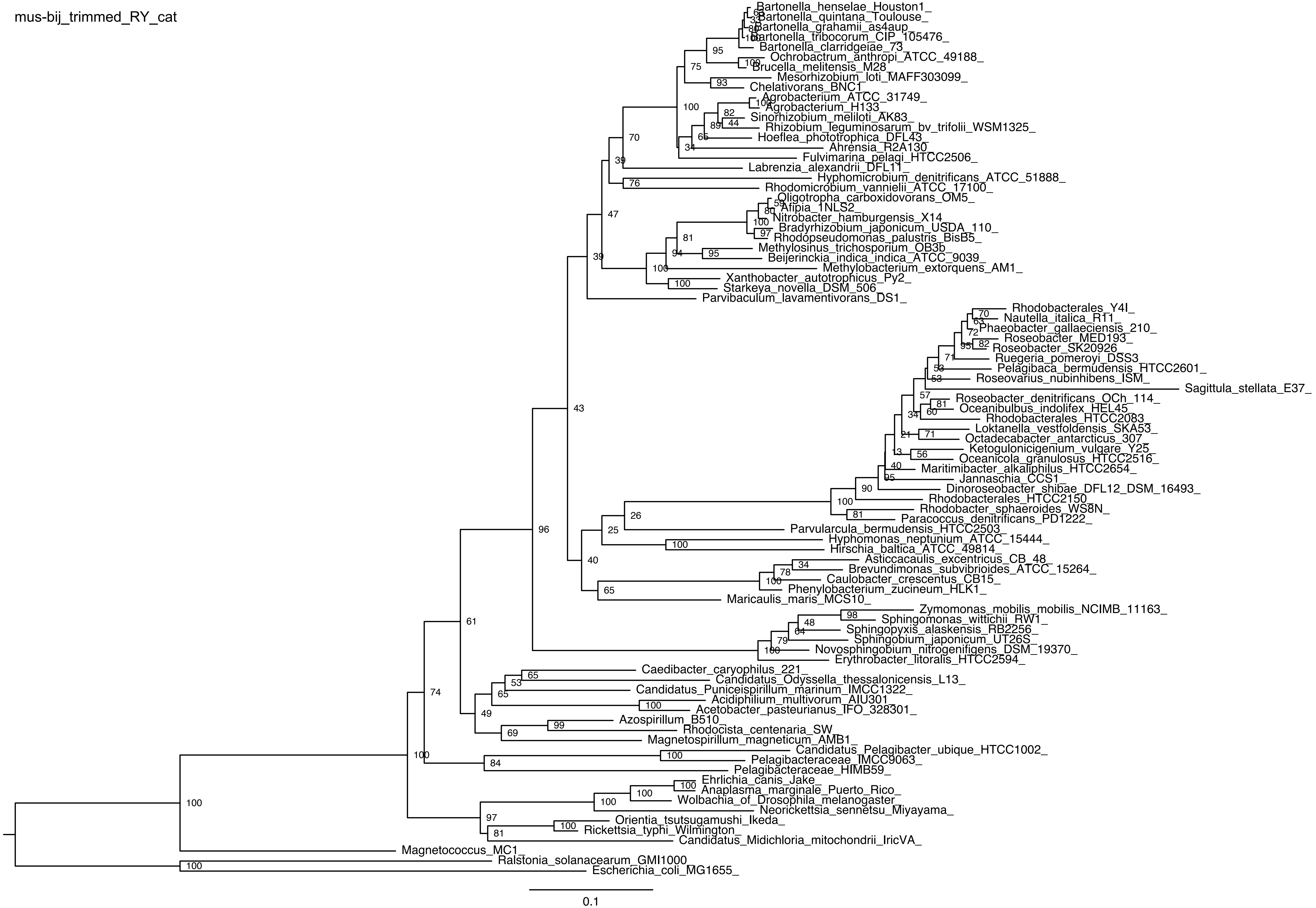

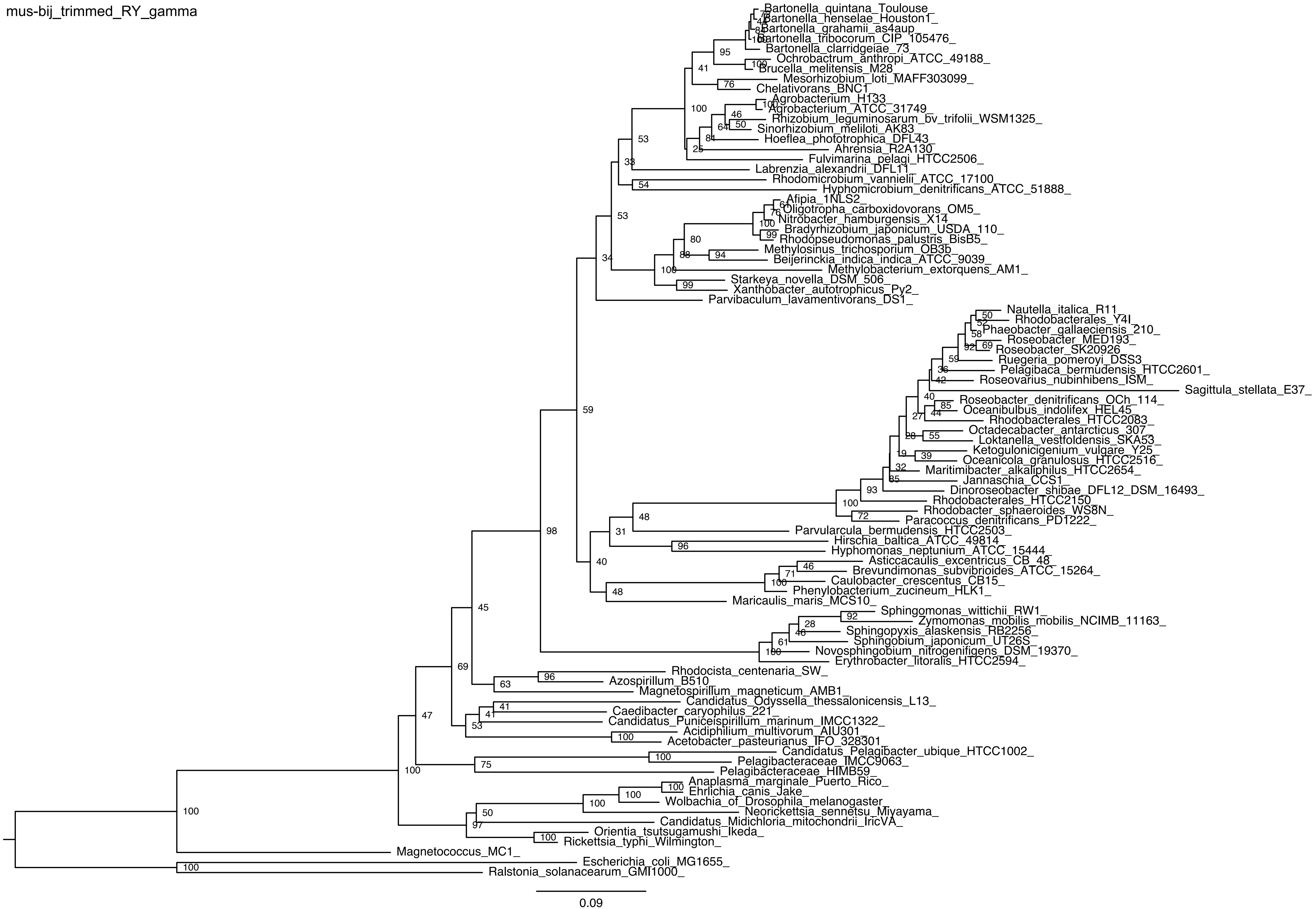
mus-bij_trimmed_RY_gamma
Bartonella_quintana_Toulouse_
Bartonella_henselae_Houston1_
Bartonella_grahamii_as4aup_
Bartonella_tribocorum_CIP_105476_
Bartonella_clarridgeiae_73_
Ochrobactrum_anthropi_ATCC_49188_
Brucella_melitensis_M28_
Mesorhizobium_loti_MAFF303099_
Chelativorans_BNC1_
Agrobacterium_H133_
Agrobacterium_ATCC_31749_
Rhizobium_leguminosarum_bv_trifolii_WSM1325_
Sinorhizobium_meliloti_AK83_
Hoeflea_phototrophica_DFL43_
Ahrensia_R2A130_
Fulvimarina_pelagi_HTCC2506_
Labrenzia_alexandrii_DFL11_
Rhodomicrobium_vannielii_ATCC_17100_
Hyphomicrobium_denitrificans_ATCC_51888_
Afipia_1NLS2_
Oligotropha_carboxidovorans_OM5_
Nitrobacter_hamburgensis_X14_
Bradyrhizobium_japonicum_USDA_110_
Rhodopseudomonas_palustris_BisB5_
Methylosinus_trichosporium_OB3b_
Beijerinckia_indica_indica_ATCC_9039_
Methylobacterium_extorquens_AM1_
Starkeya_novella_DSM_506_
Xanthobacter_autotrophicus_Py2_
Parvibaculum_lavamentivorans_DS1_
Nautella_italica_R11_
Rhodobacterales_Y4I_
Phaeobacter_gallaeciensis_210_
Roseobacter_MED193_
Roseobacter_SK20926_
Ruegeria_pomeroyi_DSS3_
Pelagibaca_bermudensis_HTCC2601_
Roseovarius_nubinhibens_ISM_
Sagittula_stellata_E37_
Roseobacter_denitrificans_OCh_114_
Oceanibulbus_indolifex_HEL45_
Rhodobacterales_HTCC2083_
Octadecabacter_antarcticus_307_
Loktanella_vestfoldensis_SKA53_
Ketogulonicigenium_vulgare_Y25_
Oceanicola_granulosus_HTCC2516_
Maritimibacter_alkaliphilus_HTCC2654_
Jannaschia_CCS1_
Dinoroseobacter_shibae_DFL12_DSM_16493_
Rhodobacterales_HTCC2150_
Rhodobacter_sphaeroides_WS8N_
Paracoccus_denitrificans_PD1222_
Parvularcula_bermudensis_HTCC2503_
Hirschia_baltica_ATCC_49814_
Hyphomonas_neptunium_ATCC_15444_
Asticcacaulis_excentricus_CB_48_
Brevundimonas_subvibrioides_ATCC_15264_
Caulobacter_crescentus_CB15_
Phenylobacterium_zucineum_HLK1_
Maricaulis_maris_MCS10_
Sphingomonas_wittichii_RW1_
Zymomonas_mobilis_mobilis_NCIMB_11163_
Sphingopyxis_alaskensis_RB2256_
Sphingobium_japonicum_UT26S_
Novosphingobium_nitrogenifigens_DSM_19370_
Erythrobacter_litoralis_HTCC2594_
Rhodocista_centenaria_SW_
Azospirillum_B510_
Magnetospirillum_magneticum_AMB1_
Candidatus_Odyssella_thessalonicensis_L13_
Caedibacter_caryophilus_221_
Candidatus_Puniceispirillum_marinum_IMCC1322_
Acidiphilium_multivorum_AIU301_
Acetobacter_pasteurianus_IFO_328301_
Candidatus_Pelagibacter_ubique_HTCC1002_
Pelagibacteraceae_IMCC9063_
Pelagibacteraceae_HIMB59_
Anaplasma_marginale_Puerto_Rico_
Ehrlichia_canis_Jake_
Wolbachia_of_Drosophila_melanogaster_
Neorickettsia_sennetsu_Miyayama_
Candidatus_Midichloria_mitochondrii_IricVA_
Orientia_tsutsugamushi_Ikeda_
Rickettsia_typhi_Wilmington_
Magnetococcus_MC1_
Escherichia_coli_MG1655_
Ralstonia_solanacearum_GMI1000_
0.09

arb-bij_trimmed_mt_RY_cat

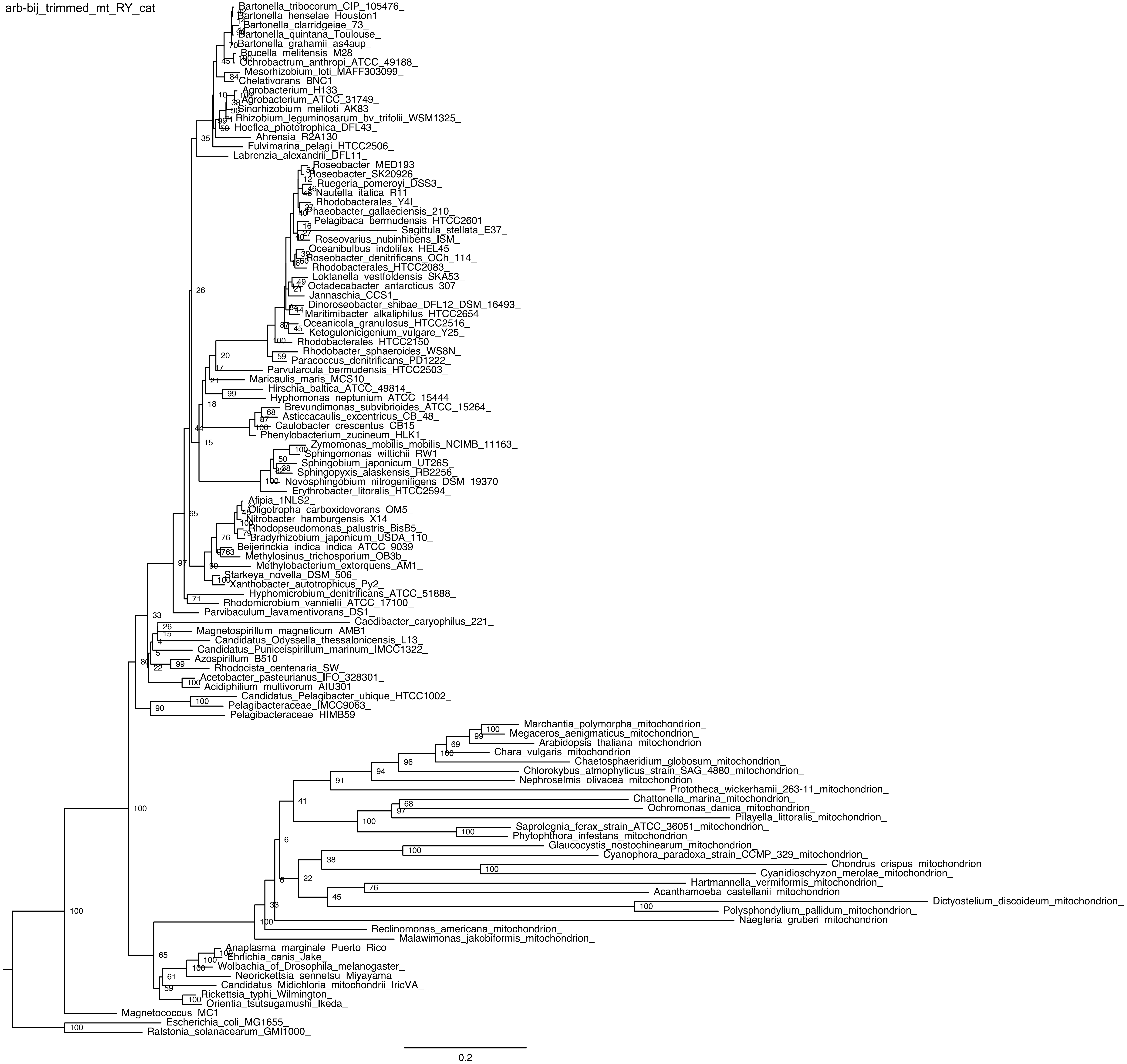

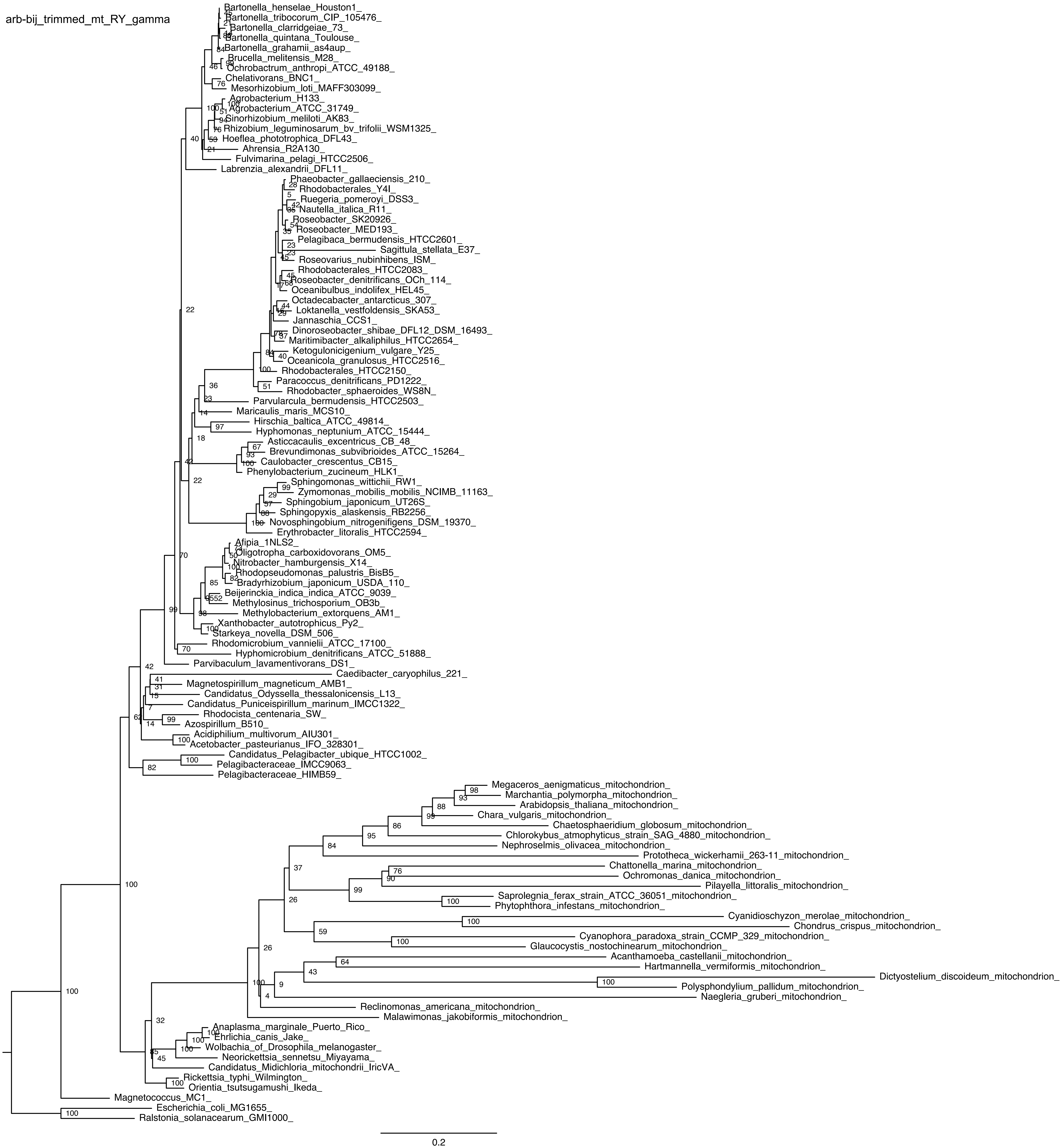

arb-bij_trimmed_mt_RY_gamma
Bartonella_henselae_Houston1_
Bartonella_tribocorum_CIP_105476_
Bartonella_clarridgeiae_73_
Bartonella_quintana_Toulouse_
Bartonella_grahamii_as4aup_
Brucella_melitensis_M28_
Ochrobactrum_anthropi_ATCC_49188_
Chelativorans_BNC1_
Mesorhizobium_loti_MAFF303099_
Agrobacterium_H133_
Agrobacterium_ATCC_31749_
Sinorhizobium_meliloti_AK83_
Rhizobium_leguminosarum_bv_trifolii_WSM1325_
Hoeflea_phototrophica_DFL43_
Ahrensia_R2A130_
Fulvimarina_pelagi_HTCC2506_
Labrenzia_alexandrii_DFL11_
Phaeobacter_gallaeciensis_210_
Rhodobacterales_Y4I_
Ruegeria_pomeroyi_DSS3_
Nautella_italica_R11_
Roseobacter_SK20926_
Roseobacter_MED193_
Pelagibaca_bermudensis_HTCC2601_
Sagittula_stellata_E37_
Roseovarius_nubinhibens_ISM_
Rhodobacterales_HTCC2083_
Roseobacter_denitrificans_OCh_114_
Oceanibulbus_indolifex_HEL45_
Octadecabacter_antarcticus_307_
Loktanella_vestfoldensis_SKA53_
Jannaschia_CCS1_
Dinoroseobacter_shibae_DFL12_DSM_16493_
Maritimibacter_alkaliphilus_HTCC2654_
Ketogulonicigenium_vulgare_Y25_
Oceanicola_granulosus_HTCC2516_
Rhodobacterales_HTCC2150_
Paracoccus_denitrificans_PD1222_
Rhodobacter_sphaeroides_WS8N_
Parvularcula_bermudensis_HTCC2503_
Maricaulis_maris_MCS10_
Hirschia_baltica_ATCC_49814_
Hyphomonas_neptunium_ATCC_15444_
Asticcacaulis_excentricus_CB_48_
Brevundimonas_subvibrioides_ATCC_15264_
Caulobacter_crescentus_CB15_
Phenylobacterium_zucineum_HLK1_
Sphingomonas_wittichii_RW1_
Zymomonas_mobilis_mobilis_NCIMB_11163_
Sphingobium_japonicum_UT26S_
Sphingopyxis_alaskensis_RB2256_
Novosphingobium_nitrogenifigens_DSM_19370_
Erythrobacter_litoralis_HTCC2594_
Afipia_1NLS2_
Oligotropha_carboxidovorans_OM5_
Nitrobacter_hamburgensis_X14_
Rhodopseudomonas_palustris_BisB5_
Bradyrhizobium_japonicum_USDA_110_
Beijerinckia_indica_indica_ATCC_9039_
Methylosinus_trichosporium_OB3b_
Methylobacterium_extorquens_AM1_
Xanthobacter_autotrophicus_Py2_
Starkeya_novella_DSM_506_
Rhodomicrobium_vannielii_ATCC_17100_
Hyphomicrobium_denitrificans_ATCC_51888_
Parvibaculum_lavamentivorans_DS1_
Caedibacter_caryophilus_221_
Magnetospirillum_magneticum_AMB1_
Candidatus_Odyssella_thessalonicensis_L13_
Candidatus_Puniceispirillum_marinum_IMCC1322_
Rhodocista_centenaria_SW_
Azospirillum_B510_
Acidiphilium_multivorum_AIU301_
Acetobacter_pasteurianus_IFO_328301_
Candidatus_Pelagibacter_ubique_HTCC1002_
Pelagibacteraceae_IMCC9063_
Pelagibacteraceae_HIMB59_
Megaceros_aenigmaticus_mitochondrion_
Marchantia_polymorpha_mitochondrion_
Arabidopsis_thaliana_mitochondrion_
Chara_vulgaris_mitochondrion_
Chaetosphaeridium_globosum_mitochondrion_
Chlorokybus_atmophyticus_strain_SAG_4880_mitochondrion_
Nephroselmis_olivacea_mitochondrion_
Prototheca_wickerhamii_263-11_mitochondrion_
Chattonella_marina_mitochondrion_
Ochromonas_danica_mitochondrion_
Pilayella_littoralis_mitochondrion_
Saprolegnia_ferax_strain_ATCC_36051_mitochondrion_
Phytophthora_infestans_mitochondrion_
Cyanidioschyzon_merolae_mitochondrion_
Chondrus_crispus_mitochondrion_
Cyanophora_paradoxa_strain_CCMP_329_mitochondrion_
Glaucocystis_nostochinearum_mitochondrion_
Acanthamoeba_castellanii_mitochondrion_
Hartmannella_vermiformis_mitochondrion_
Dictyostelium_discoideum_mitochondrion_
Polysphondylium_pallidum_mitochondrion_
Naegleria_gruberi_mitochondrion_
Reclinomonas_americana_mitochondrion_
Malawimonas_jakobiformis_mitochondrion_
Anaplasma_marginale_Puerto_Rico_
Ehrlichia_canis_Jake_
Wolbachia_of_Drosophila_melanogaster_
Neorickettsia_sennetsu_Miyayama_
Candidatus_Midichloria_mitochondrii_IricVA_
Rickettsia_typhi_Wilmington_
Orientia_tsutsugamushi_Ikeda_
Magnetococcus_MC1_
Escherichia_coli_MG1655_
Ralstonia_solanacearum_GMI1000_
0.2

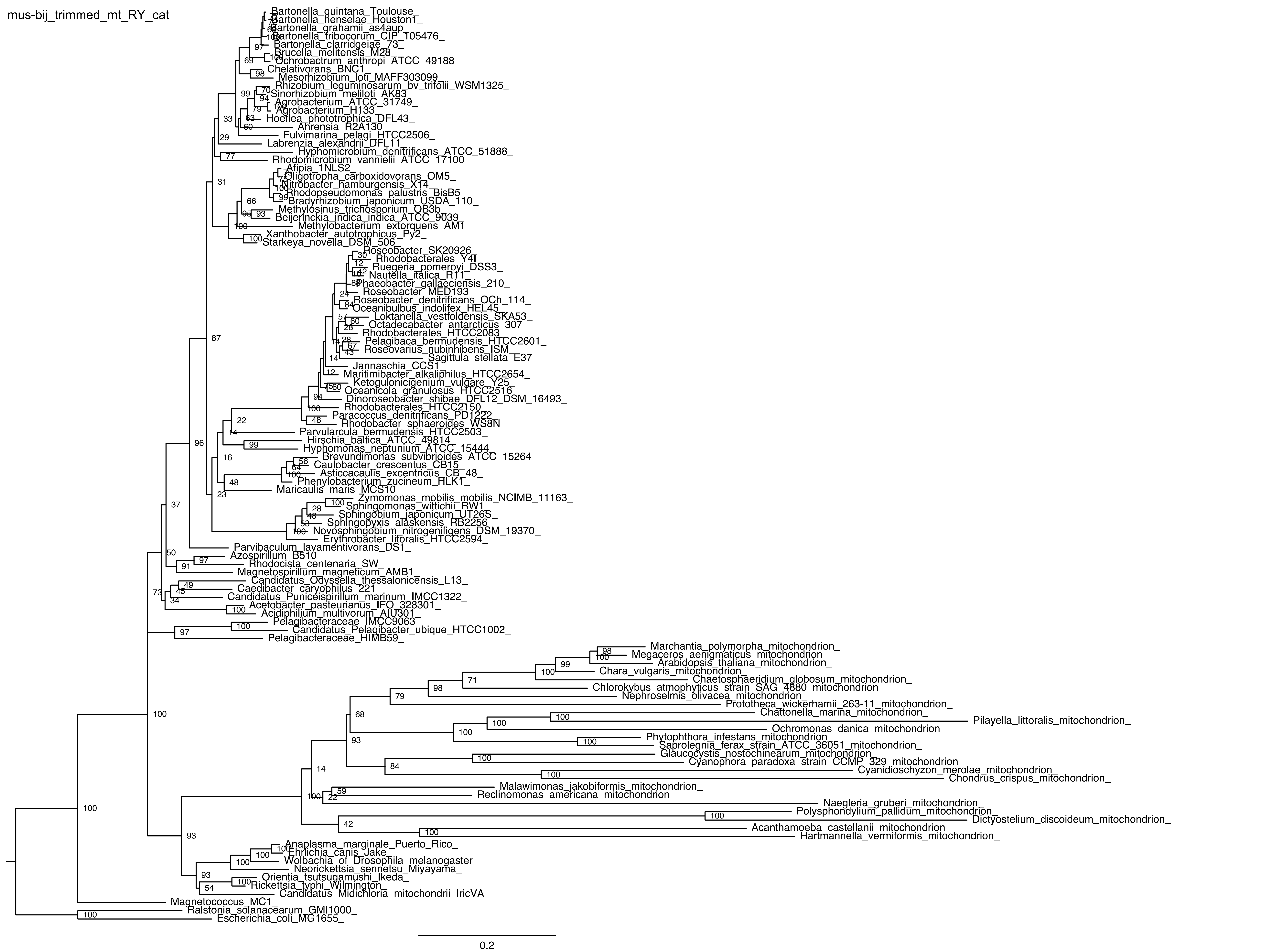
mus-bij_trimmed_mt_RY_cat
Bartonella_quintana_Toulouse_
Bartonella_henselae_Houston1_
Bartonella_grahamii_as4aup_
Bartonella_tribocorum_CIP_105476_
Bartonella_clarridgeiae_73_
Brucella_melitensis_M28_
Ochrobactrum_anthropi_ATCC_49188_
Chelativorans_BNC1_
Mesorhizobium_loti_MAFF303099_
Rhizobium_leguminosarum_bv_trifolii_WSM1325_
Sinorhizobium_meliloti_AK83_
Agrobacterium_ATCC_31749_
Agrobacterium_H133_
Hoeflea_phototrophica_DFL43_
Ahrensia_R2A130_
Fulvimarina_pelagi_HTCC2506_
Labrenzia_alexandrii_DFL11_
Hyphomicrobium_denitrificans_ATCC_51888_
Rhodomicrobium_vannielii_ATCC_17100_
Afipia_1NLS2_
Oligotropha_carboxidovorans_OM5_
Nitrobacter_hamburgensis_X14_
Rhodopseudomonas_palustris_BisB5_
Bradyrhizobium_japonicum_USDA_110_
Methylosinus_trichosporium_OB3b_
Beijerinckia_indica_indica_ATCC_9039_
Methylobacterium_extorquens_AM1_
Xanthobacter_autotrophicus_Py2_
Starkeya_novella_DSM_506_
Roseobacter_SK20926_
Rhodobacterales_Y4I_
Ruegeria_pomeroyi_DSS3_
Nautella_italica_R11_
Phaeobacter_gallaeciensis_210_
Roseobacter_MED193_
Roseobacter_denitrificans_OCh_114_
Oceanibulbus_indolifex_HEL45_
Loktanella_vestfoldensis_SKA53_
Octadecabacter_antarcticus_307_
Rhodobacterales_HTCC2083_
Pelagibaca_bermudensis_HTCC2601_
Roseovarius_nubinhibens_ISM_
Sagittula_stellata_E37_
Jannaschia_CCS1_
Maritimibacter_alkaliphilus_HTCC2654_
Ketogulonicigenium_vulgare_Y25_
Oceanicola_granulosus_HTCC2516_
Dinoroseobacter_shibae_DFL12_DSM_16493_
Rhodobacterales_HTCC2150_
Paracoccus_denitrificans_PD1222_
Rhodobacter_sphaeroides_WS8N_
Parvularcula_bermudensis_HTCC2503_
Hirschia_baltica_ATCC_49814_
Hyphomonas_neptunium_ATCC_15444_
Brevundimonas_subvibrioides_ATCC_15264_
Caulobacter_crescentus_CB15_
Asticcacaulis_excentricus_CB_48_
Phenylobacterium_zucineum_HLK1_
Maricaulis_maris_MCS10_
Zymomonas_mobilis_mobilis_NCIMB_11163_
Sphingomonas_wittichii_RW1_
Sphingobium_japonicum_UT26S_
Sphingopyxis_alaskensis_RB2256_
Novosphingobium_nitrogenifigens_DSM_19370_
Erythrobacter_litoralis_HTCC2594_
Parvibaculum_lavamentivorans_DS1_
Azospirillum_B510_
Rhodocista_centenaria_SW_
Magnetospirillum_magneticum_AMB1_
Candidatus_Odyssella_thessalonicensis_L13_
Caedibacter_caryophilus_221_
Candidatus_Puniceispirillum_marinum_IMCC1322_
Acetobacter_pasteurianus_IFO_328301_
Acidiphilium_multivorum_AIU301_
Pelagibacteraceae_IMCC9063_
Candidatus_Pelagibacter_ubique_HTCC1002_
Pelagibacteraceae_HIMB59_
Marchantia_polymorpha_mitochondrion_
Megaceros_aenigmaticus_mitochondrion_
Arabidopsis_thaliana_mitochondrion_
Chara_vulgaris_mitochondrion_
Chaetosphaeridium_globosum_mitochondrion_
Chlorokybus_atmophyticus_strain_SAG_4880_mitochondrion_
Nephroselmis_olivacea_mitochondrion_
Prototheca_wickerhamii_263-11_mitochondrion_
Chattonella_marina_mitochondrion_
Pilayella_littoralis_mitochondrion_
Ochromonas_danica_mitochondrion_
Phytophthora_infestans_mitochondrion_
Saprolegnia_ferax_strain_ATCC_36051_mitochondrion_
Glaucocystis_nostochinearum_mitochondrion_
Cyanophora_paradoxa_strain_CCMP_329_mitochondrion_
Cyanidioschyzon_merolae_mitochondrion_
Chondrus_crispus_mitochondrion_
Malawimonas_jakobiformis_mitochondrion_
Reclinomonas_americana_mitochondrion_
Naegleria_gruberi_mitochondrion_
Polysphondylium_pallidum_mitochondrion_
Dictyostelium_discoideum_mitochondrion_
Acanthamoeba_castellanii_mitochondrion_
Hartmannella_vermiformis_mitochondrion_
Anaplasma_marginale_Puerto_Rico_
Ehrlichia_canis_Jake_
Wolbachia_of_Drosophila_melanogaster_
Neorickettsia_sennetsu_Miyayama_
Orientia_tsutsugamushi_Ikeda_
Rickettsia_typhi_Wilmington_
Candidatus_Midichloria_mitochondrii_IricVA_
Magnetococcus_MC1_
Ralstonia_solanacearum_GMI1000_
Escherichia_coli_MG1655_
0.2

mus-bij_trimmed_mt_RY_gamma

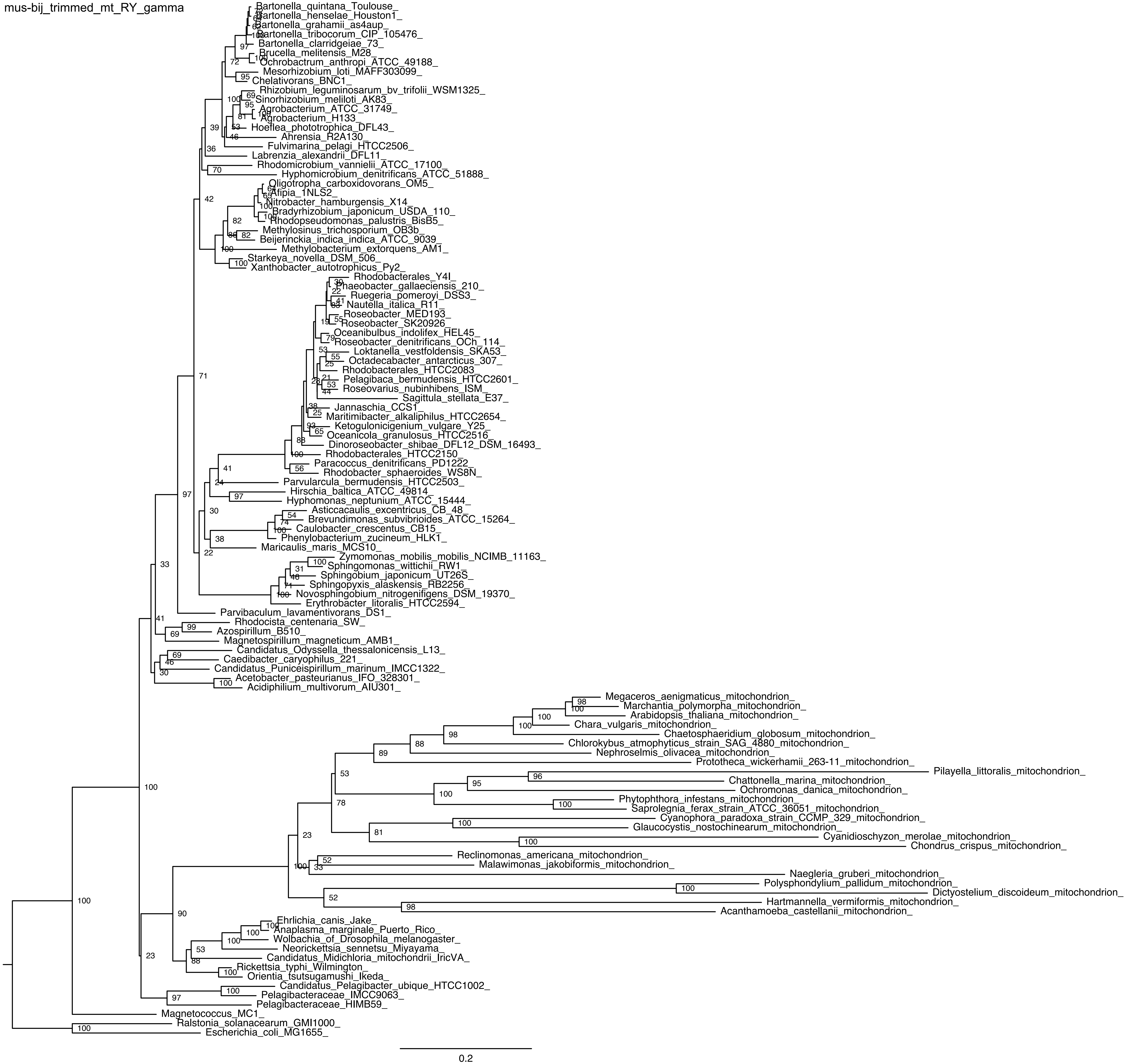

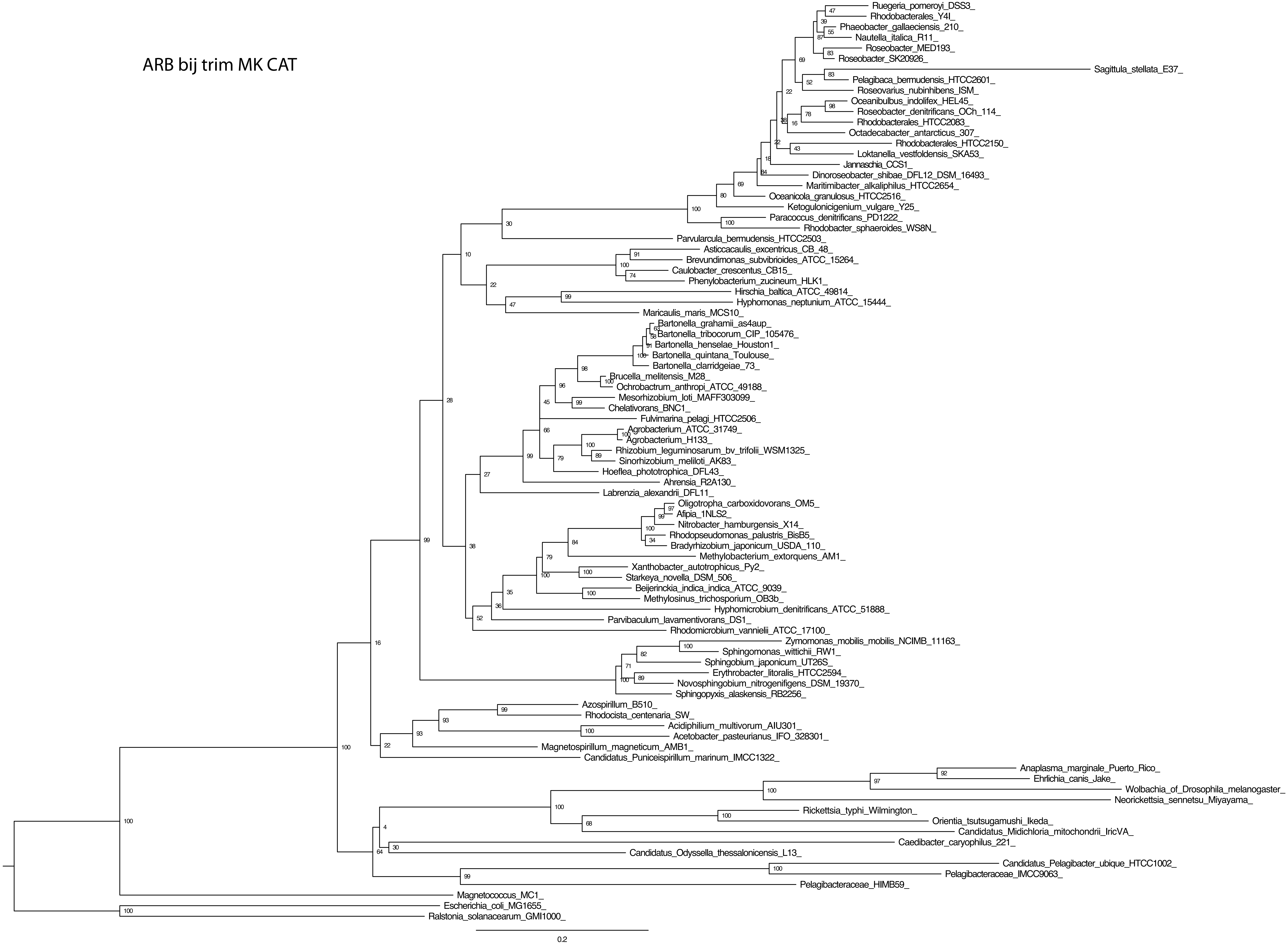
ARB bij trim MK CAT
Ruegeria_pomeroyi_DSS3_
Rhodobacterales_Y4I_
Phaeobacter_gallaeciensis_210_
Nautella_italica_R11_
Roseobacter_MED193_
Roseobacter_SK20926_
Sagittula_stellata_E37_
Pelagibaca_bermudensis_HTCC2601_
Roseovarius_nubinhibens_ISM_
Oceanibulbus_indolifex_HEL45_
Roseobacter_denitrificans_OCh_114_
Rhodobacterales_HTCC2083_
Octadecabacter_antarcticus_307_
Rhodobacterales_HTCC2150_
Loktanella_vestfoldensis_SKA53_
Jannaschia_CCS1_
Dinoroseobacter_shibae_DFL12_DSM_16493_
Maritimibacter_alkaliphilus_HTCC2654_
Oceanicola_granulosus_HTCC2516_
Ketogulonicigenium_vulgare_Y25_
Paracoccus_denitrificans_PD1222_
Rhodobacter_sphaeroides_WS8N_
Parvularcula_bermudensis_HTCC2503_
Asticcacaulis_excentricus_CB_48_
Brevundimonas_subvibrioides_ATCC_15264_
Caulobacter_crescentus_CB15_
Phenylobacterium_zucineum_HLK1_
Hirschia_baltica_ATCC_49814_
Hyphomonas_neptunium_ATCC_15444_
Maricaulis_maris_MCS10_
Bartonella_grahamii_as4aup_
Bartonella_tribocorum_CIP_105476_
Bartonella_henselae_Houston1_
Bartonella_quintana_Toulouse_
Bartonella_clarridgeiae_73_
Brucella_melitensis_M28_
Ochrobactrum_anthropi_ATCC_49188_
Mesorhizobium_loti_MAFF303099_
Chelativorans_BNC1_
Fulvimarina_pelagi_HTCC2506_
Agrobacterium_ATCC_31749_
Agrobacterium_H133_
Rhizobium_leguminosarum_bv_trifolii_WSM1325_
Sinorhizobium_meliloti_AK83_
Hoeflea_phototrophica_DFL43_
Ahrensia_R2A130_
Labrenzia_alexandrii_DFL11_
Oligotropha_carboxidovorans_OM5_
Afipia_1NLS2_
Nitrobacter_hamburgensis_X14_
Rhodopseudomonas_palustris_BisB5_
Bradyrhizobium_japonicum_USDA_110_
Methylobacterium_extorquens_AM1_
Xanthobacter_autotrophicus_Py2_
Starkeya_novella_DSM_506_
Beijerinckia_indica_indica_ATCC_9039_
Methylosinus_trichosporium_OB3b_
Hyphomicrobium_denitrificans_ATCC_51888_
Parvibaculum_lavamentivorans_DS1_
Rhodomicrobium_vannielii_ATCC_17100_
Zymomonas_mobilis_mobilis_NCIMB_11163_
Sphingomonas_wittichii_RW1_
Sphingobium_japonicum_UT26S_
Erythrobacter_litoralis_HTCC2594_
Novosphingobium_nitrogenifigens_DSM_19370_
Sphingopyxis_alaskensis_RB2256_
Azospirillum_B510_
Rhodocista_centenaria_SW_
Acidiphilium_multivorum_AIU301_
Acetobacter_pasteurianus_IFO_328301_
Magnetospirillum_magneticum_AMB1_
Candidatus_Puniceispirillum_marinum_IMCC1322_
Anaplasma_marginale_Puerto_Rico_
Ehrlichia_canis_Jake_
Wolbachia_of_Drosophila_melanogaster_
Neorickettsia_sennetsu_Miyayama_
Rickettsia_typhi_Wilmington_
Orientia_tsutsugamushi_Ikeda_
Candidatus_Midichloria_mitochondrii_IricVA_
Caedibacter_caryophilus_221_
Candidatus_Odyssella_thessalonicensis_L13_
Candidatus_Pelagibacter_ubique_HTCC1002_
Pelagibacteraceae_IMCC9063_
Pelagibacteraceae_HIMB59_
Magnetococcus_MC1_
Escherichia_coli_MG1655_
Ralstonia_solanacearum_GMI1000_
0.2

Mus bij trim MK gamma

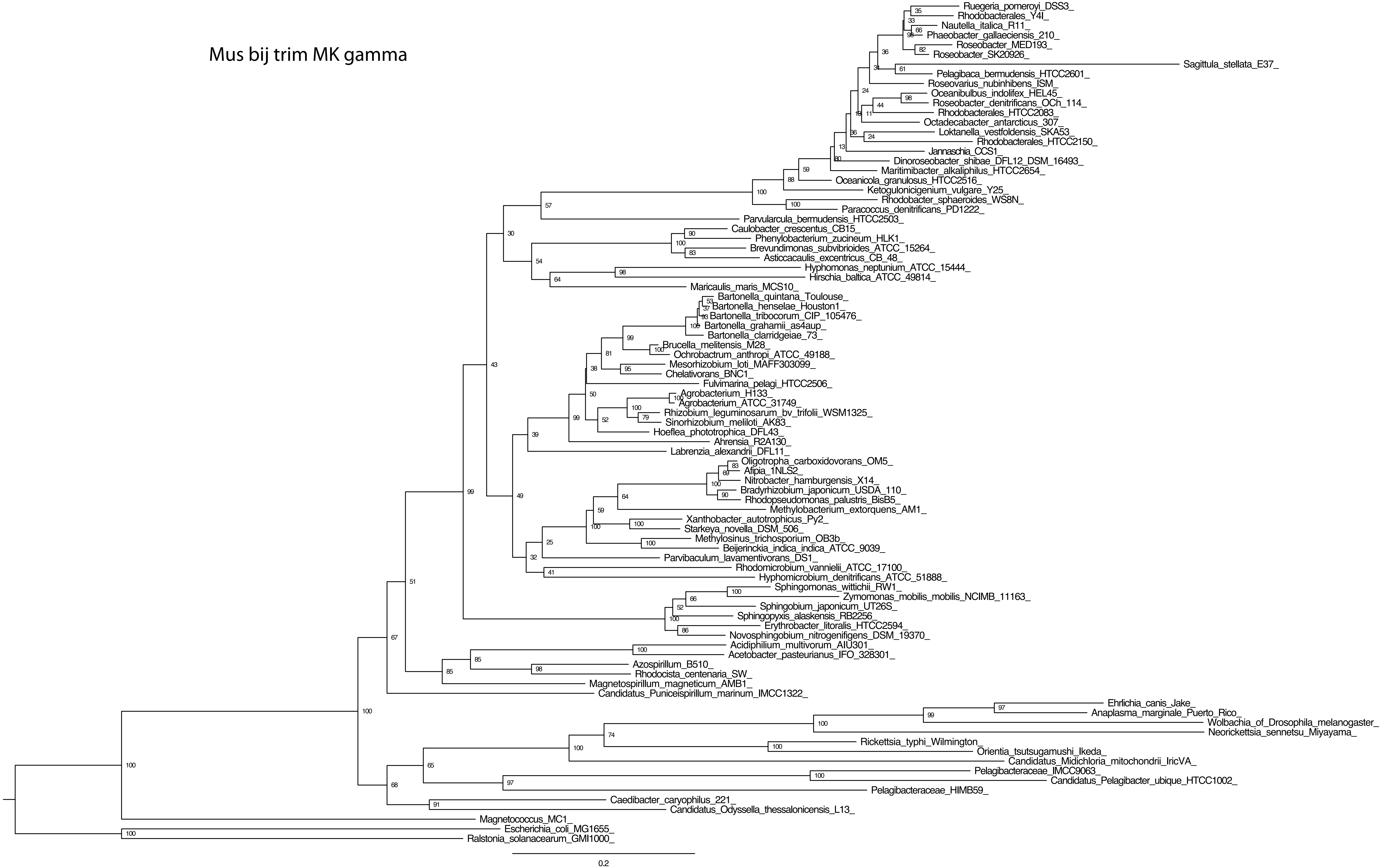

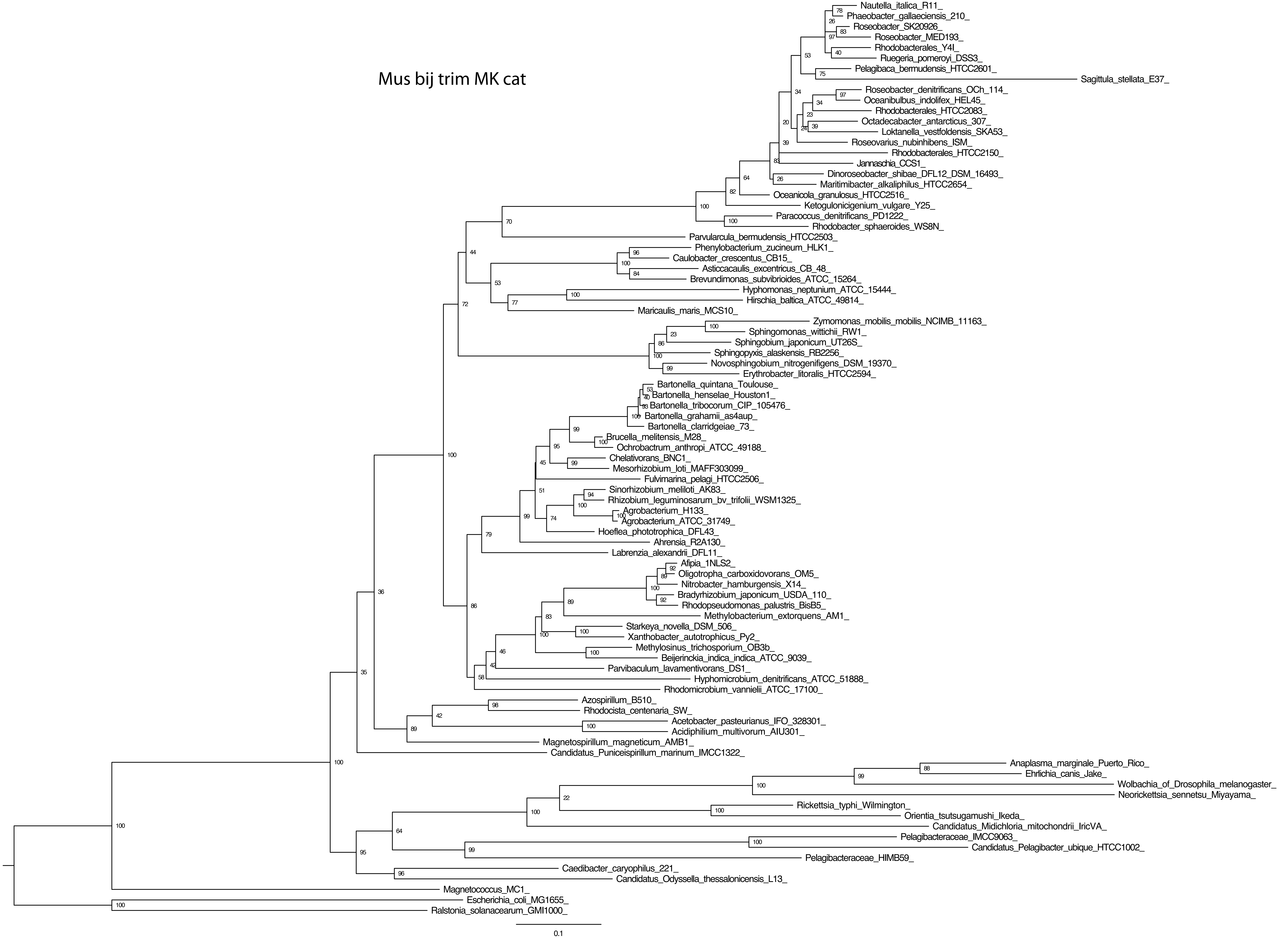
Mus bij trim MK cat
Nautella_italica_R11_
Phaeobacter_gallaeciensis_210_
Roseobacter_SK20926_
Roseobacter_MED193_
Rhodobacterales_Y4I_
Ruegeria_pomeroyi_DSS3_
Pelagibaca_bermudensis_HTCC2601_
Sagittula_stellata_E37_
Roseobacter_denitrificans_OCh_114_
Oceanibulbus_indolifex_HEL45_
Rhodobacterales_HTCC2083_
Octadecabacter_antarcticus_307_
Loktanella_vestfoldensis_SKA53_
Roseovarius_nubinhibens_ISM_
Rhodobacterales_HTCC2150_
Jannaschia_CCS1_
Dinoroseobacter_shibae_DFL12_DSM_16493_
Maritimibacter_alkaliphilus_HTCC2654_
Oceanicola_granulosus_HTCC2516_
Ketogulonicigenium_vulgare_Y25_
Paracoccus_denitrificans_PD1222_
Rhodobacter_sphaeroides_WS8N_
Parvularcula_bermudensis_HTCC2503_
Phenylobacterium_zucineum_HLK1_
Caulobacter_crescentus_CB15_
Asticcacaulis_excentricus_CB_48_
Brevundimonas_subvibrioides_ATCC_15264_
Hyphomonas_neptunium_ATCC_15444_
Hirschia_baltica_ATCC_49814_
Maricaulis_maris_MCS10_
Zymomonas_mobilis_mobilis_NCIMB_11163_
Sphingomonas_wittichii_RW1_
Sphingobium_japonicum_UT26S_
Sphingopyxis_alaskensis_RB2256_
Novosphingobium_nitrogenifigens_DSM_19370_
Erythrobacter_litoralis_HTCC2594_
Bartonella_quintana_Toulouse_
Bartonella_henselae_Houston1_
Bartonella_tribocorum_CIP_105476_
Bartonella_grahamii_as4aup_
Bartonella_clarridgeiae_73_
Brucella_melitensis_M28_
Ochrobactrum_anthropi_ATCC_49188_
Chelativorans_BNC1_
Mesorhizobium_loti_MAFF303099_
Fulvimarina_pelagi_HTCC2506_
Sinorhizobium_meliloti_AK83_
Rhizobium_leguminosarum_bv_trifolii_WSM1325_
Agrobacterium_H133_
Agrobacterium_ATCC_31749_
Hoeflea_phototrophica_DFL43_
Ahrensia_R2A130_
Labrenzia_alexandrii_DFL11_
Afipia_1NLS2_
Oligotropha_carboxidovorans_OM5_
Nitrobacter_hamburgensis_X14_
Bradyrhizobium_japonicum_USDA_110_
Rhodopseudomonas_palustris_BisB5_
Methylobacterium_extorquens_AM1_
Starkeya_novella_DSM_506_
Xanthobacter_autotrophicus_Py2_
Methylosinus_trichosporium_OB3b_
Beijerinckia_indica_indica_ATCC_9039_
Parvibaculum_lavamentivorans_DS1_
Hyphomicrobium_denitrificans_ATCC_51888_
Rhodomicrobium_vannielii_ATCC_17100_
Azospirillum_B510_
Rhodocista_centenaria_SW_
Acetobacter_pasteurianus_IFO_328301_
Acidiphilium_multivorum_AIU301_
Magnetospirillum_magneticum_AMB1_
Candidatus_Puniceispirillum_marinum_IMCC1322_
Anaplasma_marginale_Puerto_Rico_
Ehrlichia_canis_Jake_
Wolbachia_of_Drosophila_melanogaster_
Neorickettsia_sennetsu_Miyayama_
Rickettsia_typhi_Wilmington_
Orientia_tsutsugamushi_Ikeda_
Candidatus_Midichloria_mitochondrii_IricVA_
Pelagibacteraceae_IMCC9063_
Candidatus_Pelagibacter_ubique_HTCC1002_
Pelagibacteraceae_HIMB59_
Caedibacter_caryophilus_221_
Candidatus_Odyssella_thessalonicensis_L13_
Magnetococcus_MC1_
Escherichia_coli_MG1655_
Ralstonia_solanacearum_GMI1000_
0.1

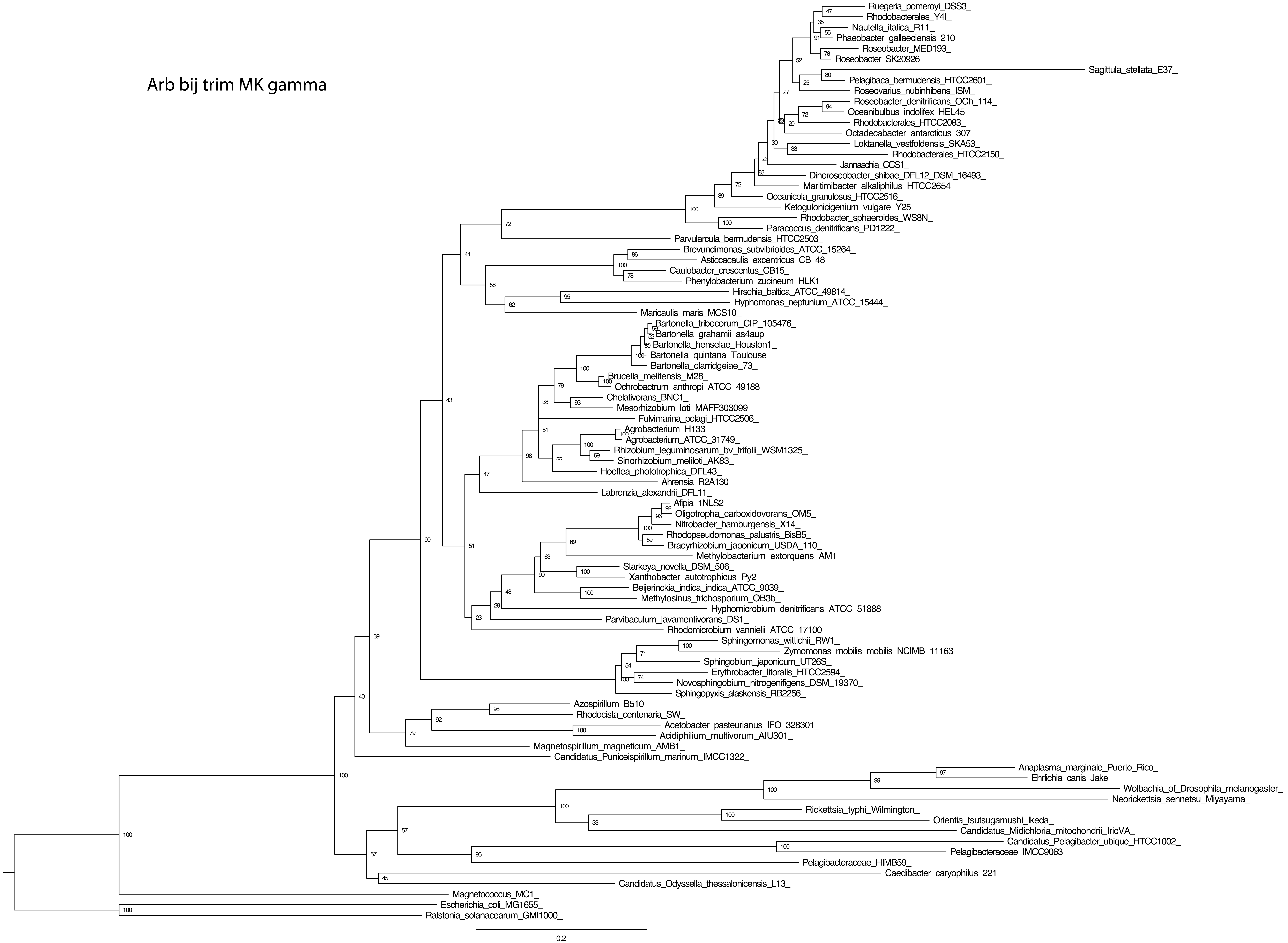
Arb bij trim MK gamma
Ruegeria_pomeroyi_DSS3_
Rhodobacterales_Y4I_
Nautella_italica_R11_
Phaeobacter_gallaeciensis_210_
Roseobacter_MED193_
Roseobacter_SK20926_
Sagittula_stellata_E37_
Pelagibaca_bermudensis_HTCC2601_
Roseovarius_nubinhibens_ISM_
Roseobacter_denitrificans_OCh_114_
Oceanibulbus_indolifex_HEL45_
Rhodobacterales_HTCC2083_
Octadecabacter_antarcticus_307_
Loktanella_vestfoldensis_SKA53_
Rhodobacterales_HTCC2150_
Jannaschia_CCS1_
Dinoroseobacter_shibae_DFL12_DSM_16493_
Maritimibacter_alkaliphilus_HTCC2654_
Oceanicola_granulosus_HTCC2516_
Ketogulonicigenium_vulgare_Y25_
Rhodobacter_sphaeroides_WS8N_
Paracoccus_denitrificans_PD1222_
Parvularcula_bermudensis_HTCC2503_
Brevundimonas_subvibrioides_ATCC_15264_
Asticcacaulis_excentricus_CB_48_
Caulobacter_crescentus_CB15_
Phenylobacterium_zucineum_HLK1_
Hirschia_baltica_ATCC_49814_
Hyphomonas_neptunium_ATCC_15444_
Maricaulis_maris_MCS10_
Bartonella_tribocorum_CIP_105476_
Bartonella_grahamii_as4aup_
Bartonella_henselae_Houston1_
Bartonella_quintana_Toulouse_
Bartonella_clarridgeiae_73_
Brucella_melitensis_M28_
Ochrobactrum_anthropi_ATCC_49188_
Chelativorans_BNC1_
Mesorhizobium_loti_MAFF303099_
Fulvimarina_pelagi_HTCC2506_
Agrobacterium_H133_
Agrobacterium_ATCC_31749_
Rhizobium_leguminosarum_bv_trifolii_WSM1325_
Sinorhizobium_meliloti_AK83_
Hoeflea_phototrophica_DFL43_
Ahrensia_R2A130_
Labrenzia_alexandrii_DFL11_
Afipia_1NLS2_
Oligotropha_carboxidovorans_OM5_
Nitrobacter_hamburgensis_X14_
Rhodopseudomonas_palustris_BisB5_
Bradyrhizobium_japonicum_USDA_110_
Methylobacterium_extorquens_AM1_
Starkeya_novella_DSM_506_
Xanthobacter_autotrophicus_Py2_
Beijerinckia_indica_indica_ATCC_9039_
Methylosinus_trichosporium_OB3b_
Hyphomicrobium_denitrificans_ATCC_51888_
Parvibaculum_lavamentivorans_DS1_
Rhodomicrobium_vannielii_ATCC_17100_
Sphingomonas_wittichii_RW1_
Zymomonas_mobilis_mobilis_NCIMB_11163_
Sphingobium_japonicum_UT26S_
Erythrobacter_litoralis_HTCC2594_
Novosphingobium_nitrogenifigens_DSM_19370_
Sphingopyxis_alaskensis_RB2256_
Azospirillum_B510_
Rhodocista_centenaria_SW_
Acetobacter_pasteurianus_IFO_328301_
Acidiphilium_multivorum_AIU301_
Magnetospirillum_magneticum_AMB1_
Candidatus_Puniceispirillum_marinum_IMCC1322_
Anaplasma_marginale_Puerto_Rico_
Ehrlichia_canis_Jake_
Wolbachia_of_Drosophila_melanogaster_
Neorickettsia_sennetsu_Miyayama_
Rickettsia_typhi_Wilmington_
Orientia_tsutsugamushi_Ikeda_
Candidatus_Midichloria_mitochondrii_IricVA_
Candidatus_Pelagibacter_ubique_HTCC1002_
Pelagibacteraceae_IMCC9063_
Pelagibacteraceae_HIMB59_
Caedibacter_caryophilus_221_
Candidatus_Odyssella_thessalonicensis_L13_
Magnetococcus_MC1_
Escherichia_coli_MG1655_
Ralstonia_solanacearum_GMI1000_
0.2

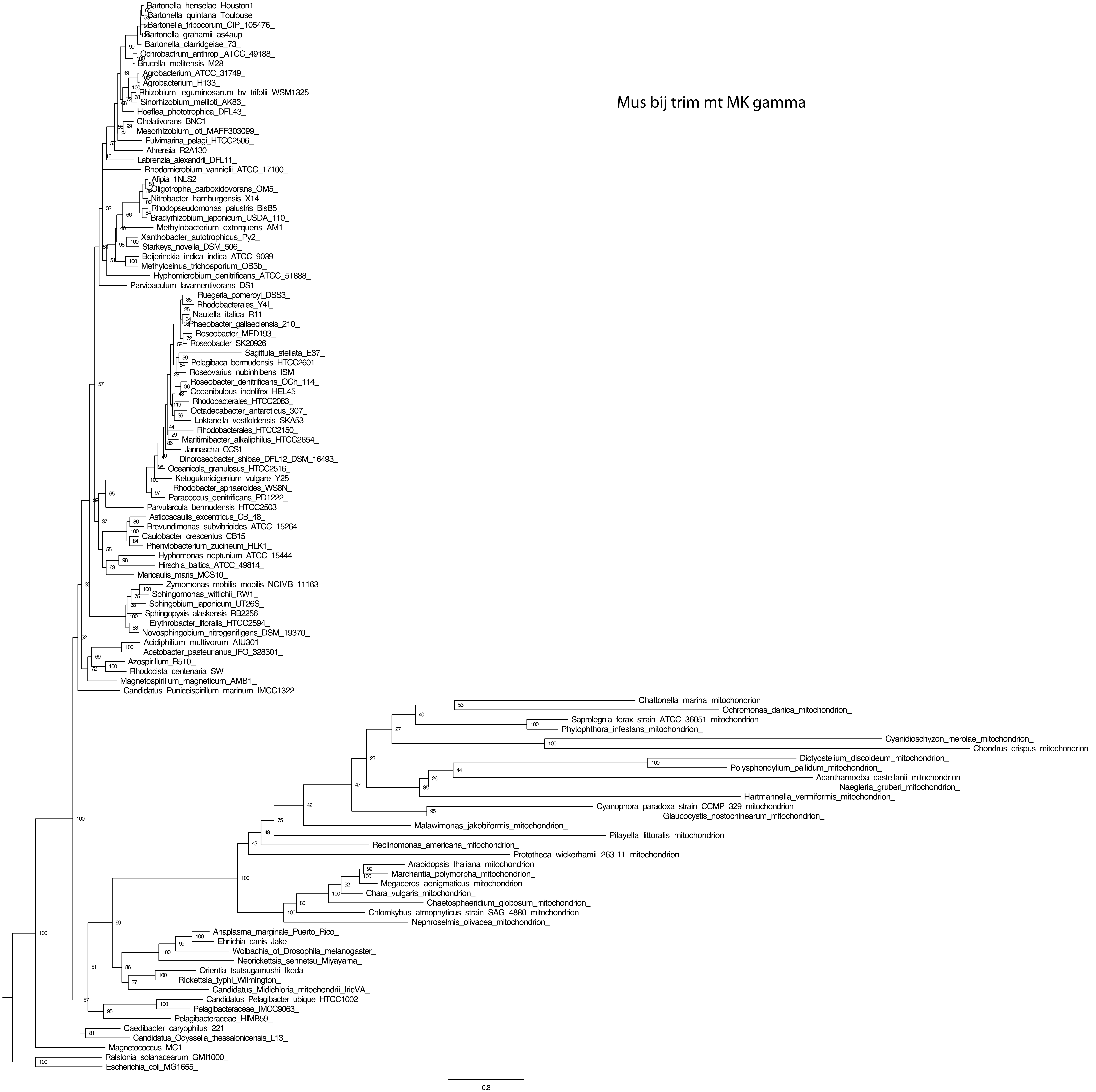

Mus bij trim mt MK gamma
Bartonella_henselae_Houston1_
Bartonella_quintana_Toulouse_
Bartonella_tribocorum_CIP_105476_
Bartonella_grahamii_as4aup_
Bartonella_clarridgeiae_73_
Ochrobactrum_anthropi_ATCC_49188_
Brucella_melitensis_M28_
Agrobacterium_ATCC_31749_
Agrobacterium_H133_
Rhizobium_leguminosarum_bv_trifolii_WSM1325_
Sinorhizobium_meliloti_AK83_
Hoeflea_phototrophica_DFL43_
Chelativorans_BNC1_
Mesorhizobium_loti_MAFF303099_
Fulvimarina_pelagi_HTCC2506_
Ahrensia_R2A130_
Labrenzia_alexandrii_DFL11_
Rhodomicrobium_vannielii_ATCC_17100_
Afipia_1NLS2_
Oligotropha_carboxidovorans_OM5_
Nitrobacter_hamburgensis_X14_
Rhodopseudomonas_palustris_BisB5_
Bradyrhizobium_japonicum_USDA_110_
Methylobacterium_extorquens_AM1_
Xanthobacter_autotrophicus_Py2_
Starkeya_novella_DSM_506_
Beijerinckia_indica_indica_ATCC_9039_
Methylosinus_trichosporium_OB3b_
Hyphomicrobium_denitrificans_ATCC_51888_
Parvibaculum_lavamentivorans_DS1_
Ruegeria_pomeroyi_DSS3_
Rhodobacterales_Y4I_
Nautella_italica_R11_
Phaeobacter_gallaeciensis_210_
Roseobacter_MED193_
Roseobacter_SK20926_
Sagittula_stellata_E37_
Pelagibaca_bermudensis_HTCC2601_
Roseovarius_nubinhibens_ISM_
Roseobacter_denitrificans_OCh_114_
Oceanibulbus_indolifex_HEL45_
Rhodobacterales_HTCC2083_
Octadecabacter_antarcticus_307_
Loktanella_vestfoldensis_SKA53_
Rhodobacterales_HTCC2150_
Maritimibacter_alkaliphilus_HTCC2654_
Jannaschia_CCS1_
Dinoroseobacter_shibae_DFL12_DSM_16493_
Oceanicola_granulosus_HTCC2516_
Ketogulonicigenium_vulgare_Y25_
Rhodobacter_sphaeroides_WS8N_
Paracoccus_denitrificans_PD1222_
Parvularcula_bermudensis_HTCC2503_
Asticcacaulis_excentricus_CB_48_
Brevundimonas_subvibrioides_ATCC_15264_
Caulobacter_crescentus_CB15_
Phenylobacterium_zucineum_HLK1_
Hyphomonas_neptunium_ATCC_15444_
Hirschia_baltica_ATCC_49814_
Maricaulis_maris_MCS10_
Zymomonas_mobilis_mobilis_NCIMB_11163_
Sphingomonas_wittichii_RW1_
Sphingobium_japonicum_UT26S_
Sphingopyxis_alaskensis_RB2256_
Erythrobacter_litoralis_HTCC2594_
Novosphingobium_nitrogenifigens_DSM_19370_
Acidiphilium_multivorum_AIU301_
Acetobacter_pasteurianus_IFO_328301_
Azospirillum_B510_
Rhodocista_centenaria_SW_
Magnetospirillum_magneticum_AMB1_
Candidatus_Puniceispirillum_marinum_IMCC1322_
Chattonella_marina_mitochondrion_
Ochromonas_danica_mitochondrion_
Saprolegnia_ferax_strain_ATCC_36051_mitochondrion_
Phytophthora_infestans_mitochondrion_
Cyanidioschyzon_merolae_mitochondrion_
Chondrus_crispus_mitochondrion_
Dictyostelium_discoideum_mitochondrion_
Polysphondylium_pallidum_mitochondrion_
Acanthamoeba_castellanii_mitochondrion_
Naegleria_gruberi_mitochondrion_
Hartmannella_vermiformis_mitochondrion_
Cyanophora_paradoxa_strain_CCMP_329_mitochondrion_
Glaucocystis_nostochinearum_mitochondrion_
Malawimonas_jakobiformis_mitochondrion_
Pilayella_littoralis_mitochondrion_
Reclinomonas_americana_mitochondrion_
Prototheca_wickerhamii_263-11_mitochondrion_
Arabidopsis_thaliana_mitochondrion_
Marchantia_polymorpha_mitochondrion_
Megaceros_aenigmaticus_mitochondrion_
Chara_vulgaris_mitochondrion_
Chaetosphaeridium_globosum_mitochondrion_
Chlorokybus_atmophyticus_strain_SAG_4880_mitochondrion_
Nephroselmis_olivacea_mitochondrion_
Anaplasma_marginale_Puerto_Rico_
Ehrlichia_canis_Jake_
Wolbachia_of_Drosophila_melanogaster_
Neorickettsia_sennetsu_Miyayama_
Orientia_tsutsugamushi_Ikeda_
Rickettsia_typhi_Wilmington_
Candidatus_Midichloria_mitochondrii_IricVA_
Candidatus_Pelagibacter_ubique_HTCC1002_
Pelagibacteraceae_IMCC9063_
Pelagibacteraceae_HIMB59_
Caedibacter_caryophilus_221_
Candidatus_Odyssella_thessalonicensis_L13_
Magnetococcus_MC1_
Ralstonia_solanacearum_GMI1000_
Escherichia_coli_MG1655_
0.3

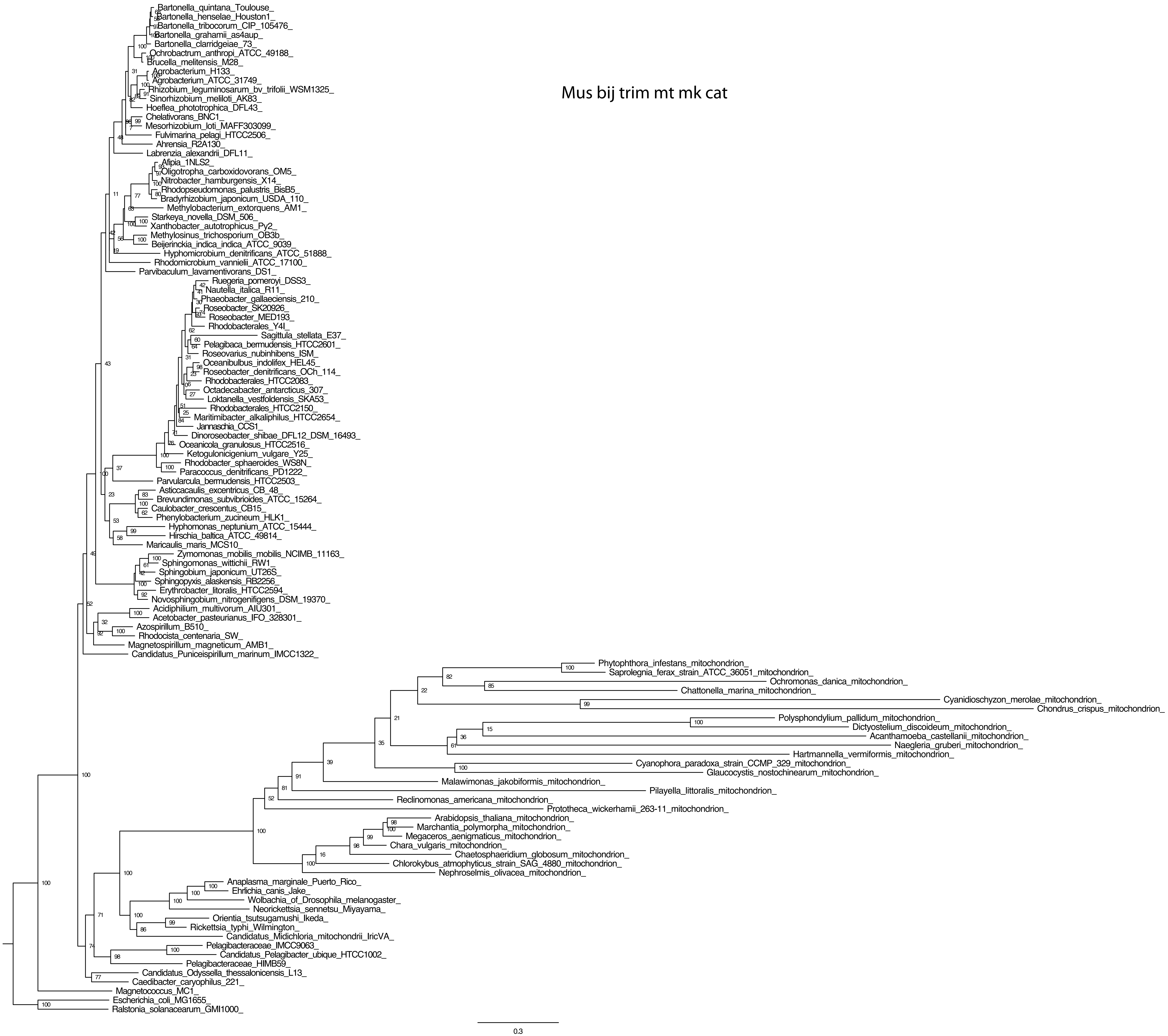
Mus bij trim mt mk cat
Bartonella_quintana_Toulouse_
Bartonella_henselae_Houston1_
Bartonella_tribocorum_CIP_105476_
Bartonella_grahamii_as4aup_
Bartonella_clarridgeiae_73_
Ochrobactrum_anthropi_ATCC_49188_
Brucella_melitensis_M28_
Agrobacterium_H133_
Agrobacterium_ATCC_31749_
Rhizobium_leguminosarum_bv_trifolii_WSM1325_
Sinorhizobium_meliloti_AK83_
Hoeflea_phototrophica_DFL43_
Chelativorans_BNC1_
Mesorhizobium_loti_MAFF303099_
Fulvimarina_pelagi_HTCC2506_
Ahrensia_R2A130_
Labrenzia_alexandrii_DFL11_
Afipia_1NLS2_
Oligotropha_carboxidovorans_OM5_
Nitrobacter_hamburgensis_X14_
Rhodopseudomonas_palustris_BisB5_
Bradyrhizobium_japonicum_USDA_110_
Methylobacterium_extorquens_AM1_
Starkeya_novella_DSM_506_
Xanthobacter_autotrophicus_Py2_
Methylosinus_trichosporium_OB3b_
Beijerinckia_indica_indica_ATCC_9039_
Hyphomicrobium_denitrificans_ATCC_51888_
Rhodomicrobium_vannielii_ATCC_17100_
Parvibaculum_lavamentivorans_DS1_
Ruegeria_pomeroyi_DSS3_
Nautella_italica_R11_
Phaeobacter_gallaeciensis_210_
Roseobacter_SK20926_
Roseobacter_MED193_
Rhodobacterales_Y4I_
Sagittula_stellata_E37_
Pelagibaca_bermudensis_HTCC2601_
Roseovarius_nubinhibens_ISM_
Oceanibulbus_indolifex_HEL45_
Roseobacter_denitrificans_OCh_114_
Rhodobacterales_HTCC2083_
Octadecabacter_antarcticus_307_
Loktanella_vestfoldensis_SKA53_
Rhodobacterales_HTCC2150_
Maritimibacter_alkaliphilus_HTCC2654_
Jannaschia_CCS1_
Dinoroseobacter_shibae_DFL12_DSM_16493_
Oceanicola_granulosus_HTCC2516_
Ketogulonicigenium_vulgare_Y25_
Rhodobacter_sphaeroides_WS8N_
Paracoccus_denitrificans_PD1222_
Parvularcula_bermudensis_HTCC2503_
Asticcacaulis_excentricus_CB_48_
Brevundimonas_subvibrioides_ATCC_15264_
Caulobacter_crescentus_CB15_
Phenylobacterium_zucineum_HLK1_
Hyphomonas_neptunium_ATCC_15444_
Hirschia_baltica_ATCC_49814_
Maricaulis_maris_MCS10_
Zymomonas_mobilis_mobilis_NCIMB_11163_
Sphingomonas_wittichii_RW1_
Sphingobium_japonicum_UT26S_
Sphingopyxis_alaskensis_RB2256_
Erythrobacter_litoralis_HTCC2594_
Novosphingobium_nitrogenifigens_DSM_19370_
Acidiphilium_multivorum_AIU301_
Acetobacter_pasteurianus_IFO_328301_
Azospirillum_B510_
Rhodocista_centenaria_SW_
Magnetospirillum_magneticum_AMB1_
Candidatus_Puniceispirillum_marinum_IMCC1322_
Phytophthora_infestans_mitochondrion_
Saprolegnia_ferax_strain_ATCC_36051_mitochondrion_
Ochromonas_danica_mitochondrion_
Chattonella_marina_mitochondrion_
Cyanidioschyzon_merolae_mitochondrion_
Chondrus_crispus_mitochondrion_
Polysphondylium_pallidum_mitochondrion_
Dictyostelium_discoideum_mitochondrion_
Acanthamoeba_castellanii_mitochondrion_
Naegleria_gruberi_mitochondrion_
Hartmannella_vermiformis_mitochondrion_
Cyanophora_paradoxa_strain_CCMP_329_mitochondrion_
Glaucocystis_nostochinearum_mitochondrion_
Malawimonas_jakobiformis_mitochondrion_
Pilayella_littoralis_mitochondrion_
Reclinomonas_americana_mitochondrion_
Prototheca_wickerhamii_263-11_mitochondrion_
Arabidopsis_thaliana_mitochondrion_
Marchantia_polymorpha_mitochondrion_
Megaceros_aenigmaticus_mitochondrion_
Chara_vulgaris_mitochondrion_
Chaetosphaeridium_globosum_mitochondrion_
Chlorokybus_atmophyticus_strain_SAG_4880_mitochondrion_
Nephroselmis_olivacea_mitochondrion_
Anaplasma_marginale_Puerto_Rico_
Ehrlichia_canis_Jake_
Wolbachia_of_Drosophila_melanogaster_
Neorickettsia_sennetsu_Miyayama_
Orientia_tsutsugamushi_Ikeda_
Rickettsia_typhi_Wilmington_
Candidatus_Midichloria_mitochondrii_IricVA_
Pelagibacteraceae_IMCC9063_
Candidatus_Pelagibacter_ubique_HTCC1002_
Pelagibacteraceae_HIMB59_
Candidatus_Odyssella_thessalonicensis_L13_
Caedibacter_caryophilus_221_
Magnetococcus_MC1_
Escherichia_coli_MG1655_
Ralstonia_solanacearum_GMI1000_
0.3

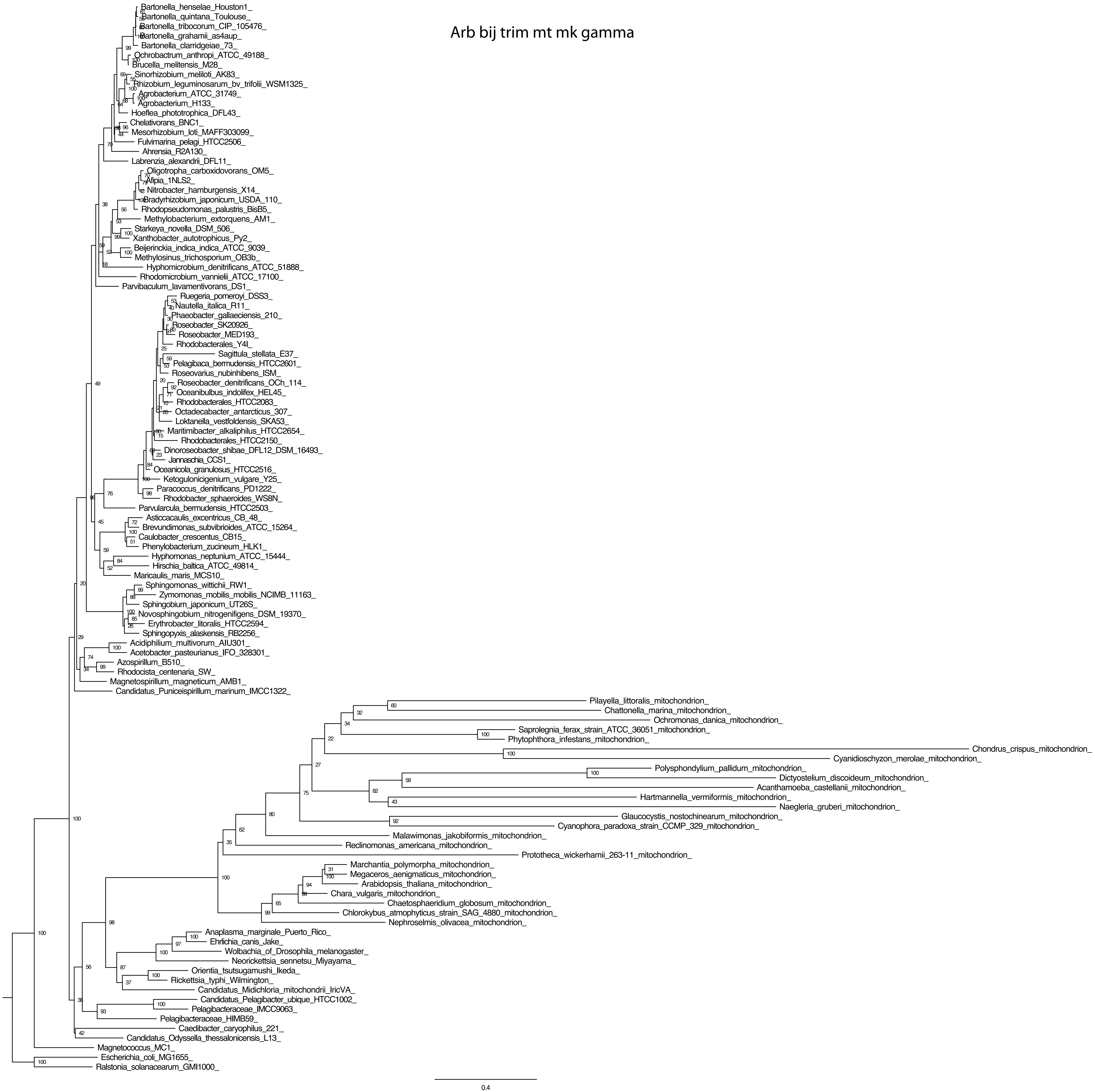
Arb bij trim mt mk gamma
Bartonella_henselae_Houston1_
Bartonella_quintana_Toulouse_
Bartonella_tribocorum_CIP_105476_
Bartonella_grahamii_as4aup_
Bartonella_clarridgeiae_73_
Ochrobactrum_anthropi_ATCC_49188_
Brucella_melitensis_M28_
Sinorhizobium_meliloti_AK83_
Rhizobium_leguminosarum_bv_trifolii_WSM1325_
Agrobacterium_ATCC_31749_
Agrobacterium_H133_
Hoeflea_phototrophica_DFL43_
Chelativorans_BNC1_
Mesorhizobium_loti_MAFF303099_
Fulvimarina_pelagi_HTCC2506_
Ahrensia_R2A130_
Labrenzia_alexandrii_DFL11_
Oligotropha_carboxidovorans_OM5_
Afipia_1NLS2_
Nitrobacter_hamburgensis_X14_
Bradyrhizobium_japonicum_USDA_110_
Rhodopseudomonas_palustris_BisB5_
Methylobacterium_extorquens_AM1_
Starkeya_novella_DSM_506_
Xanthobacter_autotrophicus_Py2_
Beijerinckia_indica_indica_ATCC_9039_
Methylosinus_trichosporium_OB3b_
Hyphomicrobium_denitrificans_ATCC_51888_
Rhodomicrobium_vannielii_ATCC_17100_
Parvibaculum_lavamentivorans_DS1_
Ruegeria_pomeroyi_DSS3_
Nautella_italica_R11_
Phaeobacter_gallaeciensis_210_
Roseobacter_SK20926_
Roseobacter_MED193_
Rhodobacterales_Y4I_
Sagittula_stellata_E37_
Pelagibaca_bermudensis_HTCC2601_
Roseovarius_nubinhibens_ISM_
Roseobacter_denitrificans_OCh_114_
Oceanibulbus_indolifex_HEL45_
Rhodobacterales_HTCC2083_
Octadecabacter_antarcticus_307_
Loktanella_vestfoldensis_SKA53_
Maritimibacter_alkaliphilus_HTCC2654_
Rhodobacterales_HTCC2150_
Dinoroseobacter_shibae_DFL12_DSM_16493_
Jannaschia_CCS1_
Oceanicola_granulosus_HTCC2516_
Ketogulonicigenium_vulgare_Y25_
Paracoccus_denitrificans_PD1222_
Rhodobacter_sphaeroides_WS8N_
Parvularcula_bermudensis_HTCC2503_
Asticcacaulis_excentricus_CB_48_
Brevundimonas_subvibrioides_ATCC_15264_
Caulobacter_crescentus_CB15_
Phenylobacterium_zucineum_HLK1_
Hyphomonas_neptunium_ATCC_15444_
Hirschia_baltica_ATCC_49814_
Maricaulis_maris_MCS10_
Sphingomonas_wittichii_RW1_
Zymomonas_mobilis_mobilis_NCIMB_11163_
Sphingobium_japonicum_UT26S_
Novosphingobium_nitrogenifigens_DSM_19370_
Erythrobacter_litoralis_HTCC2594_
Sphingopyxis_alaskensis_RB2256_
Acidiphilium_multivorum_AIU301_
Acetobacter_pasteurianus_IFO_328301_
Azospirillum_B510_
Rhodocista_centenaria_SW_
Magnetospirillum_magneticum_AMB1_
Candidatus_Puniceispirillum_marinum_IMCC1322_
Pilayella_littoralis_mitochondrion_
Chattonella_marina_mitochondrion_
Ochromonas_danica_mitochondrion_
Saprolegnia_ferax_strain_ATCC_36051_mitochondrion_
Phytophthora_infestans_mitochondrion_
Chondrus_crispus_mitochondrion_
Cyanidioschyzon_merolae_mitochondrion_
Polysphondylium_pallidum_mitochondrion_
Dictyostelium_discoideum_mitochondrion_
Acanthamoeba_castellanii_mitochondrion_
Hartmannella_vermiformis_mitochondrion_
Naegleria_gruberi_mitochondrion_
Glaucocystis_nostochinearum_mitochondrion_
Cyanophora_paradoxa_strain_CCMP_329_mitochondrion_
Malawimonas_jakobiformis_mitochondrion_
Reclinomonas_americana_mitochondrion_
Prototheca_wickerhamii_263-11_mitochondrion_
Marchantia_polymorpha_mitochondrion_
Megaceros_aenigmaticus_mitochondrion_
Arabidopsis_thaliana_mitochondrion_
Chara_vulgaris_mitochondrion_
Chaetosphaeridium_globosum_mitochondrion_
Chlorokybus_atmophyticus_strain_SAG_4880_mitochondrion_
Nephroselmis_olivacea_mitochondrion_
Anaplasma_marginale_Puerto_Rico_
Ehrlichia_canis_Jake_
Wolbachia_of_Drosophila_melanogaster_
Neorickettsia_sennetsu_Miyayama_
Orientia_tsutsugamushi_Ikeda_
Rickettsia_typhi_Wilmington_
Candidatus_Midichloria_mitochondrii_IricVA_
Candidatus_Pelagibacter_ubique_HTCC1002_
Pelagibacteraceae_IMCC9063_
Pelagibacteraceae_HIMB59_
Caedibacter_caryophilus_221_
Candidatus_Odyssella_thessalonicensis_L13_
Magnetococcus_MC1_
Escherichia_coli_MG1655_
Ralstonia_solanacearum_GMI1000_
0.4

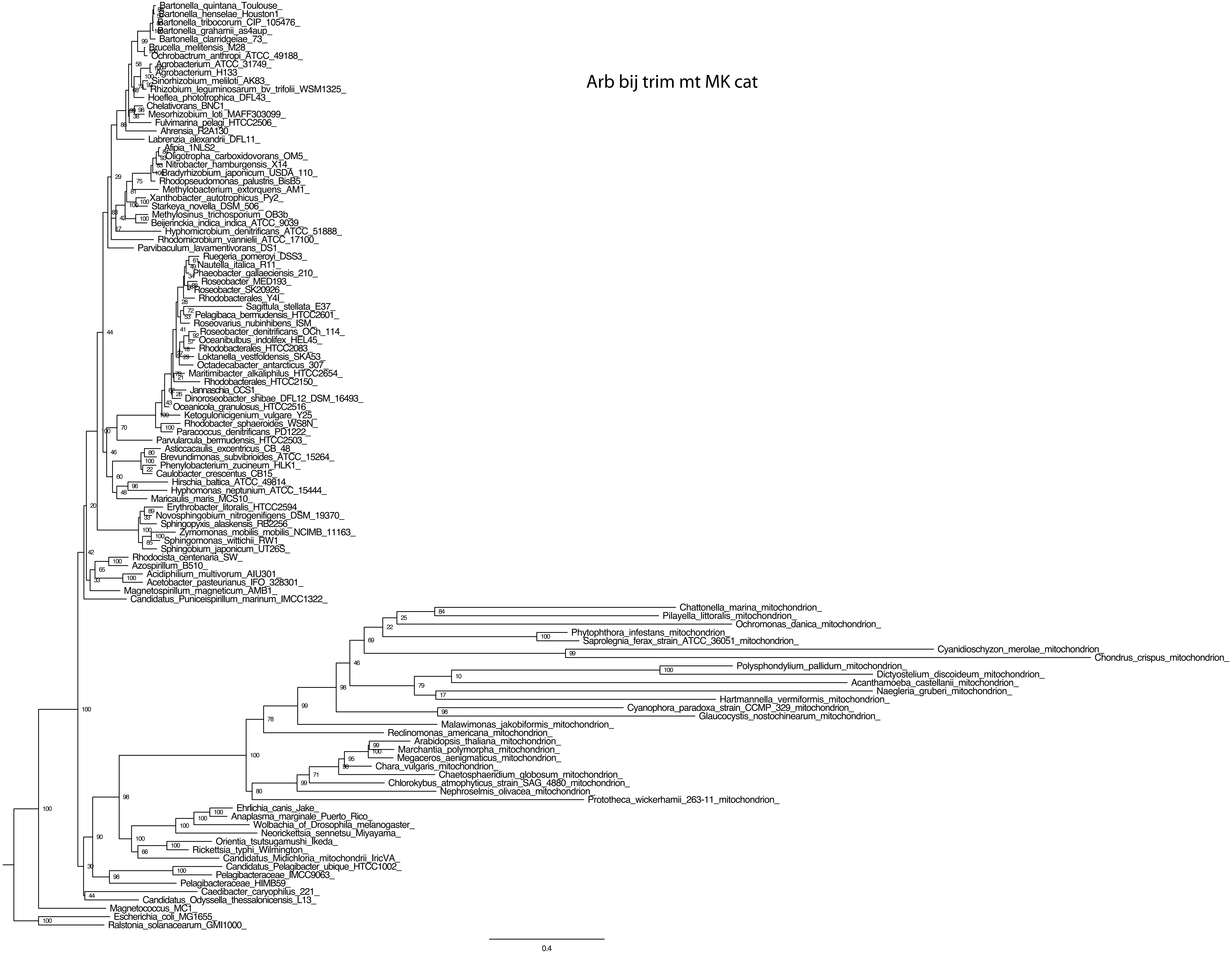
Arb bij trim mt MK cat
Bartonella_quintana_Toulouse_
Bartonella_henselae_Houston1_
Bartonella_tribocorum_CIP_105476_
Bartonella_grahamii_as4aup_
Bartonella_clarridgeiae_73_
Brucella_melitensis_M28_
Ochrobactrum_anthropi_ATCC_49188_
Agrobacterium_ATCC_31749_
Agrobacterium_H133_
Sinorhizobium_meliloti_AK83_
Rhizobium_leguminosarum_bv_trifolii_WSM1325_
Hoeflea_phototrophica_DFL43_
Chelativorans_BNC1_
Mesorhizobium_loti_MAFF303099_
Fulvimarina_pelagi_HTCC2506_
Ahrensia_R2A130_
Labrenzia_alexandrii_DFL11_
Afipia_1NLS2_
Oligotropha_carboxidovorans_OM5_
Nitrobacter_hamburgensis_X14_
Bradyrhizobium_japonicum_USDA_110_
Rhodopseudomonas_palustris_BisB5_
Methylobacterium_extorquens_AM1_
Xanthobacter_autotrophicus_Py2_
Starkeya_novella_DSM_506_
Methylosinus_trichosporium_OB3b_
Beijerinckia_indica_indica_ATCC_9039_
Hyphomicrobium_denitrificans_ATCC_51888_
Rhodomicrobium_vannielii_ATCC_17100_
Parvibaculum_lavamentivorans_DS1_
Ruegeria_pomeroyi_DSS3_
Nautella_italica_R11_
Phaeobacter_gallaeciensis_210_
Roseobacter_MED193_
Roseobacter_SK20926_
Rhodobacterales_Y4I_
Sagittula_stellata_E37_
Pelagibaca_bermudensis_HTCC2601_
Roseovarius_nubinhibens_ISM_
Roseobacter_denitrificans_OCh_114_
Oceanibulbus_indolifex_HEL45_
Rhodobacterales_HTCC2083_
Loktanella_vestfoldensis_SKA53_
Octadecabacter_antarcticus_307_
Maritimibacter_alkaliphilus_HTCC2654_
Rhodobacterales_HTCC2150_
Jannaschia_CCS1_
Dinoroseobacter_shibae_DFL12_DSM_16493_
Oceanicola_granulosus_HTCC2516_
Ketogulonicigenium_vulgare_Y25_
Rhodobacter_sphaeroides_WS8N_
Paracoccus_denitrificans_PD1222_
Parvularcula_bermudensis_HTCC2503_
Asticcacaulis_excentricus_CB_48_
Brevundimonas_subvibrioides_ATCC_15264_
Phenylobacterium_zucineum_HLK1_
Caulobacter_crescentus_CB15_
Hirschia_baltica_ATCC_49814_
Hyphomonas_neptunium_ATCC_15444_
Maricaulis_maris_MCS10_
Erythrobacter_litoralis_HTCC2594_
Novosphingobium_nitrogenifigens_DSM_19370_
Sphingopyxis_alaskensis_RB2256_
Zymomonas_mobilis_mobilis_NCIMB_11163_
Sphingomonas_wittichii_RW1_
Sphingobium_japonicum_UT26S_
Rhodocista_centenaria_SW_
Azospirillum_B510_
Acidiphilium_multivorum_AIU301_
Acetobacter_pasteurianus_IFO_328301_
Magnetospirillum_magneticum_AMB1_
Candidatus_Puniceispirillum_marinum_IMCC1322_
Chattonella_marina_mitochondrion_
Pilayella_littoralis_mitochondrion_
Ochromonas_danica_mitochondrion_
Phytophthora_infestans_mitochondrion_
Saprolegnia_ferax_strain_ATCC_36051_mitochondrion_
Cyanidioschyzon_merolae_mitochondrion_
Chondrus_crispus_mitochondrion_
Polysphondylium_pallidum_mitochondrion_
Dictyostelium_discoideum_mitochondrion_
Acanthamoeba_castellanii_mitochondrion_
Naegleria_gruberi_mitochondrion_
Hartmannella_vermiformis_mitochondrion_
Cyanophora_paradoxa_strain_CCMP_329_mitochondrion_
Glaucocystis_nostochinearum_mitochondrion_
Malawimonas_jakobiformis_mitochondrion_
Reclinomonas_americana_mitochondrion_
Arabidopsis_thaliana_mitochondrion_
Marchantia_polymorpha_mitochondrion_
Megaceros_aenigmaticus_mitochondrion_
Chara_vulgaris_mitochondrion_
Chaetosphaeridium_globosum_mitochondrion_
Chlorokybus_atmophyticus_strain_SAG_4880_mitochondrion_
Nephroselmis_olivacea_mitochondrion_
Prototheca_wickerhamii_263-11_mitochondrion_
Ehrlichia_canis_Jake_
Anaplasma_marginale_Puerto_Rico_
Wolbachia_of_Drosophila_melanogaster_
Neorickettsia_sennetsu_Miyayama_
Orientia_tsutsugamushi_Ikeda_
Rickettsia_typhi_Wilmington_
Candidatus_Midichloria_mitochondrii_IricVA_
Candidatus_Pelagibacter_ubique_HTCC1002_
Pelagibacteraceae_IMCC9063_
Pelagibacteraceae_HIMB59_
Caedibacter_caryophilus_221_
Candidatus_Odyssella_thessalonicensis_L13_
Magnetococcus_MC1_
Escherichia_coli_MG1655_
Ralstonia_solanacearum_GMI1000_
0.4
