# Supplementary material for: New rRNA Gene-Based Phylogenies of the Alphaproteobacteria Provide Perspective on Major Groups, Mitochondrial Ancestry and Phylogenetic Instability
Source: PLoS One. 2013 Dec 11;8(12):e83383. doi: 10.1371/journal.pone.0083383 (PMC3859672; doi:10.1371/journal.pone.0083383)
Supplement: Table S1 — Summary of taxa used in this study. (DOCX) [file pone.0083383.s021.docx]

**Table S1. Summary of taxa used in this study.**

**Table S1A.** Bacterial taxa present in the ‘complete’ dataset.

| **Name** | **Higher Taxon** |
| --- | --- |
| *Asticcacaulis biprosthecum* C19 | *Caulobacteraceae* (*Caulobacterales*) |
| *Asticcacaulis excentricus CB 48* | *Caulobacteraceae* (*Caulobacterales*) |
| *Brevundimonas BAL3* | *Caulobacteraceae* (*Caulobacterales*) |
| *Brevundimonas diminuta ATCC 11568* | *Caulobacteraceae* (*Caulobacterales*) |
| *Brevundimonas subvibrioides ATCC 15264* | *Caulobacteraceae* (*Caulobacterales*) |
| *Caulobacter crescentus CB15* | *Caulobacteraceae* (*Caulobacterales*) |
| *Caulobacter K31* | *Caulobacteraceae* (*Caulobacterales*) |
| *Caulobacter segnis ATCC 21756* | *Caulobacteraceae* (*Caulobacterales*) |
| *Phenylobacterium zucineum HLK1* | *Caulobacteraceae* (*Caulobacterales*) |
| *Hirschia baltica ATCC 49814* | *Hyphomonadaceae* (*Caulobacterales*) |
| *Hyphomonas neptunium ATCC 15444* | *Hyphomonadaceae* (*Caulobacterales*) |
| *Caedibacter caryophilus 221* | *Holosporales* (*ord. nov.*) |
| *Candidatus Odyssella thessalonicensis L13* | *Holosporales* (*ord. nov.*) |
| *Magnetococcus MC1* | *Magnetococcaceae* (*Magnetococcales*) |
| *Maricaulis maris MCS10* | potentially *Caulobacterales* (*v.* table S2E) |
| *Acidithiobacillus ferrooxidans ATCC 23270* | outgroup *— Acidithiobacillia* (*Proteobacteria*) |
| *Campylobacter jejuni ICDCCJ07001* | outgroup *— Epsilonproteobacteria* |
| *Escherichia coli MG1655* | outgroup *— Gammaproteobacteria* |
| *Mariprofundus ferrooxydans PV1* | outgroup *— Zetaproteobacteria* |
| *Ralstonia solanacearum GMI1000* | outgroup *— Betaproteobacteria* |
| *Thiobacillus denitrificans ATCC 25259* | outgroup *— Betaproteobacteria* |
| *Xanthomonas campestris 8004* | outgroup *—Gammaproteobacteria* |
| *Parvibaculum lavamentivorans DS1* | *Rhizobiales* (*v.* table S2E) |
| *Parvularcula bermudensis HTCC2503* | *Parvularculales* |
| *Pelagibacteraceae 313* | *Pelagibacterales* subclade II |
| *Pelagibacteraceae 440* | *Pelagibacterales* subclade II |
| *Pelagibacteraceae HIMB59* | *Pelagibacterales* subclade V |
| *Pelagibacteraceae IMCC9063* | *Pelagibacterales* subclade IIIa |
| *Candidatus Pelagibacter ubique HTCC1002* | *Pelagibacterales* subclade Ia |
| *Pelagibacteraceae HIMB5B* | *Pelagibacterales* subclade Ia |
| *Candidatus Pelagibacter HTCC7211* | *Pelagibacterales* subclade Ia |
| *Pelagibacteraceae HIMB114* | *Pelagibacterales* subclade IIIa |
| *Bartonella bacilliformis KC583* | *Bartonellaceae* (*Rhizobiales*) |
| *Bartonella clarridgeiae 73* | *Bartonellaceae* (*Rhizobiales*) |
| *Bartonella grahamii as4aup* | *Bartonellaceae* (*Rhizobiales*) |
| *Bartonella henselae Houston1* | *Bartonellaceae* (*Rhizobiales*) |
| *Bartonella quintana Toulouse* | *Bartonellaceae* (*Rhizobiales*) |
| *Bartonella tribocorum CIP 105476* | *Bartonellaceae* (*Rhizobiales*) |
| *Beijerinckia indica indica ATCC 9039* | *Beijerinckiaceae* (*Rhizobiales*) |
| *Afipia 1NLS2* | *Bradyrhizobiaceae* (*Rhizobiales*) |
| *Bradyrhizobiaceae SG6C* | *Bradyrhizobiaceae* (*Rhizobiales*) |
| *Bradyrhizobium BTAi1* | *Bradyrhizobiaceae* (*Rhizobiales*) |
| *Bradyrhizobium japonicum USDA 110* | *Bradyrhizobiaceae* (*Rhizobiales*) |
| *Bradyrhizobium ORS278* | *Bradyrhizobiaceae* (*Rhizobiales*) |
| *Brucella melitensis M28* | *Brucellaceae* (*Rhizobiales*) |
| *Ahrensia R2A130* | potentially *Rhizobiales* (*v.* table S2E) |
| *Labrenzia aggregata IAM 12614* | potentially *Rhizobiales* (*v.* table S2E) |
| *Labrenzia alexandrii DFL11* | potentially *Rhizobiales* (*v.* table S2E) |
| *Roseibium TrichSKD4* | potentially *Rhizobiales* (*v.* table S2E) |
| *Agrobacterium ATCC 31749* | *Rhizobiaceae* (*Rhizobiales*) |
| *Agrobacterium H133* | *Rhizobiaceae* (*Rhizobiales*) |
| *Candidatus Liberibacter solanacearum CLsoZC1* | *Rhizobiaceae* (*Rhizobiales*) |
| *Chelativorans BNC1* | *Phyllobacteriaceae* (*Rhizobiales*) |
| *Ensifer medicae WSM419* | *Rhizobiaceae* (*Rhizobiales*) |
| *Ensifer meliloti BL225C* | *Rhizobiaceae* (*Rhizobiales*) |
| *Fulvimarina pelagi HTCC2506* | *Rhizobiales* |
| *Hoeflea phototrophica DFL43* | *Phyllobacteriaceae* (*Rhizobiales*) |
| *Hyphomicrobium denitrificans ATCC 51888* | *Hyphomicrobiaceae* (*Rhizobiales*) |
| *Hyphomicrobium MC1* | *Hyphomicrobiaceae* (*Rhizobiales*) |
| *Mesorhizobium ciceri bv biserrulae WSM1271* | *Phyllobacteriaceae* (*Rhizobiales*) |
| *Mesorhizobium loti MAFF303099* | *Phyllobacteriaceae* (*Rhizobiales*) |
| *Mesorhizobium opportunistum WSM2075* | *Phyllobacteriaceae* (*Rhizobiales*) |
| *Methylobacterium 446* | *Methylobacteriaceae* (*Rhizobiales*) |
| *Methylobacterium chloromethanicum CM4* | *Methylobacteriaceae* (*Rhizobiales*) |
| *Methylobacterium extorquens AM1* | *Methylobacteriaceae* (*Rhizobiales*) |
| *Methylobacterium nodulans ORS 2060* | *Methylobacteriaceae* (*Rhizobiales*) |
| *Methylobacterium populi BJ001* | *Methylobacteriaceae* (*Rhizobiales*) |
| *Methylobacterium radiotolerans JCM 2831* | *Methylobacteriaceae* (*Rhizobiales*) |
| *Methylocella silvestris BL2 DSM 15510* | *Beijerinckiaceae* (*Rhizobiales*) |
| *Methylocystis Rockwell ATCC 49242* | *Methylocystaceae* (*Rhizobiales*) |
| *Methylosinus trichosporium OB3b* | *Methylocystaceae* (*Rhizobiales*) |
| *Nitrobacter hamburgensis X14* | *Bradyrhizobiaceae* (*Rhizobiales*) |
| *Nitrobacter Nb311A* | *Bradyrhizobiaceae* (*Rhizobiales*) |
| *Nitrobacter winogradskyi Nb255* | *Bradyrhizobiaceae* (*Rhizobiales*) |
| *Ochrobactrum anthropi ATCC 49188* | *Brucellaceae* (*Rhizobiales*) |
| *Ochrobactrum intermedium LMG 3301* | *Brucellaceae* (*Rhizobiales*) |
| *Oligotropha carboxidovorans OM5* | *Bradyrhizobiaceae* (*Rhizobiales*) |
| *Rhizobium etli Brasil 5* | *Rhizobiaceae* (*Rhizobiales*) |
| *Rhizobium leguminosarum bv trifolii WSM1325* | *Rhizobiaceae* (*Rhizobiales*) |
| *Rhizobium NGR234* | *Rhizobiaceae* (*Rhizobiales*) |
| *Rhizobium rhizogenes K84* | *Rhizobiaceae* (*Rhizobiales*) |
| *Rhodomicrobium vannielii ATCC 17100* | *Hyphomicrobiaceae* (*Rhizobiales*) |
| *Rhodopseudomonas palustris BisB5* | *Bradyrhizobiaceae* (*Rhizobiales*) |
| *Sinorhizobium meliloti AK83* | *Rhizobiaceae* (*Rhizobiales*) |
| *Starkeya novella DSM 506* | *Xanthobacteraceae* (*Rhizobiales*) |
| *Xanthobacter autotrophicus Py2* | *Xanthobacteraceae* (*Rhizobiales*) |
| *Azorhizobium caulinodans ORS 571* | *Xanthobacteraceae* (*Rhizobiales*) |
| *Citreicella SE45* | *Rhodobacteraceae* (*Rhodobacterales*) |
| *Dinoroseobacter shibae DFL12 DSM 16493* | *Rhodobacteraceae* (*Rhodobacterales*) |
| *Jannaschia CCS1* | *Rhodobacteraceae* (*Rhodobacterales*) |
| *Ketogulonicigenium vulgare Y25* | *Rhodobacteraceae* (*Rhodobacterales*) |
| *Loktanella CCS2* | *Rhodobacteraceae* (*Rhodobacterales*) |
| *Loktanella vestfoldensis SKA53* | *Rhodobacteraceae* (*Rhodobacterales*) |
| *Maritimibacter alkaliphilus HTCC2654* | *Rhodobacteraceae* (*Rhodobacterales*) |
| *Nautella italica R11* | *Rhodobacteraceae* (*Rhodobacterales*) |
| *Oceanibulbus indolifex HEL45* | *Rhodobacteraceae* (*Rhodobacterales*) |
| *Oceanicola granulosus HTCC2516* | *Rhodobacteraceae* (*Rhodobacterales*) |
| *Octadecabacter antarcticus 307* | *Rhodobacteraceae* (*Rhodobacterales*) |
| *Paracoccus denitrificans PD1222* | *Rhodobacteraceae* (*Rhodobacterales*) |
| *Pelagibaca bermudensis HTCC2601* | *Rhodobacteraceae* (*Rhodobacterales*) |
| *Phaeobacter gallaeciensis 210* | *Rhodobacteraceae* (*Rhodobacterales*) |
| *Rhodobacter capsulatus SB1003* | *Rhodobacteraceae* (*Rhodobacterales*) |
| *Rhodobacter sphaeroides WS8N* | *Rhodobacteraceae* (*Rhodobacterales*) |
| *Rhodobacter SW2* | *Rhodobacteraceae* (*Rhodobacterales*) |
| *Rhodobacterales HTCC2083* | *Rhodobacteraceae* (*Rhodobacterales*) |
| *Rhodobacterales HTCC2150* | *Rhodobacteraceae* (*Rhodobacterales*) |
| *Rhodobacterales Y4I* | *Rhodobacteraceae* (*Rhodobacterales*) |
| *Roseobacter denitrificans OCh 114* | *Rhodobacteraceae* (*Rhodobacterales*) |
| *Roseobacter litoralis Och 149* | *Rhodobacteraceae* (*Rhodobacterales*) |
| *Roseobacter MED193* | *Rhodobacteraceae* (*Rhodobacterales*) |
| *Roseobacter SK20926* | *Rhodobacteraceae* (*Rhodobacterales*) |
| *Roseovarius nubinhibens ISM* | *Rhodobacteraceae* (*Rhodobacterales*) |
| *Roseovarius TM1035* | *Rhodobacteraceae* (*Rhodobacterales*) |
| *Ruegeria KLH11* | *Rhodobacteraceae* (*Rhodobacterales*) |
| *Ruegeria pomeroyi DSS3* | *Rhodobacteraceae* (*Rhodobacterales*) |
| *Ruegeria TM1040* | *Rhodobacteraceae* (*Rhodobacterales*) |
| *Sagittula stellata E37* | *Rhodobacteraceae* (*Rhodobacterales*) |
| *Silicibacter lacuscaerulensis ITI1157* | *Rhodobacteraceae* (*Rhodobacterales*) |
| *Sulfitobacter GAI101* | *Rhodobacteraceae* (*Rhodobacterales*) |
| *Sulfitobacter NAS141* | *Rhodobacteraceae* (*Rhodobacterales*) |
| *Thalassiobium R2A62* | *Rhodobacteraceae* (*Rhodobacterales*) |
| *Acetobacter pasteurianus IFO 328301* | *Acetobacteraceae* (*Rhodospirillales*) |
| *Acetobacter pomorum DM001* | *Acetobacteraceae* (*Rhodospirillales*) |
| *Acetobacter tropicalis NBRC 101654* | *Acetobacteraceae* (*Rhodospirillales*) |
| *Azospirillum B510* | *Rhodospirillaceae* (*Rhodospirillales*) |
| *Gluconobacter oxydans 621H* | *Acetobacteraceae* (*Rhodospirillales*) |
| *Granulibacter bethesdensis CGDNIH1* | *Acetobacteraceae* (*Rhodospirillales*) |
| *Magnetospirillum magneticum AMB1* | *Rhodospirillaceae* (*Rhodospirillales*) |
| *Magnetospirillum magnetotacticum MS1* | *Rhodospirillaceae* (*Rhodospirillales*) |
| *Nisaea BAL199* | *Rhodospirillaceae* (*Rhodospirillales*) |
| *Rhodocista centenaria SW* | *Rhodospirillaceae* (*Rhodospirillales*) |
| *Rhodospirillum rubrum S1 ATCC 11170* | *Rhodospirillaceae* (*Rhodospirillales*) |
| *Acidiphilium cryptum JF5* | *Acetobacteraceae* (*Rhodospirillales, v.* table S2E) |
| *Acidiphilium multivorum AIU301* | *Acetobacteraceae* (*Rhodospirillales, v.* table S2E) |
| *Candidatus Puniceispirillum marinum IMCC1322* | *Acetobacteraceae* (*Rhodospirillales, v.* table S2E) |
| *Gluconacetobacter diazotrophicus PAl 5 DSM 5601* | *Acetobacteraceae* (*Rhodospirillales, v.* table S2E) |
| *Gluconacetobacter SXCC1* | *Acetobacteraceae* (*Rhodospirillales, v.* table S2E) |
| *Anaplasma centrale str Israel* | *Anaplasmatales* (*Rickettsiales*) |
| *Anaplasma marginale Puerto Rico* | *Anaplasmatales* (*Rickettsiales*) |
| *Anaplasma phagocytophilum HZ* | *Anaplasmatales* (*Rickettsiales*) |
| *Candidatus Midichloria mitochondrii IricVA* | *Midichloriaceae* (*Rickettsiales*) |
| *Ehrlichia canis Jake* | *Anaplasmataceae* (*Rickettsiales*) |
| *Ehrlichia chaffeensis Arkansas* | *Anaplasmataceae* (*Rickettsiales*) |
| *Ehrlichia ruminantium Gardel* | *Anaplasmataceae* (*Rickettsiales*) |
| *Neorickettsia risticii Illinois* | *Anaplasmataceae* (*Rickettsiales*) |
| *Neorickettsia sennetsu Miyayama* | *Anaplasmataceae* (*Rickettsiales*) |
| *Orientia tsutsugamushi Ikeda* | *Rickettsiaceae* (*Rickettsiales*) |
| *Rickettsia bellii RML369C* | *Rickettsiaceae* (*Rickettsiales*) |
| *Rickettsia canadensis McKiel* | *Rickettsiaceae* (*Rickettsiales*) |
| *Rickettsia felis URRWXCal2* | *Rickettsiaceae* (*Rickettsiales*) |
| *Rickettsia typhi Wilmington* | *Rickettsiaceae* (*Rickettsiales*) |
| *Wolbachia of Brugia malayi* | *Anaplasmataceae* (*Rickettsiales*) |
| *Wolbachia of Culex quinquefasciatus JHB* | *Anaplasmataceae* (*Rickettsiales*) |
| *Wolbachia of Drosophila melanogaster* | *Anaplasmataceae* (*Rickettsiales*) |
| *Citromicrobium bathyomarinum JL354* | *Erythrobacteraceae* (*Sphingomonadales*) |
| *Erythrobacter litoralis HTCC2594* | *Erythrobacteraceae* (*Sphingomonadales*) |
| *Erythrobacter NAP1* | *Erythrobacteraceae* (*Sphingomonadales*) |
| *Novosphingobium aromaticivorans DSM 12444* | *Sphingomonadaceae* (*Sphingomonadales*) |
| *Novosphingobium nitrogenifigens DSM 19370* | *Sphingomonadaceae* (*Sphingomonadales*) |
| *Novosphingobium PP1Y* | *Sphingomonadaceae* (*Sphingomonadales*) |
| *Sphingobium chlorophenolicum L1* | *Sphingomonadaceae* (*Sphingomonadales*) |
| *Sphingobium japonicum UT26S* | *Sphingomonadaceae* (*Sphingomonadales*) |
| *Sphingomonas S17* | *Sphingomonadaceae* (*Sphingomonadales*) |
| *Sphingomonas wittichii RW1* | *Sphingomonadaceae* (*Sphingomonadales*) |
| *Sphingopyxis alaskensis RB2256* | *Sphingomonadaceae* (*Sphingomonadales*) |
| *Zymomonas mobilis mobilis NCIMB 11163* | *Sphingomonadaceae* (*Sphingomonadales*) |

**Table S2B.** Bacterial taxa with 16S sequences only, which were added to the ‘combo’ dataset.

| **Name** | **Higher Taxon** |
| --- | --- |
| *Candidatus Captivus acidiprotistae ASL45* | *Holosporales* (*ord. nov.*) |
| *Holospora obtusa* | *Holosporales* (*ord. nov.*) |
| *Holosporaceae Env* | *Holosporales* (*ord. nov.*) |
| *Holosporaceae HB3* | *Holosporales* (*ord. nov.*) |
| *Holosporaceae HF NC 25* | *Holosporales* (*ord. nov.*) |
| *Holosporaceae M6A91* | *Holosporales* (*ord. nov.*) |
| *Holosporaceae Oh3123O11E* | *Holosporales* (*ord. nov.*) |
| *Holosporaceae soil 15* | *Holosporales* (*ord. nov.*) |
| *Caedibacter macronucleorum cm 30* | *Holosporales* (*ord. nov.*) |
| *Endosymbiont of Acanthamoeba polyphaga* | *Holosporales* (*ord. nov.*) |
| *Kopriimonas byunsanensis KOPRI 13522* | *Kiloniellales* (*v.* table S2E) |
| *Pelagibius litoralis CLUU02* | *Kiloniellales* (*v.* table S2E) |
| *Rhodospirillaceae YIM D82* | *Kiloniellales* (*v.* table S2E) |
| *Rhodovibrio salinarum* | *Kiloniellales* (*v.* table S2E) |
| *Kiloniella laminariae LD81T* | *Kiloniellales* |
| *Kordiimonas BME82* | *Kiloniellales* (*v.* table S2E) |
| *Alphaproteobacterium 14C01* | *Kiloniellales* (*v.* table S2E) |
| *Alphaproteobacterium 6ANG531* | *Kiloniellales* (*v.* table S2E) |
| *Alphaproteobacterium Ax36 F7* | *Kiloniellales* (*v.* table S2E) |
| *Bacterium DG1026* | *Kiloniellales* (*v.* table S2E) |
| *Endosymbiont of Chlamys farreri* | *Kiloniellales* (*v.* table S2E) |
| *Rhodobacterales Mfra C06* | *Kordiimondales* (*v.* table S2E) |
| *Rhodothalassium salexigens* | *Kordiimondales* (*v.* table S2E) |
| *Kordiimonas gwangyangensis GW145* | *Kordiimondales* |
| *Iodideoxidizing bacterium Mie8* | *Kordiimondales* (*v.* table S2E) |
| *Iodideoxidizing bacterium Q1* | *Kordiimondales* (*v.* table S2E) |
| *Magnetococcus M40* | *Magnetococcaceae* (*Magnetococcales*) |
| *Magnetococcus MRT130* | *Magnetococcaceae* (*Magnetococcales*) |
| *Magnetococcus MRT134* | *Magnetococcaceae* (*Magnetococcales*) |
| *Magnetococcus OTU15* | *Magnetococcaceae* (*Magnetococcales*) |
| *Magnetococcus rj12* | *Magnetococcaceae* (*Magnetococcales*) |
| *Oceanicaulis alexandrii HTCC2633* | potentially *Caulobacterales* (*v.* table S2E) |
| *Pelagibacteraceae HF4000APKG3108* | *Pelagibacterales* subclade II |
| *Pelagibacteraceae LD12* | *Pelagibacterales* subclade IIIb |
| *Pelagibacteraceae MB12A07* | *Pelagibacterales* subclade Ib |
| *Pelagibacteraceae MB21A02* | *Pelagibacterales* subclade II |
| *Afipia broomeae F186* | *Rhizobiaceae* (*Rhizobiales*) |
| *Pseudovibrio JE062* | potentially *Rhodospirillales* (*v.* table S2E) |
| *Agrobacterium tumefaciens C58UWash* | *Rhizobiaceae* (*Rhizobiales*) |
| *Agrobacterium vitis S4* | *Rhizobiaceae* (*Rhizobiales*) |
| *Ancalomicrobium adetum* | *Hyphomicrobiaceae* (*Rhizobiales*) |
| *Aurantimonas manganoxydans SI859A1* | *Rhizobiales* |
| *Bosea thiooxidans DSM 9653* | *Rhizobiales* |
| *Candidatus Liberibacter asiaticus psy62* | *Rhizobiaceae* (*Rhizobiales*) |
| *Defluvibacter lusatiae DSM11099T* | *Rhizobiales* |
| *Phyllobacterium myrsinacearum IAM 13584* | *Rhizobiales* |
| *Silicibacter TrichCH4B* | *Rhodobacteraceae* (*Rhodobacterales*) |
| *Roseomonas fauriae* | *Rhodospirillaceae* (*Rhodospirillales*) |
| *Terasakiella pusilla IFO 13613* | *Rhodospirillaceae* (*Rhodospirillales*) |
| *Defluvicoccus vanus Ben 114* | *Rhodospirillaceae* (*Rhodospirillales*) |
| *Inquilinus limosus AU476* | *Rhodospirillaceae* (*Rhodospirillales*) |
| *Insolitispirillum peregrinum peregrinum LMG 4340* | *Rhodospirillaceae* (*Rhodospirillales*) |
| *Novispirillum itersonii nipponicum* | *Rhodospirillaceae* (*Rhodospirillales*) |
| *Oceanibaculum indicum P24* | *Rhodospirillaceae* (*Rhodospirillales*) |
| *Phaeospirillum fulvum* | *Rhodospirillaceae* (*Rhodospirillales*) |
| *Rhodopila globiformis* | *Acetobacteraceae* (*Rhodospirillales*) |
| *Rhodospira trueperi* | *Rhodospirillaceae* (*Rhodospirillales*) |
| *Roseococcus thiosulatophilus* | *Acetobacteraceae* (*Rhodospirillales*) |
| *Skermanella parooensis* | *Rhodospirillaceae* (*Rhodospirillales*) |
| *Stella vacuolata DSM5901* | *Rhodospirillaceae* (*Rhodospirillales*) |
| *Telmatospirillum siberiense 264b1* | *Rhodospirillaceae* (*Rhodospirillales*) |
| *Thalassospira lucentensis* | *Rhodospirillaceae* (*Rhodospirillales*) |
| *Tistrella bauzanensis BZ78* | *Rhodospirillaceae* (*Rhodospirillales*) |
| *Tistrella mobilis* | *Rhodospirillaceae* (*Rhodospirillales*) |
| *Acidosphaera rubrifaciens* | *Acetobacteraceae* (*Rhodospirillales, v.* table S2E) |
| *Alphaproteobacterium 39* | *Rhodospirillaceae* (*Rhodospirillales, v.* table S2E) |
| *Candidatus Alysiosphaera europeae* | *Acetobacteraceae* (*Rhodospirillales, v.* table S2E) |
| *Craurococcus roseus NS130* | *Acetobacteraceae* (*Rhodospirillales, v.* table S2E) |
| *Elioraea tepidiphila TU7* | *Acetobacteraceae* (*Rhodospirillales, v.* table S2E) |
| *Endosymbiont 1b of Inanidrilus makropetalos* | *Rhodospirillaceae* (*Rhodospirillales, v.* table S2E) |
| *Endosymbiont of Olavius loisae 2* | *Rhodospirillaceae* (*Rhodospirillales, v.* table S2E) |
| *Geminicoccus roseus D23T* | *Acetobacteraceae* (*Rhodospirillales, v.* table S2E) |
| *Paracraurococcus ruber NS89* | *Acetobacteraceae* (*Rhodospirillales, v.* table S2E) |
| *Rhodospirillales HF0200 01O14* | *Rhodospirillaceae* (*Rhodospirillales, v.* table S2E) |
| *SAR116 OCS126* | *Rhodospirillaceae* (*Rhodospirillales, v.* table S2E) |
| *SAR116 OCS24* | *Rhodospirillaceae* (*Rhodospirillales, v.* table S2E) |
| *SAR116 OCS28* | *Rhodospirillaceae* (*Rhodospirillales, v.* table S2E) |
| *Candidatus Nicolleia massiliensis* | *Midichloriaceae* (*Rickettsiales*) |
| *Rickettsiales ID25L* | *Midichloriaceae* (*Rickettsiales*) |
| *Rickettsiales Montezuma* | *Midichloriaceae* (*Rickettsiales*) |
| *Candidatus Cryptoprodotis polytropus A10* | *Rickettsiaceae* (*Rickettsiales*) |
| *Candidatus Xenohaliotis californiensis* | *Anaplasmataceae* (*Rickettsiales, v.* table S2E) |
| *Endosymbiont of Diophrys sp* | *Rickettsiaceae* (*Rickettsiales, v.* table S2E) |
| *Rickettsiales lakePohlsee 4* | *Rickettsiaceae* (*Rickettsiales, v.* table S2E) |
| *Sneathiella BFLP8* | *Sneathiellales* |
| *Sneathiella chinensis CBMAI 737* | *Sneathiellales* |
| *alphaproteobacterium SGPZ642* | *Sneathiellales* (*v.* table S2E) |

**Table S2C.** Mitochondria used in the trees.

| **Mitochondrial host** | **Host grouping** |
| --- | --- |
| *Acanthamoeba castellanii* | amoeba |
| *Arabidopsis thaliana* | plant (thale cress) |
| *Chaetosphaeridium globosum* | green alga |
| *Chara vulgaris* | green alga |
| *Chattonella marina* | raphidophyte |
| *Chlorokybus atmophyticus* strain SAG 4880 | green alga |
| *Chondrus crispus* | red alga (carrageen moss) |
| *Cyanidioschyzon merolae* | red alga |
| *Cyanophora paradoxa* strain CCMP 329 | glaucophyte |
| *Dictyostelium discoideum* | amoeba (slime mold) |
| *Glaucocystis nostochinearum* | glaucophyte |
| *Hartmannella vermiformis* | amoeba |
| *Malawimonas jakobiformis* | unicellular eukaryote |
| *Marchantia polymorpha* | plant (liverwort) |
| *Megaceros aenigmaticus* | plant (hornwort) |
| *Naegleria gruberi* | amoebo-flagellate |
| *Nephroselmis olivacea* | green alga |
| *Ochromonas danica* | golden alga |
| *Phytophthora infestans* | Oomycete |
| *Pilayella littoralis* | brown alga |
| *Polysphondylium pallidum* | amoeba (slime mould) |
| *Prototheca wickerhamii* 263-11 | green alga |
| *Reclinomonas americana* | unicellular eukaryote |
| *Saprolegnia ferax* strain ATCC 36051 | Oomycete (water mould) |

**Table S2D.** Mitochondria that were initially included, but were rejected due to redundancy, inadequate sequence length or poor ARB aligner score.

| **Mitochondrial host** | **Host grouping** |
| --- | --- |
| *Beta vulgaris subsp. vulgaris* | plant (beetroot) |
| *Zea mays strain NB* | plant (corn) |
| *Vitis vinifera* cultivar Pinot noir | plant (grape) |
| *Pleurozia purpurea* | plant (liverwort) |
| *Physcomitrella patens* | plant (moss) |
| *Carica papaya* | plant (papaya) |
| *Brassica napus* | plant (rapeseed) |
| *Oryza sativa Japonica Group* | plant (rice) |
| *Nicotiana tabacum* | plant (tobacco) |
| *Citrullus lanatus* | plant (watermelon) |
| *Triticum aestivum* | plant (wheat) |
| *Oryza rufipogon* | plant (wild rice) |
| *Hyaloraphidium curvatum* | green alga |
| *Micromonas sp. RCC299* | green alga |
| *Oltmannsiellopsis viridis* | green alga |
| *Ostreococcus tauri* | green alga |
| *Pseudendoclonium akinetum* | green alga |
| *Chlamydomonas eugametos* | green alga |
| *Synedra acus* | diatom alga |
| *Desmarestia viridis* | brown alga (kelp) |
| *Porphyra purpurea* | brown alga (laver) |
| *Laminaria digitata* | brown alga (oarweed) |
| *Phytophthora ramorum* | oomycete (water mould) |
| *Phytophthora sojae* | oomycete (water mould) |
| *Pycnococcus provasolii* | ciliate protozoan |
| *Paramecium aurelia* | ciliate protozoan |
| *Tetrahymena malaccensis strain MP75* | ciliate protozoan |
| *Tetrahymena paravorax strain RP* | ciliate protozoan |
| *Tetrahymena pigmentosa strain UM1060* | ciliate protozoan |
| *Tetrahymena pyriformis* | ciliate protozoan |
| *Tetrahymena thermophila strain SB210* | ciliate protozoan |
| *Hemiselmis andersenii strain CCMP 644* | cryptomonad |
| *Rhodomonas salina* | cryptomonad |
| *Trichloplax adhaerens* | animal (basal) |
| *Geodia neptuni* | animal (glass sponge) |
| *Montastraea annularis* | animal (coral) |
| *Aurelia aurita* | animal (jellyfish) |
| *Monosiga brevicollis* | animal (choanoflagellate) |
| *Aphrocallistes vastus* | animal (sponge) |
| *Ciona intestinalis* | animal (sea squirt) |
| *Rotaria rotatoria* | animal (rotifer) |
| *Architeuthis dux* | animal (giant squid) |
| *Drosophila melanogaster* | animal (fruit fly) |
| *Limulus polyphemus* | animal (horseshoe crab) |
| *Ophiopholis aculeata* | animal (starfish) |
| *Balanoglossus carnosus* | animal (acorn worm) |
| *Homo sapiens* | animal (human) |
| *Xiphinema americanum* | animal (nematode) |
| *Caenorhabditis elegans* | animal (nematode) |
| *Moniliophthora perniciosa* | fungus (Witches' broom) |
| *Saccharomyces cerevisiae* | fungus (yeast) |
| *Gigaspora rosea* | fungus (basal) |
| *Blastocladiella emersonii* | fungus (aquatic fungus) |

**Table S2E.** The previous classifications of several taxa did not match the phylogenies we saw. In our analyses we applied the following changes, in order for various clades to be monophyletic.

| **Taxon** | **Previous classification** | **These results** | **Notes** |
| --- | --- | --- | --- |
| *Ahrensia sp. R2A130* | *Rhodobacterales* | *Caulobacterales* |  |
| *Kopriimonas byunsanensis* | *Kopriimonadales* | *Kiloniellales* | *Kiloniellales* and *Kopriimonadales* are synonymous. |
| *Labrenzia aggregata and L. alexandrii* | *Rhodobacterales* | *Rhizobiales* |  |
| *Pelagibius litoralis* | *Rhodospirillales* | *Kiloniellales* |  |
| *Pseudovibrio sp. JE062* | *Rhodobacterales* | *Rhizobiales* |  |
| *Unclassified Rhodobacterales sp. Mfra C06* | *Rhodobacterales* | *Kordiimonadales* |  |
| *Unclassified Rhodospirillales sp. YIM D82* | *Rhodospirillales* | *Kiloniellales* |  |
| *Rhodothalassium salexigens* | *Rhodobacterales* / *Rhodothalassiales* | *Kordiimonadales* |  |
| *Rhodovibrio salinarum* | *Rhodospirillales* | *Kiloniellales* |  |
| *Roseibium sp*. Trich SKD4 | *Rhodobacterales* | *Rhizobiales* |  |
| *Roseomonas fauriae* | *Acetobacteraceae* | *Rhodospirillaceae* |  |
| *Terasakiella pusilla* | *Rhizobiales* | *Rhodospirillales* |  |
| *Hirschia baltica* | *Rhodobacterales* (NCBI) / *Caulobacterales* (LPSN) | *Caulobacterales* | NCBI / LPSN mismatch. |
| *Hyphomonas neptunium* | *Rhodobacterales* (NCBI) / *Caulobacterales* (LPSN) | *Caulobacterales* | NCBI / LPSN mismatch. |
| *Midichloria mitochondrii, Nicolleia massiliensis*, strain “Montezuma” | *Unclassified Rickettsiaceae* | *Midichloriaceae* | Montagna *et al.* (2013). |
| *Maricaulis maris* and *Oceanicaulis axlexadrii* | *Rhodobacterales* | *Uncertain* | Could not classify with full certainty. Potentially in the *Caulobacterales*. |
| *Parvibaculum lavamentivorans* | *Rhizobiales* | *Uncertain* | Could not classify with full certainty, but probably in the *Rhizobiales*. |
| *Caedibacter caryophilus and C. macronucleorum* | *Rickettsiales (NCBI) /*  *Thiotrichales, Gamma-proteobacteria (LPSN)* | *Holosporales* | Unclassified at family level in NCBI. Classification in LPSN predates Bergey’s manual. |
| *Xenohaliotis californiensis* | *Unclassified Rickettsiales* | *Anaplasmataceae, Rickettsiales* |  |
| *Alysiosphaera europeae, Craurococcus roseus, Elioraea tepidiphila, Geminococcus roseus* | *Unclassified Rhodospirillales* | *Acetobacteraceae, Rhodospirillales* |  |
